# Expeditious Total Synthesis of Hemiasterlin through a Convergent Multicomponent Strategy and Its Use in Targeted Cancer Therapeutics

**DOI:** 10.1002/anie.202010090

**Published:** 2020-10-12

**Authors:** Jiraborrirak Charoenpattarapreeda, Stephen J. Walsh, Jason S. Carroll, David R. Spring

**Affiliations:** ^1^ Department of Chemistry University of Cambridge Lensfield Road Cambridge CB2 1EW UK; ^2^ Cancer Research (UK) Cambridge Institute University of Cambridge Robinson Way Cambridge CB2 0RE UK

**Keywords:** antibody–drug conjugates, hemiasterlin, multicomponent reactions, targeted therapeutics, total synthesis

## Abstract

Hemiasterlin is an antimitotic marine natural product with reported sub‐nanomolar potency against several cancer cell lines. Herein, we describe an expeditious total synthesis of hemiasterlin featuring a four‐component Ugi reaction (Ugi‐4CR) as the key step. The convergent synthetic strategy enabled rapid access to taltobulin (HTI‐286), a similarly potent synthetic analogue. This short synthetic sequence enabled investigation of both hemiasterlin and taltobulin as cytotoxic payloads in antibody–drug conjugates (ADCs). These novel ADCs displayed sub‐nanomolar cytotoxicity against HER2‐expressing cancer cells, while showing no activity against antigen‐negative cells. This study demonstrates an improved synthetic route to a highly valuable natural product, facilitating further investigation of hemiasterlin and its analogues as potential payloads in targeted therapeutics.

## Graphical abstract

Hemiasterlin, a highly cytotoxic marine natural product (see structure), was synthesised by a multicomponent approach with a longest linear sequence of 10 steps. Its use in a targeted therapeutic was demonstrated by incorporation as a payload in an antibody–drug conjugate (ADC), which showed mid‐picomolar cytotoxicity against HER2‐expressing cancer cell lines.

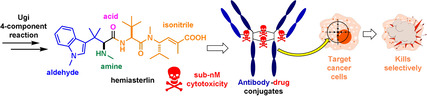

Hemiasterlins (**1**–**4**),[^1^, ^2^, ^3^, ^4^] criamides (**5**, **6**)^[2]^, and milnamide A (**7**),^[5]^ are a family of cytotoxic tripeptide natural products isolated from marine sponges (Figure 1). Hemiasterlin (**1**), the most toxic of the family, acts as an antimitotic agent by disrupting microtubule dynamics causing mitotic arrest and cell death with low‐ to sub‐nanomolar potencies against several cancer cell lines.[^6^, ^7^, ^8^, ^9^] Several studies have detailed investigations into discerning the pharmacophore of hemiasterlin with the aim of generating efficacious anticancer agents. These efforts lead to the development of taltobulin (also known as HTI‐286, **8**),[^8^, ^10^] a synthetic analogue of hemiasterlin, which was also shown to bind at the vinca domain between the α‐ and β‐subunits of tubulin.[^11^, ^12^, ^13^] In the early 2000s, HTI‐286 advanced to Phase II clinical trials for the treatment of non‐small cell lung cancer (NSCLC).^[14]^ Its development was halted by Wyeth for business reasons.^[15]^

**Figure 1:**
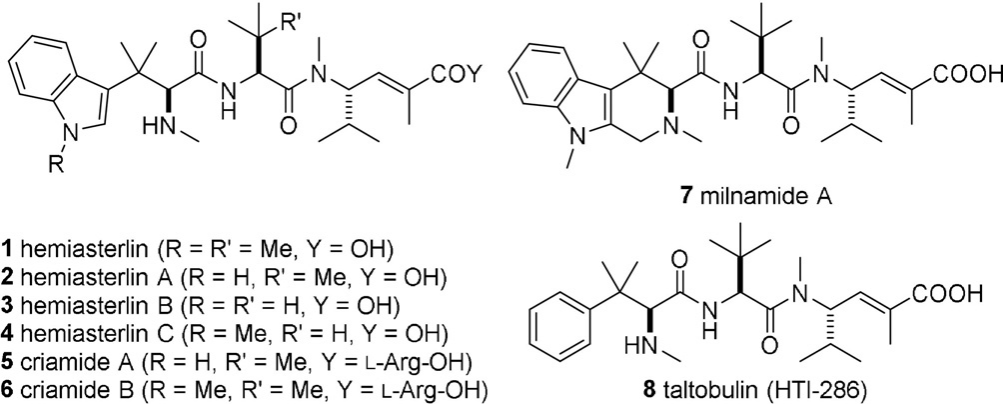
Hemiasterlin (**1**), its family of marine natural products, and a synthetic analogue taltobulin (HTI‐286, **8**).

Three synthetic strategies for the total synthesis of hemiasterlin have been reported. Andersen et al. used Evans’ oxazolidinone to produce an enantiomerically pure tetramethyltryptophan unit and the route was highly linear with a longest linear sequence (LLS) of 17 steps (total 23 steps).^[16]^ Vedejs and Kongkittingam utilised an *N*‐benzothiazole‐2‐sulfonyl (Bts) protecting group for peptide bond formation and (*R*)‐2‐phenylglycinol chiral auxiliary for synthesising the tetramethyltryptophan subunit with an LLS of 13 steps (total 20 steps).^[17]^ Finally, Lindel and co‐workers employed organocatalytic α‐hydrazination using a proline‐based tetrazole catalyst in their total synthesis with a 15‐step LLS (total 21 steps).^[18]^

The highly cytotoxic properties of hemiasterlin and its reported analogues make them attractive candidates for use in targeted therapeutics, such as antibody–drug conjugates (ADCs). ADCs utilise the exquisite targeting ability of antibodies to deliver cytotoxic payloads to specific cell types, such as cancer cells.[^19^, ^20^] This strategy has witnessed renewed clinical success and interest in recent years, with nine ADCs now approved by the US Food and Drug Administration and more than 80 others undergoing clinical evaluation.[^21^, ^22^, ^23^] Key to the success of any ADC is the potency of the drug payload; only a tiny fraction of the administered ADC reaches and is internalised by its target cells.^[19]^ Therefore, the limited number of intracellular drug molecules must be sufficiently potent (typically sub‐nanomolar in vitro IC_50_) to effect the desired cell death. This requirement means that most ADC payloads are large, lipophilic species with difficult and laborious synthetic routes, increasing the cost of an already expensive development process. We hypothesised that if synthetic access to hemiasterlin could be improved, it could potentially serve as a useful payload for the ADC field due to its high potency, relatively small size and hydrophilic zwitterionic structure. Indeed, STRO‐002, an ADC developed by Sutro Biopharm, uses the hemiasterlin analogue 3‐aminophenyl hemiasterlin as the payload. This ADC is currently undergoing a Phase I clinical trial for the treatment of ovarian and endometrial cancer (clinical trial number: NCT03748186).^[24]^

To commence investigations, we envisaged that the synthetic route to hemiasterlin could be simplified via a convergent, multicomponent approach (Scheme 1). Previously Lesma et al. has reported the use of a four‐component Ugi reaction (Ugi‐4CR) to generate analogues of hemiasterlin with the l‐*tert*‐leucine central amino acid replaced by l‐valine.^[25]^ In this vein, it was hypothesised that a similar Ugi‐4CR could be used to generate dipeptide **9** from simple starting materials. Coupling of this dipeptide to unsaturated γ‐amino ester **10**, accessible from *N*‐Boc‐*N*‐methylvaline via a reported procedure,^[8]^ would then complete the synthesis of the desired natural product.

**Scheme 1:**
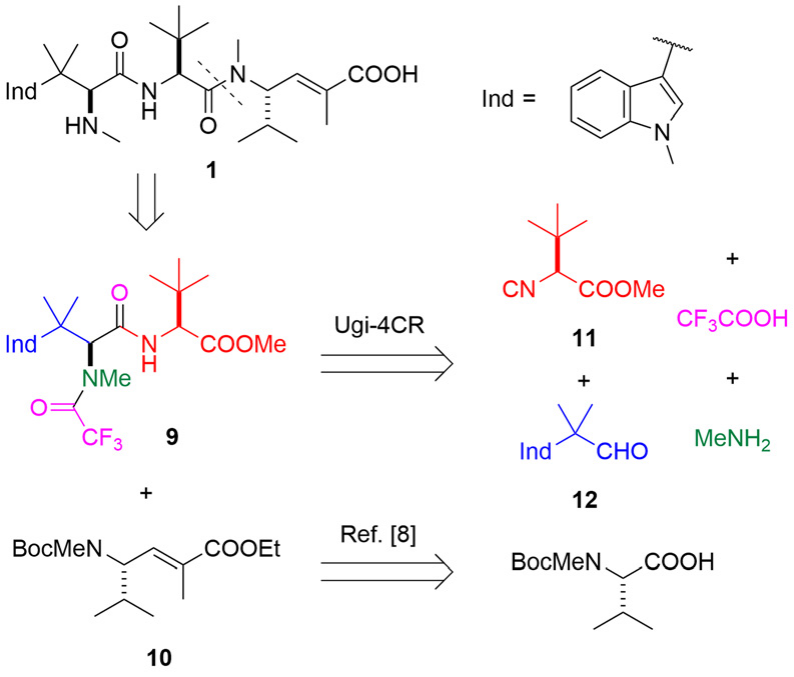
Retrosynthetic analysis of hemiasterlin (**1**).

Accordingly, our synthesis of fragment **11** commenced with the conversion of methyl ester of l‐*tert*‐leucine to formamide **13** by heating to reflux in ethyl formate (Scheme 2 a).^[26]^ Triphosgene‐mediated dehydration proceeded smoothly to produce isonitrile **11** in excellent yield (91 % over two steps).^[27]^ The aldehyde fragment **12** was synthesised in four steps according to the procedure from Nieman et al. (see SI for synthesis).^[8]^ Briefly, starting from methyl (1*H*‐indol‐3‐yl)acetate, trimethylation in two steps, followed by a reduction by DIBAL‐H gave homobenzylic alcohol **S4**. Subsequent Ley–Griffith oxidation produced aldehyde **12** (70 % over four steps).^[28]^ With isonitrile **11** and aldehyde **12** in hand, the Ugi‐4CR was undertaken via their reaction with methylamine and trifluoroacetic acid in the presence of 3 Å molecular sieves, a procedure adapted from Lesma et al. (Scheme 2 b).^[25]^ A separable mixture of diastereomers **9 a** and **9 b** were obtained in good yield (70 %, *dr* 1:1.3), of which we identified the absolute stereochemistry by X‐ray crystallography analysis.^[50]^ Trifluoroacetic acid was used as the resulting *N*‐trifluoroacetamide is stable under mild ester hydrolysis condition and can also be removed orthogonally using reductive methods.[^29^, ^30^] It was found that the use of 3 Å molecular sieves were critical to reaction progression; alternative drying reagents resulted in the formation of undesired side products (Scheme S1). Amide coupling of the two fragments, dipeptide (**9 a** or **9 b**) with amino ester **14** (see SI for synthesis^[8]^) was then attempted. While Lesma and co‐workers could perform the fragment coupling to generate their tripeptide library with the use of HBTU as the coupling agent,^[25]^ unfortunately, our screening of various amide coupling agents did not afford formation of the desired product **15** (Table S1). Complex reaction mixtures or apparent intramolecular cyclisation of dipeptide **9 a** or **9 b** to form an undesired oxazolone were observed under all conditions tested. Given the success of the Ugi reaction to generate fragment **9**, it was hypothesised that an analogous Ugi reaction could facilitate fragment coupling to complete the natural product synthesis. Favourably, isonitrile **17** was identified as the key intermediate for this strategy, which could be accessed from common intermediates synthesised in the initial route.

**Scheme 2:**
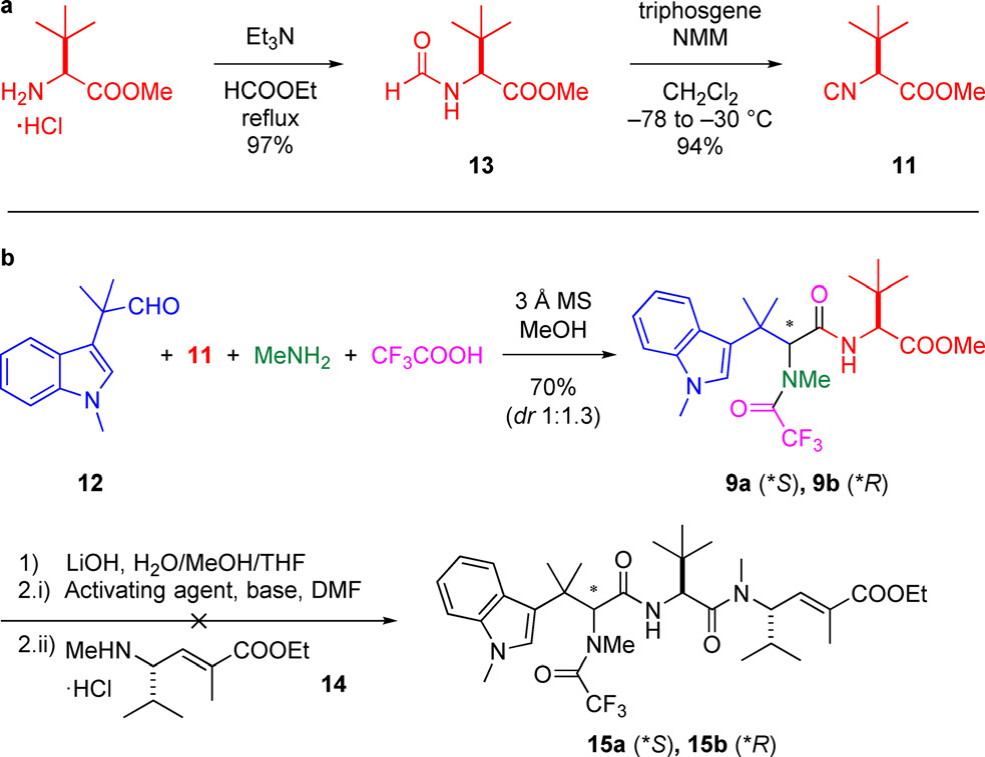
a) Synthesis of isonitrile **11**. b) The four‐component Ugi reaction and the subsequent failed fragment amide coupling. NMM=*N*‐methylmorpholine, MS=molecular sieves, DMF=*N*,*N*‐dimethylformamide.

The synthesis of isonitrile **17** began by Boc‐deprotection of **10** and amide coupling of the free amine with Boc‐Tle‐OH, affording dipeptide **16** in excellent yield (Scheme 3 a). Subsequent Boc‐deprotection, formamide formation, and triphosgene‐mediated dehydration^[27]^ produced the required isonitrile **17** (65 % yield over five steps). Next, the Ugi‐4CR to complete the synthesis was attempted, which resulted in only 20 % yield of the desired product (Table S4). A significant amount of an oxazole by‐product was observed by LCMS analysis of the reaction mixture. Isocyanoacetamides have been used in three‐component Ugi‐type reactions for synthesis of oxazoles due to the higher basicity of the amide oxygen, which promotes intramolecular cyclisation (Scheme S2).^[31]^ We hypothesised that by increasing the concentration of trifluoroacetate anion in the reaction, we may be able to outcompete this intramolecular process. Pleasingly, addition of CF_3_COONa increased the overall yield of the Ugi‐4CR to 73 % (*dr* 1:1.4, Scheme 3 a). X‐ray crystallography analysis of **15 a** was used to determine its absolute stereochemistry, which corresponded to the stereochemistry of hemiasterlin (Scheme 3 b).^[50]^

**Scheme 3:**
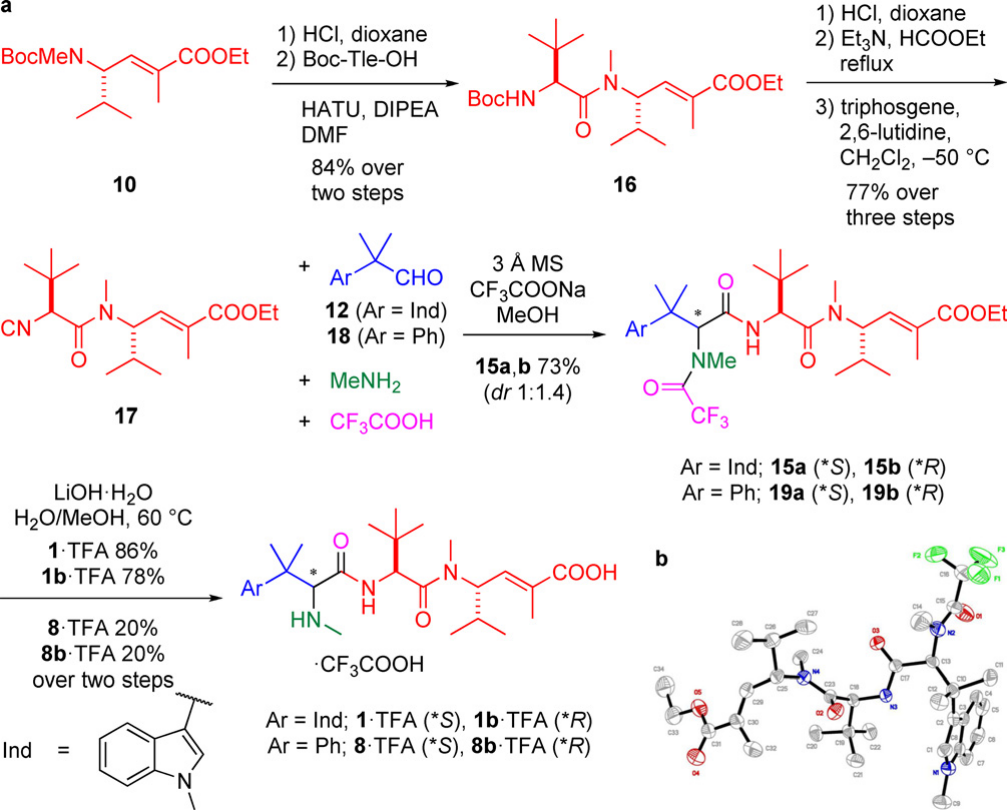
a) Final route to the synthesis of hemiasterlin (**1**) and taltobulin (**8**). b) X‐ray crystal structure of intermediate **15 a**.^[50]^ HATU=hexafluorophosphate azabenzotriazole tetramethyluronium, DIPEA=diisopropylethylamine, TFA=trifluoroacetic acid.

The low diastereoselectivity observed in the Ugi‐4CR step was similarly experienced in many previous total syntheses which utilised isocyanide‐based multicomponent reactions (IMCRs),[^32^, ^33^, ^34^, ^35^, ^36^] where notable exceptions were when cyclic imines were used.[^37^, ^38^] Consequently, several groups have reported the use of chiral phosphoric acids (CPAs) to induce enantioselectivity for IMCRs.[^39^, ^40^, ^41^, ^42^] Thus, we attempted a screen of CPAs to improve the diastereoselectivity, but no improvement was observed (Table S2).

Lastly, the Ugi products **15** were hydrolysed to give hemiasterlin **1** and its epimer **1 b** in 86 % and 78 % yield, respectively. Taltobulin **8** and its epimer **8 b** were also synthesised via the same synthetic route (Scheme 3 a), demonstrating the amenability of the route for analogue synthesis. Hemiasterlin and taltobulin were each synthesised in 14 and 12 total steps, respectively (both with an LLS of 10), in good overall yield.

Having successfully developed a superior synthetic route to both hemiasterlin and taltobulin, we next wanted to investigate the utility of these natural products in ADCs. First, it was necessary to modify these warheads with bioconjugation linkers, to facilitate attachment to an antibody. We have recently reported the development of divinylpyrimidine (DVP) linkers as efficient reagents for the selective and stable modification of antibodies via disulfide rebridging.[^43^, ^44^] The cathepsin‐cleavable Val‐Ala‐PABC was incorporated in the linker design as it was deemed essential to release an unmodified drug from the antibody.^[45]^ Finally, to enable a convergent synthesis, it was hypothesised that modification of the warhead moiety with an azide and the DVP linker with an alkyne would allow the use of a copper‐catalysed azide–alkyne cycloaddition (CuAAC) to complete the synthesis of the desired linker–drugs.

Accordingly, deprotection of Alloc‐Val‐Ala‐PABA (**S8**) was achieved via treatment with Pd(PPh_3_)_4_, followed by amide coupling of the free amine with N_3_‐PEG_4_‐COOH, yielding azide **S10** (Scheme S3). Activation of the benzyl alcohol was achieved via reaction with bis(4‐nitrophenyl)carbonate to generate mixed carbonate **22** (56 % yield over three steps). It was then found that the presence of a free carboxylic acid on hemiasterlin or taltobulin hindered carbamate formation. Thus, selective removal of the trifluoroacetamide from **15 a** and **19 a** was achieved using NaBH_4_ reduction (59 % for **20** and 99 % for **21**, Scheme 4 a). The free amines were then reacted with activated carbonate **22** to form the corresponding carbamates **23** and **24**. It was then hypothesised that an alkynyl DVP could be prepared via a strategy resembling solid‐phase peptide synthesis. Introduction of a reactive alkyne could be achieved through propargyl glycine (propargylGly), while incorporation of polyethylene glycol (PEG) chains and glutamic acid residues would improve the aqueous solubility of the linker–drug and decrease the hydrophobicity and aggregation propensity of the resultant ADCs. Thus, peptidomimetic H‐PEG_2_‐Glu_3_‐PEG_2_‐propargylGly‐NH_2_⋅TFA (**S11**) was synthesised using a standard Fmoc‐protecting‐group protocol. Following cleavage from the resin and purification, amide coupling of amine **S11** with DVP–carboxylic acid **S12** produced the desired linker **25** (Scheme S4). CuAAC reaction of DVP–alkyne **25** with azides **23** and **24** was followed by ester hydrolysis to give the final linker–drug compounds **26** and **27** after reverse‐phase chromatography purification (Scheme 4 a).

**Scheme 4:**
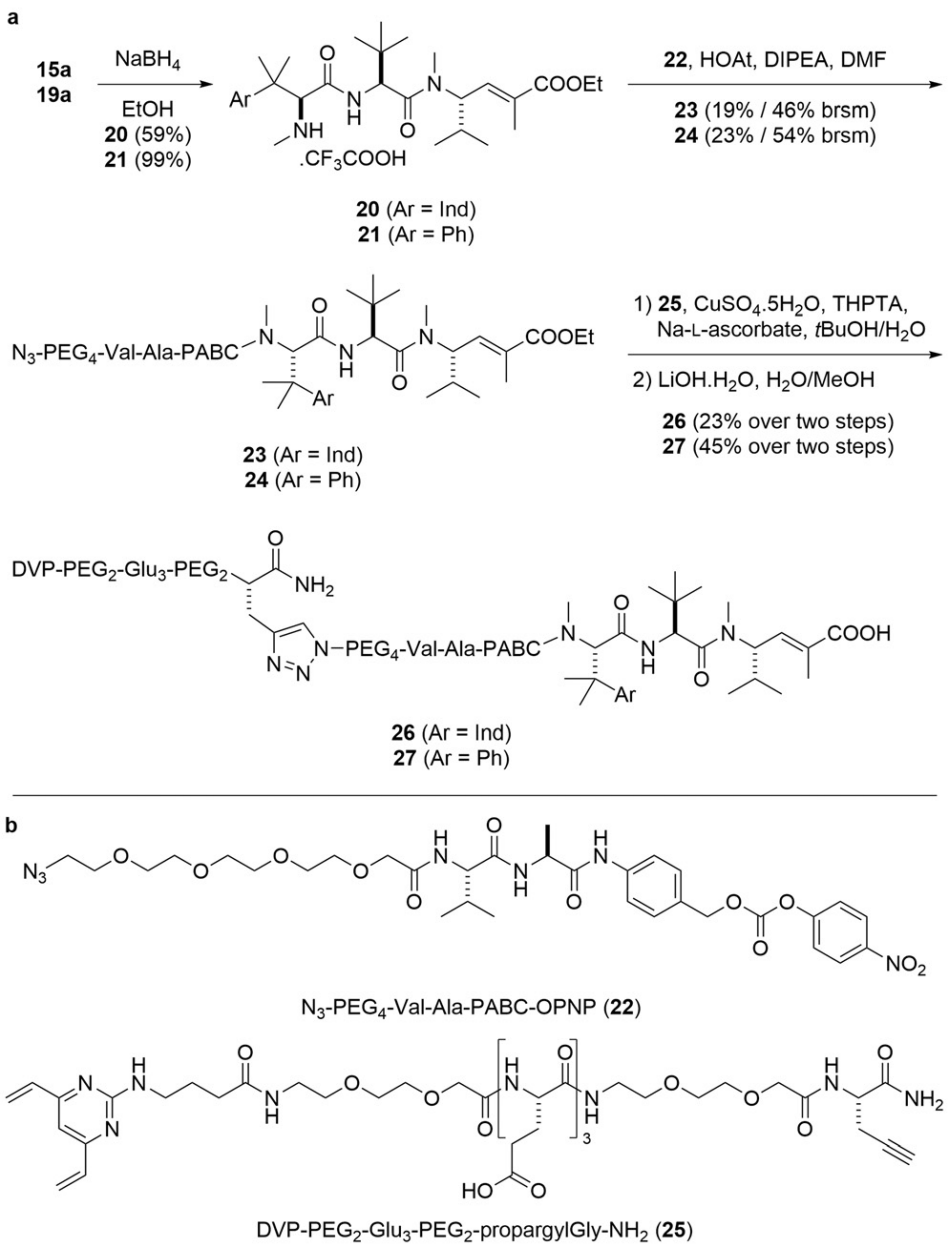
a) Synthesis of linker–drug compounds **26** and **27**. b) Structures of **22** and **25** (see the Supporting Information for the syntheses). PABC=*p*‐aminobenzyloxycarbonyl, HOAt=1‐hydroxy‐7‐azabenzotriazole, THPTA=tris(3‐hydroxypropyltriazolylmethyl)amine, PNP=*p*‐nitrophenyl.

Trastuzumab is an FDA‐approved monoclonal antibody targeting HER2, a transmembrane receptor that is overexpressed in 20–30 % breast cancers. Trastuzumab also constitutes the antibody component in two of the marketed ADCs (Kadcyla^®^ and Enhertu^®^).[^21^, ^46^, ^47^, ^48^] Trastuzumab was chosen for this study to enable comparison of hemiasterlin‐based ADCs with other reported trastuzumab ADCs loaded with alternative payloads. To commence ADC synthesis, the four interchain disulfides in trastuzumab were reduced via treatment with tris(2‐carboxyethyl)phosphine hydrochloride (TCEP) for 1 hour at 37 °C, revealing eight reactive thiols. The reduced antibody was then reacted with the DVP linker–drug compounds **26** and **27** for 4 hours at 37 °C. Pleasingly, LCMS and SDS‐PAGE analysis revealed >95 % conversion to the rebridged antibody species **ADC 1** and **ADC 2** with a loading of four drug molecules per antibody (Scheme 5, Figures S1—S3). The SDS‐PAGE analysis also showed significant formation of the half‐antibody species whereby the hinge disulfides did not undergo interchain rebridging; instead, non‐native intrachain cross‐linking of the reduced heavy chain cysteines occurred (Scheme 5 b). This is in line with the usage of DVP as a rebridging linker.^[43]^ In addition, size‐exclusion chromatography analysis demonstrated that both ADCs had ≥99.5 % monomeric content, confirming minimal aggregation propensity with this linker–payload (Figure S4, Table S3).

**Scheme 5:**
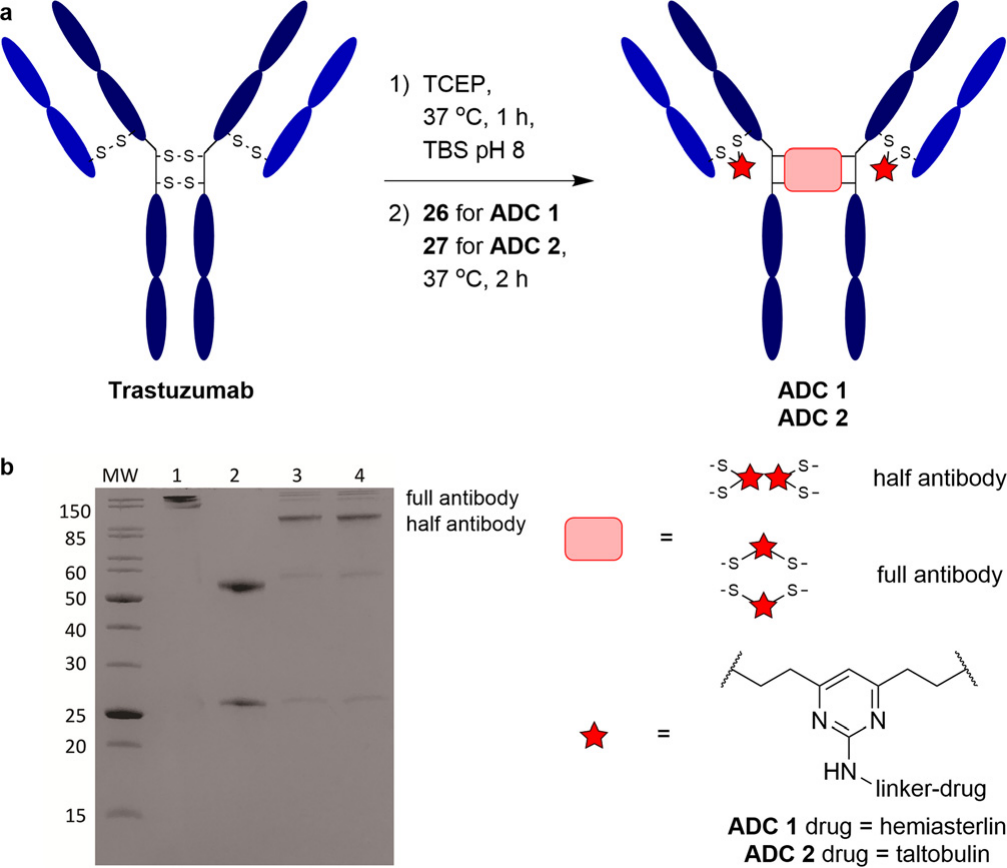
a) Synthesis of **ADC 1** and **ADC 2**. b) SDS‐PAGE analysis of the two ADCs; lane 1 is nonreducing, lanes 2–4 are reducing. Lanes: MW=molecular‐weight marker, 1) trastuzumab, 2) reduced trastuzumab, 3) **ADC 1**, 4) **ADC 2**. TCEP=tris(2‐carboxyethyl)phosphine, TBS=Tris‐buffered saline (TrisHCl 25 mM, NaCl 25 mM, EDTA 0.5 mM, pH 8).

To investigate the biological activity of hemiasterlin, taltobulin and their corresponding ADCs, their effect on the cell viability of both HER2‐positive (SKBR3, BT474) and HER2‐negative (MCF7) cell lines was determined (Figure 2, Table 1). Hemiasterlin exhibited sub‐nanomolar cytotoxicity against all cell lines, whilst the potency of taltobulin was approximately one order of magnitude lower.^[9]^ Pleasingly, both **ADC 1** and **ADC 2** displayed exquisite cytotoxicity against SKBR3 and BT474 cells, comparable to that reported for an analogous cathepsin‐cleavable trastuzumab–MMAE ADC (Table S5).[^44^, ^49^] Furthermore, both ADCs had negligible activity against MCF7 cells at the concentrations tested. These combined data suggest that both hemiasterlin and taltobulin have the potential to serve as cytotoxic payloads in the development of targeted therapeutics and that they can generate equivalent potency as clinically validated payloads.

**Figure 2:**
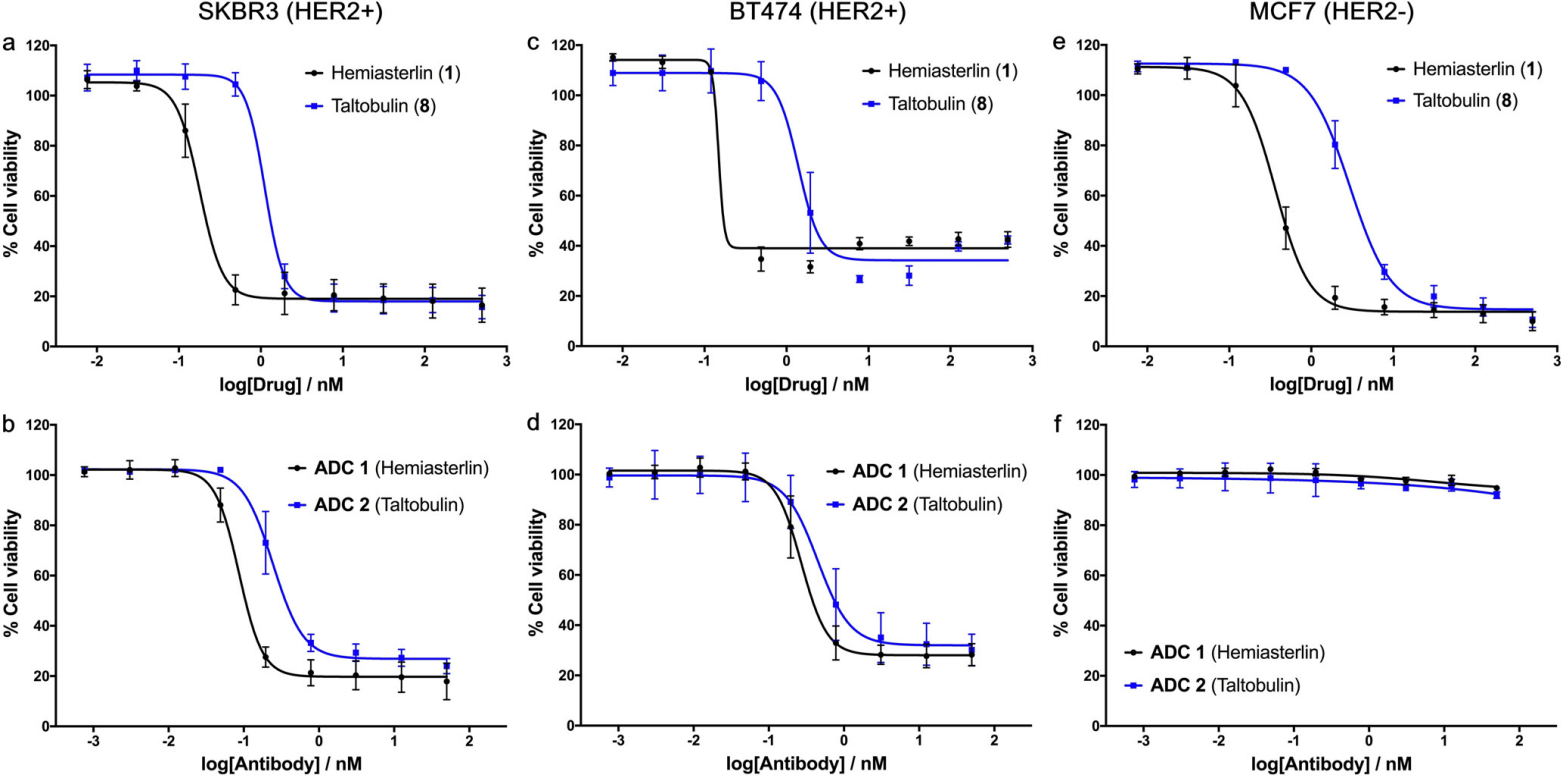
Cellular viability assays of **1**, **8**, **ADC 1**, and **ADC 2** in a, b) HER2‐positive SKBR3 cells, c, d) HER2‐positive BT474 cells, and e, f) HER2‐negative MCF7 cells.

**Table 1:** In vitro cellular evaluation of **1**, **8**, **ADC 1** and **ADC 2**.

IC_50_ [nM]^[a]^	SKBR3 (HER2+)	BT474 (HER2+)	MCF7 (HER2−)
hemiasterlin (**1**)	0.18	0.15	0.37
taltobulin (**8**)	1.12	1.40	3.00
**ADC 1**	0.086	0.27	>50
**ADC 2**	0.25	0.45	>50

[a] Each data point is an average of independent triplicates.

In conclusion, the total synthesis of hemiasterlin has been accomplished via a four‐component Ugi reaction with a longest linear sequence of 10 steps, in 11 % overall yield. Our improved synthetic approach allows for simple analogue exploration on the *N*‐terminus of the molecule, which was demonstrated by the synthesis of taltobulin, a synthetic analogue of hemiasterlin. ADCs synthesised from the two compounds showed exceptionally potent and selective bioactivity, similar to that of an analogous MMAE ADC. With its routine synthesis and high cytotoxicity, this study paves the way for the future use of hemiasterlin and its analogues as payloads in ADC therapeutics. Furthermore, this represents the first documented use of hemiasterlin and its ADC in the treatment of breast cancer, showcasing its potential in the treatment of this disease.

## Conflict of interest

The authors declare no conflict of interest.

## Supporting information

As a service to our authors and readers, this journal provides supporting information supplied by the authors. Such materials are peer reviewed and may be re‐organized for online delivery, but are not copy‐edited or typeset. Technical support issues arising from supporting information (other than missing files) should be addressed to the authors.

Supplementary
