# Supplementary material for: Expeditious Total Synthesis of Hemiasterlin through a Convergent Multicomponent Strategy and Its Use in Targeted Cancer Therapeutics
Source: Angew Chem Int Ed Engl. 2020 Oct 12;59(51):23045–50. doi: 10.1002/anie.202010090 (PMC7756509; doi:10.1002/anie.202010090)
Supplement: Supplementary file 1 — Supplementary [file ANIE-59-23045-s001.pdf]

Angewandte Chemie
Eine Zeitschrift der Gesellschaft Deutscher Chemiker

Supporting Information

Expeditious Total Synthesis of Hemiasterlin through a Convergent Multicomponent Strategy and Its Use in Targeted Cancer Therapeutics

*Jiraborrirak Charoenpattarapreeda, Stephen J. Walsh, Jason S. Carroll, and David R. Spring**

anie_202010090_sm_miscellaneous_information.pdf

Table of Contents

Table of Contents 2
Experimental Procedures 3
General Information........ 3
Synthesis of hemiasterlin and taltobulin 5
Linker-drug synthesis 18
Reaction condition screening 25
Fragment amide coupling........ 25
Attempt at improving diastereoselectivity of the Ugi reaction 27
ADC synthesis........ 28
Size Exclusion Chromatography (SEC) 32
Cellular viabilty assays 33
Crystallographic Data 34
Supplementary Figures and Tables 37
NMR Spectra........ 40
Analytical HPLC traces........ 84
References 91

Experimental Procedures

General Information

All reactions were carried out under nitrogen atmosphere using oven-dried glassware at room temperature unless otherwise stated. Temperatures of -78 °C were maintained using a dry ice acetone bath. Temperatures of 0 °C were maintained using an ice-water bath. Room temperature (rt) refers to ambient temperatures. All reagents were used as received from commercial sources or prepared as described in the literature unless otherwise stated. Dichloromethane (CH_2Cl_2), methanol (MeOH) and toluene were distilled from calcium hydride. Tetrahydrofuran (THF) was dried using sodium wire and distilled from a mixture of calcium hydride and lithium aluminium hydride with triphenylmethane as indicator. Diethyl ether (Et_2O) was distilled from a mixture of calcium hydride and lithium aluminium hydride. Ethyl acetate (EtOAc) was distilled before use; 40-60 petroleum ether was distilled before use and refers to the fraction between 40-60 °C. Acetonitrile (MeCN), anhydrous dimethylformamide (DMF) and *tert*-butyl alcohol (*t*BuOH) were purchased from commercial sources and used without further purification. Reactions were monitored by thin layer chromatography (TLC) using pre-coated Merck glass backed silica gel 60 F_{254} plates and visualised by quenching of UV fluorescence (λ_{Max} = 254 nm) or by staining with potassium permanganate. Retention factors (R_f) are quoted to 0.01.

Flash column chromatography was carried out using slurry-packed Merck 9385 Kieselgel 60 SiO_2 (230-400 mesh) or Combiflash Rf200 automated chromatography system with Redisep® normal-phase silica flash columns (35-70 µm) or Redisep® reverse-phase C18-silica flash columns (20-40 µm). Yields refer to chromatographically and spectroscopically pure compounds unless otherwise stated.

Melting points (mp) were obtained using a Büchi Melting Point B-545 or Gallenkamp MPD350.BM2.5 melting point apparatus and are uncorrected.

Optical rotations were recorded on an Anton-Paar MCP polarimeter. $[\alpha]_D$ values are reported in deg dm^{-1} cm^3 g^{-1} at 589 nm, concentration (c) is given in g (100 mL)$^{-1}$.

Proton magnetic resonance spectra were recorded using an internal deuterium lock (at ambient temperature unless stated otherwise) on Bruker Avance III HD (400 MHz, Smart Probe), Bruker Avance III HD (400 MHz, BBO Probe), Bruker Neo Prodigy (400 MHz, Prodigy Cryoprobe), Bruker Avance III (400 MHz, QNP Cryoprobe), Bruker Avance III (500 MHz; DCH Cryoprobe), Bruker Avance (600 MHz, BBI probe), or Bruker Avance II+ (700 MHz, TBO Cryoprobe) spectrometers. Proton assignments are supported by ^1H-^1H COSY, ^1H-^{13}C HSQC or ^1H-^{13}C HMBC spectra, or by analogy. Chemical shifts (δ_H) are quoted in ppm to the nearest 0.01 ppm and are referenced to the residual non-deuterated solvent peak.[1] Discernible coupling constants (J) are reported as measured values in Hz, rounded to the nearest 0.1 Hz. Data are reported as: chemical shift, multiplicity (br, broad; s, singlet; d, doublet; t, triplet; q, quartet; qn, quintet; m, multiplet; ABq, AB quartet; or a combination thereof), coupling constants, and number of nuclei.

Carbon magnetic resonance spectra were recorded using an internal deuterium lock at ambient temperature on Bruker Avance III HD (101 MHz, Smart Probe), Bruker Avance III HD (101 MHz, BBO Probe), Bruker Neo Prodigy (101 MHz, Prodigy Cryoprobe), Bruker Avance III (101 MHz, QNP Cryoprobe), Bruker Avance III (126 MHz; DCH Cryoprobe), or Bruker Avance II+ (176 MHz, TBO Cryoprobe) spectrometers with broadband proton decoupling. Carbon spectra assignments are supported by DEPT editing, ^1H-^{13}C HSQC or ^1H-^{13}C HMBC spectra, or by analogy. Chemical shifts (δ_C) are quoted in ppm to the nearest 0.1 ppm (or to the nearest 0.01 ppm when two or more distinguishable peaks have the same chemical shifts when rounded to the nearest 0.1 ppm) and are referenced to the deuterated solvent peak.[1] Coupling constants between carbon and other nuclei (X) over *n* bonds ($^nJ_{C-X}$) are reported as measured values in Hz, rounded to the nearest 0.1 Hz. Data are reported as: chemical shift, multiplicity (if not a singlet), coupling constants, and number of nuclei (if not one).

Fluorine magnetic resonance spectra were recorded on Bruker Avance III (376 MHz; QNP Cryoprobe) or Bruker Avance III HD (376 MHz; Smart probe) spectrometers with proton decoupling. Chemical shifts (δ_F) are quoted in ppm to the nearest 0.1 ppm and are referenced to $CFCl_3$. Data are reported as: chemical shift, multiplicity (if not a singlet), coupling constants, and number of nuclei (if not one).

Magnetic resonance spectra were processed using TopSpin v. 4.0 (Bruker). Two or more possible assignments were given when signals could not be distinguished by any means. Measured coupling constants are reported for mutually coupled signals; coupling constants are labelled apparent in the absence of an observed mutual coupling, or multiplet when none can be determined.

Infrared (IR) spectra were recorded neat on a Perkin-Elmer Spectrum One spectrometer using an ATR sampling accessory either as solids or liquid films. Selected absorptions ($\tilde{\nu}_{max}$) are reported in wavenumbers (cm^{-1}) with the following abbreviations: w, weak; m, medium; s, strong; br, broad.

Ultraviolet-visible (UV-Vis) spectra were recorded on a NanoDrop™ One spectrophotometer. The absorption maxima (λ_{max}) are reported in nanometres (nm).

High resolution mass spectrometry (HRMS) measurements were recorded with a Micromass Q-TOF, Waters Vion IMS Qtof or a Waters LCT Premier TOF mass spectrometer using Electrospray ionisation (ESI) techniques. Mass values are reported within the ±5 ppm error limit.

SUPPORTING INFORMATION

Liquid chromatography-mass spectrometry (LCMS) chromatographs were recorded using a Waters ACQUITY H-Class UPLC with an ESCi Multi-Mode Ionisation Waters SQ Detector 2 spectrometer using MassLynx 4.1 software; ESI refers to the electrospray ionisation technique; LC system: solvent A: 2 mM NH_4OAc in water/MeCN (95:5); solvent B: MeCN; solvent C: 2% formic acid; column: ACQUITY UPLC® CSH C18 (2.1 mm × 50 mm, 1.7 µm, 130 Å) at 40 °C; gradient: 5 – 95 % B with constant 5 % C over 1 min at flow rate of 0.6 mL min^{-1}; detector: PDA eλ Detector 220 – 800 nm, interval 1.2 nm

High-performance liquid chromatography (HPLC): Analytical chromatographs were obtained on an Agilent 1260 Infinity using a Supercosil ABZ+PLUS column (150 mm × 4.6 mm, 3 µm) eluting with a linear gradient system (solvent A: 0.05% (v/v) TFA in water, solvent B: 0.05% (v/v) TFA in MeCN) over 15 min at a flow rate of 1 mL min^{-1}. Retention times (t_R) reported refer to analytical chromatographs and are quoted to 0.01 minutes. Semi-preparative HPLC was carried out on an Agilent 1260 Infinity using a Supercosil ABZ+PLUS column (250 mm × 21.2 mm, 5 µm) eluting with a linear gradient system (solvent A: 0.1% (v/v) TFA in water, solvent B: 0.05% (v/v) TFA in MeCN) over 20 min at a flow rate of 20 mL min^{-1}. HPLC was monitored by UV absorbance at 220 and 254 nm.

Protein LC–MS was performed on a Xevo G2-S TOF mass spectrometer coupled to an Acquity UPLC system using an Acquity UPLC BEH300 C4 column (1.7 µm, 2.1 × 50 mm). H_2O with 0.1% formic acid (solvent A) and 95% MeCN and 5% water with 0.1% formic acid (solvent B), were used as the mobile phase at a flow rate of 0.2 mL/min. The gradient was programmed as follows: 95% A for 0.93 min, then a gradient to 100% B over 4.28 min, then 100% B for 1.04 minutes, then a gradient to 95% A over 1.04 min. The electrospray source was operated with a capillary voltage of 2.0 kV and a cone voltage of 150 V. Nitrogen was used as the desolvation gas at a total flow of 850 L h^{-1}. Total mass spectra were reconstructed from the ion series using the MaxEnt algorithm preinstalled on MassLynx software (v4.1 from Waters) according to the manufacturer's instructions. Trastuzumab samples were deglycosylated with PNGase F (New England Biolabs) prior to LC-MS analysis.

Synthesis of hemiasterlin and taltobulin

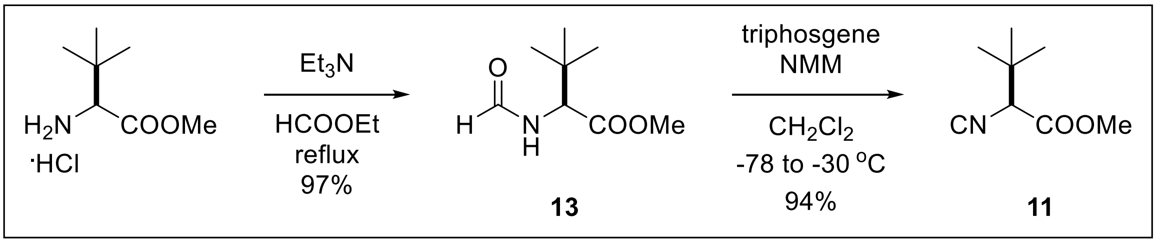

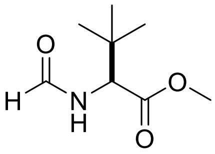

Methyl (*S*)-2-formamido-3,3-dimethylbutanoate (13)

Triethylamine (1.53 mL, 11.0 mmol) was added to a suspension of methyl (*S*)-*tert*-leucinate hydrochloride (1.82 g, 10.0 mmol) in ethyl formate (15 mL). The reaction mixture was refluxed for 20 hours, allowed to cool to room temperature, then filtered through a short silica plug. The solvent was removed *in vacuo* to give formamide **13** as a white solid (1.68 g, 9.70 mmol, 97%). The product was used in the next step without further purification.

$\boldsymbol{R_f}$ = 0.52 (EtOAc)

$[\alpha]_D^{20}$ = +13.4° (c = 1.06, $CHCl_3$, lit.[2] +14.2°, c = 1.0)

^{1}H NMR (400 MHz, $CDCl_3$): Major rotamer δ_H = 8.24 (d, *J* = 0.8 Hz, 1 H), 6.23 (br s, 1 H), 4.57 (dd, *J* = 9.6, 0.7 Hz, 1 H), 3.74 (s, 3 H), 0.99 (s, 9 H); Minor rotamer (observed peaks) δ_H = 7.99 (d, *J* = 11.7 Hz, 1 H), 3.79 (d, *J* = 10.6 Hz, 1 H), 3.76 (s, 3 H)

^{13}C NMR (101 MHz, $CDCl_3$): Major rotamer δ_C = 171.9, 160.8, 58.6, 52.1, 34.9, 26.6; Minor rotamer δ_C = 170.7, 163.6, 63.7, 52.2, 34.8, 26.3

HRMS (ESI): *m/z* $[M+H]^+$ calcd for $C_8H_{16}NO_3$: 174.1130; found: 174.1128 (Δ = -1.1 ppm)

Prepared according to Ackermann *et al*.[2] Spectroscopic data were in accordance with the literature.[2]

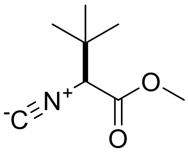

Methyl (*S*)-2-isocyano-3,3-dimethylbutanoate (11)

To a solution of methyl (*S*)-2-formamido-3,3-dimethylbutanoate **13** (866 mg, 5.00 mmol) in anhydrous CH_2Cl_2 (50 mL) was added *N*-methylmorpholine (1.10 mL, 10.0 mmol). The mixture was then cooled to -78 °C. Triphosgene (519 mg, 1.75 mmol) was then added in a single portion and the reaction mixture stirred at -78 °C for 5 minutes before being slowly warmed to -30 °C and then stirred at this temperature for 3 hours. The reaction was then quenched with H_2O (50 mL) and the phases separated. The aqueous phase was then extracted with Et_2O (3 × 50 mL). The combined organic phases were dried with Na_2SO_4, and the solvent was removed *in vacuo* (the bath temperature was kept below 15 °C). The crude product was purified by flash column chromatography (eluting gradient Et_2O/30-40 petroleum ether from 0:1 to 1:4) to give isocyanide **11** as a clear colourless liquid (731 mg, 4.71 mmol, 94%).

$\boldsymbol{R_f}$ = 0.64 (EtOAc/hexane 1:1)

$[\alpha]_D^{20}$ = +33.3° (c = 0.990, $CHCl_3$, lit.[2] +39.7°, c = 1.0)

^{1}H NMR (400 MHz, $CDCl_3$): δ_H = 4.00 (s, 1 H), 3.81 (s, 3 H), 1.10 (s, 9 H)

^{13}C NMR (101 MHz, $CDCl_3$): δ_C = 166.3, 160.0, 66.4 (t, *J* = 7.0 Hz), 52.8, 35.2, 26.2

HRMS (ESI): *m/z* $[M+H]^+$ calcd for $C_8H_{14}NO_2$: 156.1025; found: 156.1022 (Δ = -1.9 ppm)

Procedure adapted from Zhu *et al*.[3] Spectroscopic data were in accordance with the literature.[2]

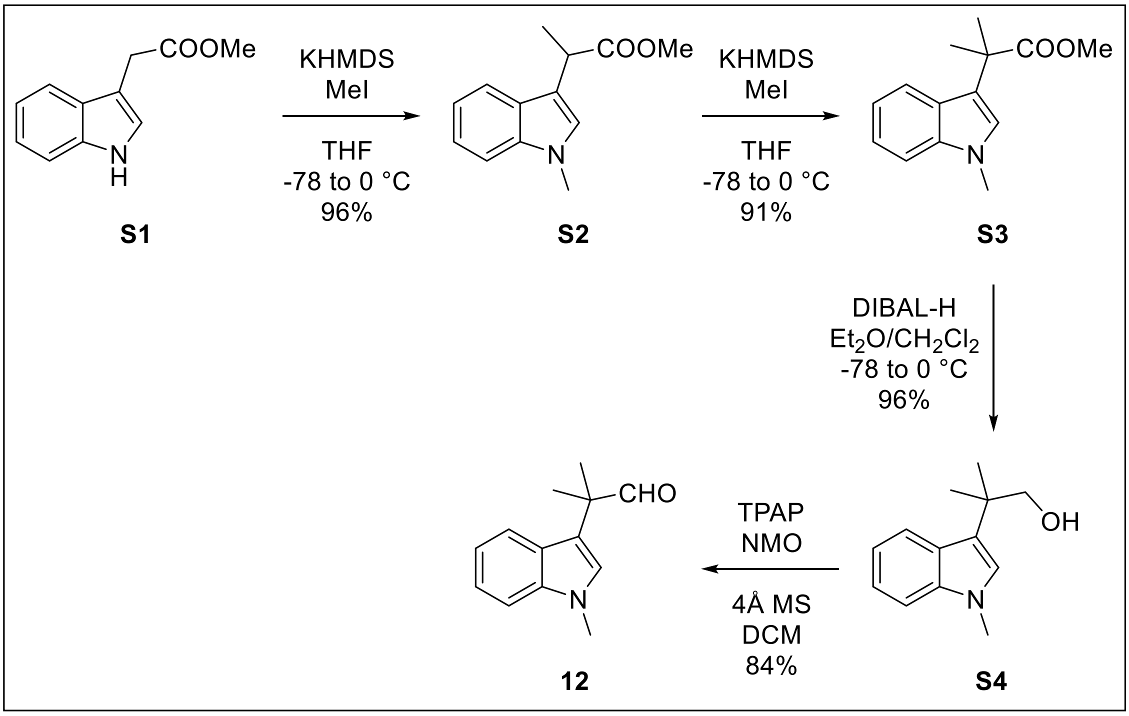

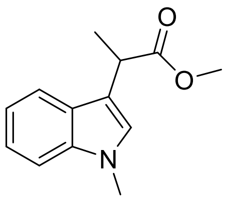

(±)-Methyl 2-(1-methyl-1*H*-indol-3-yl)propanoate (S1)

A solution of KHMDS in THF (0.91 M, 33.0 mL, 30.0 mmol) was diluted with freshly distilled THF (100 mL) and then cooled to -78 °C. A solution of methyl 2-(1*H*-indol-3-yl)acetate **S1** (1.89 g, 10.0 mmol) in THF (46 mL) was then added slowly. The reaction mixture was warmed to 0 °C and stirred for 1 hour before re-cooling to -78 °C. Methyl iodide (5.0 mL, 80 mmol) was then added slowly to the reaction mixture which was then warmed to 0 °C and stirred for 1 hour before being placed in a freezer (-30 °C) for 17 h. The reaction was then quenched with H_2O (135 mL) and then extracted with Et_2O (3 × 135 mL). The combined organic phases were washed with brine (135 mL), dried with anhydrous $MgSO_4$, and the solvent removed *in vacuo*. The resulting crude oil was purified by flash column chromatography (eluting gradient Et_2O/40-60 petroleum ether from 0:1 to 1:4) to give indole **S2** as a viscous yellow oil (2.08 g, 9.57 mmol, 96%).

$\boldsymbol{R_f}$ = 0.17 (Et_2O/40-60 petroleum ether 1:4)

^{1}H NMR (500 MHz, $CDCl_3$): δ_H = 7.68 (dt, *J* = 8.0, 0.9 Hz, 1 H), 7.31 (dt, *J* = 8.2, 0.8 Hz, 1 H), 7.25 (ddd, *J* = 8.2, 7.0, 1.1 Hz, 1 H), 7.14 (ddd, *J* = 8.0, 7.0, 1.1 Hz, 1 H), 7.02 (s, 1 H), 4.05 (q, 1 H, *J* = 7.1 Hz), 3.77 (s, 3 H), 3.69 (s, 3 H), 1.62 (d, *J* = 7.2 Hz, 3 H)

^{13}C NMR (126 MHz, $CDCl_3$): δ_C = 175.8, 137.1, 126.9, 126.4, 121.9, 119.4, 119.2, 114.1, 109.4, 52.1, 36.9, 32.9, 18.2

HRMS (ESI): *m/z* $[M+H]^+$ calcd for $C_{13}H_{16}NO_2$: 218.1176; found: 218.1181 (Δ = -2.3 ppm)

Procedure adapted from Nieman *et al*.[4] Spectroscopic data were in accordance with the literature.[4]

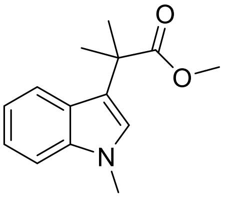

Methyl 2-methyl-2-(1-methyl-1*H*-indol-3-yl)propanoate (S3)

A solution of KHMDS in THF (0.91 M, 13.2 mL, 12.0 mmol) was diluted with freshly distilled THF (94 mL) and then cooled to -78 °C. A solution of (±)-methyl 2-(1-methyl-1*H*-indol-3-yl)propanoate **S2** (1.74 g, 8.00 mmol) in THF (38 mL) was then added slowly. The reaction mixture was warmed to 0 °C and stirred for 1 hour before re-cooling to -78 °C. Methyl iodide (3.0 mL, 48 mmol) was then added slowly to the reaction mixture which was then warmed to 0 °C and stirred for 45 min. The reaction was then quenched with H_2O (80 mL) and then extracted with Et_2O (3 × 80 mL). The combined organic phases were washed with brine (80 mL), dried with anhydrous $MgSO_4$, and the solvent removed *in vacuo*. The resulting crude product was purified by flash column chromatography (eluting gradient Et_2O/40-60 petroleum ether from 0:1 to 1:4) to give indole **S3** as a white solid (1.69 g, 7.31 mmol, 91%).

***R*$_f$** = 0.21 (Et_2O/40-60 petroleum ether 1:4)

^{1}H NMR (500 MHz, $CDCl_3$): δ_H = 7.65 (dt, *J* = 8.0, 0.9 Hz, 1 H), 7.31 (dt, *J* = 8.3, 0.9 Hz, 1 H), 7.23 (ddd, *J* = 8.2, 7.0, 1.1 Hz, 1 H), 7.10 (ddd, *J* = 8.0, 7.0, 1.1 Hz, 1 H), 6.95 (s, 1 H), 3.77 (s, 3 H), 3.65 (s, 3 H), 1.71 (s, 6 H)

^{13}C NMR (126 MHz, $CDCl_3$): δ_C = 177.8, 137.6, 126.2, 125.4, 121.7, 120.4, 119.3, 119.1, 109.5, 52.3, 42.1, 32.8, 26.4

HRMS (ESI): *m/z* $[M+H]^+$ calcd for $C_{14}H_{18}NO_2$: 232.1329; found: 232.1332 (Δ = -1.3 ppm)

Procedure adapted from Nieman *et al*.[4] Spectroscopic data were in accordance with the literature.[4]

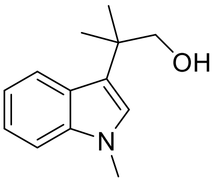

2-Methyl-2-(1-methyl-1H-indol-3-yl)propan-1-ol (S4)

Methyl 2-methyl-2-(1-methyl-1*H*-indol-3-yl)propanoate (**S3**) (1.27 g, 5.50 mmol) was dissolved in Et_2O (60 mL) and CH_2Cl_2 (16 mL) and cooled to -78 °C. A solution of DIBAL-H in THF (1.0 M, 19.3 mL, 19.3 mmol) was then added slowly, and the reaction mixture was warmed to 0 °C and stirred for 3 hours. The reaction was then quenched with H_2O (12 mL), allowed to warm to room temperature, and saturated aqueous solution of potassium sodium tartrate (28 mL) were added. The phases were separated, and the aqueous phase was extracted with Et_2O (2 × 60 mL). The combined organic phases were washed with brine (60 mL), dried with anhydrous $MgSO_4$, and the solvent removed *in vacuo*. The resulting crude product was purified by flash column chromatography (eluting gradient Et_2O/40-60 petroleum ether from 1:20 to 1:1) to give alcohol **S4** as a white solid (1.07 g, 5.26 mmol, 96%).

***R*$_f$** = 0.24 (Et_2O/40-60 petroleum ether 1:1)

^{1}H NMR (400 MHz, $CDCl_3$): δ_H = 7.79 (d, *J* = 8.0 Hz, 1 H), 7.34 (d, *J* = 8.2 Hz, 1 H), 7.28-7.22 (m, 1 H), 7.12 (ddd, *J* = 8.0, 7.0, 1.0 Hz, 1 H), 6.92 (s, 1 H), 3.81 (s, 3 H), 3.77 (s, 3 H), 1.47 (s, 6 H)

^{13}C NMR (101 MHz, $CDCl_3$): δ_C = 138.0, 127.2, 126.1, 121.6, 121.1, 119.5, 118.8, 109.7, 71.6, 37.7, 32.8, 25.6

HRMS (ESI): *m/z* $[M+H]^+$ calcd for $C_{13}H_{18}NO$: 204.1383; found: 204.1377 (Δ = -2.9 ppm)

Procedure adapted from Nieman *et al*.[4] Spectroscopic data were in accordance with the literature.[4]

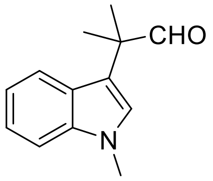

2-Methyl-2-(1-methyl-1*H*-indol-3-yl)propanal (12)

To a mixture of 2-methyl-2-(1-methyl-1*H*-indol-3-yl)propan-1-ol (**S4**) (1.02 g, 5.00 mmol), *N*-methylmorpholine oxide (1.05 g, 9.00 mmol), and 4 Å powdered molecular sieves (1.09 g) in dry CH_2Cl_2 (36 mL) was added tetrapropylammonium perruthenate (87.9 mg, 0.250 mmol). The black reaction mixture was stirred at room temperature for 20 hours, filtered through Celite®, and concentrated *in vacuo*. The crude black oil was purified by flash column chromatography (eluting gradient Et_2O/40-60 petroleum ether from 0:1 to 1:5) to give aldehyde **12** as a pale-yellow solid (846 mg, 4.20 mmol, 84%).

R$_f$ = 0.21 (Et_2O/40-60 petroleum ether 1:4)

mp = 66-68 °C (lit.[4] 61-63 °C)

^{1}H NMR (400 MHz, $CDCl_3$): δ_H = 9.50 (s, 1 H), 7.57 (d, *J* = 8.1 Hz, 1 H), 7.34 (d, *J* = 8.2 Hz, 1 H), 7.26 (t, *J* = 8.0 Hz, 1 H), 7.11 (t, *J* = 7.5 Hz, 1 H), 6.98 (s, 1 H), 3.80 (s, 3 H), 1.56 (s, 6 H)

^{13}C NMR (101 MHz, $CDCl_3$): δ_C = 202.4, 137.8, 126.8, 126.3, 122.0, 120.4, 119.4, 115.2, 109.7, 46.6, 33.0, 22.1

HRMS (ESI): *m/z* $[M+H]^+$ calcd for $C_{13}H_{16}NO$: 202.1232; found: 202.1228 (Δ = -2.0 ppm)

Procedure adapted from Nieman *et al*.[4] Spectroscopic data were in accordance with the literature.[4]

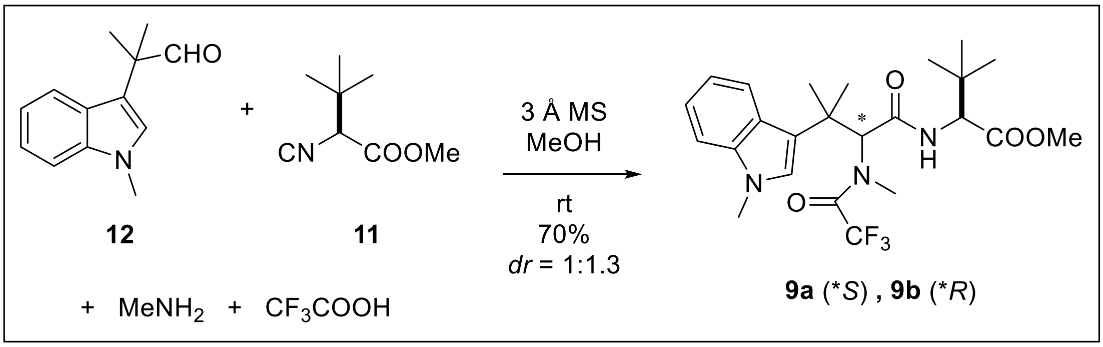

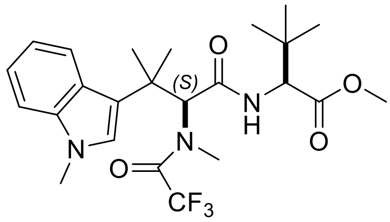

Methyl (*S*)-3,3-dimethyl-2-((*S*)-3-methyl-3-(1-methyl-1*H*-indol-3-yl)-2-(2,2,2-trifluoro-*N*-methylacetamido)butanamido)butanoate (9a)

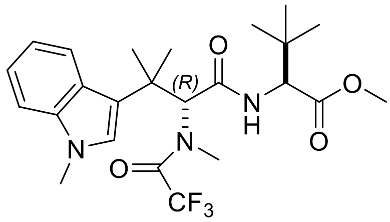

and **methyl (*S*)-3,3-dimethyl-2-((*R*)-3-methyl-3-(1-methyl-1*H*-indol-3-yl)-2-(2,2,2-trifluoro-*N*-methylacetamido)butanamido)butanoate (9b)**

Methylamine solution in MeOH (2 M, 1.10 mL, 2.20 mmol) was added to a solution of 2-methyl-2-(1-methyl-1*H*-indol-3-yl)propanal (**12**) (403 mg, 2.00 mmol) in dry MeOH (5.1 mL). 3 Å molecular sieves (1.8 g) was then added and the reaction was stirred at room temperature for 2 hours before trifluoroacetic acid (184 µL, 2.40 mmol) was added. The reaction mixture was stirred for further 20 minutes before methyl (*S*)-2-isocyano-3,3-dimethylbutanoate (**11**) (373 mg, 2.40 mmol) was added. The reaction was stirred at room temperature for further 19 hours, filtered through Celite®, and the solvent removed *in vacuo*. The crude product was purified by flash column chromatography (eluting gradient EtOAc/40-60 petroleum ether from 0:1 to 1:3) to give amide **9a** as a white solid (291 mg, 0.602 mmol, 30%) and **9b** as a white solid (382 mg, 0.790 mmol, 40%). Small amount of both isomers was re-crystallised from hexane/Et_2O for X-ray crystallographic analysis.

9a

R_f = 0.23 (EtOAc/40-60 petroleum ether 1:3)

mp = 150-157 °C

$[\alpha]_D^{25}$ = -21.7° (c = 1.44, $CHCl_3$)

^{1}H NMR (400 MHz, $CDCl_3$): δ_H = 8.27 (d, *J* = 8.0 Hz, 1 H), 7.33 (d, *J* = 7.6 Hz, 1 H), 7.31-7.26 (m, 1 H), 7.22 (ddd, *J* = 8.0, 6.8, 1.4 Hz, 1 H), 7.09 (s, 1 H), 6.33 (s, 1 H), 5.91 (d, *J* = 8.5 Hz, 1 H), 4.04 (d, *J* = 8.6 Hz, 1 H), 3.77 (s, 3 H), 3.67 (s, 3 H), 3.46 (q, $^5J_{H-F}$ = 1.9 Hz, 3 H), 1.69 (s, 3 H), 1.50 (s, 3 H), 0.45 (s, 9 H)

^{13}C NMR (101 MHz, $CDCl_3$): δ_C = 171.9, 168.4, 159.4 (app d, $^2J_{C-F}$ = 35.6 Hz), 138.2, 127.1, 124.7, 122.6, 121.1, 120.7, 120.2, 117.0 (app d, $^1J_{C-F}$ = 288.3 Hz), 109.8, 63.0, 60.4, 51.7, 39.3, 35.0 (q, $^4J_{C-F}$ = 4.2 Hz), 33.6, 32.9, 27.5, 26.0, 24.5

^{19}F NMR (376 MHz, $CDCl_3$): δ_F = -69.5

IR (ATR): $\tilde{\nu}_{max}$ / cm^{-1} = 3358 (w), 2972 (w), 1738 (m), 1673 (s)

HRMS (ESI): *m/z* $[M+Na]^+$ calcd for $C_{24}H_{32}F_3N_3{}^{23}NaO_4$: 506.2237; found: 506.2238 (Δ = 0.2 ppm)

9b

R_f = 0.35 (EtOAc/40-60 petroleum ether 1:3)

mp = 94-97 °C

$[\alpha]_D^{25}$ = +60.4° (c = 0.747, $CHCl_3$)

^{1}H NMR (400 MHz, $CDCl_3$): δ_H = 8.16 (dd, *J* = 7.0, 1.2 Hz, 1 H), 7.32 (dd, *J* = 7.5, 1.7 Hz, 1 H), 7.28-7.19 (m, 2 H), 6.91 (s, 1 H), 6.12 (s, 1 H), 5.63 (d, *J* = 9.3 Hz, 1 H), 4.07 (d, *J* = 9.0 Hz, 1 H), 3.72 (s, 3 H), 3.51 (s, 3 H), 3.47 (q, $^5J_{H-F}$ = 1.9 Hz, 3 H), 1.74 (s, 3 H), 1.49 (s, 3 H), 0.45 (s, 9 H)

^{13}C NMR (101 MHz, $CDCl_3$): δ_C = 170.7, 167.6, 159.2 (app d, $^2J_{C-F}$ = 36.0 Hz), 138.0, 126.4, 125.1, 122.1, 120.7, 120.1, 119.9, 116.8 (q, $^1J_{C-F}$ = 288.2 Hz), 109.8, 63.0, 60.4, 51.7, 39.3, 35.0 (q, $^4J_{C-F}$ = 4.2 Hz), 33.6, 32.9, 27.5, 26.0, 24.5

^{19}F NMR (376 MHz, $CDCl_3$): δ_F = -69.5

IR (ATR): $\tilde{\nu}_{max}$ / cm^{-1} = 3347 (w), 2967 (w), 1743 (m), 1690 (m), 1673 (s)

HRMS (ESI): *m/z* $[M+H]^+$ calcd for $C_{24}H_{33}F_3N_3O_4$: 484.2418; found: 484.2397 (Δ = -4.3 ppm)

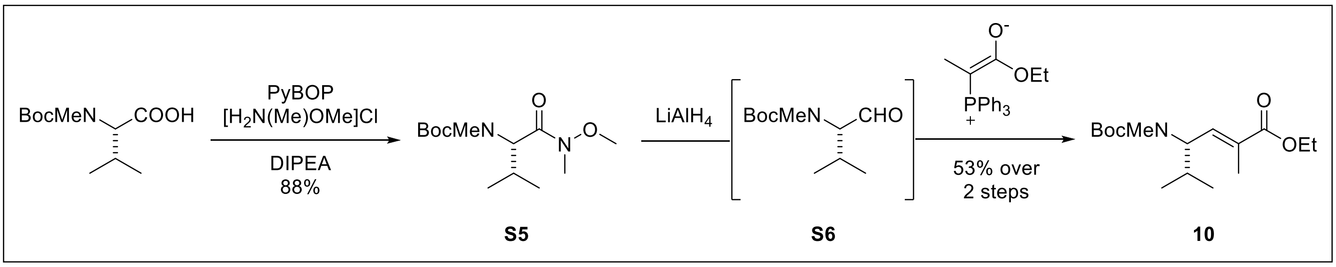

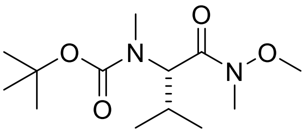

(*S*)-*N*$^{\alpha}$-*tert*-butoxycarbonyl-*N*$^{\alpha}$-methylvalin-*N*-methoxy-*N*-methylamide (S5)

N-Boc-*N*-methylvaline (3.70 g, 16.0 mmol), *N*,*O*-dimethylhydroxylamine hydrochloride (2.03 g, 20.8 mmol), and PyBOP (8.74 g, 16.8 mmol) were dissolved in dry CH_2Cl_2 (16 mL). The mixture was cooled to 0 °C before DIPEA (8.36 mL, 48.0 mmol) was added and stirred at this temperature for 1 minute. The reaction was allowed to warm to room temperature and stirred for a further 2.5 hours. Et_2O (100 mL) was then added and the mixture was washed successively with 3 M aqueous HCl (3 × 35 mL), saturated aqueous $NaHCO_3$ (3 × 35 mL), and brine (3 × 35 mL). The organic phase was then dried with anhydrous $MgSO_4$, and the solvent was removed *in vacuo*. The crude product was then purified by flash column chromatography (eluting gradient Et_2O/40-60 petroleum ether from 0:1 to 1:1) to give Weinreb amide **S5** as a slightly yellow oil (3.86 g, 14.1 mmol, 88%)

R$_f$ = 0.50 (EtOAc/40-60 petroleum ether 1:1)

$[\alpha]_D^{25}$ = -128.7° (c = 1.07, $CHCl_3$, lit.[5] $[\alpha]_D^{20}$ = -163.6°, c = 1.3, $CHCl_3$)

^{1}H NMR (400 MHz, $CDCl_3$): Major rotamer δ_H = 5.00 (br d, *J* = 9.6 Hz, 1 H), 3.72 (s, 3 H), 3.20* (br s, 3 H), 2.83 (s, 3 H), 2.35-2.17* (m, 1 H), 1.46 (s, 9 H); Minor rotamer δ_H = 4.70 (br s, 1 H), 3.68 (s, 3 H), 3.20* (br s, 3 H), 2.80 (s, 3 H), 2.35-2.17* (m, 1 H), 1.49 (s, 9 H); Unassigned† δ_H = 0.90 (d, *J* = 6.8 Hz, 2 H), 0.88 (d, *J* = 6.8 Hz, 4 H)

^{13}C NMR (101 MHz, $CDCl_3$): Major rotamer δ_C = 172.3, 156.3, 80.0, 61.9, 58.1, 32.1, 29.8, 28.5, 27.3; Minor rotamer δ_C = 171.2, 155.7, 79.7, 61.8, 59.9, 32.0, 29.2, 28.6, 27.1; Unassigned† δ_C = 19.9, 19.4, 18.5

*These signals appear as single signals and belong to both rotamers.
†Unassigned signals cannot be unambiguously assigned to either rotamers. For ^{1}H NMR, the numbers of nuclei quoted are with respect to the sum of both rotamers.

HRMS (ESI): *m/z* $[M+Na]^+$ calcd for $C_{13}H_{26}N_2{}^{23}NaO_4$: 297.1785; found: 297.1784 (Δ = -0.3 ppm)

Procedure adapted from Nieman *et al*.[4] Spectroscopic data were in accordance with the literature.[4]

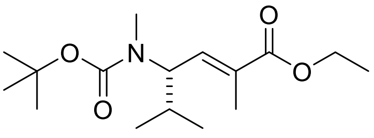

Ethyl (2*E*,4*S*)-*N*-*tert*-butoxycarbonyl-*N*-methyl-4-amino-2,5-dimethylhex-2-enoate (10)

Lithium aluminium hydride (190 mg, 5.00 mmol) was added to the solution of Weinreb amide **S5** (1.37 g, 5.00 mmol) in dry THF (5 mL) in one portion at -78 °C. The reaction mixture was then warmed to 0 °C and stirred for 15 minutes. The mixture was then poured into a stirring ice-cold aqueous solution of $KHSO_4$ (0.25 M, 40 mL) and the phases were separated. The aqueous phase was then extracted with Et_2O (3 × 30 mL). The combined organic phases were quickly washed 1 M aqueous HCl (2 × 30 mL), saturated aqueous $NaHCO_3$ (2 × 30 mL), and brine (30 mL), dried with anhydrous $MgSO_4$, and concentrated *in vacuo* (the bath temperature was kept lower than 20 °C) to give the crude aldehyde **S6** as a clear volatile liquid which was used in the next step without further purification.

The crude aldehyde obtained was dissolved in dry CH_2Cl_2 (5 mL) and [(1-ethoxycarbonyl)ethylidene]triphenylphosphorane (2.72 g, 7.50 mmol) was added. The reaction mixture was stirred at room temperature for 20 hours. The reaction was quenched with addition of H_2O (15 mL) and the phases were separated. The aqueous phase was extracted with Et_2O (3 × 30 mL), and the combined organic phases were washed with brine (30 mL), dried with anhydrous $MgSO_4$, and the solvent was removed *in vacuo*. The crude product was purified

by flash column chromatography (eluting gradient Et_2O/40-60 petroleum ether from 0:1 to 1:4) to give unsaturated ester **10** as a colourless oil (798 mg, 2.67 mmol, 53% over two steps).

R$_f$ = 0.21 (EtOAc/40-60 petroleum ether 1:4)

$[\alpha]_D^{25}$ = -83.5° (c = 1.00, $CHCl_3$, lit.[4] +61.1°, c = 9.1, $CHCl_3$)

^{1}H NMR (400 MHz, $CDCl_3$): Rotameric mixtures* δ_H = 6.66 (br d, *J* = 9.2 Hz, 1 H), 4.64-4.50 (m, 0.5 H), 4.26-4.38 (m, 0.5 H), 4.21 (q, *J* = 7.1 Hz, 2 H), 2.70 (br s, 3 H), 1.94-1.81 (m, 1 H), 1.91 (s, 3 H), 1.47 (s, 9 H), 1.31 (t, *J* = 7.1 Hz), 0.92 (d, *J* = 6.6 Hz), 0.86 (d, *J* = 6.3 Hz)

^{13}C NMR (126 MHz, $CDCl_3$): Rotameric mixtures* δ_C = 168.1 (br), 155.9, 155.7, 139.0, 138.9, 131.9, 131.1, 79.9, 79.5, 61.2, 60.9, 59.8, 59.1, 30.5, 29.3, 29.1, 28.6, 19.7, 19.3, 19.0, 14.4, 14.1, 13.3 (br)

*Signals are unassigned to individual rotamers as most protons give the same signals to both rotamers.

HRMS (ESI): *m/z* $[M+Na]^+$ calcd for $C_{16}H_{29}N^{23}NaO_4$: 322.1989; found: 322.1984 (Δ = -1.6 ppm)

Procedure adapted from Nieman *et al*.[4] Spectroscopic data were in accordance with the literature.[4]

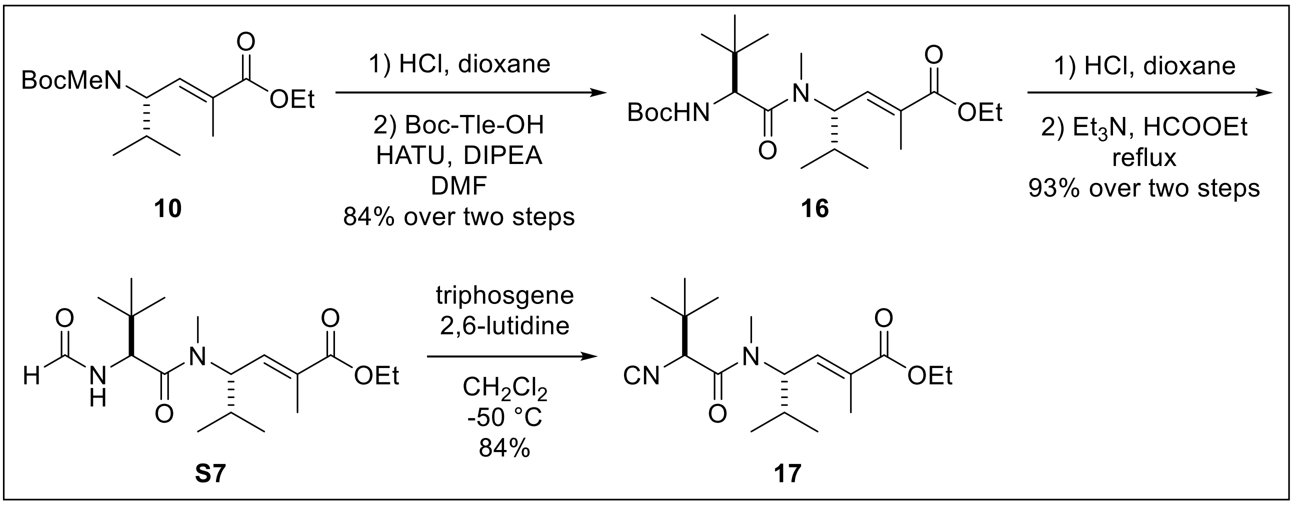

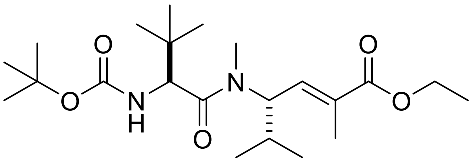

Ethyl (*S*,*E*)-4-((*S*)-2-((*tert*-butoxycarbonyl)amino)-*N*,3,3-trimethylbutanamido)-2,5-dimethylhex-2-enoate (16)

To a solution of Boc-protected amine **10** (2.29 g, 7.65 mmol) in dioxane (19 mL) was added a solution of HCl in dioxane (4 M, 19.1 mL, 76.5 mmol). The reaction mixture was then stirred at room temperature for 15 hours before Et_2O (100 mL) was added. The white precipitate formed was filtered under reduced pressure and washed with copious amount of Et_2O to give HCl salt of ethyl (2*E*,4*S*)-*N*-methyl-4-amino-2,5-dimethylhex-2-enoate as a white powder (1.61 g, 6.83 mmol, 90%). The product was used in the next step without further purification.

To a solution of (*S*)-*N*-*tert*-butoxycarbonyl-*tert*-leucine (3.15 g, 13.6 mmol) and HATU (5.17 g, 13.6 mmol) in DMF (30 mL) at 0 °C was added DIPEA (2.37 mL, 13.6 mmol). The reaction mixture was stirred for 5 minutes before a solution of HCl salt of ethyl (2*E*,4*S*)-*N*-methyl-4-amino-2,5-dimethylhex-2-enoate (1.59 g, 6.7 mmol) and DIPEA (2.37 mL, 13.6 mmol) in DMF (15 mL) was added over 2 minutes. The reaction mixture was stirred for 18 hours before ethyl acetate (500 mL) was added. The mixture was then successively washed with 1 M aqueous HCl (3 × 100 mL), saturated aqueous $NaHCO_3$ (3 × 100 mL), and brine (100 mL). The organic phase was then dried with anhydrous $MgSO_4$, and the solvent was removed *in vacuo*. The crude product was purified by flash column chromatography (eluting gradient EtOAc/40-60 petroleum ether from 0:1 to 1:5) to give amide **16** as a white solid (2.59 g, 6.28 mmol, 93%)

R$_f$ = 0.48 (EtOAc/40-60 petroleum ether 1:3)

[α]D25 = -126.9° (c = 0.864, $CHCl_3$, lit.[4] -76.9°, c = 2.43, $CHCl_3$)

^{1}H NMR (400 MHz, $CDCl_3$): δ_H = 6.63 (app dd, *J* = 9.3, 1.4 Hz, 1 H), 5.20 (d, *J* = 10.0 Hz, 1 H), 5.09 (t, *J* = 9.9 Hz, 1 H), 4.41 (d, *J* = 10.2 Hz, 1 H), 4.18 (q, *J* = 7.1 Hz, 2 H), 2.97 (s, 3 H), 1.93-1.82 (m, 4 H), 1.40 (s, 9 H), 1.29 (t, *J* = 7.1 Hz, 3 H), 0.94 (s, 9 H), 0.86 (d, *J* = 6.6 Hz, 3 H), 0.82 (d, *J* = 6.6 Hz, 3 H)

^{13}C NMR (101 MHz, $CDCl_3$): δ_C = 172.5, 167.9, 156.2, 138.7, 132.7, 79.6, 60.9, 56.4, 56.0, 35.0, 31.1, 30.2, 28.4, 26.5, 19.6, 18.7, 14.3, 13.9

LCMS (ESI): *m/z* $[M+H]^+$ calcd for $C_{22}H_{41}N_2O_5$: 413.3; found: 413.4

Spectroscopic data were in accordance with the literature.[4]

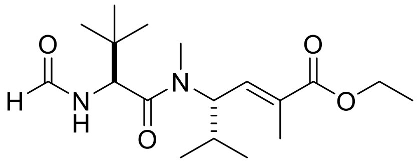

Ethyl (*S*,*E*)-4-((*S*)-2-formamido-*N*,3,3-trimethylbutanamido)-2,5-dimethylhex-2-enoate (S7)

To a solution of Boc-protected amine **16** (744 mg, 1.80 mmol) in dioxane (4.5 mL) was added a solution of HCl in dioxane (4 M, 4.50 mL, 18.0 mmol). The reaction mixture was then stirred at room temperature for 14 hours before volatiles were removed *in vacuo* to give the crude deprotected amine. Ethyl formate (60 mL) and triethylamine (276 µL) were added and the reaction mixture was refluxed for 20 hours. The mixture was then filtered through short silica gel plug and eluted with EtOAc. The volatiles were removed *in vacuo* to give formamide **S7** as a colourless viscous oil which solidified into white needles under stream of N_2 over several days (569 mg, 1.67 mmol, 93% over two steps). This compound was used in the next step without further purification.

R_f = 0.50 (EtOAc)

mp = 71-75 °C

[α]D25 = -129.3° (c = 0.760, $CHCl_3$)

^{1}H NMR (400 MHz, $CDCl_3$): δ_H = 8.21 (d, *J* = 1.0 Hz), 6.64 (app dd, *J* = 9.4, 1.3 Hz, 1 H), 6.46 (br d, *J* = 9.3 Hz, 1 H), 5.09 (t, *J* = 10.0 Hz, 1 H), 4.94 (d, *J* = 9.7 Hz, 1 H), 4.20 (q, *J* = 7.1 Hz, 2 H), 2.99 (s, 3 H), 1.97-1.83 (m, 4 H),1.31 (t, *J* = 7.1 Hz, 3 H), 0.97 (s, 9 H), 0.88 (d, *J* = 6.5 Hz, 3 H), 0.82 (d, *J* = 6.5 Hz, 3 H)

^{13}C NMR (101 MHz, $CDCl_3$): δ_C = 171.3, 167.8, 160.8, 138.3, 132.9, 61.0, 56.3, 53.3, 35.7, 31.3, 30.1, 26.6, 19.5, 18.9, 14.3, 14.0

IR (ATR): $\tilde{\nu}_{max}$ / cm^{-1} = 3264 (w), 2965 (m), 1713 (m), 1674 (m), 1633 (s)

HRMS (ESI): *m/z* $[M+Na]^+$ calcd for $C_{18}H_{32}N_2{}^{23}NaO_4$: 363.2254; found: 363.2261 (Δ = 1.9 ppm)

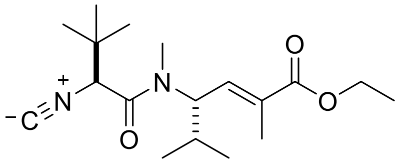

Ethyl (*S*,*E*)-4-((*S*)-2-isocyano-*N*,3,3-trimethylbutanamido)-2,5-dimethylhex-2-enoate (17)

To a solution of formamide **S7** (529 mg, 1.55 mmol) in CH_2Cl_2 (31 mL) at -50 °C was added 2,6-lutidine (812 µL, 6.98 mmol) and triphosgene (276 mg, 0.93 mmol) in one portion. The reaction mixture was stirred at -50 °C for 21 h before quenching by addition of saturated aqueous $NaHCO_3$ (45 mL) and CH_2Cl_2 (45 mL). The phases were then separated. The organic phase was then washed successively with 1 M aqueous HCl (30 mL), saturated aqueous $NaHCO_3$ (30 mL), and brine (30 mL), dried with anhydrous Na_2SO_4, filtered, and the solvent removed *in vacuo*. The crude oil was purified by flash column chromatography (eluting gradient EtOAc/40-60 petroleum ether from 0:1 to 1:5) to give isocyanide **17** as a white solid (423 mg, 1.31 mmol, 84%).

R_f = 0.38 (EtOAc/40-60 petroleum ether 1:3)

mp = 102-104 °C

[α]$_D$^{25} = -86.9° (c = 0.452, $CHCl_3$)

^{1}H NMR (400 MHz, $CDCl_3$): δ_H = 6.63 (app dd, *J* = 9.2, 1.4 Hz, 1 H), 5.10 (t, *J* = 9.9 Hz, 1 H), 4.21 (q, *J* = 7.2 Hz, 2 H), 4.19 (s, 1 H), 2.94 (s, 3 H), 2.01-1.90 (m, 1 H), 1.89 (d, *J* = 1.4 Hz, 3 H), 1.31 (t, *J* = 7.1 Hz, 3 H), 1.12 (s, 9 H), 0.94 (d, *J* = 6.7 Hz, 3 H), 0.91 (d, *J* = 6.5 Hz, 3 H)

^{13}C NMR (101 MHz, $CDCl_3$): δ_C = 167.7, 164.9, 158.4 (br), 137.7, 133.4, 61.7, 61.1, 56.7, 35.7, 31.1, 30.5, 26.2, 19.6, 18.8, 14.4, 13.8

IR (ATR): $\tilde{\nu}_{max}$ / cm^{-1} = 2964 (m), 2153 (m), 1715 (s), 1646 (s)

HRMS (ESI): *m/z* $[M+Na]^+$ calcd for $C_{18}H_{30}N_2{}^{23}NaO_3$: 345.2149; found: 345.2153 (Δ = 1.2 ppm)

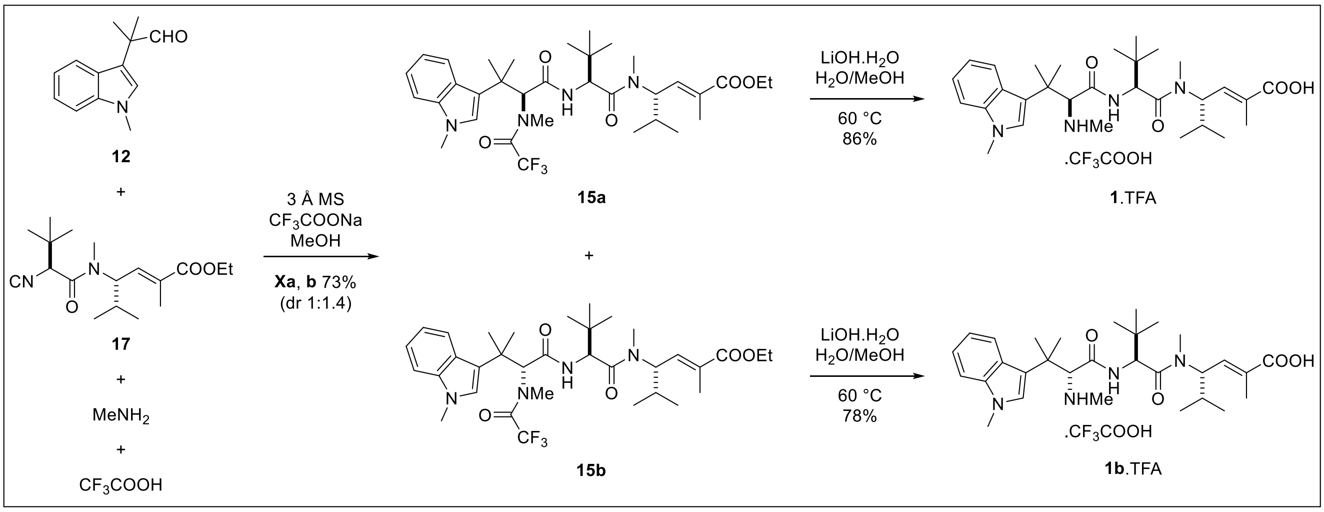

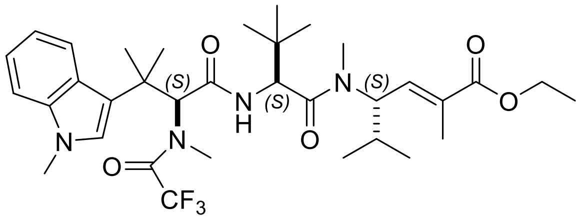

***N*-trifluoroacetyl hemiasterlin ethyl ester (15a)**

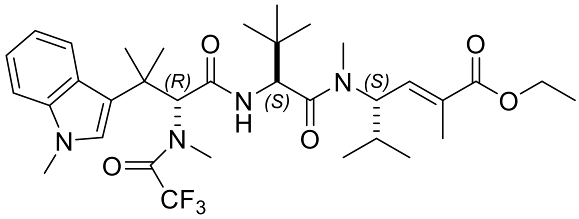

and *N*-trifluoroacetyl *epi*-hemiasterlin ethyl ester (15b)

Methylamine solution in MeOH (2 M, 55 µL, 0.11 mmol) was added to a solution of 2-methyl-2-(1-methyl-1*H*-indol-3-yl)propanal (**12**) (20.1 mg, 0.100 mmol) in dry MeOH (0.2 mL). 3 Å molecular sieves (100 mg) was then added and the reaction was stirred at room temperature for 2 hours before trifluoroacetic acid (9.78 µL, 0.120 mmol) and sodium trifluoroacetate (16.3 mg, 0.120 mmol) were added. The reaction mixture was stirred for further 30 minutes before isocyanide **17** (38.7 mg, 0.120 mmol) was added. The reaction was stirred at room temperature for further 19 hours, filtered through Celite®, and the solvent removed *in vacuo*. The crude product was purified by flash column chromatography (eluting gradient EtOAc/40-60 petroleum ether from 0:1 to 1:3) to give protected hemiasterlin **15a** as a white solid (26.5 mg, 41%) and protected *epi*-hemiasterlin **15b** as a white solid (20.8 mg, 32%). A small amount of **15a** was re-crystallised using slow evaporation method with ethyl acetate/hexane (1:5) as a solvent for X-ray crystallographic analysis.

15a (*S*,*S*,*S*)

R_f = 0.24 (EtOAc/40-60 petroleum ether 1:3)

mp = 181-184 °C

[α]$_D^{25}$ = -103.8° (c = 0.212, $CHCl_3$)

^{1}H NMR (400 MHz, $CDCl_3$): δ_H = 8.28 (d, *J* = 8.1 Hz, 1 H), 7.33 (d, *J* = 8.3 Hz, 1 H), 7.28 (ddd, *J* = 8.1, 6.9, 1.0, 1 H), 7.21 (ddd, *J* = 8.0, 6.9, 1.3 Hz, 1 H), 7.18 (s, 1 H), 6.64 (dq, *J* = 9.2, 1.5 Hz, 1 H), 6.38 (s, 1 H), 6.32 (br d, *J* = 8.7 Hz, 1 H), 5.07 (dd, *J* = 10.5, 9.5 Hz, 1 H), 4.42 (d, *J* = 8.8 Hz, 1 H), 4.20 (q, *J* = 7.1 Hz, 2 H), 3.78 (s, 3 H), 3.37 (q, $^5J_{H\text{-}F}$ = 1.9 Hz, 3 H), 2.97 (s, 3 H), 1.97-1.87 (m, 4 H), 1.61 (s, 3 H), 1.47 (s, 3 H), 1.30 (t, *J* = 7.1 Hz, 3 H), 0.91 (d, *J* = 6.6 Hz, 3 H), 0.87 (d, *J* = 6.6 Hz, 3 H), 0.44 (s, 9 H)

^{13}C NMR (101 MHz, $CDCl_3$): δ_C = 171.3, 168.6, 167.8, 159.3 (q, $^2J_{C\text{-}F}$ = 35.5 Hz), 138.9, 138.3, 132.7, 127.0, 124.7, 122.6, 121.2, 120.9, 120.1, 117.0 (app d, $^1J_{C\text{-}F}$ = 288.1 Hz), 109.7, 63.2, 61.0, 56.2, 55.5, 39.2, 35.1 (q, $^4J_{C\text{-}F}$ = 4.1 Hz), 34.0, 32.8, 31.3, 30.2, 27.7, 26.0, 24.3, 19.6, 19.0, 14.3, 14.0

^{19}F NMR (376 MHz, $CDCl_3$): δ_F = -69.5

IR (ATR): $\tilde{\nu}_{max}$ / cm^{-1} = 3359 (m), 2967 (m), 2925 (m), 1706 (m), 1686 (m), 1663 (m), 1634 (m)

HRMS (ESI): *m/z* [M+Na]$^+$ calcd for $C_{34}H_{49}F_3N_4{}^{23}NaO_5$: 673.3547; found: 673.3564 (Δ = 2.5 ppm)

15b (*R*,*S*,*S*)

R_f = 0.18 (EtOAc/40-60 petroleum ether 1:3)

mp = 150-153 °C

[α]$_D^{25}$ = -16.2° (c = 0.222, $CHCl_3$)

^{1}H NMR (400 MHz, $CDCl_3$): δ_H = 8.07 (d, *J* = 7.4 Hz, 1 H), 7.29 (dd, *J* = 7.5, 1.3 Hz, 1 H), 7.24-7.15 (m, 2 H), 6.90 (s, 1 H), 6.54 (dq, *J* = 9.0, 1.5 Hz, 1 H), 5.98 (s, 1 H), 5.95 (d, *J* = 9.5 Hz, 1 H), 4.91 (dd, *J* = 10.5, 9.2 Hz, 1 H), 4.49 (d, *J* = 9.6 Hz, 1 H), 4.17 (q, *J* = 7.1 Hz, 2 H), 3.71 (s, 3 H), 3.39 (q, $^5J_{H\text{-}F}$ = 1.9 Hz, 3 H), 2.82 (s, 3 H), 1.79 (d, *J* = 1.4 Hz, 3 H), 1.78-1.70 (m, 4 H), 1.49 (s, 3 H), 1.28 (t, *J* = 7.1 Hz, 3 H), 0.81 (d, *J* = 6.6 Hz, 3 H), 0.55 (d, *J* = 6.6 Hz, 3 H), 0.40 (s, 9 H)

^{13}C NMR (101 MHz, $CDCl_3$): δ_C = 170.9, 167.8, 167.4, 159.0 (q, $^2J_{C\text{-}F}$ = 35.6 Hz), 138.6, 138.1, 133.0, 126.6, 125.5, 122.1, 120.8, 120.0, 119.9, 116.8 (q, $^1J_{C\text{-}F}$ = 288.3 Hz), 109.9, 62.9, 60.9, 55.6, 54.3, 39.8, 34.5, 33.7 (q, $^4J_{C\text{-}F}$ = 4.1 Hz), 32.8, 30.9, 30.3, 27.3, 25.7, 24.5, 19.4, 18.0, 14.3, 13.8

^{19}F NMR (376 MHz, $CDCl_3$): δ_F = -69.7

IR (ATR): $\tilde{\nu}_{max}$ / cm^{-1} = 3316 (m), 2960 (m), 1687 (s), 1672 (s), 1634 (s)

HRMS (ESI): *m/z* [M+Na]$^+$ calcd for $C_{34}H_{49}F_3N_4{}^{23}NaO_5$: 673.3547; found: 673.3562 (Δ = 2.2 ppm)

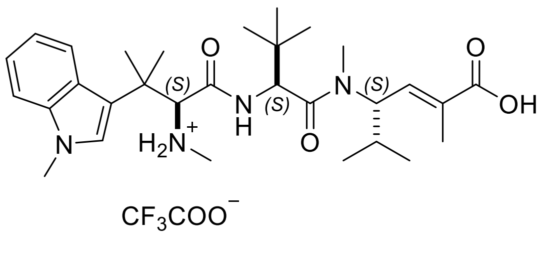

Hemiasterlin trifluoroacetate salt (1•TFA)

LiOH•H_2O (16.0 mg, 0.381 mmol) was added to a solution of *N*-trifluoroacetyl hemiasterlin ethyl ester **15a** (15.5 mg, 23.8 µmol) in H_2O/MeOH (1:1, 2 mL). The reaction was heated to 60 °C and stirred for 8 days. The mixture was then purified by reverse-phase semi-preparative HPLC (30-46%B over 16 min) to give trifluoroacetate salt of hemiasterlin **1**•TFA as a white powder (13.1 mg, 20.4 µmol, 86%).

t_R / min = 10.14 (5-95%B over 15 min)

[α]$_D^{25}$ = -24.1° (c = 0.108, MeOH, lit.[4] -76°, c = 0.7)

^{1}H NMR (700 MHz, DMSO-d$_6$): δ_H = 12.48 (br s, 1 H), 8.96-8.78 (m, 2 H), 8.11 (br s, 1 H), 7.46 (d, *J* = 8.1 Hz, 1 H), 7.35 (br s, 1 H), 7.21 (t, *J* = 7.5 Hz, 1 H), 7.17 (s, 1 H), 7.09 (t, *J* = 7.3 Hz, 1 H), 6.68 (d, *J* = 9.4 Hz, 1 H), 4.94 (t, *J* = 10.0 Hz, 1 H), 4.86 (d, *J* = 8.2 Hz, 1 H), 4.43 (br s, 1 H), 3.76 (s, 3 H), 3.03 (s, 3 H), 2.23 (br s, 3 H), 2.07-1.96 (m, 1 H), 1.80 (s, 3 H), 1.41 (s, 3 H), 1.38 (s, 3 H), 1.00 (s, 9 H), 0.81 (d, *J* = 6.6 Hz, 3 H), 0.79 (d, *J* = 6.2 Hz, 3 H)

^{13}C NMR (176 MHz, DMSO-d$_6$): δ_C = 170.1, 168.6, 165.7, 157.7 (q, $^2J_{C\text{-}F}$ = 30.9 Hz), 138.4, 137.8, 131.8, 128.8, 125.0, 121.3, 120.4, 118.5, 117.4 (q, $^1J_{C\text{-}F}$ = 300.7 Hz), 116.3, 110.2, 67.4, 56.3, 55.6, 37.6, 34.8, 33.5, 32.5, 31.1, 28.8, 27.0, 26.4, 26.4, 19.4, 18.9, 13.6

^{19}F NMR (376 MHz, DMSO-d$_6$): δ_F = -73.4

IR (ATR): $\tilde{\nu}_{max}$ / cm^{-1} = 2967 (m), 1673 (s)

UV-Vis: λ_{max} / nm = 229, 287 (1 mM in 1:4 MeCN:H_2O, lit.[4] 216, 273 in MeOH)

HRMS (ESI): *m/z* [M+H]$^+$ calcd for $C_{30}H_{47}N_4O_2$: 527.3592; found: 527.3606 (Δ = 2.7 ppm)

We found that while the NMR data corresponded well to the literature,[4] the optical rotatory power in MeOH deviates significantly. Spectroscopic data were in accordance with the literature.[4]

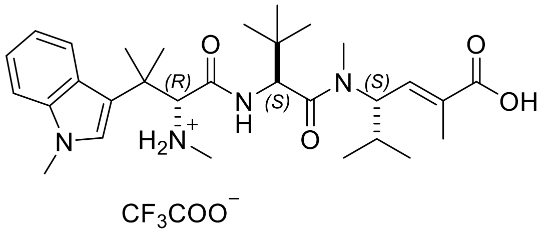

***epi*-hemiasterlin trifluoroacetate salt (1b•TFA)**

LiOH•H_2O (256.2 mg, 0.624 mmol) was added to a solution of *N*-trifluoroacetyl *epi*-hemiasterlin ethyl ester **15b** (25.4 mg, 39.0 µmol) in H_2O/MeOH (1:1, 3 mL). The reaction was heated to 60 °C and stirred for 7 days. The mixture was then purified by reverse-phase semi-preparative HPLC (30-46%B over 16 min) to give trifluoroacetate salt of *epi*-hemiasterlin **1b**•TFA as a white powder (19.5 mg, 30.9 µmol, 78%).

t_R / min = 10.06 (5-95%B over 15 min)

$[\alpha]_D^{25}$ = -101.8° (c = 0.108, MeOH)

^{1}H NMR (700 MHz, DMSO-d$_6$): δ_H = 12.51 (br s, 1 H), 9.00 (br s, 1 H), 8.89 (br s, 1 H), 8.09 (d, *J* = 7.8 Hz, 1 H), 7.54 (br s, 1 H), 7.44 (d, *J* = 8.2 Hz, 1 H), 7.20 (t, *J* = 7.8 Hz, 1 H), 7.18 (s, 1 H), 7.08 (t, *J* = 7.5 Hz, 1 H), 6.66 (d, *J* = 9.5 Hz, 1 H), 4.90 (t, *J* = 10.0 Hz, 1 H), 4.76 (d, *J* = 9.0 Hz, 1 H), 4.53 (d, *J* = 9.7 Hz, 1 H), 3.75 (s, 3 H), 3.02 (s, 3 H), 2.17 (s, 3 H), 2.05-1.95 (m, 1 H), 1.78 (s, 3 H), 1.51 (s, 3 H), 1.47 (s, 3 H), 0.89 (s, 9 H), 0.79 (d, *J* = 6.6 Hz, 3 H), 0.72 (d, *J* = 6.5 Hz, 3 H)

^{13}C NMR (176 MHz, DMSO-d$_6$): δ_C = 170.7, 168.6, 166.5, 157.7 (q, $^2J_{C\text{-}F}$ = 30.4 Hz), 138.4, 137.8, 131.9, 128.6, 125.0, 121.3, 120.6, 118.5, 117.5 (app d, $^1J_{C\text{-}F}$ = 303.6 Hz), 116.3, 110.1, 67.6, 56.2, 55.6, 37.1, 34.2, 33.1, 32.5, 31.1, 28.8, 26.6, 26.3, 23.1, 19.4, 18.8, 13.6

^{19}F NMR (376 MHz, DMSO-d$_6$): δ_F = -73.4

IR (ATR): $\tilde{\nu}_{max}$ / cm^{-1} = 2970 (m), 1671 (s)

UV-Vis: λ_{max} / nm = 229, 288 (1 mM in 1:4 MeCN:H_2O)

HRMS (ESI): *m/z* [M+H]$^+$ calcd for $C_{30}H_{47}N_4O_2$: 527.3592; found: 527.3620 (Δ = 5.3 ppm)

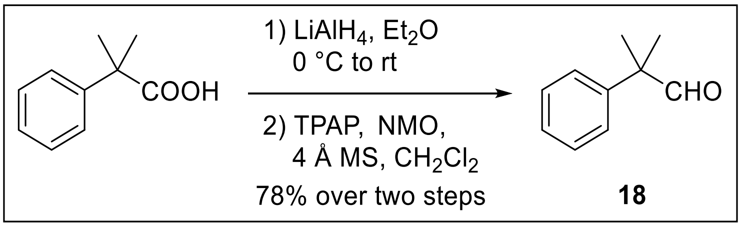

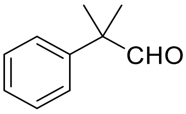

2-Methyl-2-phenylpropanal (18)

2-Methyl-2-phenylpropanoic acid (657 mg, 4.00 mmol) was dissolved in dry Et_2O (80 mL) and cooled to 0 °C. $LiAlH_4$ (493 mg, 13.0 mmol) was added to the mixture in 3 portions over 10 minutes. The reaction was allowed to warm to room temperature and stirred for 90 minutes before being cooled to 0 °C. The reaction was quenched by the slow addition of 1 M aqueous HCl (10 mL) and the phases separated. The aqueous phase was extracted with Et_2O (3 × 25 mL) and the combined organic phases were dried with anhydrous $MgSO_4$. The solvent was removed *in vacuo* to give the crude alcohol which was then re-dissolved in dry CH_2Cl_2 (29 mL). *N*-methylmorpholine oxide (844 mg, 7.20 mmol), 4 Å powdered molecular sieves (870 mg), and tetrapropylammonium perruthenate (70.3 mg, 0.200 mmol) were added to the solution. The black reaction mixture was stirred at room temperature for 20 hours, filtered through Celite®, and concentrated *in vacuo* (the bath temperature was kept below 20 °C). The crude black oil was purified by flash column chromatography (eluting gradient Et_2O/30-40 petroleum ether from 0:1 to 1:50) to give aldehyde **18** as a colourless liquid (460 mg, 3.11 mmol, 78% over two steps).

***R**$_f$* = 0.53 (Et_2O/40-60 petroleum ether 1:5)

^{1}H NMR (400 MHz, $CDCl_3$): δ_H = 9.52 (s, 1 H), 7.40 (t, *J* = 7.5 Hz, 2 H), 7.33-7.27 (m, 3 H), 1.48 (s, 6 H)

^{13}C NMR (101 MHz, $CDCl_3$): δ_C = 202.4, 141.3, 129.0, 127.4, 126.8, 50.6, 22.6

Spectroscopic data were in accordance with the literature[6].

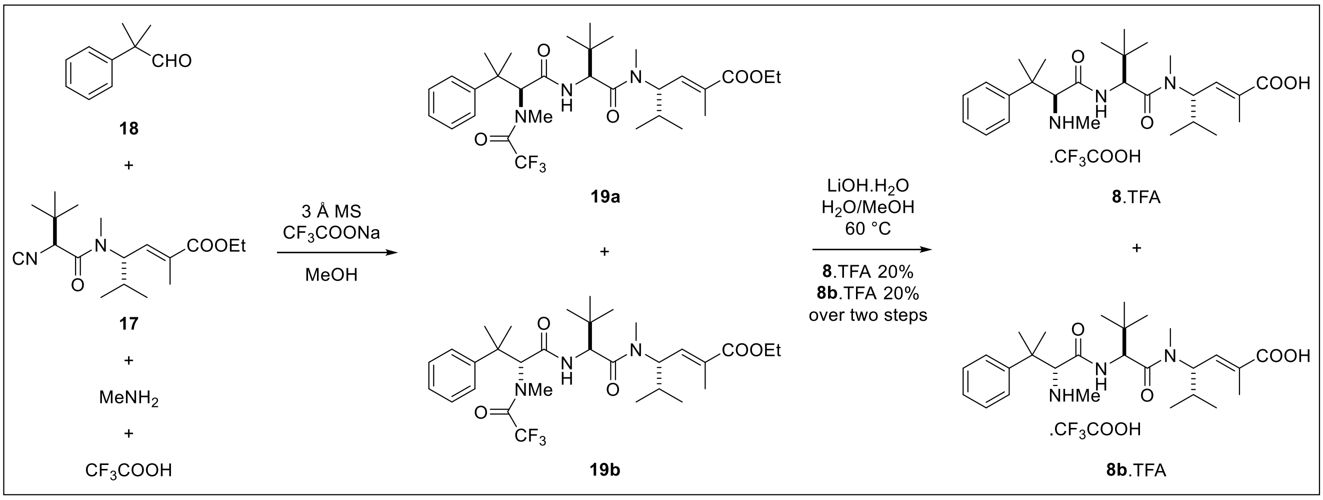

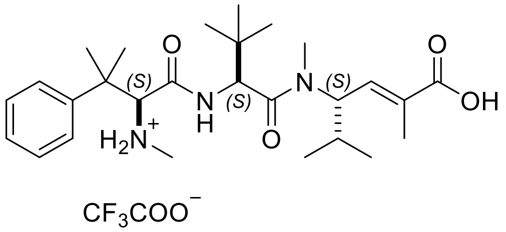

Taltobulin trifluoroacetate salt (8•TFA)

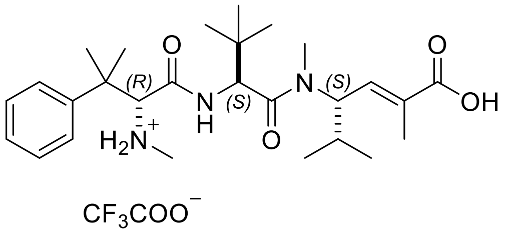

and *epi*-Taltobulin trifluoroacetate salt (8b•TFA)

Methylamine solution in MeOH (2 M, 110 µL, 0.220 mmol) was added to a mixture of 2-methyl-2-phenylpropanal **18** (29.6 mg, 0.200 mmol), 3 Å molecular sieves (200 mg) in dry MeOH (0.4 mL) and the reaction was stirred at room temperature for 2 hours. Trifluoroacetic acid (18.4 µL, 0.240 mmol) and sodium trifluoroacetate (81.6 mg, 0.600 mmol) were added. The reaction mixture was stirred for further 25 minutes before isocyanide **17** (65.0 mg, 0.202 mmol) was added. The reaction was stirred at room temperature for further 19 hours, filtered through Celite®, and the solvent removed *in vacuo*. The crude product was purified by flash column chromatography (eluting gradient EtOAc:40-60 petroleum ether from 0:1 to 1:3) to give partially separable diastereomeric mixture of protected taltobulin **19a** (80% purity, 28.1 mg, 47.0 µmol, 24%) and protected *epi*-taltobulin **19b** (80% purity, 38.5 mg, 64.4 µmol, 32%) which were used in the next step without further purification.

The protected taltobulin **19a** and **19b** were dissolved separately in H_2O/MeOH (1:1, 3 mL). LiOH•H_2O (16 eq.) was added to each reaction. The reaction mixtures were heated to 60 °C and stirred for 8 days. The mixtures were then purified by reverse-phase semi-preparative HPLC (5-38%B over 70 min for **19a**, 5-36%B over 76 min for **19b**) to give the trifluoroacetate salt of taltobulin **8**•TFA as a white powder (23.0 mg, 39.1 µmol, 20% over two steps) and the trifluoroacetate salt of *epi*-taltobulin **8b**•TFA as a white powder (24.0 mg, 40.8 µmol, 20% over two steps).

8•TFA (*S*,*S*,*S*)

t_R / min = 9.19 (5-95%B over 15 min)

$[\alpha]_D^{25}$ = -26.5° (c = 0.102, MeOH, lit.[4] $[\alpha]_D^{22}$ = -21.7°, c = 0.23)

^{1}H NMR (700 MHz, CD_3OD): δ_H = 7.54 (d, *J* = 7.5 Hz, 2 H), 7.45 (t, *J* = 7.7 Hz, 2 H), 7.35 (t, *J* = 7.3 Hz, 1 H), 6.78 (app dd, *J* = 9.7, 1.4 Hz, 1 H), 5.05 (t, *J* = 10.1 Hz, 1 H), 4.92 (s, 1 H), 4.36 (s, 1 H), 3.14 (s, 3 H), 2.50 (s, 3 H), 2.08-2.01 (m, 1 H), 1.91 (d, *J* = 1.4 Hz, 3 H), 1.47 (s, 3 H), 1.38 (s, 3 H), 1.07 (s, 9 H), 0.92-0.88 (m, 6 H)

^{13}C NMR (176 MHz, CD_3OD): δ_C = 172.3, 170.8, 167.1, 145.0, 139.7, 133.6, 130.4, 128.9, 127.4, 71.3, 58.5, 57.8, 42.1, 36.0, 34.2, 31.9, 30.8, 29.8, 26.9, 21.5, 19.8*, 19.8*, 14.2

*Denoted signals with equal chemical shifts to 1 decimal place appear as two distinguishable signals.

^{19}F NMR (376 MHz, DMSO-d_6): δ_F = -76.9

IR (ATR): $\tilde{\nu}_{max}$ / cm^{-1} = 2966 (m), 1669 (s), 1625 (s)

UV-Vis: λ_{max} / nm = 206, 212 (1 mM in 1:4 MeCN:H_2O)

HRMS (ESI): *m/z* $[M+H]^+$ calcd for $C_{27}H_{44}N_3O_6$: 474.3326; found: 474.3344 (Δ = 3.8 ppm)

Spectroscopic data were in accordance with the literature.[4]

8b•TFA (*R*,*S*,*S*)

t_R / min = 9.32 (5-95%B over 15 min)

$[\alpha]_D^{25}$ = -89.7° (c = 0.107, MeOH, lit.[4] $[\alpha]_D^{22}$ = -96.7°, c = 2.4)

^{1}H NMR (700 MHz, CD_3OD): δ_H = 7.51 (d, *J* = 7.5 Hz, 2 H), 7.44 (t, *J* = 7.8 Hz, 2 H), 7.32 (t, *J* = 7.3 Hz, 1 H), 6.75 (app dd, *J* = 9.7, 1.3 Hz, 1 H), 5.03 (t, *J* = 10.2 Hz, 1 H), 4.55 (s, 1 H), 4.33 (s, 1 H), 3.12 (s, 3 H), 2.47 (s, 3 H), 2.06-1.98 (m, 1 H), 1.88 (d, *J* = 1.3 Hz, 3 H), 1.54 (s, 3 H), 1.48 (s, 3 H), 0.91 (s, 9 H), 0.90-0.86 (m, 6 H)

^{13}C NMR (176 MHz, CD_3OD): δ_C = 173.1, 170.8, 167.7, 145.0, 139.8, 133.6, 130.3, 128.8, 127.4, 71.3, 58.7, 58.5, 41.5, 35.0, 34.0, 31.8, 30.8, 27.1, 27.0, 24.0, 19.8, 19.6, 14.1

^{19}F NMR (376 MHz, DMSO-d_6): δ_F = -76.9

IR (ATR): $\tilde{\nu}_{max}$ / cm^{-1} = 2975 (m), 1669 (s)

UV-Vis: λ_{max} / nm = 206, 214.5 (1 mM in 1:4 MeCN:H_2O)

HRMS (ESI): *m/z* $[M+H]^+$ calcd for $C_{27}H_{44}N_3O_6$: 474.3326; found: 474.3343 (Δ = 3.6 ppm)

Spectroscopic data were in accordance with the literature.[4]

Linker-drug synthesis

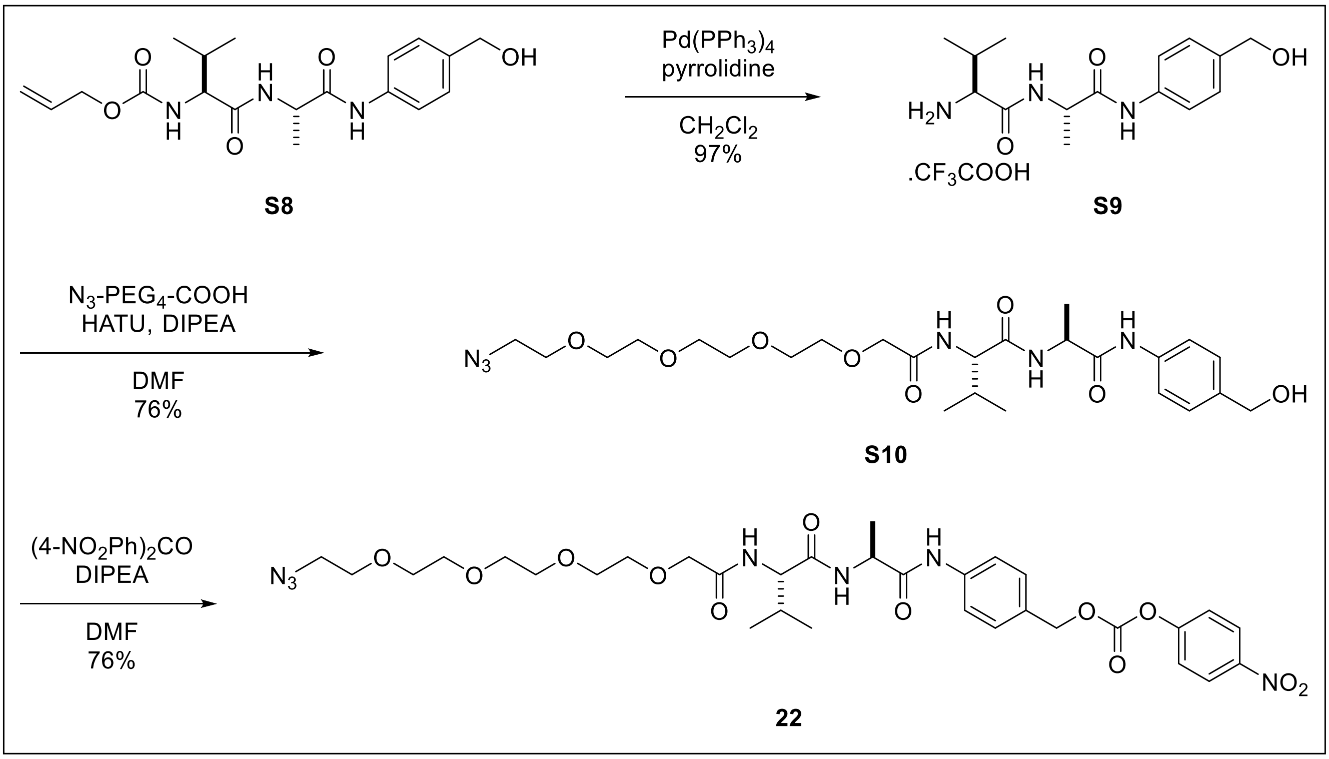

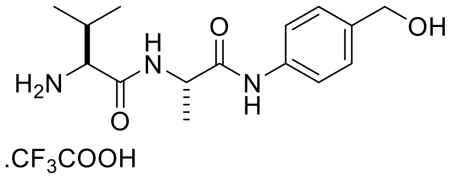

H-Val-Ala-PABA•TFA (S9)

Pyrrolidine was (83.5 µL, 1.00 mmol) was added to a solution of Alloc-Val-Ala-PABA **S8** (189 mg, 0.500 mmol) and $Pd(PPh_3)_4$ (28.9 mg, 0.0250 mmol) in CH_2Cl_2/DMF (7:1, 20 mL). The reaction mixture was stirred at room temperature for 20 h and the volatiles were

removed *in vacuo*. The crude mixture was then purified with reverse-phase semi-preparative HPLC (5-36%B over 8 min) to give the trifluoroacetate salt of amine **S9** as a white powder (198 mg, 0.486 mmol, 97%)

t_R / min = 4.79 (5-95%B over 15 min)

$[\alpha]_D^{25}$ = -31.3° (c = 0.668, MeOH)

^{1}H NMR (400 MHz, CD_3OD): δ_H = 7.53 (d, *J* = 8.2 Hz, 2 H), 7.30 (d, *J* = 8.2 Hz, 2 H), 4.56 (s, 2 H), 4.57-4.49 (m, 1 H), 3.71 (d, *J* = 5.7 Hz, 1 H), 2.23 (octet, *J* = 6.7 Hz, 1 H), 1.47 (d, *J* = 7.1 Hz, 3 H), 1.09 (d, *J* = 6.9 Hz, 3 H), 1.05 (d, *J* = 6.9 Hz)

^{13}C NMR (101 MHz, CD_3OD): δ_C = 172.6, 169.3, 138.7*, 138.7*, 128.6, 121.1, 64.8, 59.6, 51.2, 31.6, 18.9, 18.1, 17.8

*Denoted signals with equal chemical shifts to 1 decimal place appear as two distinguishable signals.

^{19}F NMR (376 MHz, CD_3OD): δ_F = -77.0

IR (ATR): $\tilde{\nu}_{max}$ / cm^{-1}: 3281 (br m), 2971 (m), 1658 (s), 1606 (m)

HRMS (ESI): *m/z* $[M+H]^+$ calcd for $C_{15}H_{24}N_3O_3$: 294.1812; found: 294.1808 (Δ = -1.4 ppm)

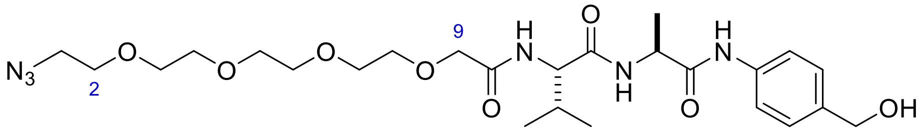

N_3-PEG$_4$-Val-Ala-PABA (S10)

HATU (47.9 mg, 0.126 mmol) was added to a solution of 14-azido-3,6,9,12-tetraoxatetradecanoic acid (0.5 M in TBME, 264 µL, 0.132 mmol) and DIPEA (25.1 µL, 0.144 mmol) in DMF (1 mL) and the mixture was stirred at room temperature for 1 minute. The activated acid solution was then added to the mixture of H-Val-Ala-PABA•TFA **S9** (48.9 mg, 0.120 mmol) and DIPEA (41.8 µL, 0.240 mmol) in DMF (1 mL). The reaction mixture was stirred for 16 hours and then ethyl acetate (40 mL) was added. The mixture was washed successively with saturated aqueous NH_4Cl (2 × 10 mL), saturated aqueous $NaHCO_3$ (2 × 10 mL), and brine (10 mL), then dried over anhydrous Na_2SO_4. The solvent was removed *in vacuo* and the crude product purified using reverse-phase semi-preparative HPLC (10-70%B over 20 min) to give N_3-PEG$_4$-Val-Ala-PABA **S10** as a white solid (50.1 mg, 90.7 µmol, 76%).

t_R / min = 8.17 (5-95%B over 15 min)

^{1}H NMR (400 MHz, CD_3OD): δ_H = 7.54 (d, *J* = 8.6 Hz, 2 H), 7.30 (d, *J* = 8.7 Hz, 2 H), 4.56 (s, 2 H), 4.48 (q, *J* = 7.1 Hz, 1 H), 4.31 (d, *J* = 6.9 Hz, 1 H), 4.06 (s, 2 H), 3.76-3.60 (m, 14 H), 3.35 (t, *J* = 4.9 Hz, 2 H), 2.14 (octet, *J* = 6.8 Hz, 1 H), 1.44 (d, *J* = 7.1 Hz, 3 H), 1.00 (d, *J* = 7.1 Hz, 3 H), 0.97 (d, *J* = 6.8 Hz, 3 H)

^{13}C NMR (101 MHz, CD_3OD): δ_C = 173.0, 172.9, 172.7, 138.8, 138.6, 128.6, 121.1, 72.2*, 71.6, 71.6, 71.5, 71.4, 71.1*, 64.8, 59.4, 51.8, 51.1, 32.4, 19.8, 18.7, 18.0

*Signals from 72.2 to 71.1 ppm (inclusive) overlap greatly and only six distinguishable signals for C-2 to C-9 were observed.

IR (ATR): $\tilde{\nu}_{max}$ / cm^{-1} = 3282 (m), 2873 (m), 2098 (m), 1662 (m), 1630 (m)

HRMS (ESI): *m/z* $[M+Na]^+$ calcd for $C_{25}H_{40}N_6{}^{23}NaO_8$: 575.2800; found: 575.2809 (Δ = 1.6 ppm)

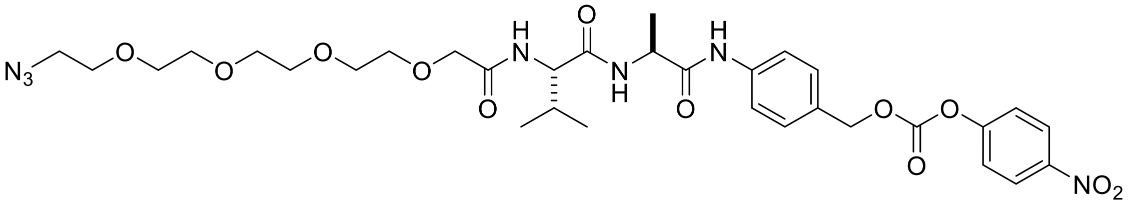

N_3-PEG$_4$-Val-Ala-PABC-OPNP (22)

DIPEA (176 µL, 1.01 mmol) was added to a solution of N_3-PEG$_4$-Val-Ala-PABA **S10** (112 mg, 0.202 mmol) and bis(4-nitrophenyl)carbonate (92.2 mg, 0.303 mmol) in DMF (1 mL) and the reaction mixture was stirred at room temperature for 16 h before ethyl acetate (50 mL) was added. The mixture was washed with saturated aqueous solution of Na_2CO_3 (6 × 15 mL), dried over anhydrous $MgSO_4$, and the solvent was removed *in vacuo*. The crude product was purified by flash column chromatography (eluting gradient MeOH:CH_2Cl_2 from 0:1 to 1:20) to give N_3-PEG$_4$-Val-Ala-PABC-OPNP **22** as a colourless oil which solidified under stream of N_2 over several days (110 mg, 0.153 mmol, 76%).

R_f = 0.19 (MeOH/CH_2Cl_2 = 1:20)

^{1}H NMR (400 MHz, $CDCl_3$): δ_H = 8.90 (s, 1 H), 8.28-8.23 (m, 2 H), 7.63-7.58 (m, 2 H), 7.41-7.34 (m, 5 H), 7.19 (br d, *J* = 7.2 Hz, 1 H), 5.23 (s, 2 H), 4.64 (qn, *J* = 7.1 Hz, 1 H), 4.33 (dd, *J* = 7.9, 7.0 Hz, 1 H), 4.11, 4.04 (ABq, J_{AB} = 15.8 Hz, 2 H), 3.76-3.61 (m, 14 H), 3.37 (t, *J* = 5.0 Hz, 2 H), 2.21 (octet, *J* = 6.7 Hz, 1 H), 1.45 (d, *J* = 7.1 Hz, 3 H), 0.99 (d, *J* = 6.8 Hz, 3 H), 0.98 (d, *J* = 6.8 Hz, 3 H)

^{13}C NMR (101 MHz, $CDCl_3$): δ_C = 171.7, 171.1, 170.4, 155.6, 152.5, 145.5, 138.9, 129.9, 129.8, 125.4, 121.9, 120.0, 71.3*, 70.8, 70.8, 70.7, 70.7, 70.7, 70.4, 70.4, 70.1*, 58.8, 50.8, 49.9, 30.6, 19.4, 18.4, 17.2

*Signals between 71.3 and 70.1 ppm which have the same chemical shifts to 1 decimal place are distinguishable.

IR (ATR): $\tilde{\nu}_{max}$ / cm^{-1} = 3273 (m), 2871 (m), 2097 (m), 1759 (m), 1665 (m), 1633 (s)

HRMS (ESI): *m/z* [M+Na]$^+$ calcd for $C_{32}H_{43}N_7{}^{23}NaO_{12}$: 740.2862; found: 740.2871 (Δ = 1.2 ppm)

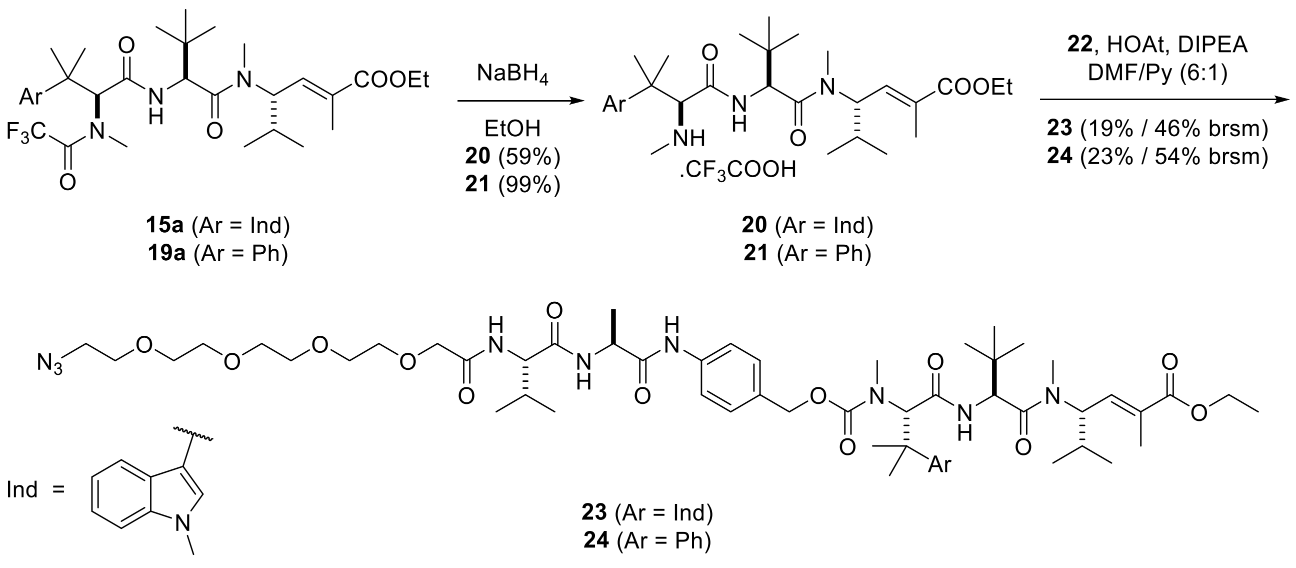

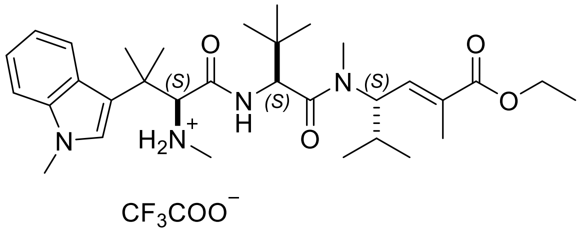

Hemiasterlin ethyl ester trifluoroacetate salt (20)

$NaBH_4$ (11.8 mg, 0.310 mmol) was added to a solution of *N*-trifluoroacetyl hemiasterlin ethyl ester **15a** (37.0 mg, 56.9 μmol) in EtOH (5 mL) and the reaction mixture was stirred at room temperature for 24 h. The reaction was quenched by an addition of H_2O (10 mL) and then extracted with CH_2Cl_2 (4 × 10 mL). The combined organic phases were dried over anhydrous $MgSO_4$, and the solvent was removed *in vacuo*. The crude product was purified by reverse-phase semi-preparative HPLC (5-45%B over 20 min) to give trifluoroacetate salt of amine **20** as a white powder (22.6 mg, 33.8 μmol, 59%).

t_R / min = 11.51 (5-95%B over 15 min)

$[\alpha]_D^{25}$ = -33.8° (c = 0.346, MeOH)

^{1}H NMR (400 MHz, CD_3OD): δ_H = 8.09 (d, *J* = 8.1 Hz, 1 H), 7.44 (d, *J* = 8.2 Hz, 1 H), 7.26 (ddd, *J* = 8.1, 7.1, 0.9 Hz, 1 H), 7.15 (ddd, *J* = 8.0, 7.0, 0.9 Hz, 1 H), 7.14 (s, 1 H), 6.77 (app dd, *J* = 9.7, 1.5 Hz, 1 H), 5.07 (t, *J* = 10.6 Hz, 1 H), 4.95 (s, 1 H), 4.54 (s, 1 H), 4.21 (q, *J* = 7.1 Hz, 2 H), 3.81 (s, 3 H), 3.15 (s, 3 H), 2.45 (s, 3 H), 2.12-2.00 (m, 1 H), 1.93 (d, *J* = 1.4 Hz, 3 H), 1.58 (s, 3 H), 1.47 (s, 3 H), 1.30 (t, *J* = 7.1 Hz, 3 H), 1.05 (s, 9 H), 0.91 (d, *J* = 6.7 Hz, 3 H), 0.90 (d, *J* = 6.7 Hz, 3 H)

^{13}C NMR (101 MHz, CD_3OD): δ_C = 172.4, 169.1, 167.1, 140.0, 139.7, 133.4, 129.5, 126.2, 123.1, 121.0, 120.4, 117.5, 111.2, 69.8, 62.1, 58.3, 57.9, 39.5, 35.9, 34.3, 33.0, 32.0, 30.8, 27.4, 26.9, 23.2, 19.8*, 19.8*, 14.5, 14.2

*Denoted signals with equal chemical shifts to 1 decimal place appear as two distinguishable signals.

^{19}F NMR (376 MHz, CD_3OD): δ_F = -77.1

IR (ATR): $\tilde{\nu}_{max}$ / cm^{-1} = 2966 (m), 1670 (s)

HRMS (ESI): *m/z* $[M+H]^+$ calcd for $C_{32}H_{51}N_4O_4$: 555.3905; found: 555.3907 (Δ = 0.4 ppm)

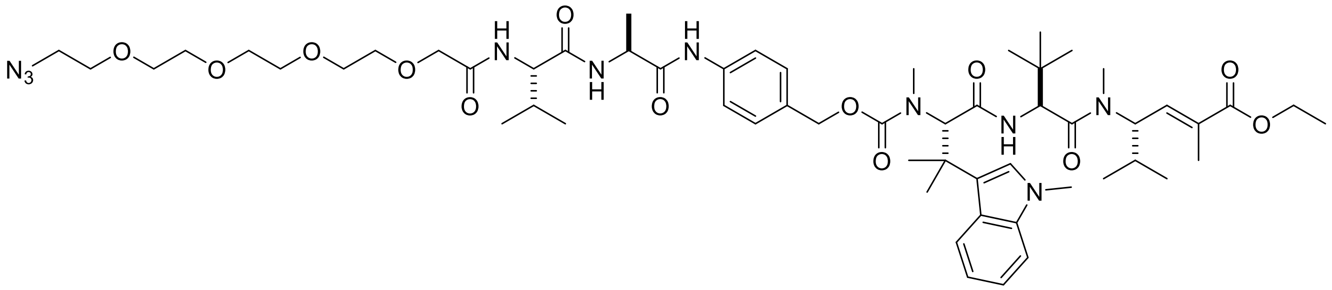

N_3-PEG$_4$-Val-Ala-PABC-hemiasterlin-OEt (23)

To a solution of hemiasterlin ethyl ester trifluoroacetate salt **20** (18.9 mg, 28.3 μmol), N_3-PEG$_4$-Val-Ala-PABC-OPNP **22** (43.1 mg, 60.0 μmol), and HOAt (4.08 mg, 30.0 μmol) in DMF (540 μL) was added pyridine (90 μL, 1.11 mmol) and DIPEA (20.9 μL, 120 μmol). The reaction was stirred at room temperature for 7 days before the volatiles were removed under a stream of N_2. The crude mixture was purified by reverse-phase semi-preparative HPLC (20-95%B over 20 min) to give carbamate **23** as a white solid (6.1 mg, 5.4 μmol, 19%, 46%brsm) and the starting material hemiasterlin ethyl ester trifluoroacetate salt was recovered (11.1 mg, 16.6 μmol, 59%).

t_R / min = 14.68 (5-95%B over 15 min)

^{1}H NMR (400 MHz, $CDCl_3$): Unassigned due to complex rotameric mixture.

HRMS (ESI): *m/z* $[M+H]^+$ calcd for $C_{58}H_{89}N_{10}O_{13}$: 1133.6611; found: 1133.6606 (Δ = -0.4 ppm)

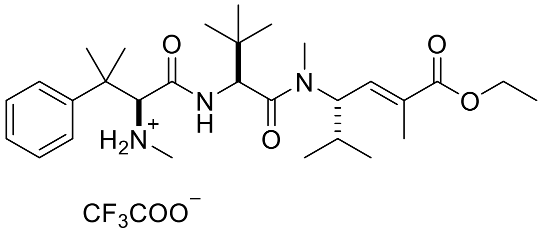

Taltobulin ethyl ester trifluoroacetate salt (21)

$NaBH_4$ (25.5 mg, 0.670 mmol) was added to a solution of *N*-trifluoroacetyl taltobulin ethyl ester **19a** (3.35:1 dr, 80.3 mg, 0.134 mmol) in EtOH (10 mL) and the reaction mixture was stirred at room temperature for 24 h. The reaction was quenched by an addition of H_2O (15 mL) and then extracted with CH_2Cl_2 (4 × 15 mL). The combined organic phases were dried over anhydrous $MgSO_4$, and the solvent was removed *in vacuo*. The crude product was purified by reverse-phase semi-preparative HPLC (5-45%B over 75 min) to give amine **21** as a white powder (62.8 mg, 0.102 mmol, 99% w.r.t. the desired isomer).

t_R / min = 10.87 (5-95%B over 15 min)

$[\alpha]_D^{25}$ = -43.5° (c = 0.524, MeOH)

^{1}H NMR (400 MHz, CD_3OD): δ_H = 7.54 (d, *J* = 8.0 Hz, 2 H), 7.45 (t, *J* = 7.6 Hz, 2 H), 7.35 (t, *J* = 7.2 Hz, 1 H), 6.76 (d, *J* = 9.7 Hz, 1 H), 5.05 (t, *J* = 10.1 Hz, 1 H), 4.91 (s, 1 H), 4.36 (s, 1 H), 4.21 (q, *J* = 7.2 Hz, 2 H), 3.14 (s, 3 H), 2.50 (s, 3 H), 2.11-1.99 (m, 1 H), 1.93 (s, 3 H), 1.47 (s, 3 H), 1.38 (s, 3 H), 1.30 (t, *J* = 7.1 Hz, 3 H), 1.06 (s, 9 H), 0.90 (d, *J* = 6.5 Hz, 6 H)

^{13}C NMR (101 MHz, CD_3OD): δ_C = 172.3, 169.1, 167.0, 145.0, 139.7, 133.5, 130.4, 128.9, 127.4, 71.2, 62.1, 58.3, 57.8, 42.1, 35.9, 34.2, 31.9, 30.8, 29.8, 26.9, 21.5, 19.8*, 19.8*, 14.5, 14.2

*Denoted signals with equal chemical shifts to 1 decimal place appear as two distinguishable signals.

^{19}F NMR (376 MHz, CD_3OD): δ_F = -77.2

IR (ATR): $\tilde{\nu}_{max}$ / cm^{-1} = 2970 (m), 1713 (m), 1669 (s), 1627 (s)

HRMS (ESI): *m/z* $[M+H]^+$ calcd for $C_{29}H_{48}N_3O_4$: 502.3639; found: 502.3648 (Δ = 1.8 ppm)

Spectroscopic data were in accordance with the literature.[4]

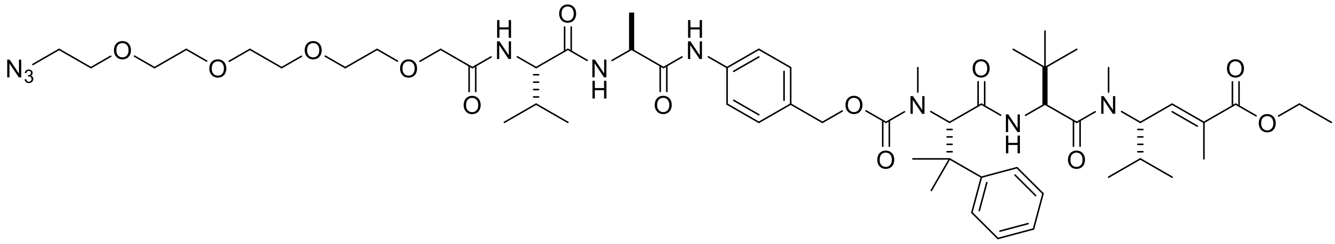

N_3-PEG$_4$-Val-Ala-PABC-taltobulin-OEt (24)

To a solution of taltobulin ethyl ester trifluoroacetate salt **21** (24.6 mg, 40.0 µmol), N_3-PEG$_4$-Val-Ala-PABC-OPNP **22** (57.4 mg, 60.0 µmol), and HOAt (5.44 mg, 40.0 µmol) in DMF (720 µL) was added pyridine (120 µL, 1.48 mmol) and DIPEA (27.9 µL, 160 µmol). The reaction was stirred at room temperature for 7 days before the volatiles were removed under a stream of N_2. The crude mixture was purified by reverse-phase semi-preparative HPLC (20-95%B over 20 min) to give carbamate **24** as a yellow solid (10.1 mg, 9.35 µmol, 23%, 54%brsm) and the starting material taltobulin ethyl ester trifluoroacetate salt was recovered (14.0 mg, 22.7 µmol, 57%).

t_R / min = 14.70 (5-95%B over 15 min)

^{1}H NMR (600 MHz, $CDCl_3$): Unassigned due to complex rotameric mixture.

HRMS (ESI): *m/z* $[M+Na]^+$ calcd for $C_{55}H_{85}N_9{}^{23}NaO_{13}$: 1102.6159; found: 1102.6146 (Δ = -1.2 ppm)

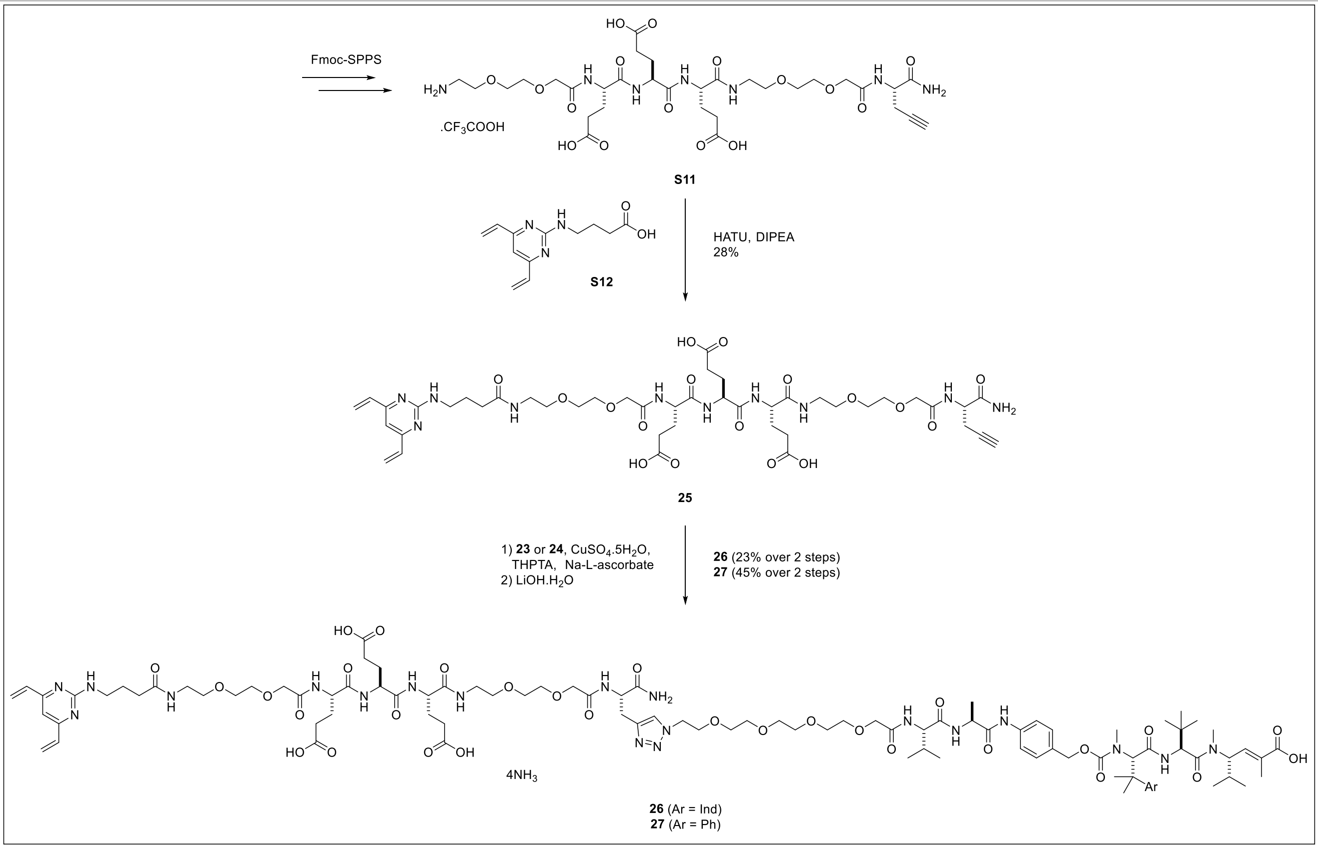

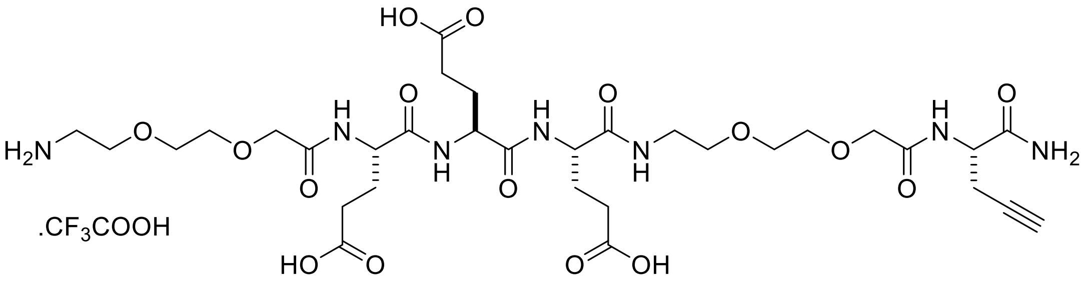

H-PEG$_2$-Glu$_3$-PEG$_2$-propargylGly-NH$_2$•TFA (S11)

The peptide was synthesised by standard manual Fmoc solid-phase peptide synthesis (Fmoc-SPPS) on LL Rink Amide resin (0.51 mmol/g, 1.00 g). Fmoc-deprotection was performed using 20% piperidine solution in DMF (1 × 1 min, 2 × 5 min).

The coupling was performed by the following: Fmoc-protected amino acids (4 eq.) were activated with HATU (4 eq.) and DIPEA (8 eq.) in DMF (0.4 M of amino acid) for 10 s. The mixture was then added to the resin and agitated by a stream of N_2 for 5 min. The *t*Bu protecting group was used for the side-chain of Glu.

Completion of peptide couplings were monitored using chloranil test. To a small amount of resin swelled in CH_2Cl_2 was added acetaldehyde (200 µL) then followed by saturated chloranil solution in toluene (50 µL). The mixture was shaken for 5 min at room temperature. Resin beads remaining colourless indicated a complete coupling whereas green colouration indicated an incomplete coupling. Any incomplete couplings were subjected to a second round of coupling.

Peptide cleavage and global deprotection of all amino acid side chains was achieved by the treatment of dried resin with TFA/CH_2Cl_2/H_2O/TIPS (92.5:2.5:2.5:2.5, 20 mL) at 42 °C for 40 min. The mixture was then filtered through cotton wool, and volatiles were removed under a stream of nitrogen. The crude peptides were triturated with Et_2O and then washed with Et_2O (3 × 15 mL) before purification by reverse-phase semi-preparative HPLC (5-25%B over 9 min) to give the peptide **S11** as a hygroscopic white powder (285 mg, 0.315 mmol, 62% yield).

t$_R$ / min = 5.06 (5-95%B over 15 min)

HRMS (ESI): *m/z* [M-H]$^-$ calcd for $C_{32}H_{50}N_7O_{16}$: 788.3314; found: 788.3320 (Δ = -0.8 ppm)

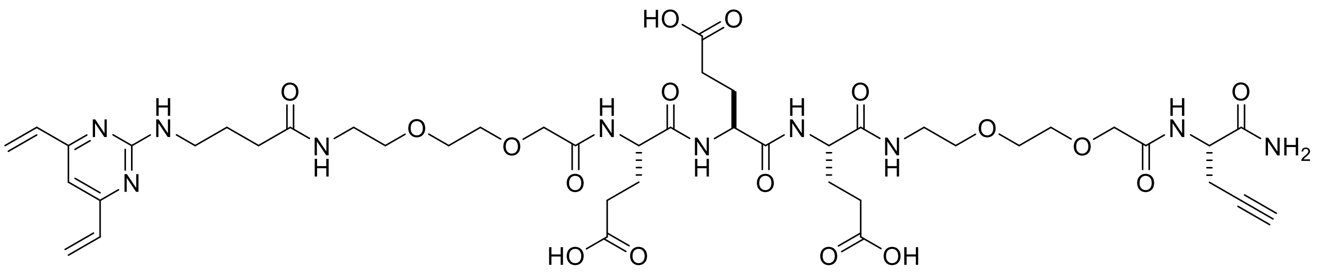

DVP-PEG$_2$-Glu$_3$-PEG$_2$-propargylGly-NH$_2$ (25)

DIPEA (11.0 µL, 63.0 µmol) was added to a solution of 4-((4,6-divinylpyrimidin-2-yl)amino)butanoic acid[7] **S12** (12.3 mg, 52.5 µmol) and HATU (19.0 mg, 50.0 µmol) in DMF (0.5 mL) and the solution was stirred at room temperature for 5 min. The activated acid solution was then added to a solution of peptide **S11** (43.2 mg, 47.8 µmol) and DIPEA (69.7 µL, 400 µmol) in DMF (0.5 mL) and the reaction mixture was stirred at room temperature for 1 h before purification by automatic reverse-phase flash column chromatography to give alkynyl DVP **25** as a white powder (13.4 mg, 13.3 µmol, 28%).

t$_R$ / min = 5.68 (5-95%B over 15 min)

HRMS (ESI): *m/z* $[M]^+$ calcd for $C_{44}H_{64}N_{10}O_{17}$: 1004.4409; found: 1004.4404 (Δ = 0.5 ppm)

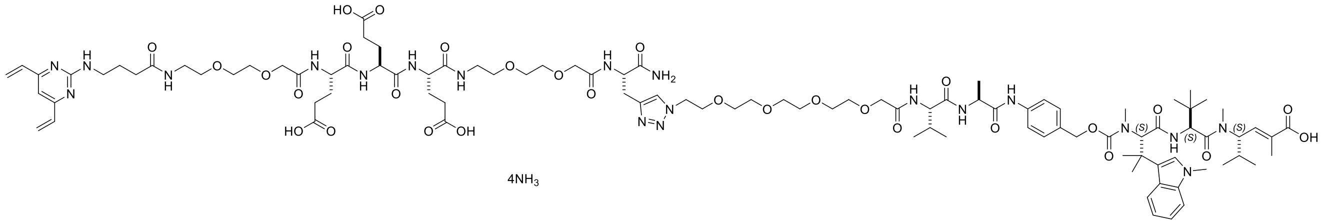

DVP-PEG$_2$-Glu$_3$-PEG$_2$-triazole-PEG$_4$-Val-Ala-PABC-hemiasterlin ammonium salt (26)

$CuSO_4•5H_2O$ (0.29 mg, 1.2 µmol) and THPTA (0.84 mg, 1.9 µmol) were dissolved in a degassed mixture of H_2O/*t*BuOH (1:1, 0.5 mL) and stirred at room temperature for 1 minute before Na-L-ascorbate (0.96 mg, 4.9 µmol) was added. The colourless copper solution was then added to a degassed solution of azide **23** (1.10 mg, 0.971 µmol) and alkyne **25** (1.95 mg, 1.94 µmol) in H_2O/*t*BuOH (0.5 mL). The reaction mixture was stirred at room temperature for 3 h before the solvent was removed by lyophilisation. The resulting crude triazole ester was re-dissolved in H_2O/MeOH (1:1, 1 mL). Aqueous $LiOH•H_2O$ solution (0.357 M, 13.6 µL, 4.86 µmol) was added to the solution and the reaction was stirred at room temperature for 6 days during which extra $LiOH•H_2O$ was added until the reaction went to completion. The volatiles were then removed *in vacuo* and the resulting crude product was purified by automatic reverse-phase flash column chromatography to give linker-drug **26** as a white powder (0.49 mg, 0.22 µmol, 23% over two steps).

t$_R$ / min = 10.66 (5-95%B over 15 min)

HRMS (ESI): *m/z* $[M+2H]^{2+}$ calcd for $C_{100}H_{150}N_{20}O_{30}$: 1055.5408; found: 1055.5422 (Δ = 1.3 ppm)

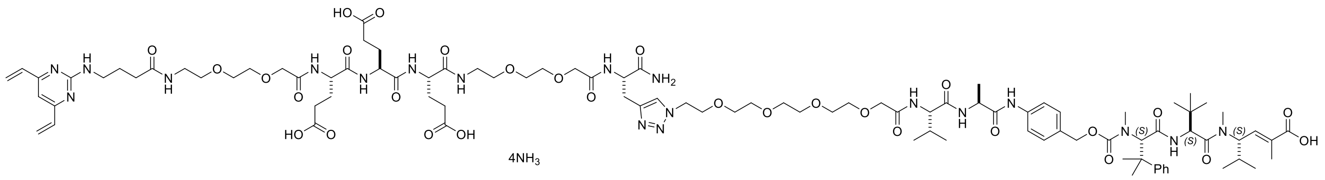

DVP-PEG$_2$-Glu$_3$-PEG$_2$-triazole-PEG$_4$-Val-Ala-PABC-taltobulin ammonium salt (27)

$CuSO_4•5H_2O$ (0.53 mg, 2.1 µmol) and THPTA (1.53 mg, 3.52 µmol) were dissolved in a degassed mixture of H_2O/*t*BuOH (1:1, 0.9 mL) and stirred at room temperature for 1 minute before Na-L-ascorbate (1.74 mg, 8.78 µmol) was added. The colourless copper solution was then added to a degassed solution of azide **24** (1.90 mg, 1.76 µmol) and alkyne **25** (3.54 mg, 3.52 µmol) in H_2O/*t*BuOH (0.9 mL). The reaction mixture was stirred at room temperature for 3 h before the solvent was removed by lyophilisation. The resulting crude triazole ester was re-dissolved in H_2O/MeOH (1:1, 2 mL). Aqueous $LiOH•H_2O$ solution (0.357 M, 24.6 µL, 8.78 µmol) was added to the

solution and the reaction was stirred at room temperature for 5 days during which extra LiOH•H_2O was added until the reaction went to completion. The volatiles were then removed *in vacuo* and the resulting crude product was purified by automatic reverse-phase flash column chromatography to give linker-drug **27** as a white powder (1.70 mg, 0.800 µmol, 45% over two steps).

t_R / min = 10.63 (5-95%B over 15 min)

HRMS (ESI): *m/z* $[M+2H]^{2+}$ calcd for $C_{97}H_{147}N_{19}O_{30}$: 1029.0275; found: 1029.0275 (Δ = 0.0 ppm)

Reaction condition screening

Fragment amide coupling

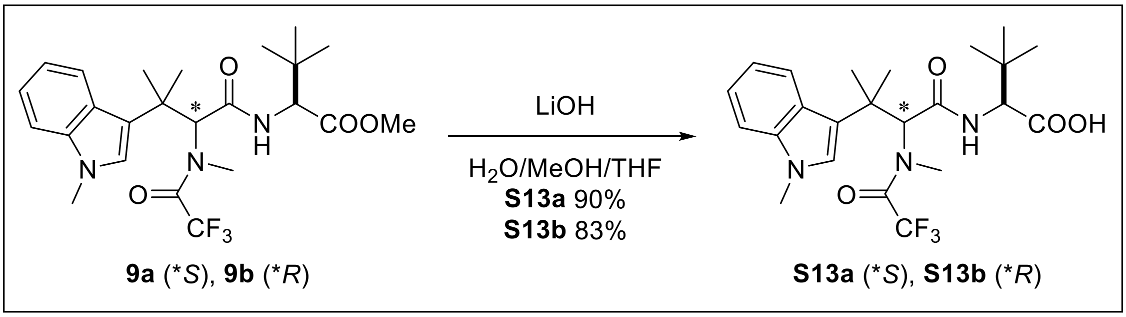

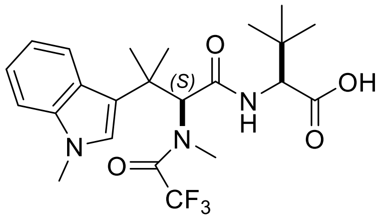

(*S*)-3,3-dimethyl-2-((*S*)-3-methyl-3-(1-methyl-1*H*-indol-3-yl)-2-(2,2,2-trifluoro-*N*-methylacetamido)butanamido)butanoic acid (S13a)

To a solution of ester **9a** (96.7 mg, 0.200 mmol) in MeOH/THF (1:1, 8 mL) was added an aqueous solution of LiOH (0.25 M, 4.00 mL, 1.00 mmol) which resulted in a cloudy reaction mixture. The reaction mixture was stirred at room temperature for 4 days before H_2O (12 mL) was added and then washed with Et_2O (2 × 10 mL). The aqueous phase was then acidified with 5% aqueous H_3PO_4 to pH 2-3 and was then extracted with EtOAc (3 × 10 mL). The combined organic phases were dried over anhydrous Na_2SO_4 and the solvent was removed *in vacuo* to give the crude acid **S13a** as a white solid (84.4 mg, 0.180 mmol, 90%) which was used in the next step without further purification.

mp 200 °C (dec)

$[\alpha]_D^{25}$ = -15.6° (c = 0.343, $CHCl_3$)

^{1}H NMR (400 MHz, $CDCl_3$): δ_H = 8.26 (d, *J* = 8.0 Hz, 1 H), 7.35-7.20 (m, 3 H), 7.03 (s, 1 H), 6.35 (s, 1 H), 5.89 (d, *J* = 8.1 Hz, 1 H), 4.02 (d, *J* = 8.6 Hz, 1 H), 3.74 (s, 3 H), 3.49 (s, 3 H), 1.71 (s, 3 H), 1.51 (s, 3 H), 0.50 (s, 9 H)

^{13}C NMR (101 MHz, $CDCl_3$): δ_C = 175.5, 168.9, 159.5 (app d, $^2J_{C\text{-}F}$ = 35.5 Hz), 138.2, 127.0, 124.7, 122.7, 121.0, 120.6, 120.3, 117.0 (app d, $^1J_{C\text{-}F}$ = 287.5 Hz), 109.9, 63.0, 60.6, 39.5, 35.0 (q, $^4J_{C\text{-}F}$ = 3.8 Hz), 33.6, 32.8, 27.5, 26.0, 24.5

^{19}F NMR (376 MHz, $CDCl_3$): δ_F = -69.5

IR (ATR): $\tilde{\nu}_{max}$ / cm^{-1} = 2965 (m), 1663 (s)

HRMS (ESI): *m/z* $[M-H]^-$ calcd for $C_{23}H_{29}F_3N_3O_4$: 468.2116; found: 468.2106 (Δ = -2.1 ppm)

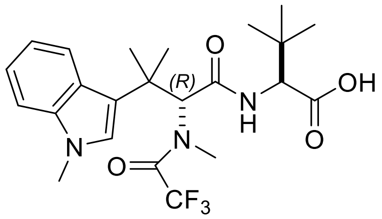

(*S*)-3,3-dimethyl-2-((*R*)-3-methyl-3-(1-methyl-1*H*-indol-3-yl)-2-(2,2,2-trifluoro-*N*-methylacetamido)butanamido)butanoic acid (S13b)

To a solution of ester **9b** (96.7 mg, 0.200 mmol) in MeOH/THF (1:1, 8 mL) was added an aqueous solution of LiOH (0.25 M, 4.00 mL, 1.00 mmol) which resulted in a cloudy reaction mixture. The reaction mixture was stirred at room temperature for 4 days before H_2O (12 mL) was added and then washed with Et_2O (2 × 10 mL). The aqueous phase was then acidified with 5% aqueous H_3PO_4 to pH 2-3 and was then extracted with EtOAc (3 × 10 mL). The combined organic phases were dried over anhydrous Na_2SO_4 and the solvent was removed *in vacuo* to give the crude acid **S13b** as a white solid (78.4 mg, 0.167 mmol, 83%) which was used in the next step without further purification.

mp 104-108 °C

$[\alpha]_D^{25}$ = +55.0° (c = 0.420, $CHCl_3$)

^{1}H NMR (400 MHz, $CDCl_3$): δ_H = 8.13 (d, *J* = 7.5 Hz, 1 H), 7.29-7.12 (m, 3 H), 6.92 (s, 1 H), 6.15 (s, 1 H), 5.94 (d, *J* = 8.7 Hz, 1 H), 4.06 (d, *J* = 8.7 Hz, 1 H), 3.71 (s, 3 H), 3.45 (s, 3 H), 1.74 (s, 3 H), 1.50 (s, 3 H), 0.50 (s, 9 H)

^{13}C NMR (101 MHz, $CDCl_3$): δ_C = 174.8, 167.8, 159.4 (app d, $^2J_{C\text{-}F}$ = 35.8 Hz), 138.2, 126.5, 125.2, 122.3, 120.7, 120.2, 120.0, 116.9 (app d, $^1J_{C\text{-}F}$ = 288.2 Hz), 110.0, 63.1, 60.1, 39.8, 34.2 (q, $^4J_{C\text{-}F}$ = 4.3 Hz), 34.1, 32.8, 27.4, 26.0, 24.6

^{19}F NMR (376 MHz, $CDCl_3$): δ_F = -69.6

IR (ATR): $\tilde{\nu}_{max}$ / cm^{-1} = 2968 (m), 1738 (m), 1665 (s)

HRMS (ESI): *m/z* [M-H]$^-$ calcd for $C_{23}H_{29}F_3N_3O_4$: 468.2116; found: 468.2107 (Δ = -1.9 ppm)

Table S1 Screening of conditions for fragment amide coupling of **S13** with **14**

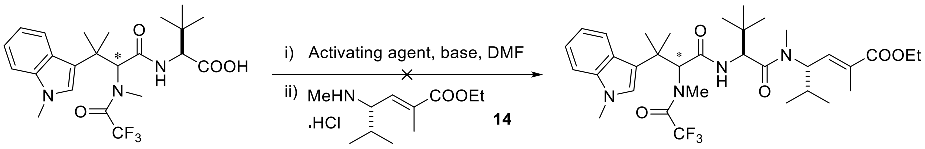

S13a (**S*), **S13b** (**R*) **15a** (**S*), **15b** (**R*)

Conditions	%yield
HBTU, DIPEA, DCM[8],a	0c
HATU, DIPEA, DMF[9],a	0
EDCI, HOAt, NMM, DMF[10],a	0
DIC, HOAt, $CuCl_2$, DIPEA, DMF[11],b	0
DIC, NMM, $CuCl_2$, DMF[11],b	0
BTFFH, DIPEA, DCM, 80 °C (sealed tube)[12],b	0
BTFFH, DIPEA, DCM, 80 °C (microwave) [12],b	0

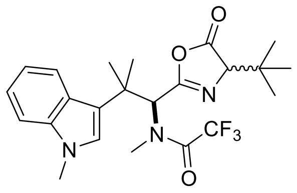

S14

[a]**S13a** was used; [b]**S13b** was used; [c]Oxazolone **S14** (59% yield) was isolated.

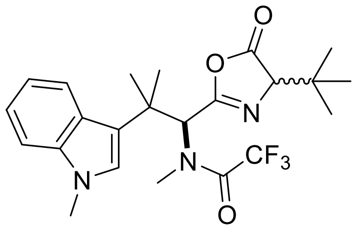

Oxazolone S14 (as an inseparable diastereomeric mixture of 1.7:1 *dr*)

***R**$_f$* = 0.28 (EtOAc/40-60 petroleum ether = 1:5)

^{1}H NMR (400 MHz, $CDCl_3$): Major diastereomer δ_H = 7.97 (d, *J* = 8.0 Hz, 1 H), 6.29 (d, *J* = 1.1 Hz, 1 H), 3.80 (d, *J* = 1.4 Hz, 1 H), 1.83 (s, 3 H), 1.60 (s, 3 H), 0.78 (s, 9 H); Minor diastereomer δ_H = 7.93 (d, *J* = 8.1 Hz, 1 H), 6.22 (d, *J* = 1.4 Hz, 1 H), 1.80 (s, 3 H), 1.62 (s, 3 H), 0.87 (s, 9 H); Overlapping / indistinguishable signals* δ_H = 7.30-7.26 (m, 1 H), 7.25-7.18 (m, 1 H), 7.17-7.11 (m, 1 H), 6.93-6.90 (m, 1 H), 3.75-3.72 (m, 3.4 H), 3.21-3.17 (m, 3 H)

*The numbers of proton nuclei quoted are with respect to the sum of both diastereomers.

^{13}C NMR (101 MHz, $CDCl_3$): Major diastereomer δ_C = 175.9, 160.6, 158.7 (app d, $^2J_{C\text{-}F}$ = 36.1 Hz), 137.6, 127.1, 125.6, 121.7‡, 120.9, 119.5, 119.4‡, 116.6† (app d, $^1J_{C\text{-}F}$ = 287.6 Hz), 109.6‡, 73.4, 59.6, 39.7, 35.5, 33.8 (q, $^4J_{C\text{-}F}$ = 4.4 Hz), 32.8, 27.4, 25.8, 25.4; Minor diastereomer δ_C = 176.1, 161.2, 158.5 (app d, $^2J_{C\text{-}F}$ = 36.1 Hz), 137.7, 127.0, 125.9, 121.7‡, 121.0, 119.4‡, 119.0‡, 116.6† (app d, $^1J_{C\text{-}F}$ = 287.6 Hz), 109.6‡, 73.1, 60.4, 39.8, 35.4, 33.9 (q, $^4J_{C\text{-}F}$ = 3.9 Hz), 32.7, 27.4‡, 26.2, 26.1

‡Denoted signals with equal chemical shifts to 1 decimal place appear as two distinguishable signals.

†Appear as a single signal.

^{19}F NMR (376 MHz, $CDCl_3$): Major diastereomer δ_F = -70.8; Minor diastereomer δ_F = -70.7

IR (ATR): $\tilde{\nu}_{max}$ / cm^{-1} = 2977 (m), 1822 (s), 1676 (s)

HRMS (ESI): *m/z* $[M+H]^+$ calcd for $C_{23}H_{29}F_3N_3O_3$: 452.2161 ; found: 452.2158 (Δ = -0.7 ppm)

Attempt at improving diastereoselectivity of the Ugi reaction

Methylamine solution in MeOH or THF (2 M, 27.5 µL, 0.0550 mmol) was added to a mixture of 2-methyl-2-(1-methyl-1*H*-indol-3-yl)propanal (**12**) (10.1 mg, 0.0500 mmol) and 3 Å molecular sieves (200 mg) in dry solvent (1 mL). The reaction was stirred at room temperature for 2 hours before trifluoroacetic acid (4.59 µL, 0.0600 mmol), additives, and a chiral phosphoric acid (2.5 µmol) were added. The reaction mixture was stirred for further 30 minutes before isocyanide **17** (17.7 mg, 0.0550 mmol) was added. The reaction was stirred at room temperature for further 19 hours, filtered through Celite®, eluted with copious amount of MeOH, and the solvent removed *in vacuo*. The crude product was analysed by ^{19}F-NMR using 4,4'-difluorobenzophenone as an internal standard.

Table S2 Screening of conditions for improving diastereoselectivity of the Ugi reaction for generating **15**

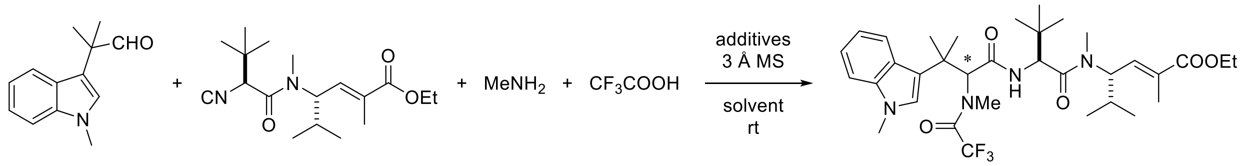

12 **17** 1.1 eq. 1.1 eq. 1.2 eq. **15a** (*S), **15b** (*R)

Entry	Solvent (0.05 M)	Additives (1.2 eq.)	Catalyst (5 mol%)	%yield (NMR)*	*dr*† = (*S*):(*R*)
1‡	MeOH (0.5 M)	CF_3COONa	-	73‖	1:1.4
2	MeOH	CF_3COONa	-	20	1:1.3
3	MeOH	CF_3COONa	**CPA1**	15	1:1.4
4	CH_2Cl_2	CF_3COONa	-	7	1:1.5

5	CH_2Cl_2	CF_3COOH, Et_3N	-	58	1:1.4
6	CH_2Cl_2	CF_3COOH, Et_3N	**CPA1**	15	1:1.3
7	CH_2Cl_2	CF_3COOH, Et_3N	**CPA2**	31	1:1.3
8	CH_2Cl_2	CF_3COOH, Et_3N	**CPA3**	22	1:1.3
9	CH_2Cl_2	CF_3COOH, Et_3N	**CPA4**	6	1:1.4

*Analysed by ^{19}F-NMR using 4,4'-difluorobenzophenone as the internal standard. †Determined with ^{19}F-NMR of the crude product. ‡1.2 eq. of **17**. ǁIsolated yield.

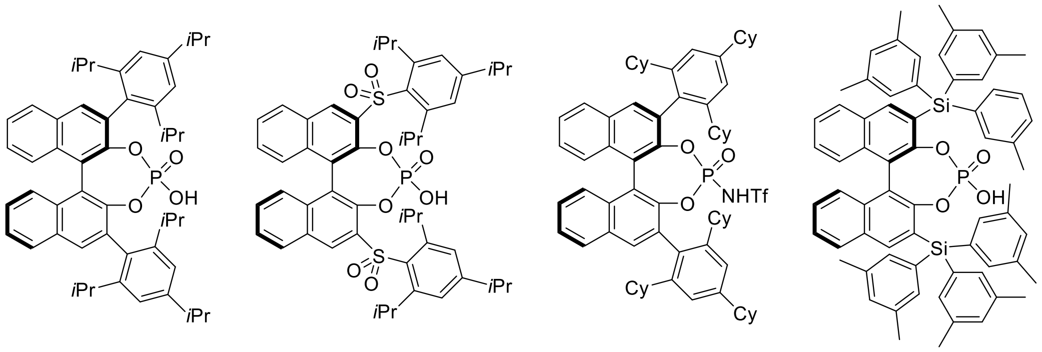

CPA1 **CPA2** **CPA3** **CPA4**

ADC synthesis

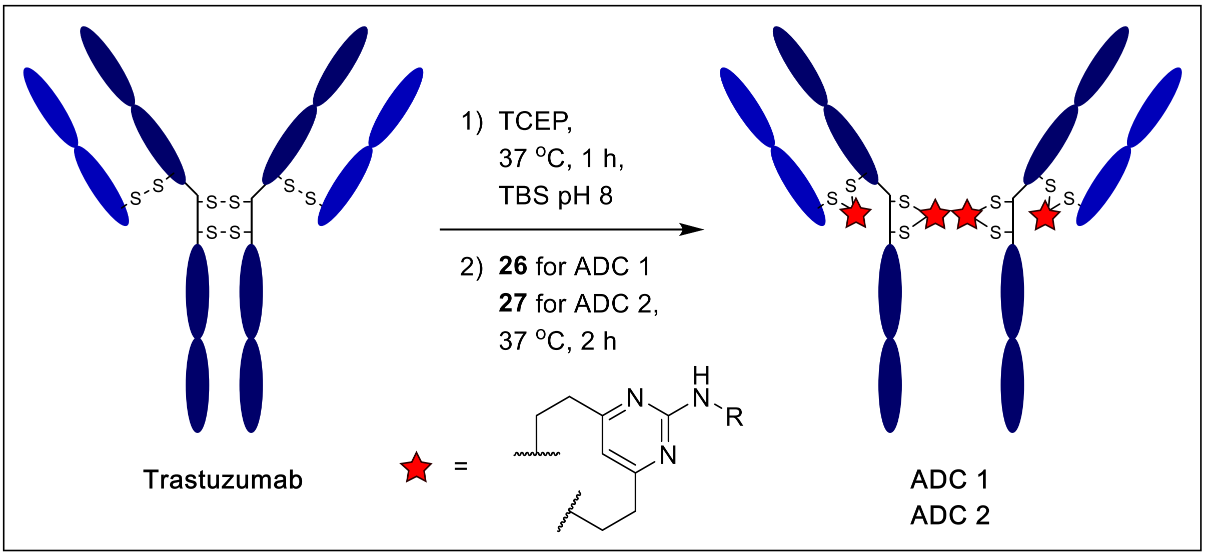

The ADCs were synthesised following literature procedure[13]:
To a solution of trastuzumab (50 µL, 17 µM, 2.5 mg/mL) in TBS buffer (25 mM Tris•HCl pH 8, 25 mM NaCl, 0.5 mM EDTA) was added TCEP solution (5 mM, 10 eq.). The reaction mixture was vortexed and incubated at 37 °C for 1 h. The linker-drug stock solution in DMSO (20 mM, 60 eq.) was added (final DMSO percentage at 10% v/v) and the reaction mixture was vortexed and incubated at 37 °C for 4 h. The excess reagents were removed using a Zeba™ Spin desalting column (40,000 MWCO, 0.5 mL, ThermoFisher) pre-equilibrated with PBS. The reaction product was completely buffer exchanged into PBS by repeated diafiltration using an Amicon-Ultra centrifugal filter (10,000 MWCO, Merck Millipore). LCMS and SDS-PAGE analysis demonstrated >95% conversion to the desired conjugate.

ADC 1
ADC 1 was synthesised by using DVP-PEG$_2$-Glu$_3$-PEG$_2$-triazole-PEG$_4$-Val-Ala-PABC-hemiasterlin ammonium salt **26** as the linker-drug.

a)

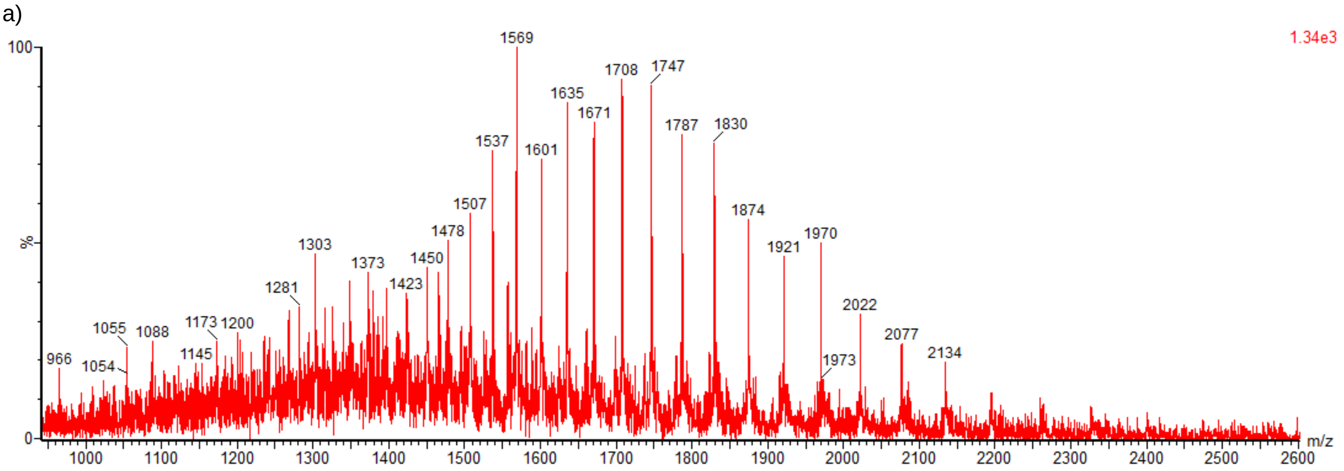

b)

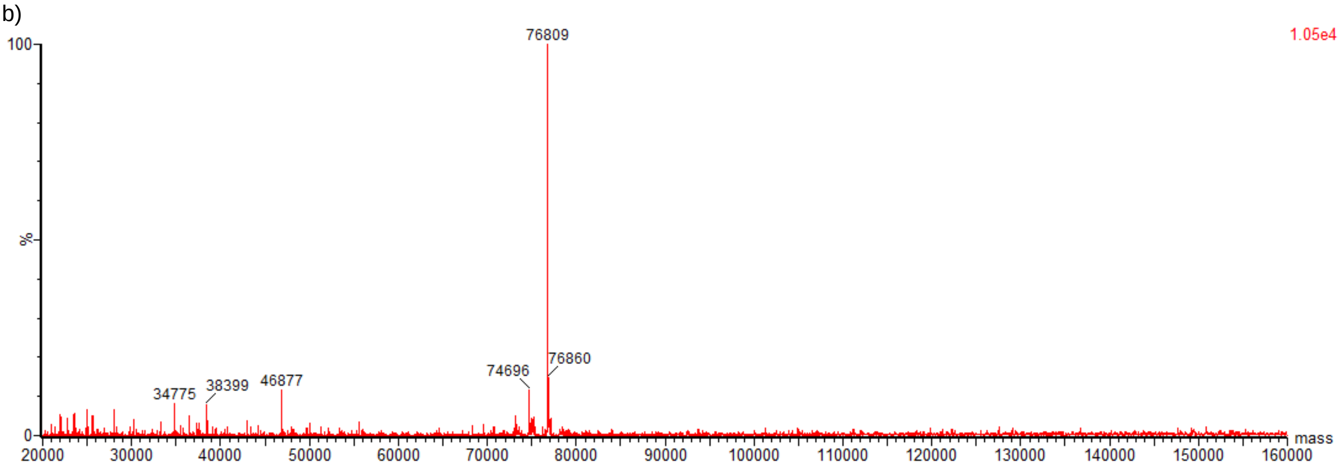

Figure S1 LCMS analysis of **ADC 1** a) non-deconvoluted MS b) deconvoluted MS, expected 76806 Da, found 76809 Da

ADC 2

ADC 2 was synthesised by using DVP-PEG$_2$-Glu$_3$-PEG$_2$-triazole-PEG$_4$-Val-Ala-PABC-taltobulin ammonium salt **27** as the linker-drug.

a)

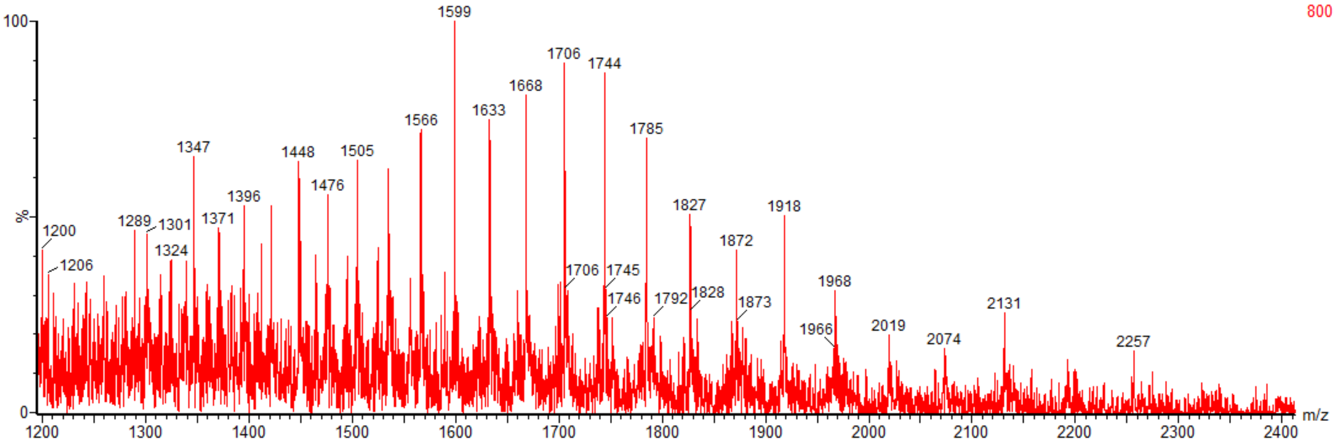

b)

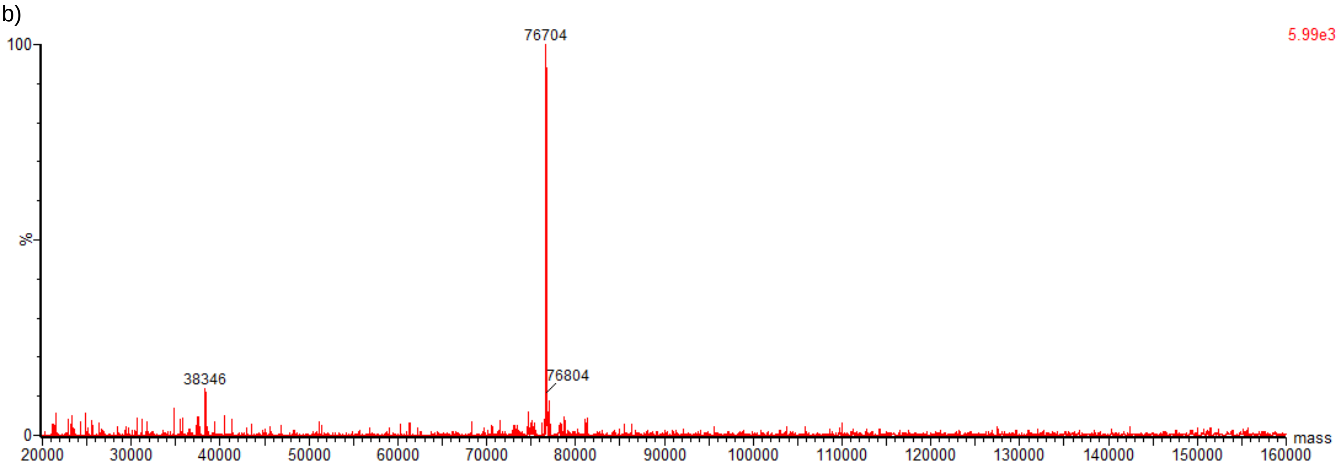

Figure S2 LCMS analysis of **ADC 2** a) non-deconvoluted MS b) deconvoluted MS, expected 76700 Da, found 76704 Da

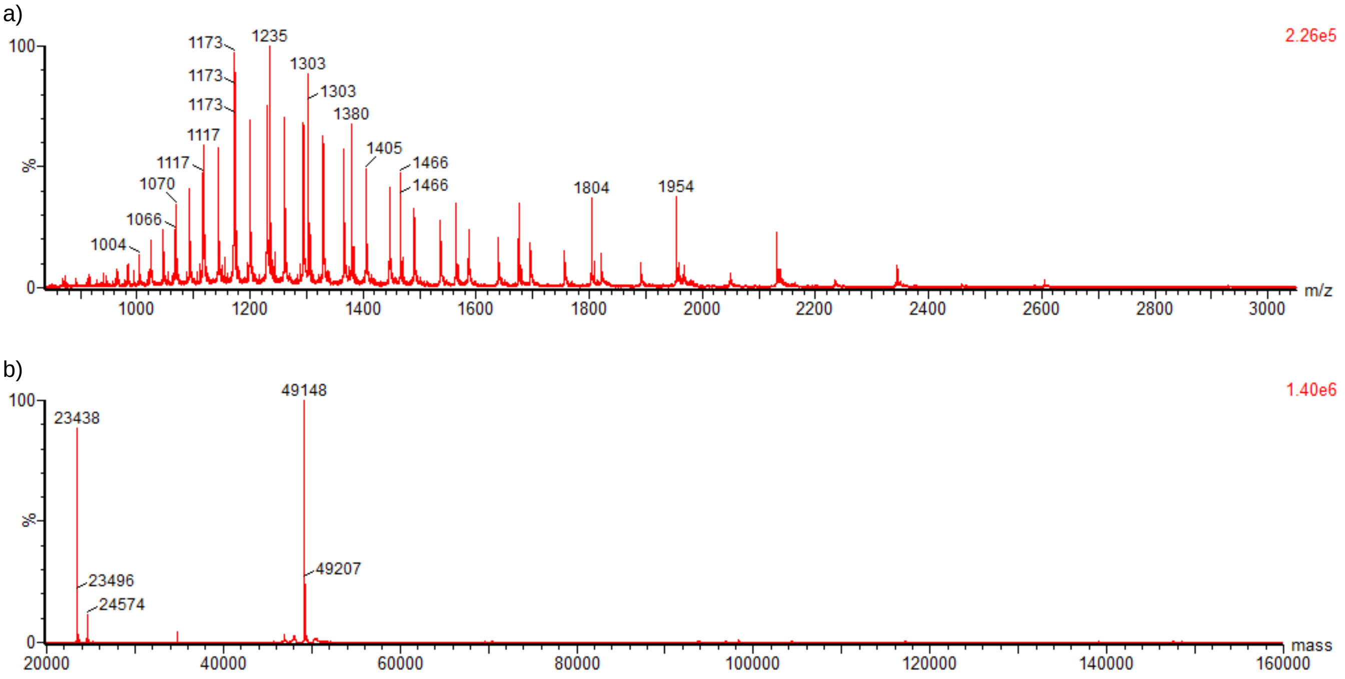

Figure S3 LCMS analysis of reduced trastuzumab a) non-deconvoluted MS b) deconvoluted MS, expected light chain 23439 Da, found 23438 Da; expected heavy chain 49149 Da, found 49148 Da.

Size Exclusion Chromatography (SEC)

SEC was performed on an ÄKTA Pure chromatography system using a Superdex 200 10/300 gel filtration column equilibrated with PBS. The protein was eluted using PBS and the UV trace measured at 280 nm.

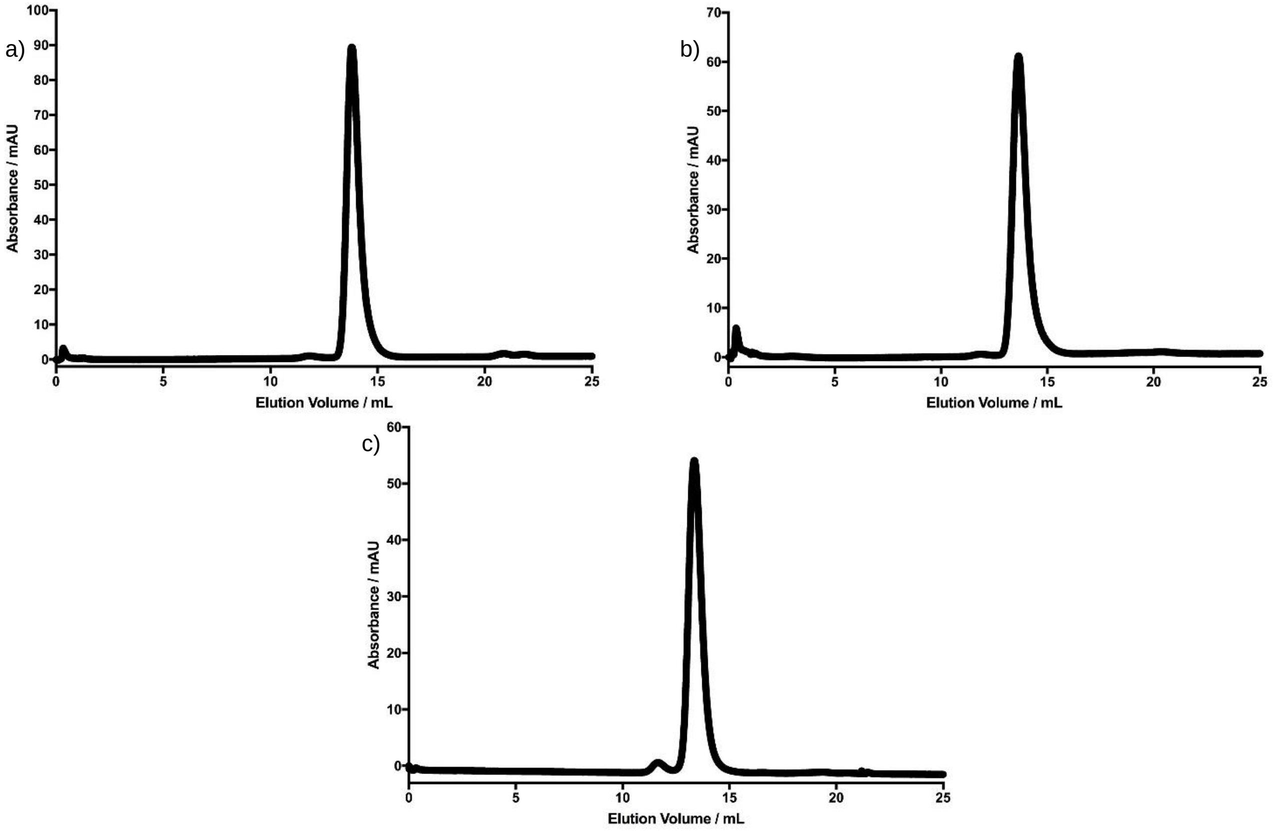

Figure S4 Chromatograms from SEC analysis of a) trastuzumab b) **ADC 1** c) **ADC 2**

Table S3 Monomeric content of trastuzumab, **ADC 1**, and **ADC 2**

ADC	% monomeric content
Trastuzumab	99.9
**ADC 1**	99.9
**ADC 2**	99.5

Cells Lines

HER2-positive SKBR3 and BT474 cells were obtained from the American Type Culture Collection (ATCC) and HER2-negative MCF7 cells were obtained from the European Collection of Authenticated Cell Cultures (ECACC). SKBR3 cells were maintained in high glucose McCoy’s 5A medium, supplemented with 10% heat-inactivated foetal-bovine serum (FBS), 50 U/mL penicillin and 50 µg/mL streptomycin. MCF7 cells were maintained in Dulbecco’s Modified Eagle Medium (DMEM) supplemented with 10% heat-inactivated fetal-bovine serum (FBS), 2 mM L- glutamine, 50 U/mL penicillin and 50 µg/mL streptomycin. BT474 cells were maintained in RPMI-1640 medium supplemented with 10% heat-inactivated fetal-bovine serum (FBS), 2 mM L-glutamine, 50 U/mL penicillin and 50 µg/mL streptomycin. All cell lines were incubated at 37 °C with 5% CO_2.

Cellular viabilty assays

Cells were seeded in 96-well plates for 24 h at 37 °C with 5% CO_2. SKBR3 cells were seeded at 20,000 cells/well, BT474 cells were seeded at 20,000 cells/well and MCF7 cells were seeded at 7,500 cells/well. Serial dilutions of **ADC 1**, **ADC 2**, **1**, **8** and trastuzumab were added to the cells in complete growth medium and incubated at 37 °C with 5% CO_2 for 96 h. Cell viability was measured using CellTiter-Glo viability assay (Promega) according to the manufacturer's instructions. Cell viability was plotted as a percentage of untreated cells. Each measurement was taken in triplicate and three independent repeats were performed.

SUPPORTING INFORMATION

Crystallographic Data

Methyl (*S*)-3,3-dimethyl-2-((*S*)-3-methyl-3-(1-methyl-1*H*-indol-3-yl)-2-(2,2,2-trifluoro-*N*-methylacetamido)butanamido)butanoate (9a)

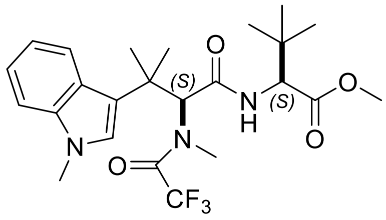

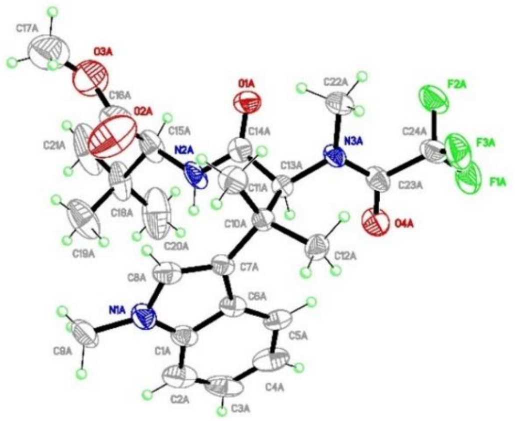

CCDC number	2012415
**Crystal data**	
**Chemical formula**	$C_{24}H_{32}F_3N_3O_4$
***M*r**	483.52
**Crystal system, space group**	Monoclinic, $P2_1$
**Temperature (K)**	180
***a*, *b*, *c* (Å)**	18.9031 (7), 12.3366 (4), 21.0506 (7)
**β (°)**	90.002 (2)
***V* (Å³)**	4909.0 (3)
Z	8
***F*(000)**	2048
**D_x (Mg m^{-3})**	1.308
**Radiation type**	Cu *K*α
**No. of reflections for cell measurement**	9656
**θ range (°) for cell measurement**	4.2–66.6
**μ (mm^{-1})**	0.88
**Crystal shape**	Lath
**Crystal size (mm³)**	0.18 × 0.10 × 0.06
**Data collection**	
**Diffractometer**	Bruker D8-QUEST PHOTON-100
**Scan method**	ω and ϕ–scans
**Absorption correction**	Multi-scan<br>*SADABS* (Bruker, 2014)
**T_{min}, T_{max}**	0.661, 0.753
**No. of measured, independent and observed [*I* > 2σ(*I*)] reflections**	51831, 16567, 13315
**R_{int}**	0.070
**θ values (°)**	θ_{max} = 67.2, θ_{min} = 2.3
**$(\sin \theta/\lambda)_{max}$ (Å^{-1})**	0.598
**Refinement**	
**$R[F^2 > 2\sigma(F^2)]$, $wR(F^2)$, *S***	0.055, 0.126, 1.05
**No. of reflections**	16567
**No. of parameters**	1258
**No. of restraints**	541
**H-atom treatment**	H-atom parameters constrained
**$\Delta\rho_{max}$, $\Delta\rho_{min}$ (e Å^{-3})**	0.26, -0.29
**Absolute structure**	Flack x determined using 4819 quotients [(I+)-(I-)]/[(I+)+(I-)] (Parsons, Flack and Wagner, Acta Cryst. B69 (2013) 249-259).
**Absolute structure parameter**	-0.03 (9)

SUPPORTING INFORMATION

Methyl (*S*)-3,3-dimethyl-2-((*R*)-3-methyl-3-(1-methyl-1*H*-indol-3-yl)-2-(2,2,2-trifluoro-*N*-methylacetamido)butanamido)butanoate (9b)

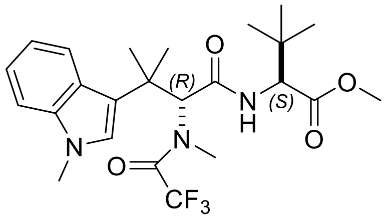

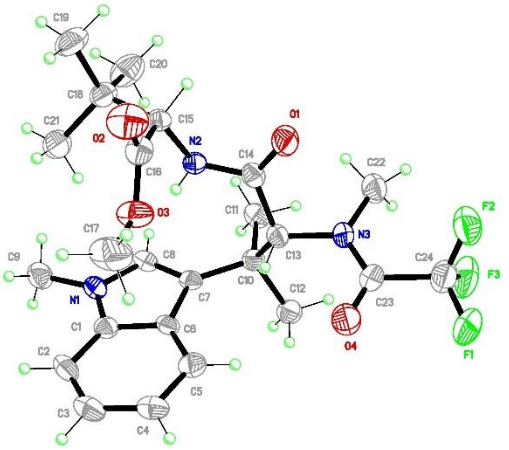

**CCDC**	**2012414**
**Crystal data**	
**Chemical formula**	$C_{24}H_{32}F_3N_3O_4$
***M*r**	483.52
**Crystal system, space group**	Orthorhombic, $P2_12_12_1$
**Temperature (K)**	180
***a*, *b*, *c* (Å)**	10.2753 (4), 13.3147 (4), 17.9428 (5)
***V* (Å³)**	2454.80 (14)
Z	4
***F*(000)**	1024
**D_x (Mg m^{-3})**	1.308
**Radiation type**	Cu *K*α
**No. of reflections for cell measurement**	4770
**θ range (°) for cell measurement**	4.1–66.6
**μ (mm^{-1})**	0.88
**Crystal shape**	Lath
**Crystal size (mm³)**	0.16 × 0.10 × 0.04
**Data collection**	
**Diffractometer**	Bruker D8-QUEST PHOTON-100
**Scan method**	ω and ϕ–scans
**Absorption correction**	Multi-scan<br>*SADABS* (Bruker, 2014)
**T_{min}, T_{max}**	0.653, 0.753
**No. of measured, independent and observed [*I* > 2σ(*I*)] reflections**	12912, 4214, 3378
**R_{int}**	0.055
**θ values (°)**	θ_{max} = 66.7, θ_{min} = 4.1
**(sin θ/λ)$_{max}$ (Å^{-1})**	0.596
**Refinement**	
**$R[F^2 > 2\sigma(F^2)]$, $wR(F^2)$, *S***	0.045, 0.109, 1.03
**No. of reflections**	4214
**No. of parameters**	319
**H-atom treatment**	H atoms treated by a mixture of independent and constrained refinement
**$\Delta\rho_{max}$, $\Delta\rho_{min}$ (e Å^{-3})**	0.17, -0.17
**Absolute structure**	Flack x determined using 1196 quotients [(I+)-(I-)]/[(I+)+(I-)] (Parsons, Flack and Wagner, Acta Cryst. B69 (2013) 249-259).
**Absolute structure parameter**	0.04 (18)

***N*-trifluoroacetyl hemiasterlin ethyl ester (15a)**

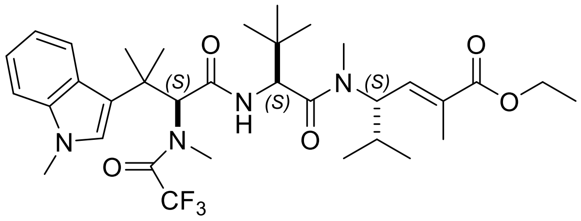

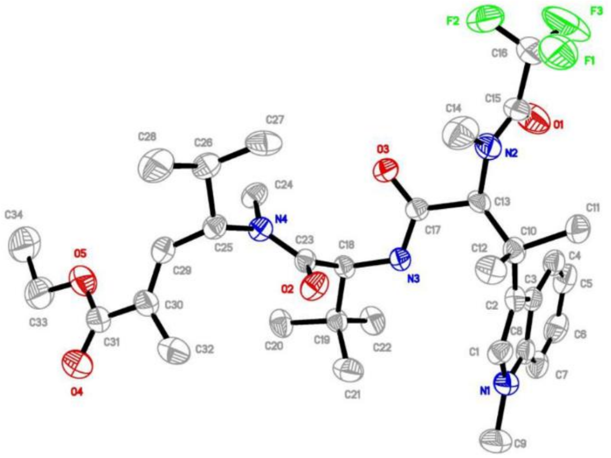

**CCDC**	**2012776**
**Crystal data**	
**Chemical formula**	$C_{34}H_{49}F_3N_4O_5$
***M*r**	650.77
**Crystal system, space group**	Monoclinic, $P2_1$
**Temperature (K)**	180
***a*, *b*, *c* (Å)**	16.2983 (9), 6.1314 (4), 17.8581 (10)
**b (°)**	96.979 (3)
***V* (Å^3)**	1771.36 (18)
Z	2
***F*(000)**	696
**D_x (Mg m^{-3})**	1.220
**Radiation type**	Cu *K*α
**No. of reflections for cell measurement**	4141
**θ range (°) for cell measurement**	2.7–62.0
**μ (mm^{-1})**	0.77
**Crystal shape**	Needle
**Crystal size (mm^3)**	0.45 × 0.03 × 0.03
**Data collection**	
**Diffractometer**	Bruker D8-QUEST PHOTON-100
**Scan method**	ω and ϕ–scans
**Absorption correction**	Multi-scan *SADABS* (Bruker, 2014)
**T_{min}, T_{max}**	0.608, 0.753
**No. of measured, independent and observed [*I* > 2s(*I*)] reflections**	24424, 6173, 3784
**R_{int}**	0.146
**θ values (°)**	θ_{max} = 66.7, θ_{min} = 2.5
**$(\sin \theta/\lambda)_{max}$ (Å^{-1})**	0.596
**Refinement**	
**$R[F^2 > 2\sigma(F^2)]$, $wR(F^2)$, *S***	0.073, 0.186, 1.01
**No. of reflections**	6173
**No. of parameters**	428
**No. of restraints**	1
**H-atom treatment**	H-atom parameters constrained
**$\Delta\rho_{max}$, $\Delta\rho_{min}$ (e Å^{-3})**	0.20, -0.21
**Absolute structure**	Classical Flack method preferred over Parsons because s.u. lower.
**Absolute structure parameter**	0.0 (5)

Supplementary Figures and Tables

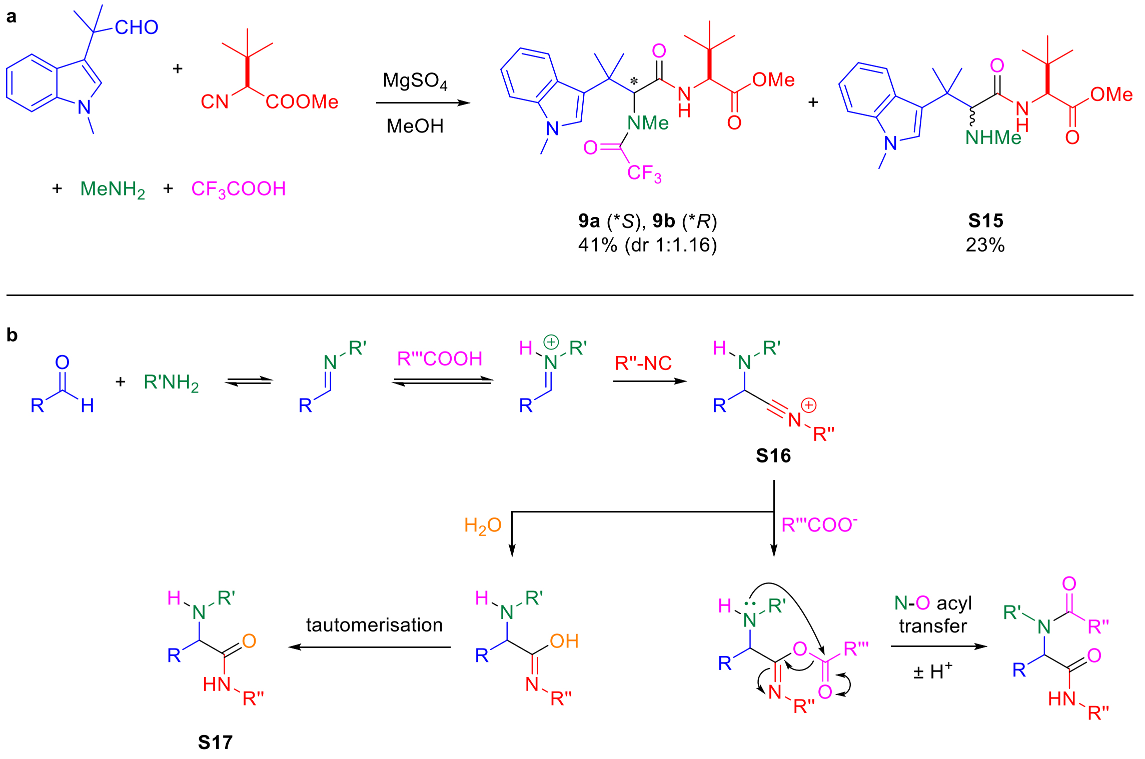

Scheme S1 a) Using anhydrous $MgSO_4$ as a drying reagent resulted in the formation of free amine **S15** side-product. b) The proposed mechanism of Ugi reaction leading to the undesired product **S17**, if H_2O, instead of carboxylate anion, attacks the nitrilium intermediate **S16**.

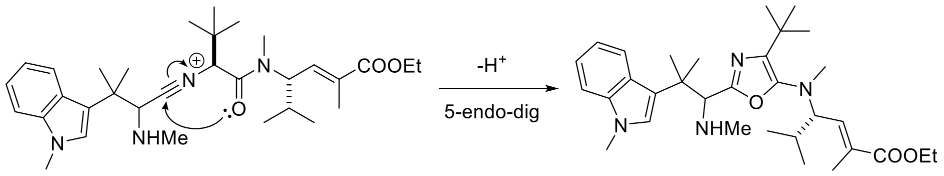

Scheme S2 Proposed cyclisation of the nitrilium intermediate to form a putative undesired oxazole side-product.

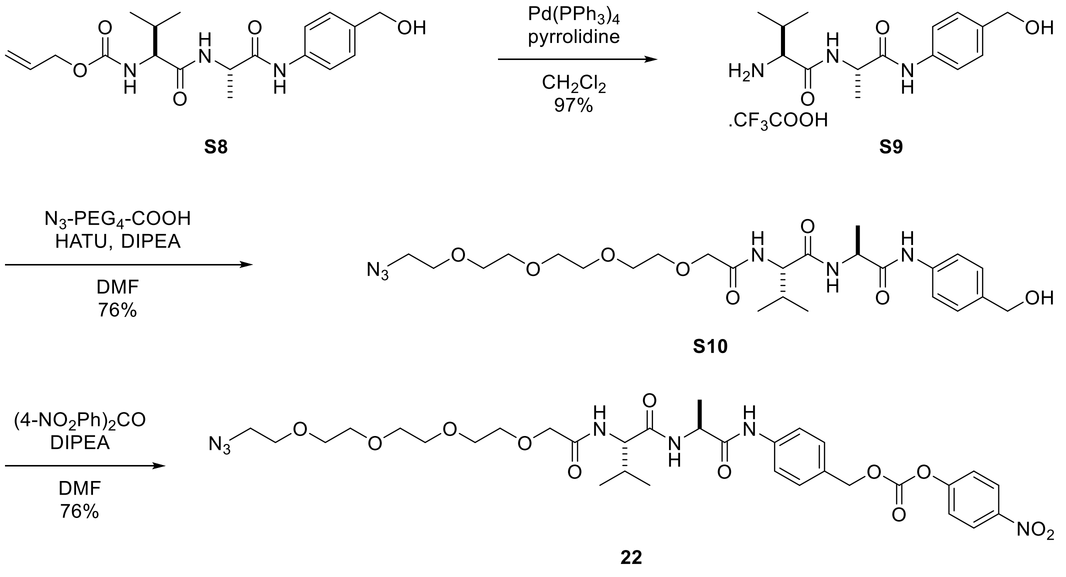

Scheme S3 Synthesis of activated carbonate **22**

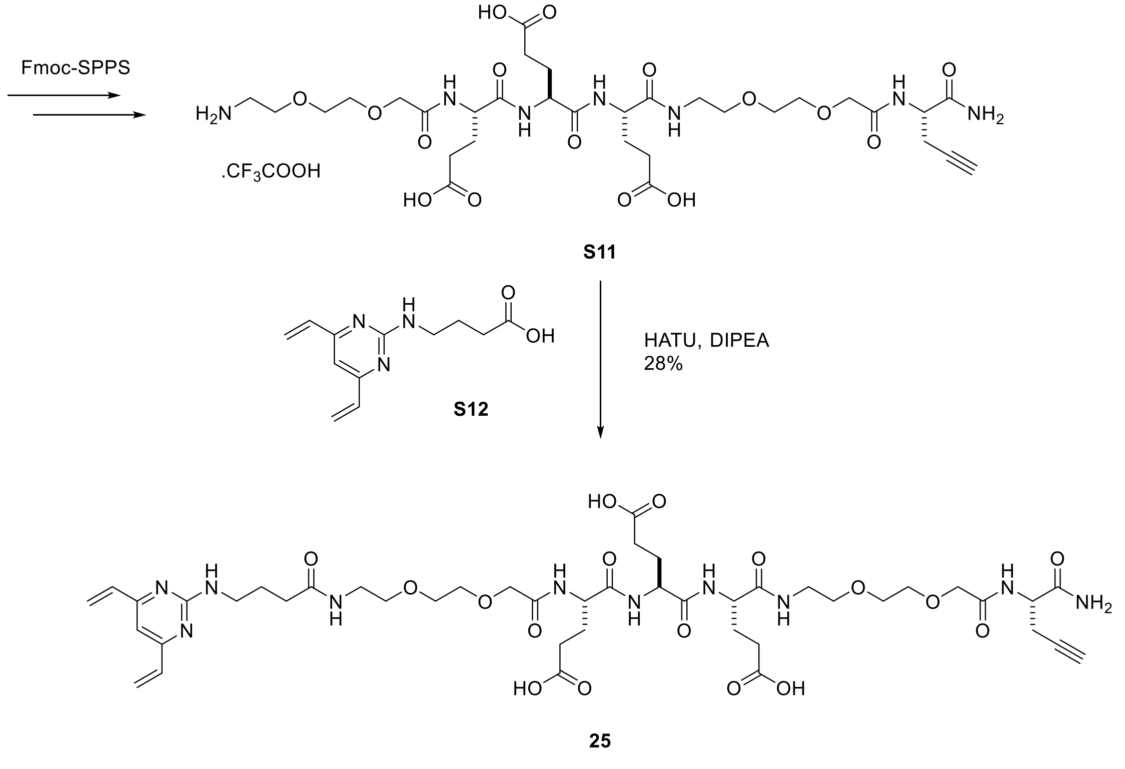

Scheme S4 Synthesis of DVP-alkyne **25**

Table S4 Optimisation for the second attempt at Ugi-4CR.

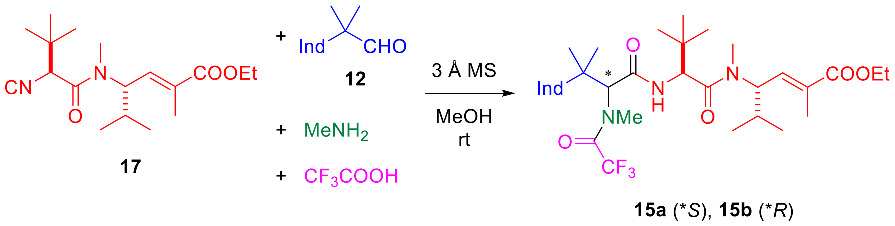

Entry	TFA eq.	Additive	Isolated yield (*dr* = 15a:15b)*	Remarks
1	1.2	-	20% (*dr* 1:1.3)	Oxazole side product observed
2	2.5	-	-	Complex mixture
3	1.2	CF_3COONa (1.2 eq.)	73% (*dr* 1:1.4)	

*Diastereomeric ratios were determined from ^{19}F-NMR of the crude product

Table S5 *In vitro* cellular evaluation of **1**, **8**, **ADC 1** and **ADC 2** in comparison with MMAE and Tras-MMAE.

IC_{50} (nM)†	SKBR3 (HER2+)	BT474 (HER2+)	MCF7 (HER2-)
Hemiasterlin (**1**)	0.18	0.15	0.37
Taltobulin (**8**)	1.12	1.40	3.00
MMAE*	0.08	0.12	0.2
**ADC 1**	0.086	0.27	>50
**ADC 2**	0.25	0.45	>50
Tras-MMAE*	0.041	0.092	>30

†Each data point is an average of independent triplicates. *Data from Walsh *et al.*[13] and Bargh *et al.*[7] MMAE = monomethyl auristatin E.

NMR Spectra

Methyl (*S*)-2-formamido-3,3-dimethylbutanoate (13)

^{1}H-NMR

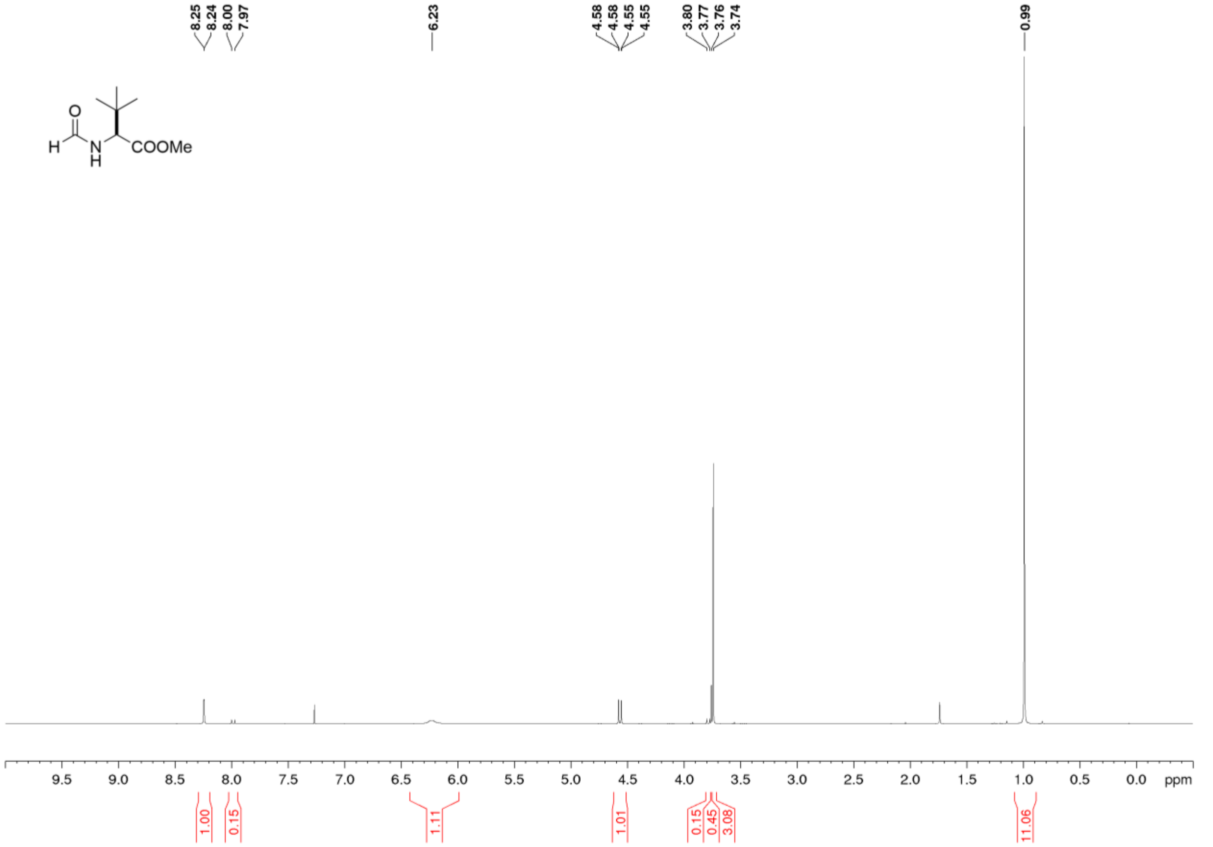

^{13}C-NMR

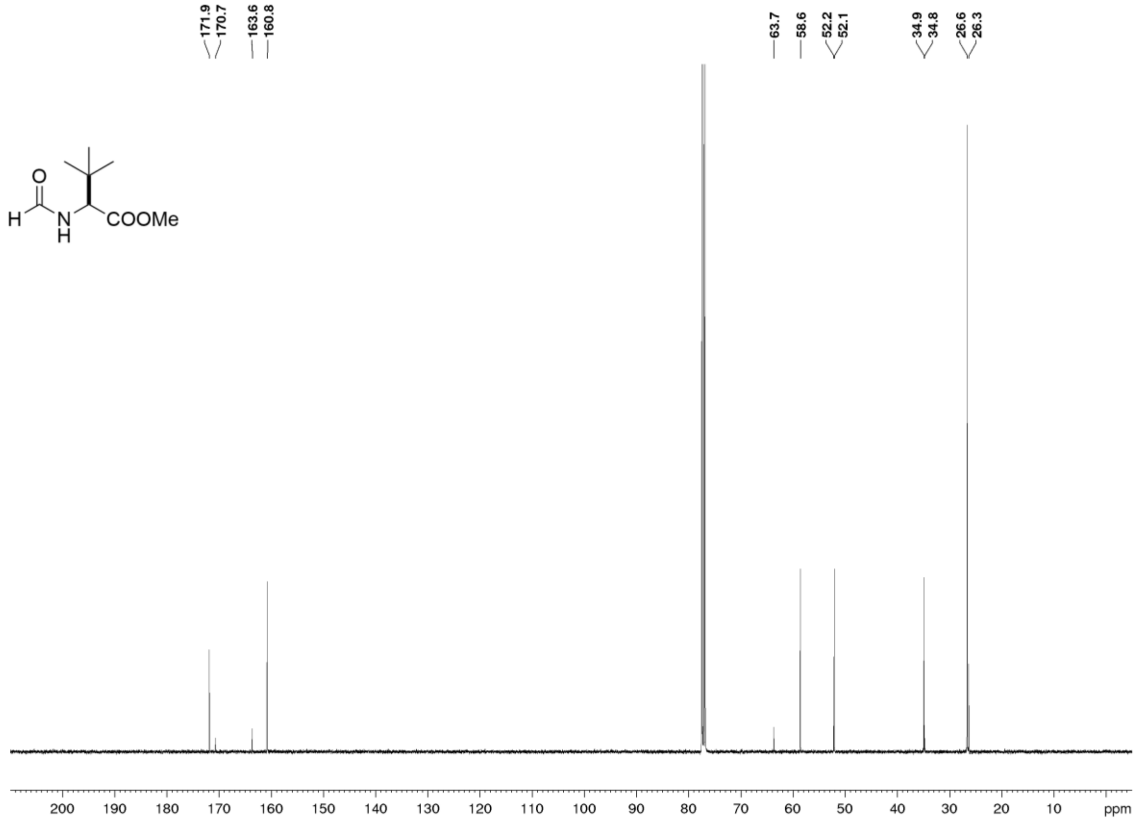

Methyl (*S*)-2-isocyano-3,3-dimethylbutanoate (11)

^{1}H-NMR

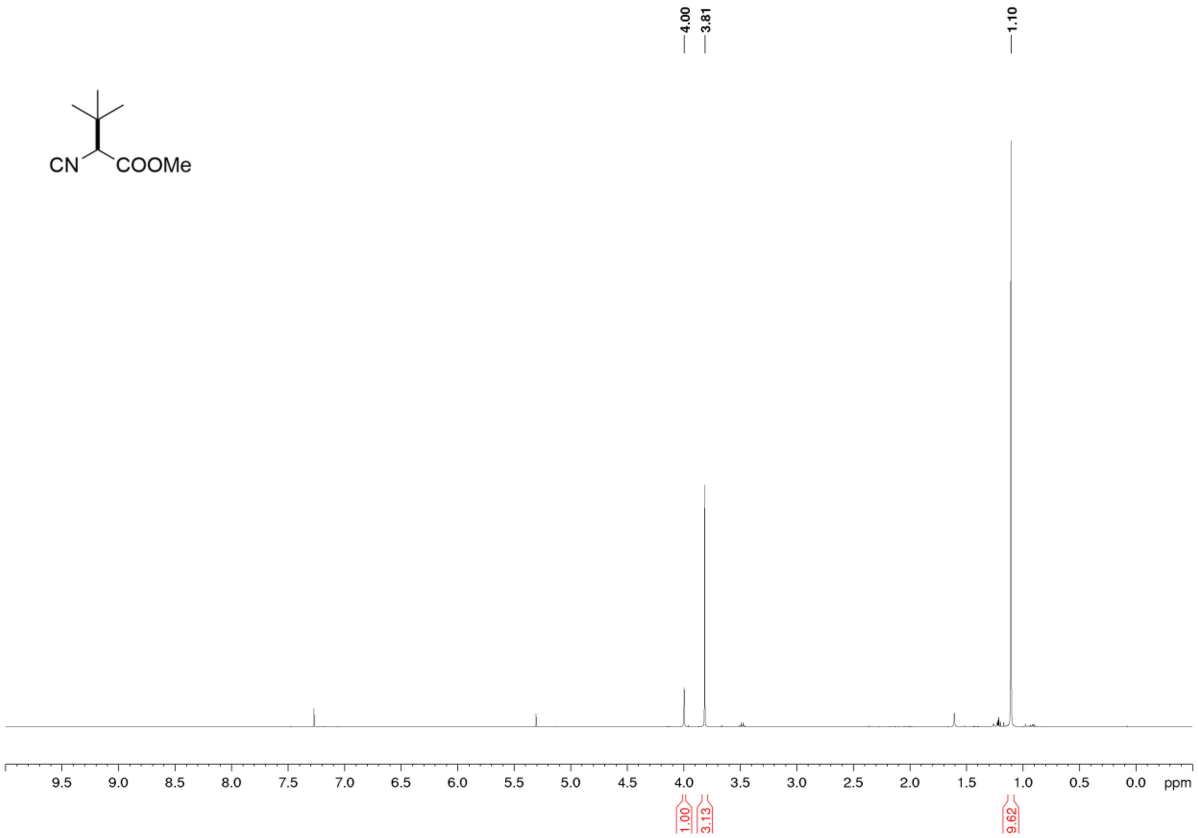

^{13}C-NMR

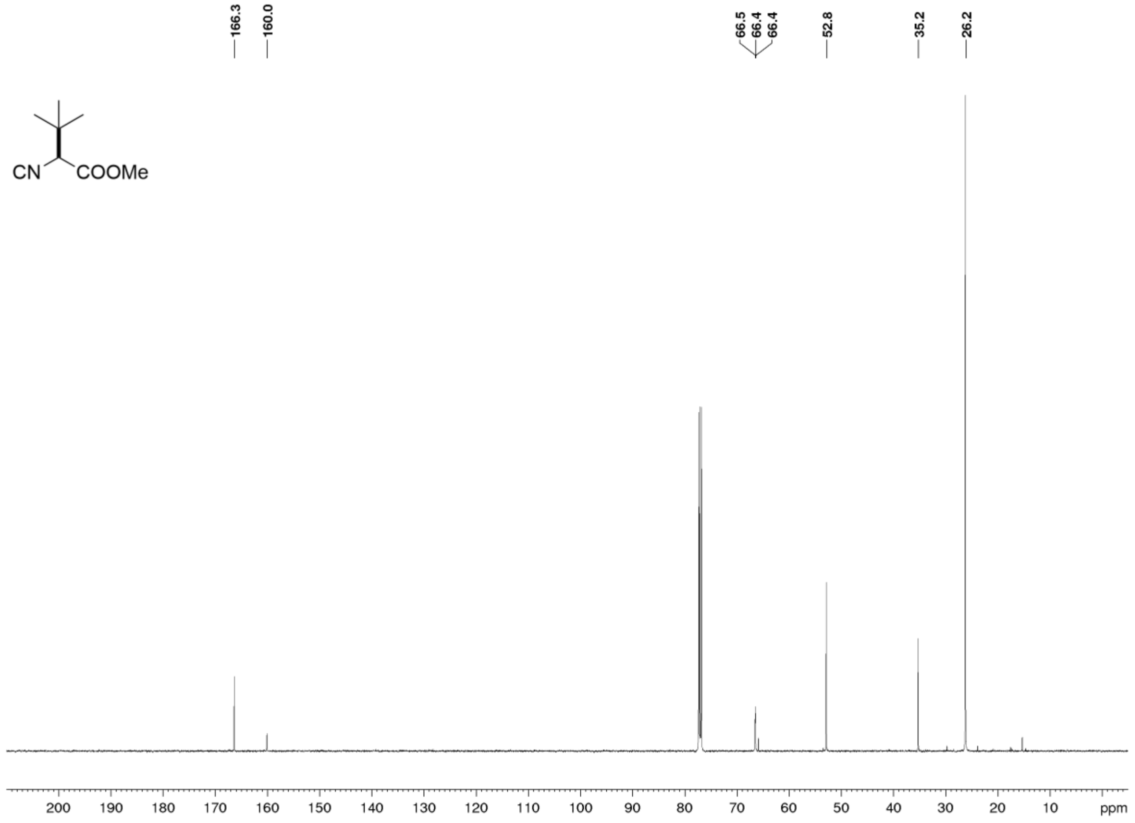

SUPPORTING INFORMATION

Methyl 2-(1-methyl-1*H*-indol-3-yl)propanoate (S1)

^{1}H-NMR

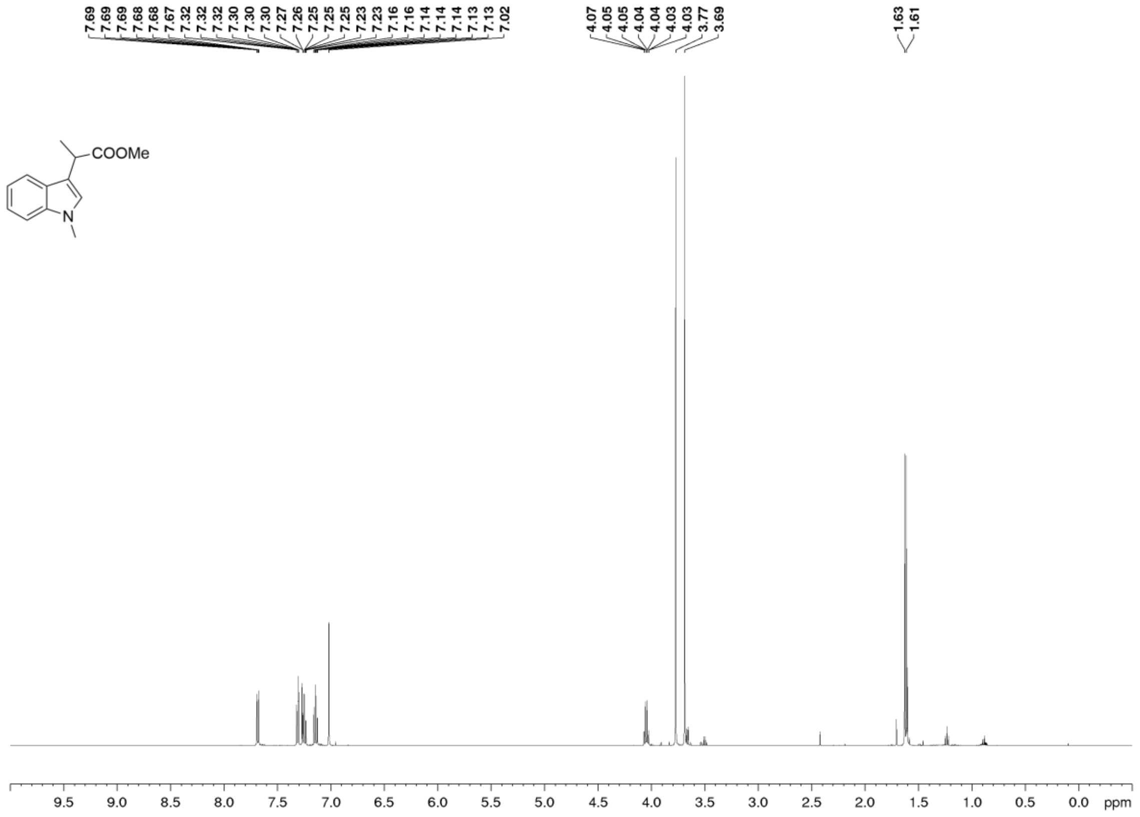

^{13}C-NMR

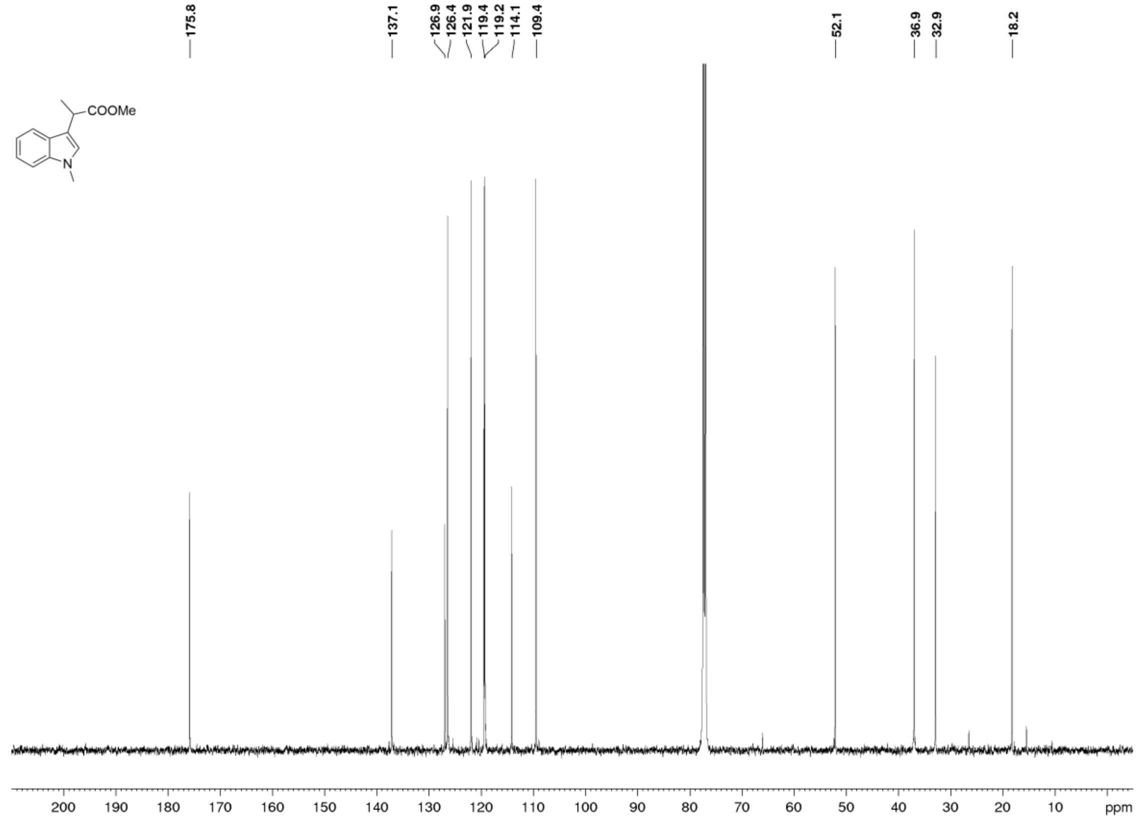

Methyl 2-methyl-2-(1-methyl-1*H*-indol-3-yl)propanoate (S3)

^{1}H-NMR

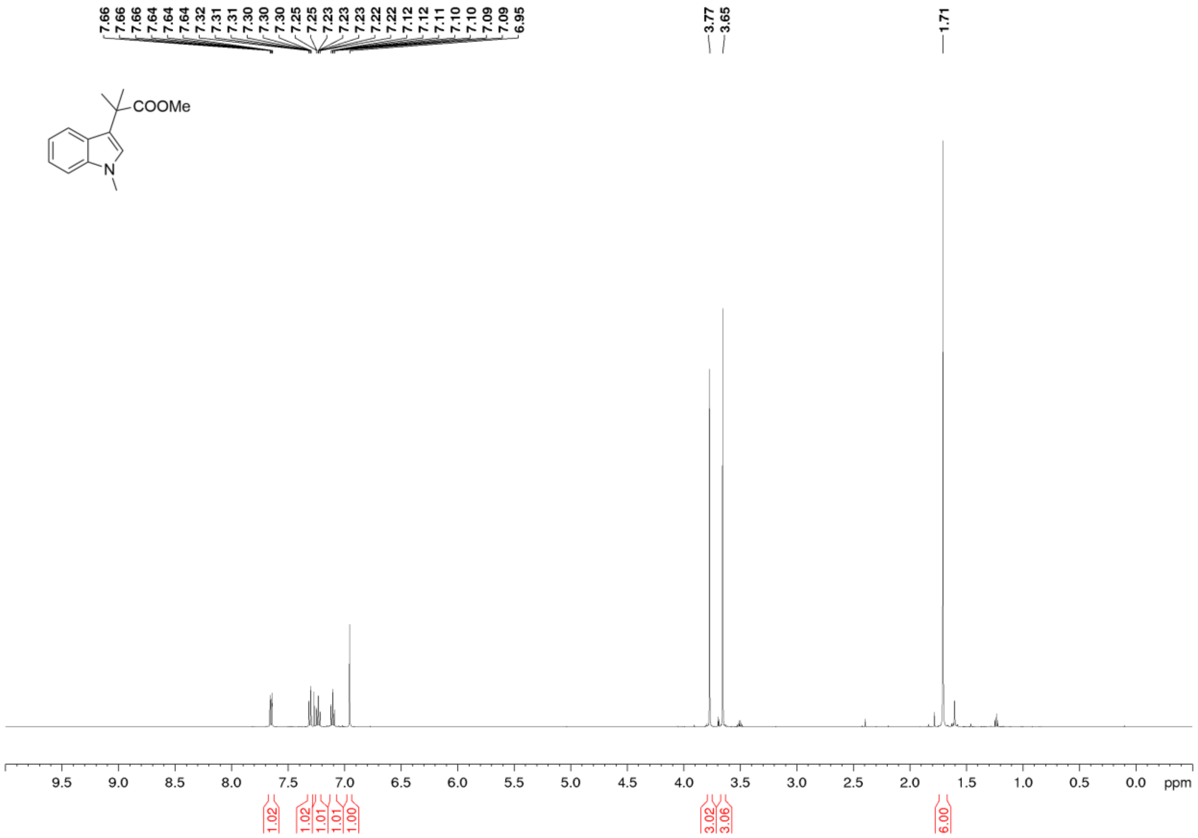

^{13}C-NMR

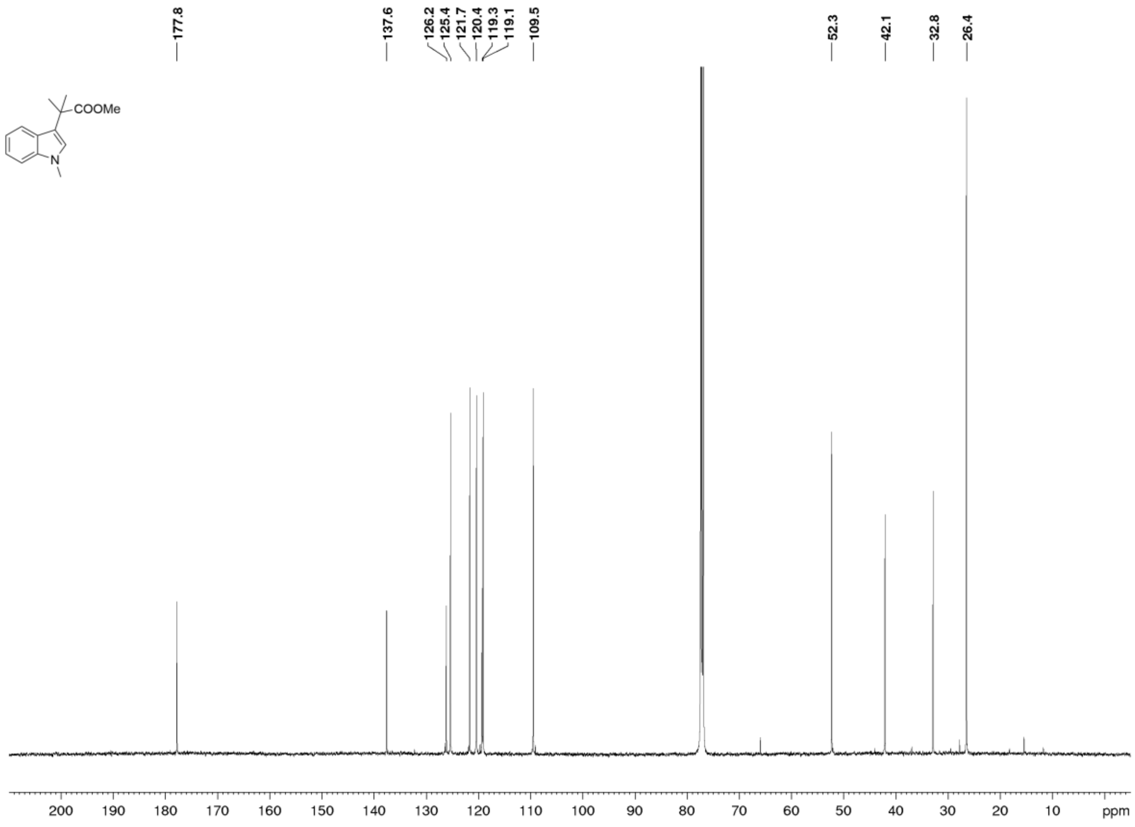

2-Methyl-2-(1-methyl-1*H*-indol-3-yl)propan-1-ol (S4)

^{1}H-NMR

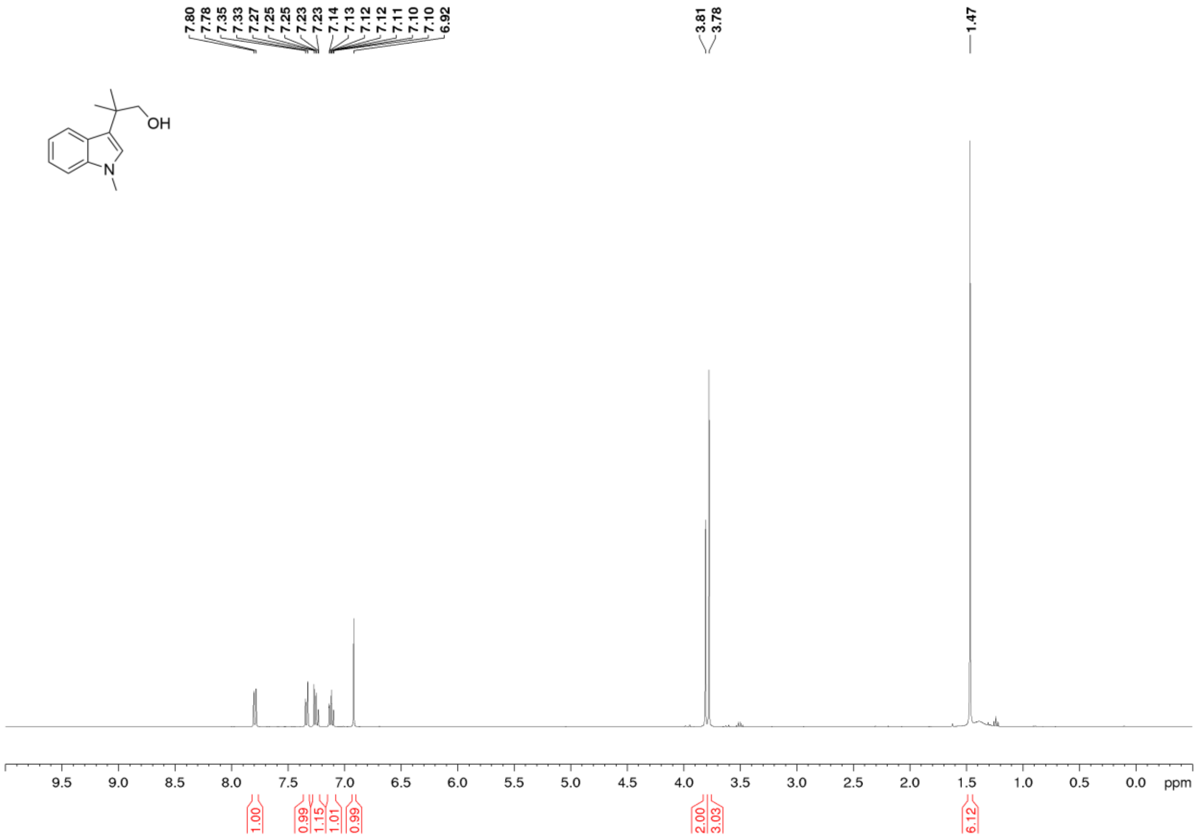

^{13}C-NMR

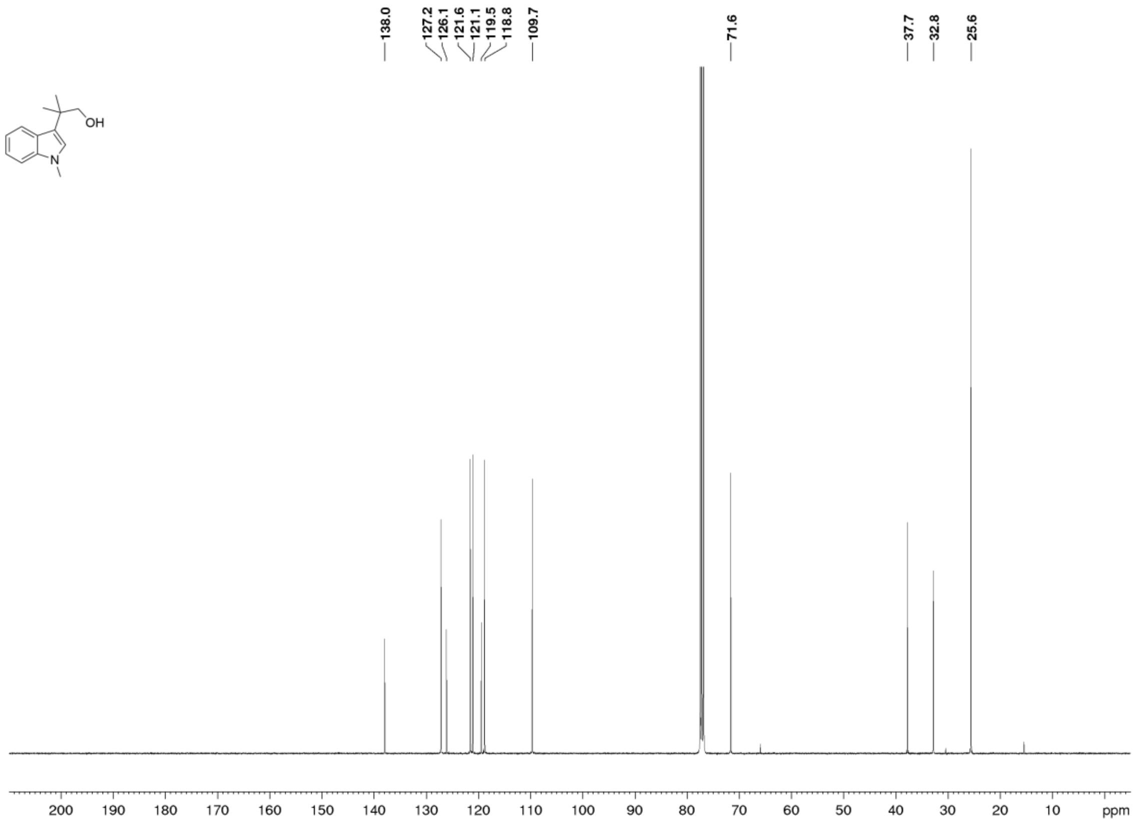

2-Methyl-2-(1-methyl-1*H*-indol-3-yl)propanal (12)

^{1}H-NMR

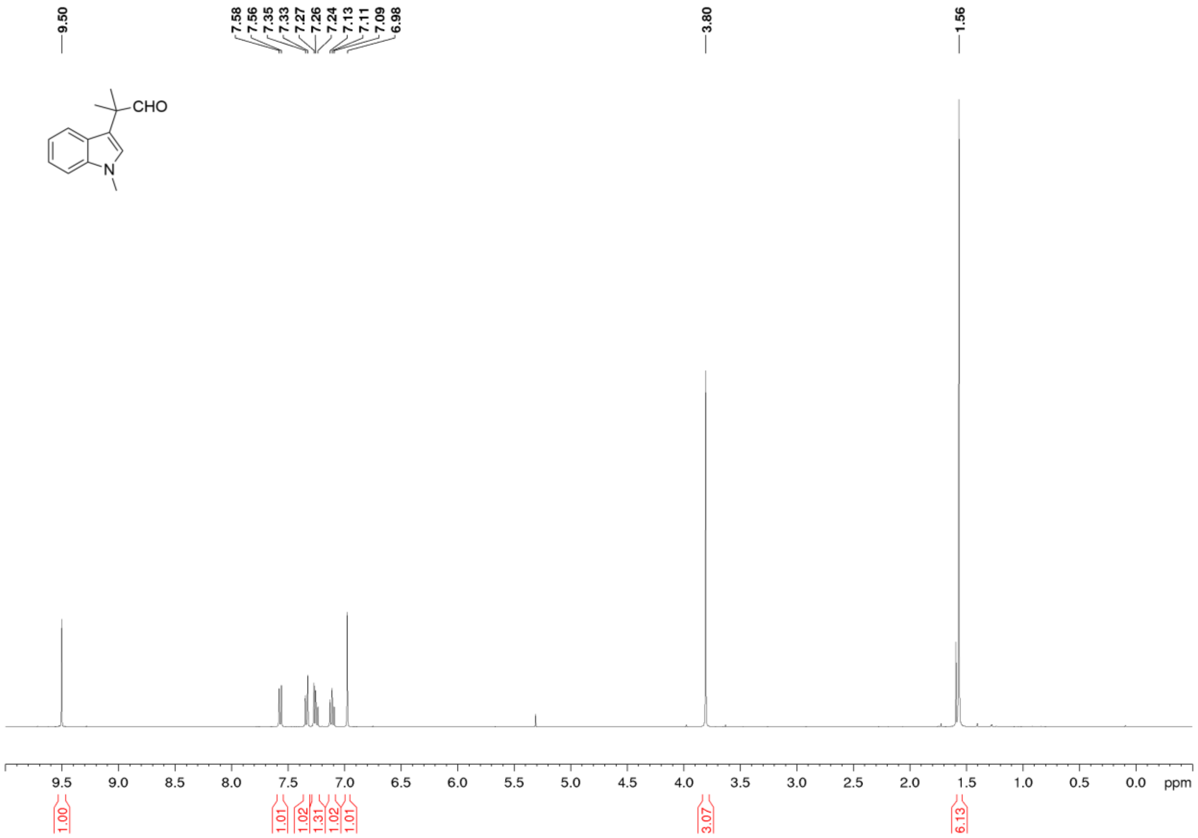

^{13}C-NMR

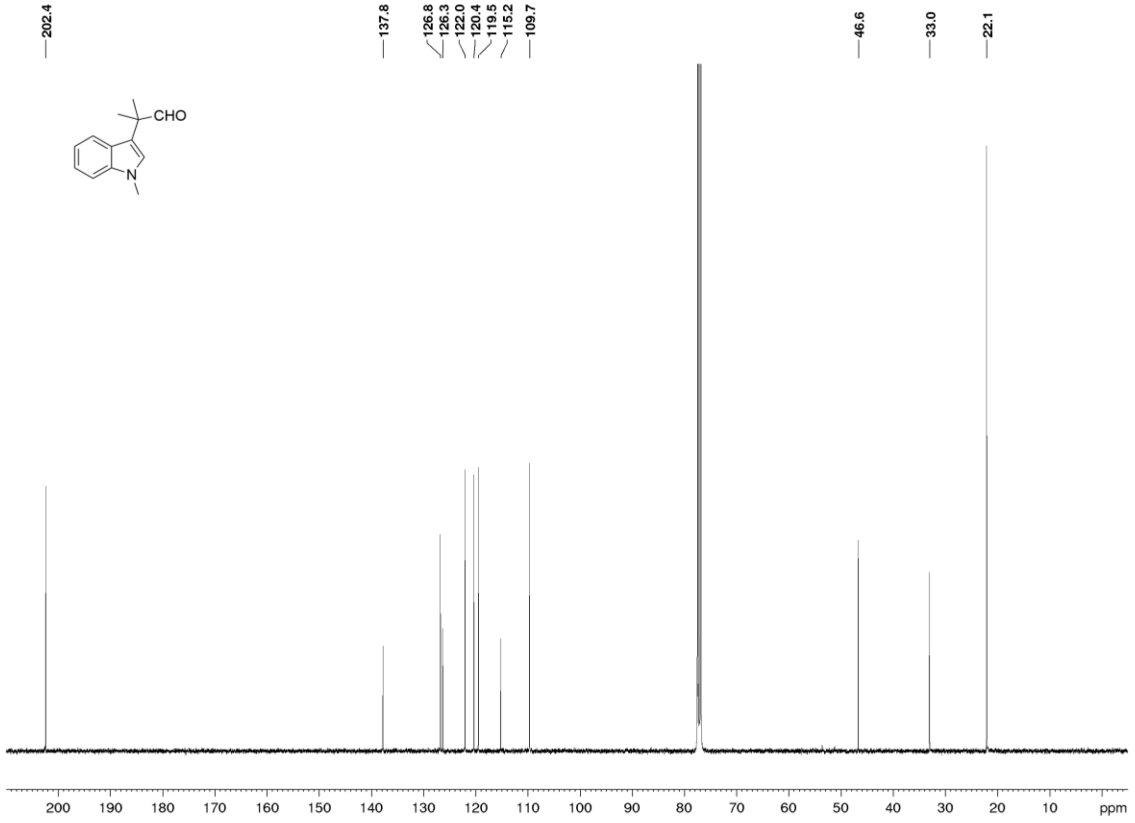

Methyl (*S*)-3,3-dimethyl-2-((*S*)-3-methyl-3-(1-methyl-1*H*-indol-3-yl)-2-(2,2,2-trifluoro-*N*-methylacetamido)butanamido)butanoate (9a)

^{1}H-NMR

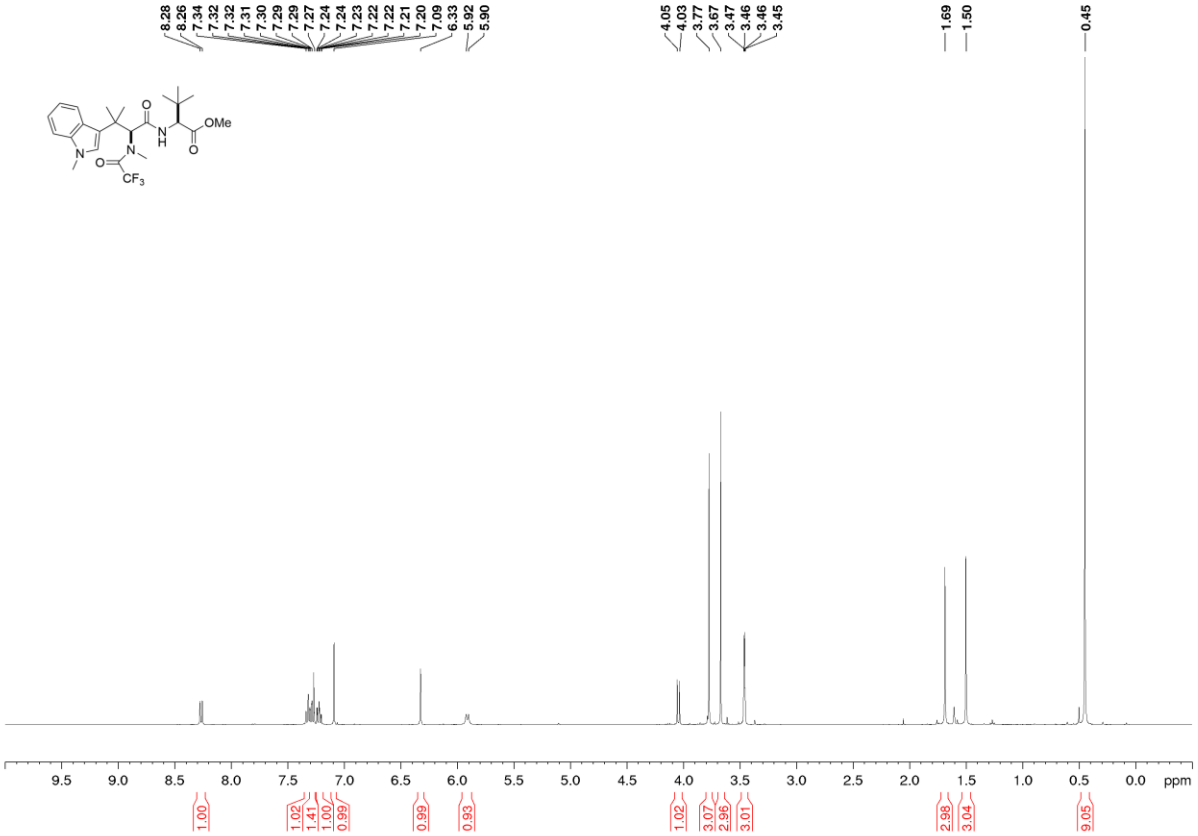

^{13}C-NMR

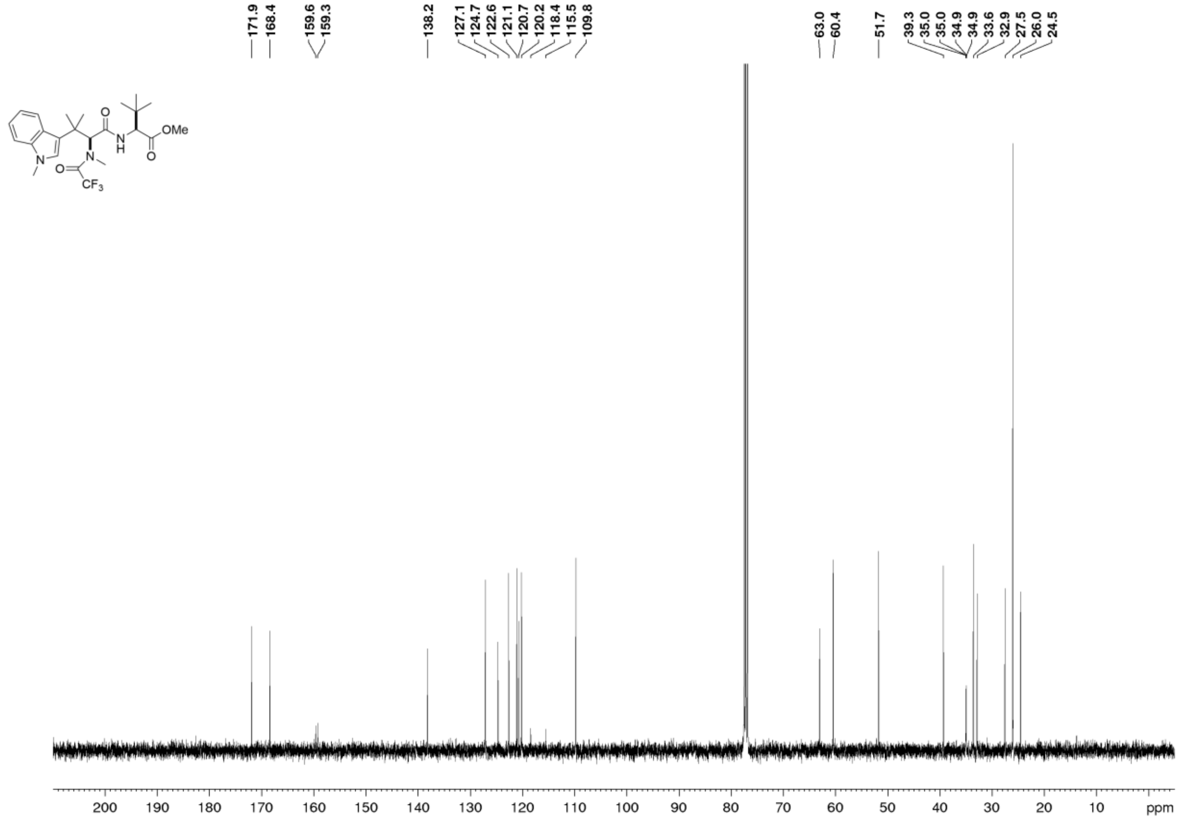

^{19}F-NMR

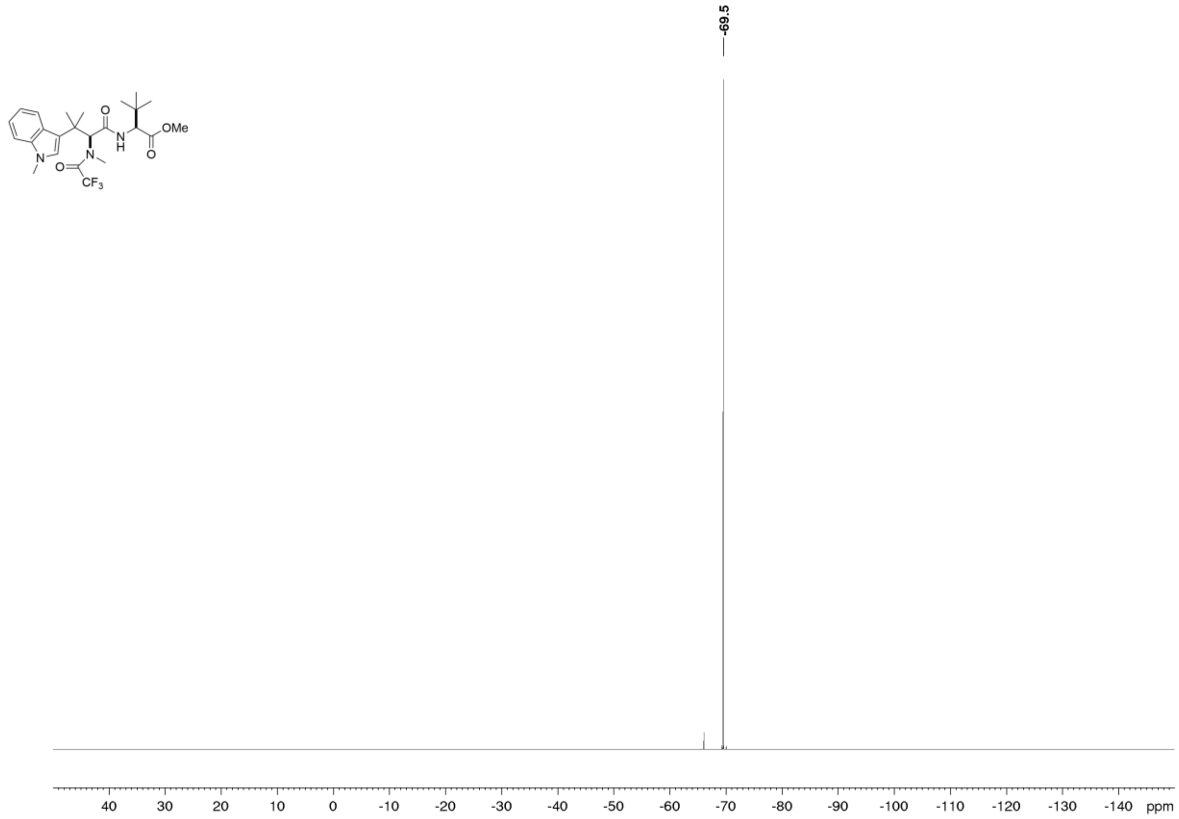
OMe
N
H
O
N
O
CF3
-69.5
40
30
20
10
0
-10
-20
-30
-40
-50
-60
-70
-80
-90
-100
-110
-120
-130
-140
ppm

Methyl (*S*)-3,3-dimethyl-2-((*R*)-3-methyl-3-(1-methyl-1*H*-indol-3-yl)-2-(2,2,2-trifluoro-*N*-methylacetamido)butanamido)butanoate (9b)

^{1}H-NMR

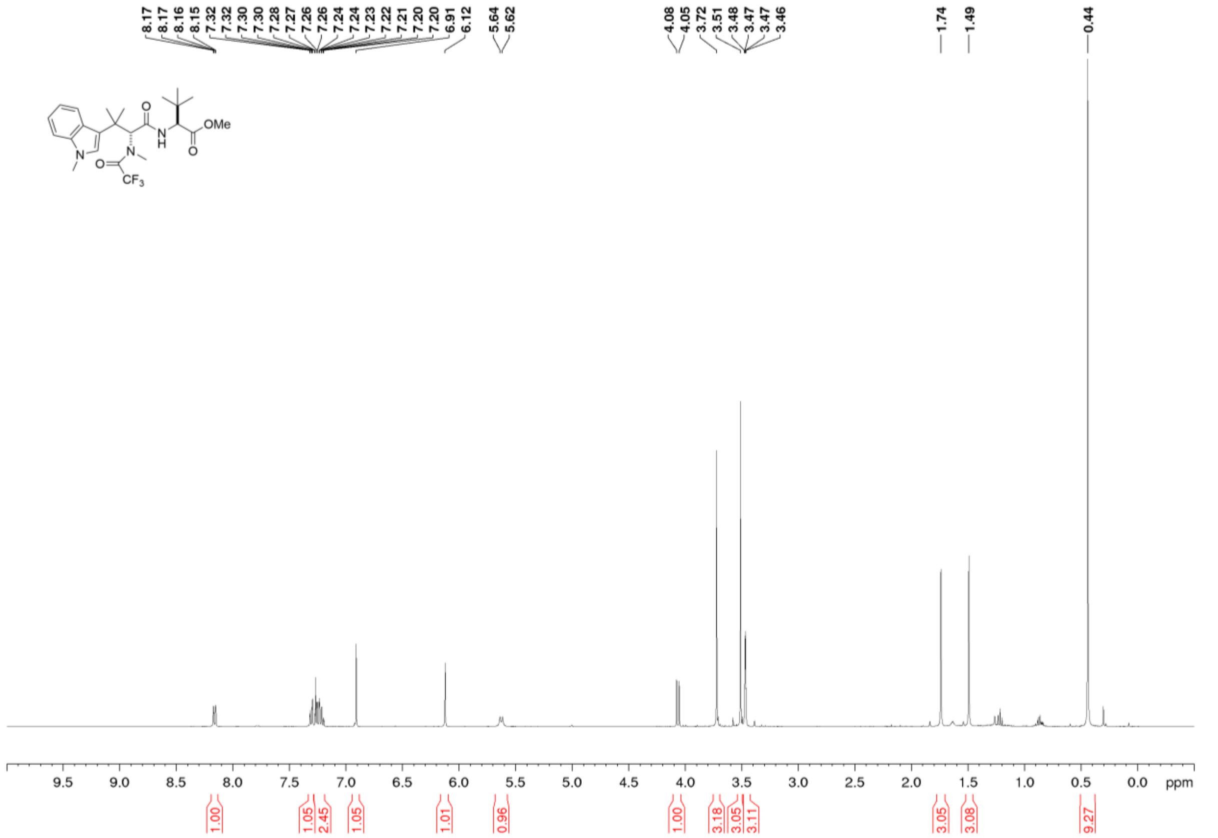

^{13}C-NMR

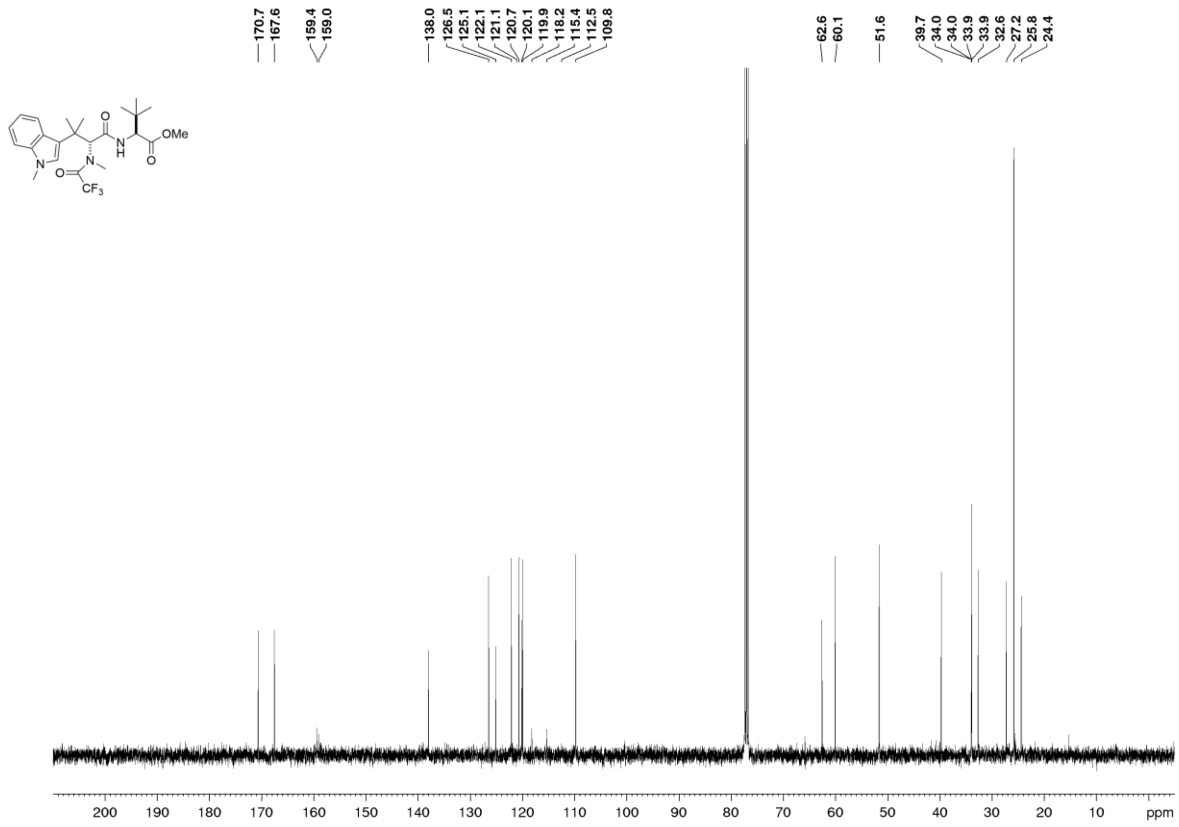

^{19}F-NMR

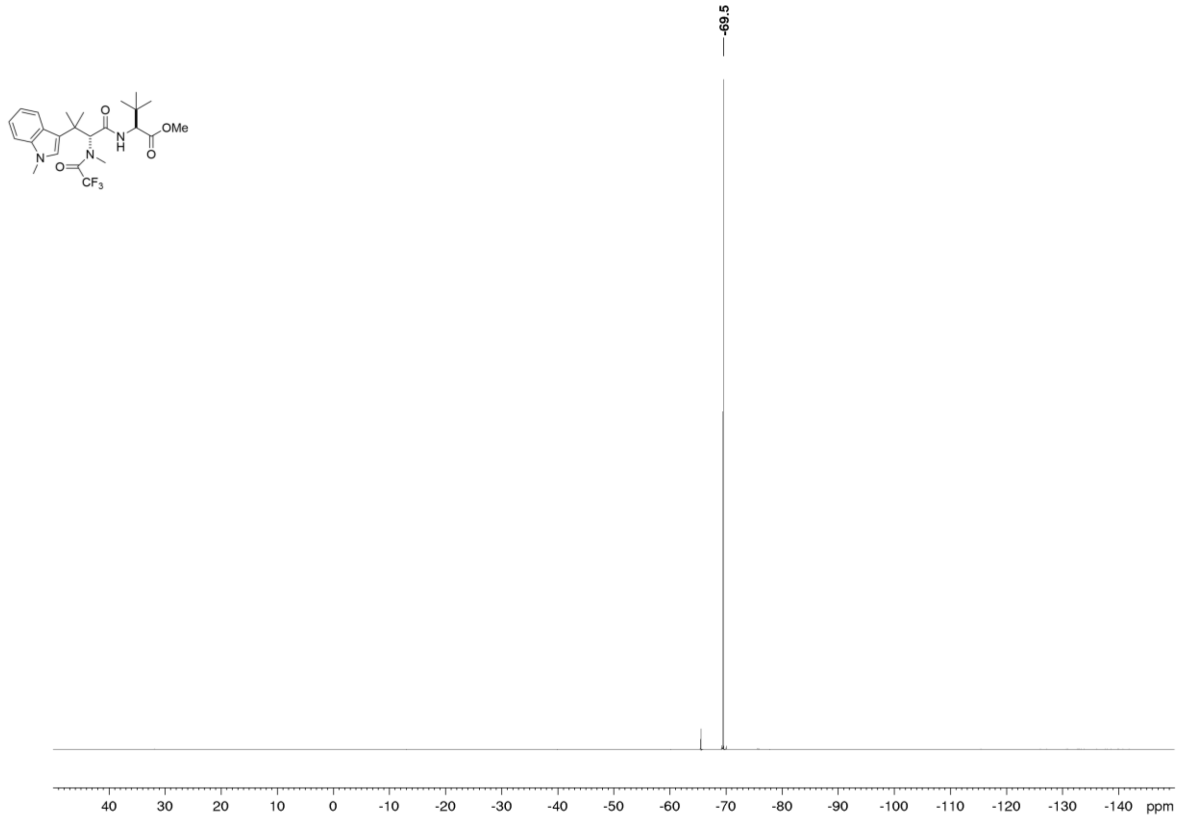
-69.5
OMe
N
H
O
N
O
CF3
ppm
40
30
20
10
0
-10
-20
-30
-40
-50
-60
-70
-80
-90
-100
-110
-120
-130
-140

(*S*)-*N*^α^-tert-butoxycarbonyl-*N*^α^-methylvalin-*N*-methoxy-*N*-methylamide (S5)

^{1}H-NMR

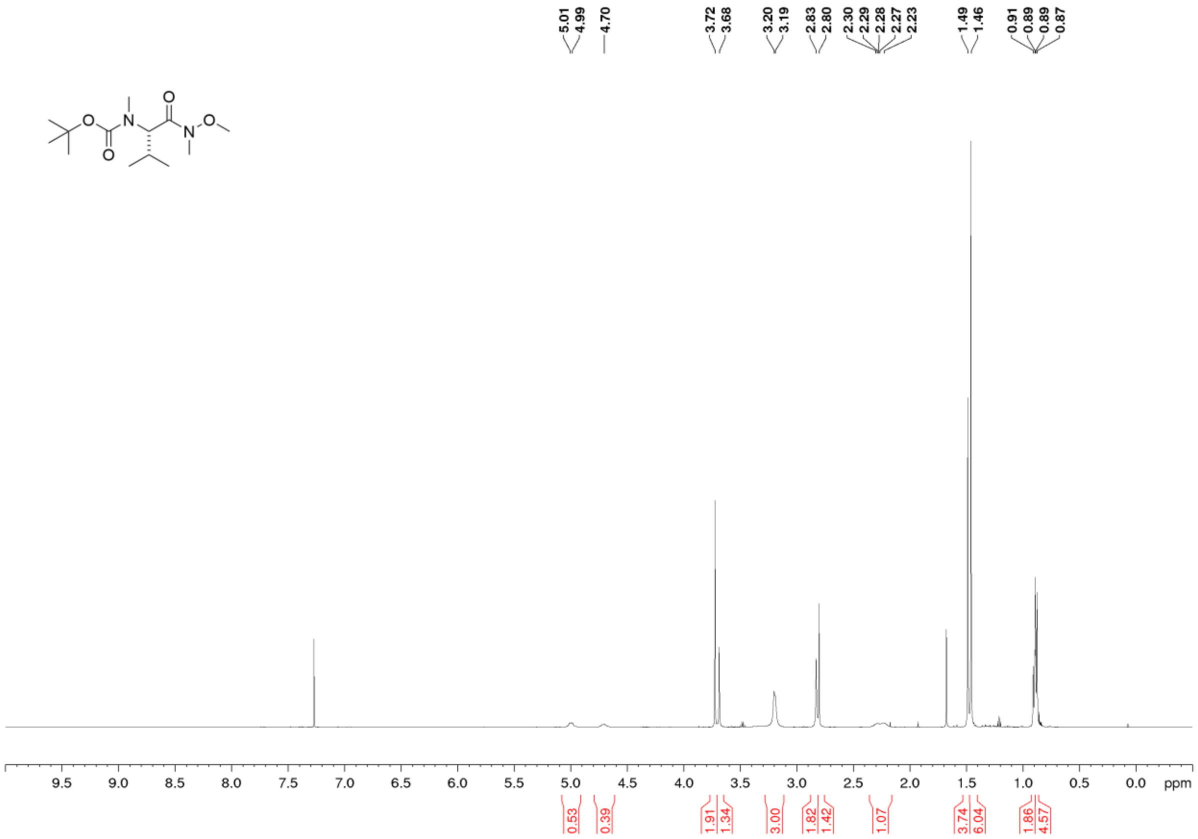

^{13}C-NMR

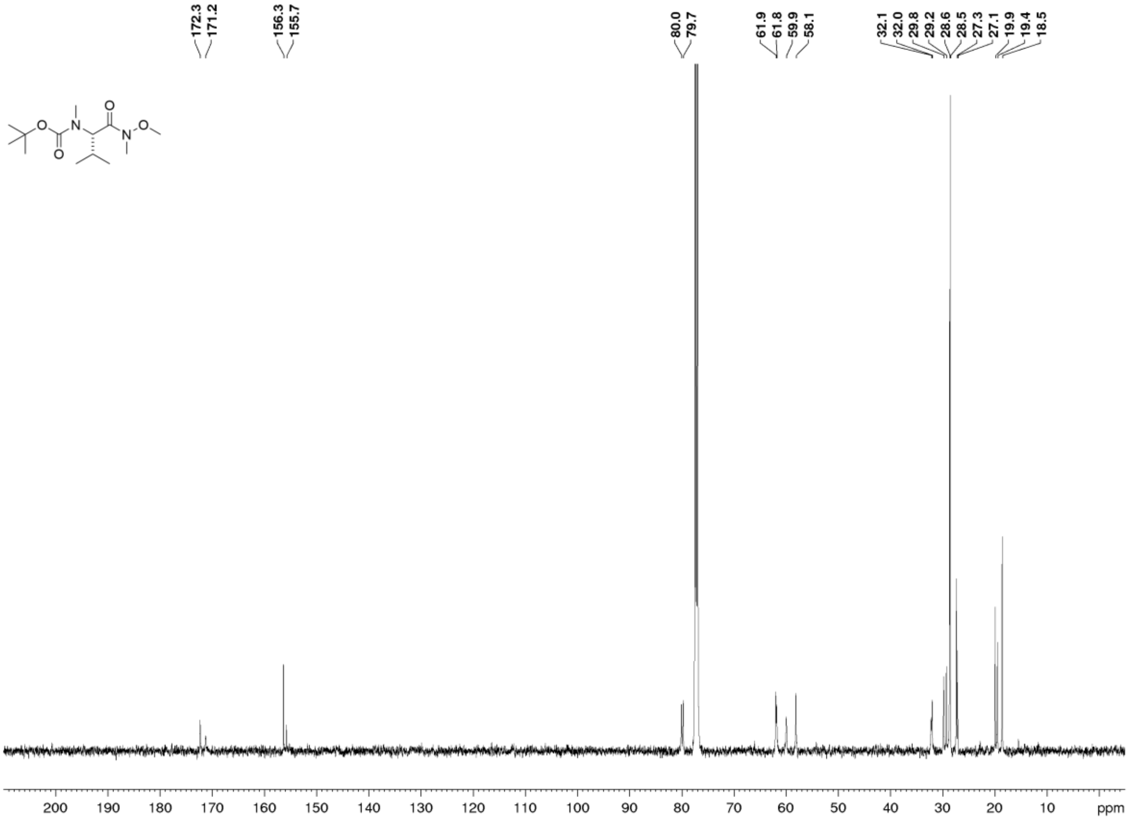

Ethyl (2*E*,4*S*)-*N*-*tert*-butoxycarbonyl-*N*-methyl-4-amino-2,5-dimethylhex-2-enoate (10)

^{1}H-NMR

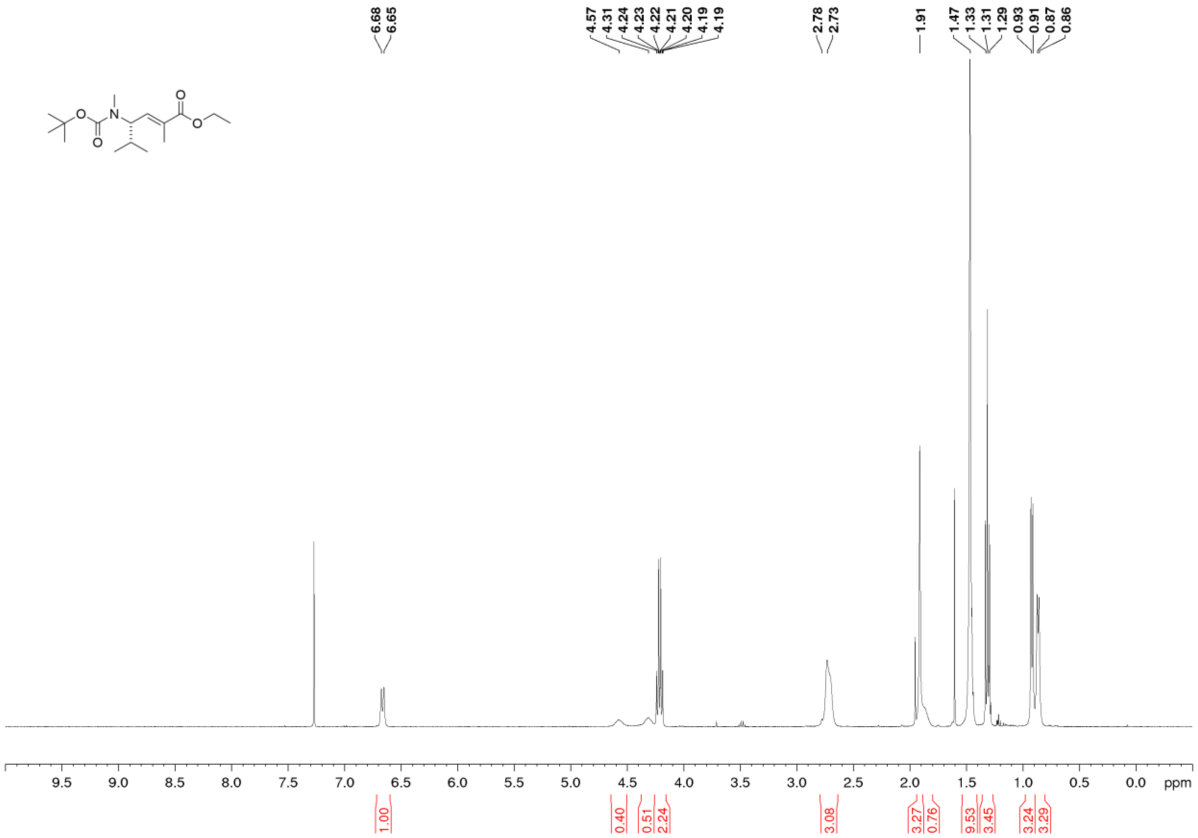

^{13}C-NMR

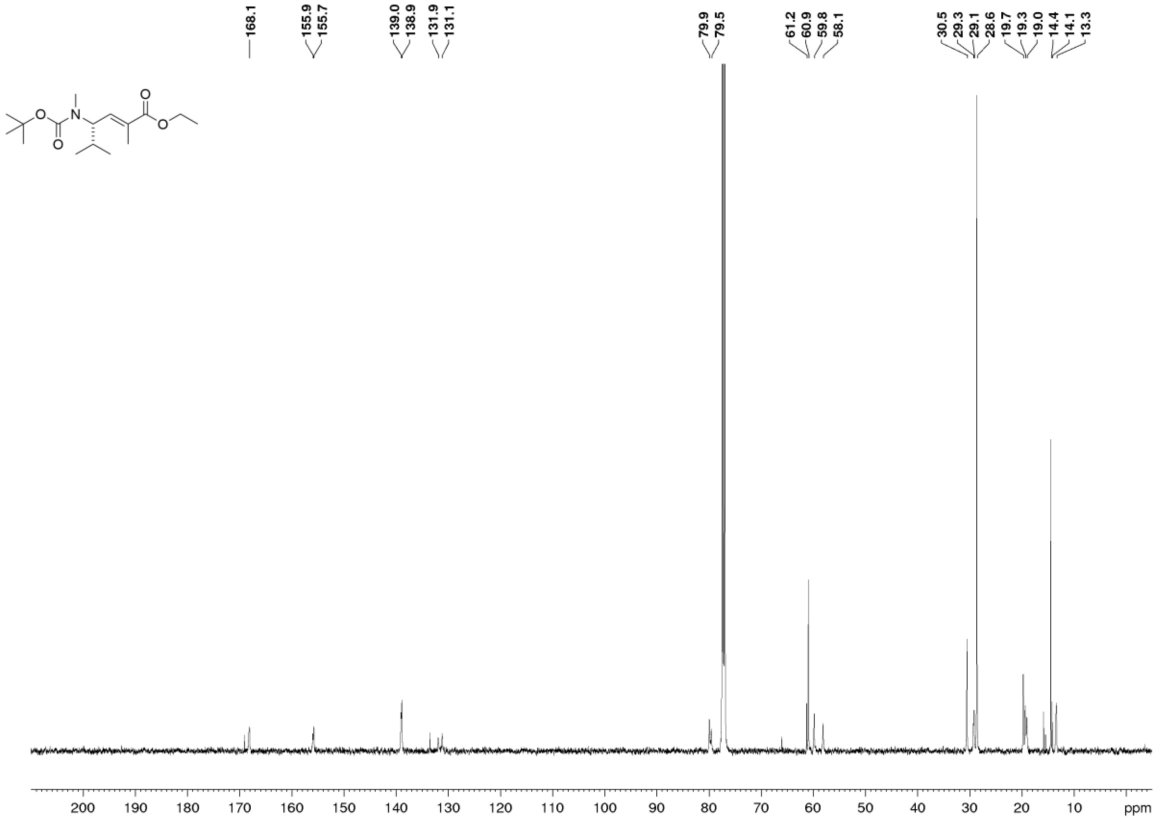

Ethyl (*S*,*E*)-4-((*S*)-2-((*tert*-butoxycarbonyl)amino)-*N*,3,3-trimethylbutanamido)-2,5-dimethylhex-2-enoate (16)

^{1}H-NMR

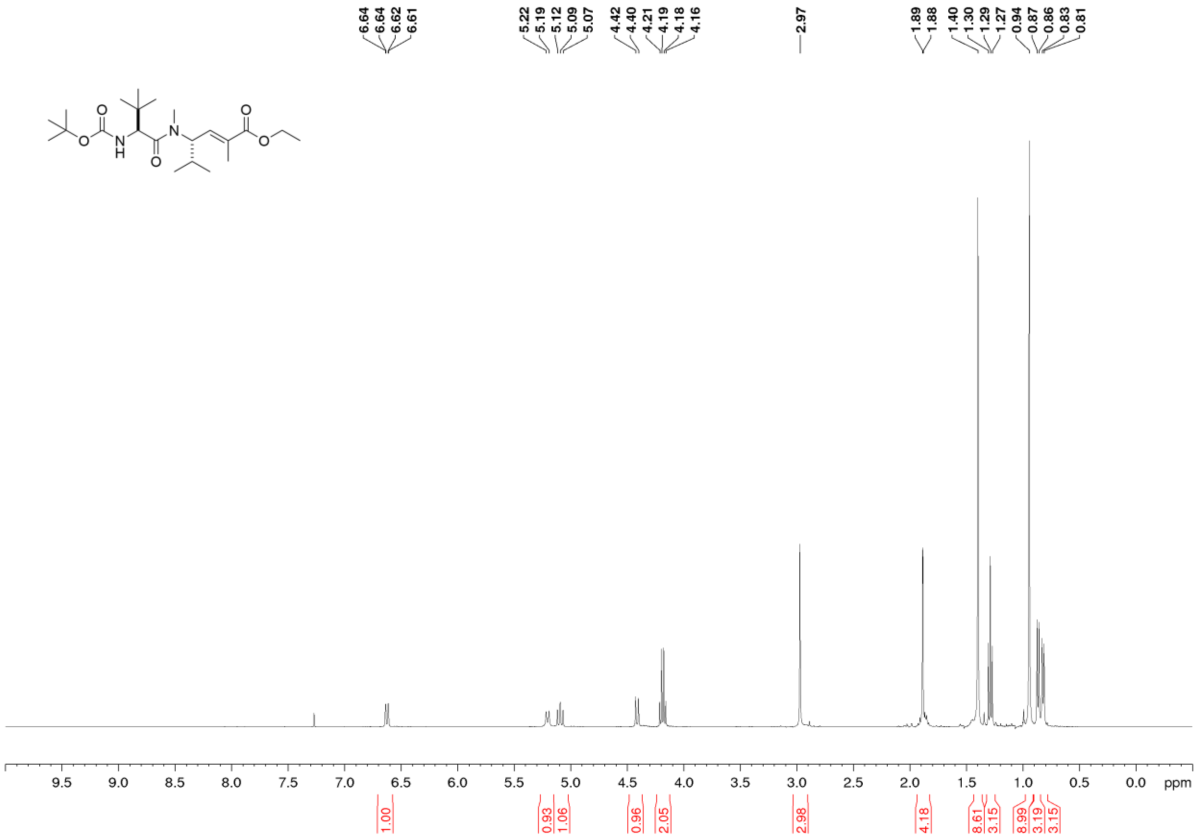

^{13}C-NMR

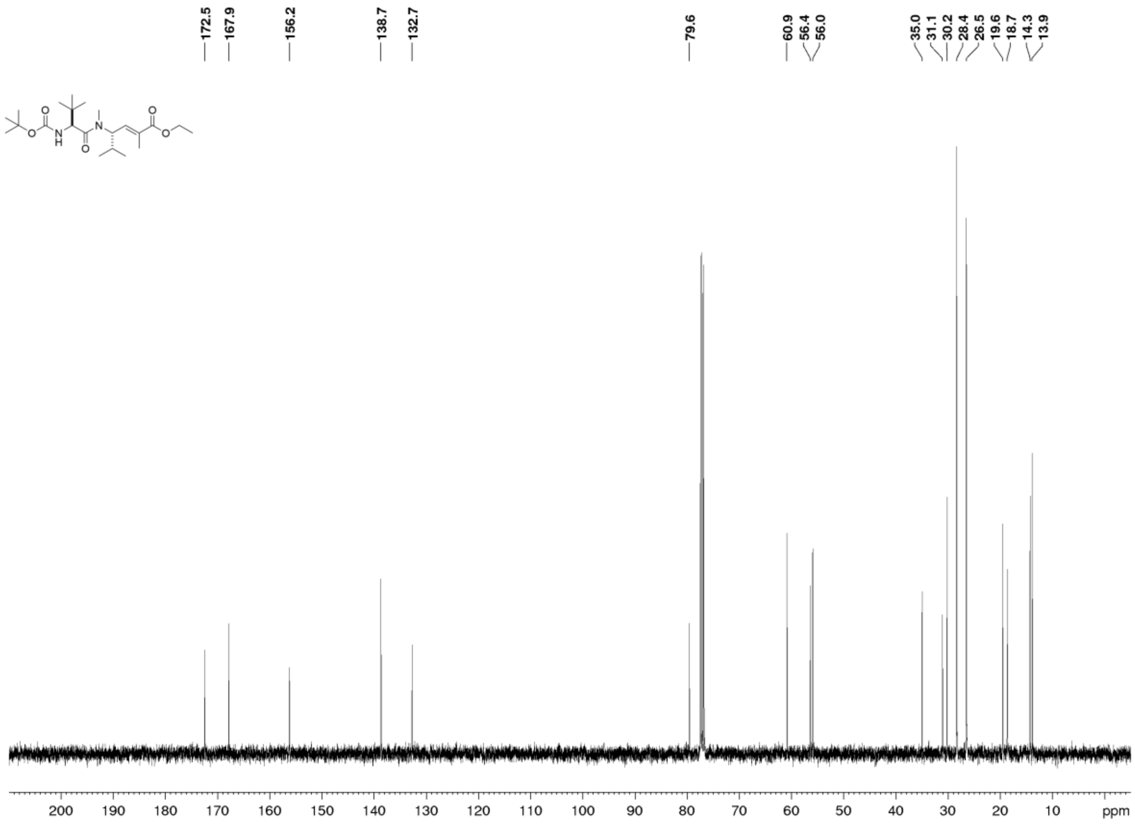

Ethyl (*S*,*E*)-4-((*S*)-2-formamido-*N*,3,3-trimethylbutanamido)-2,5-dimethylhex-2-enoate (S7)

^{1}H-NMR

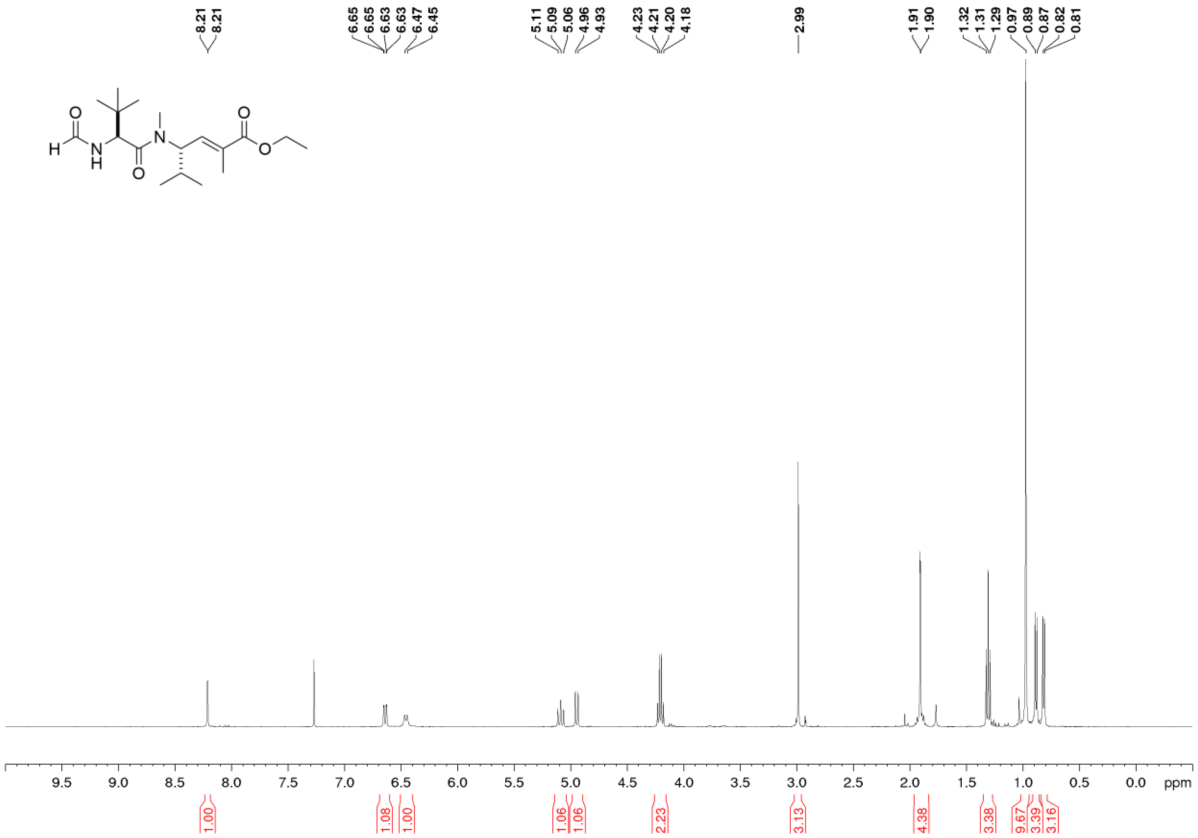

^{13}C-NMR

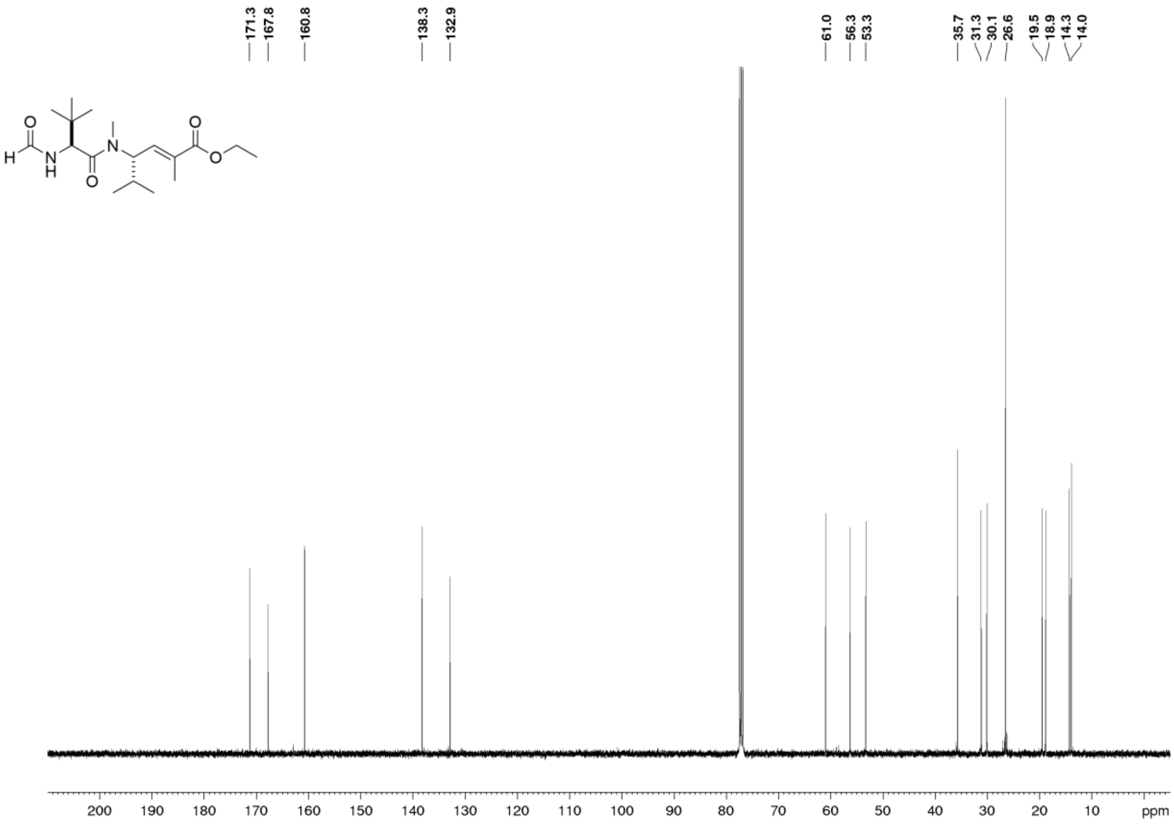

Ethyl (*S*,*E*)-4-((*S*)-2-isocyano-*N*,3,3-trimethylbutanamido)-2,5-dimethylhex-2-enoate (17)

^{1}H-NMR

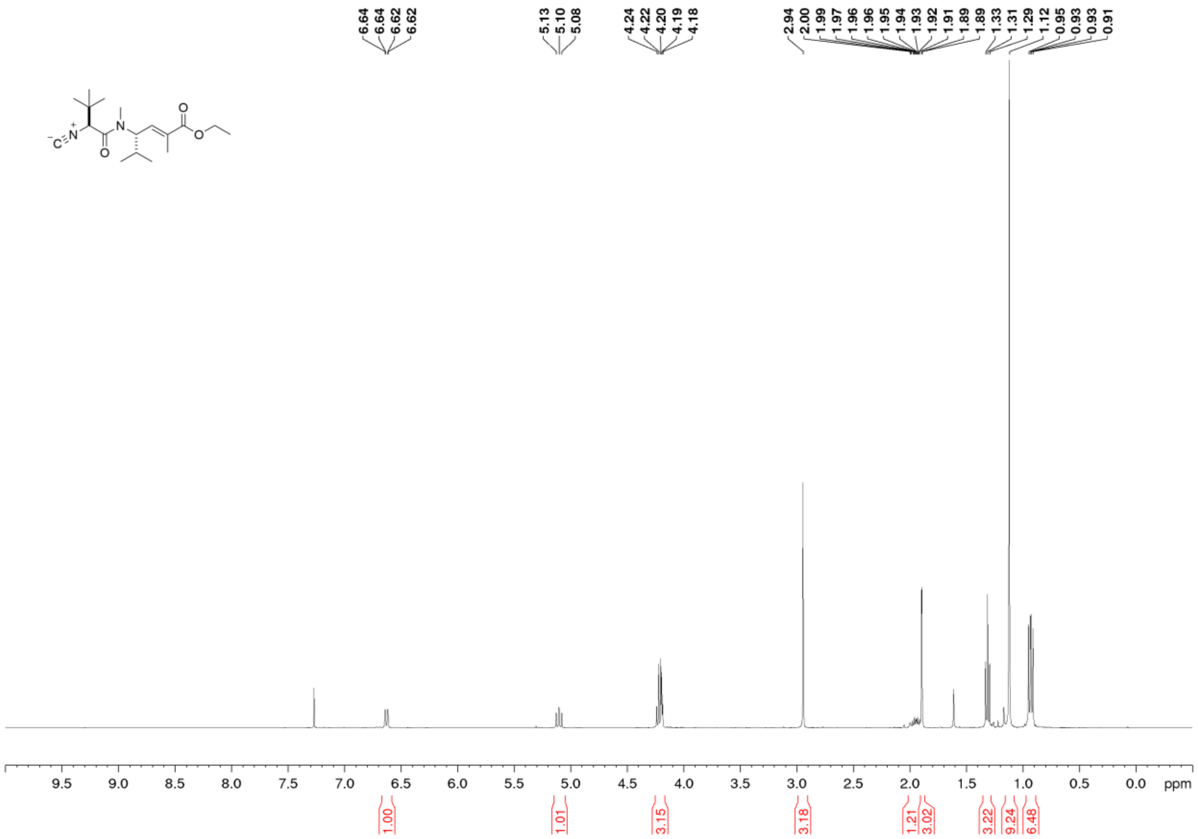

^{13}C-NMR

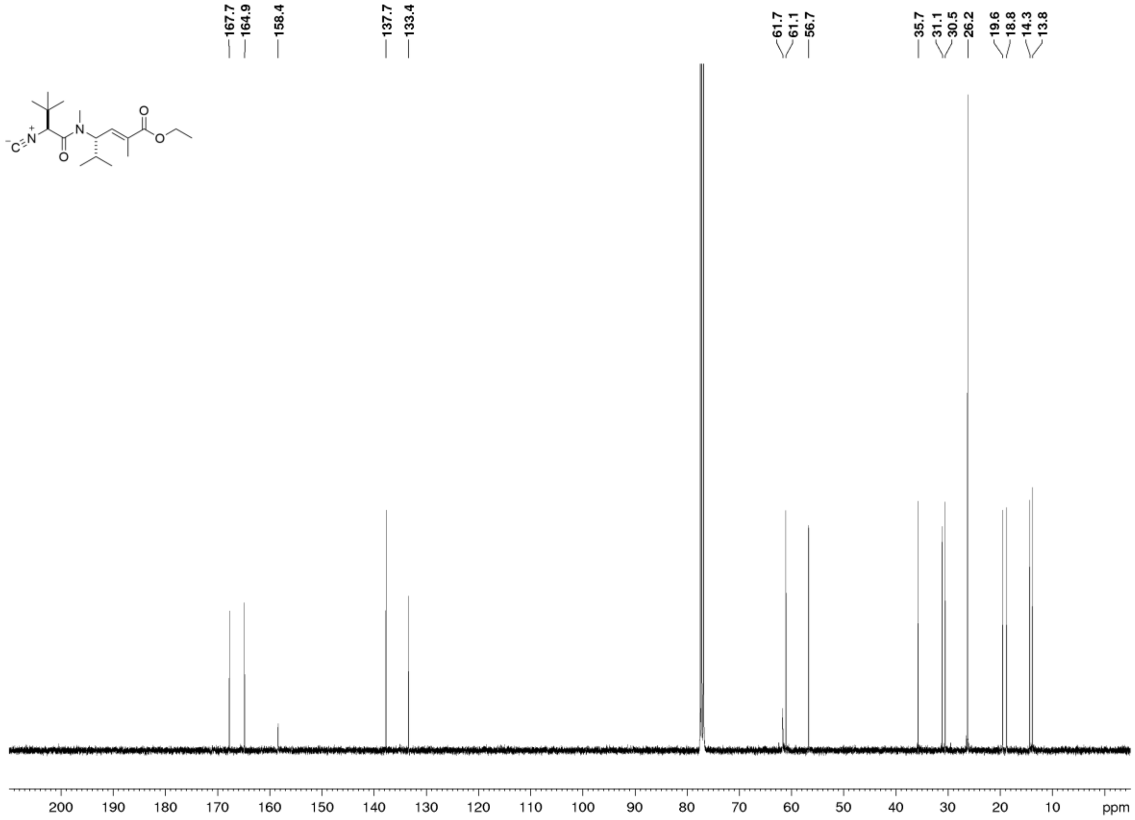

***N*-trifluoroacetyl hemiasterlin ethyl ester (15a)**

^{1}H-NMR

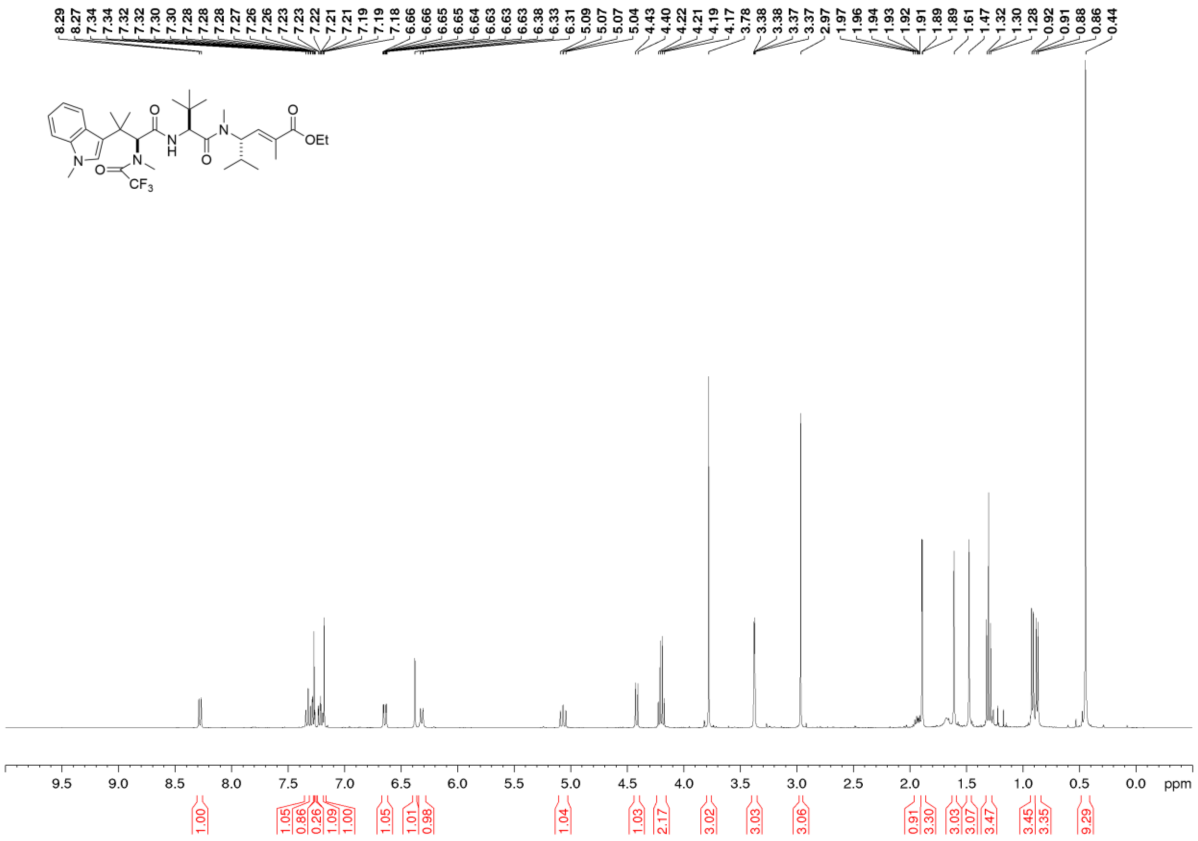

^{13}C-NMR

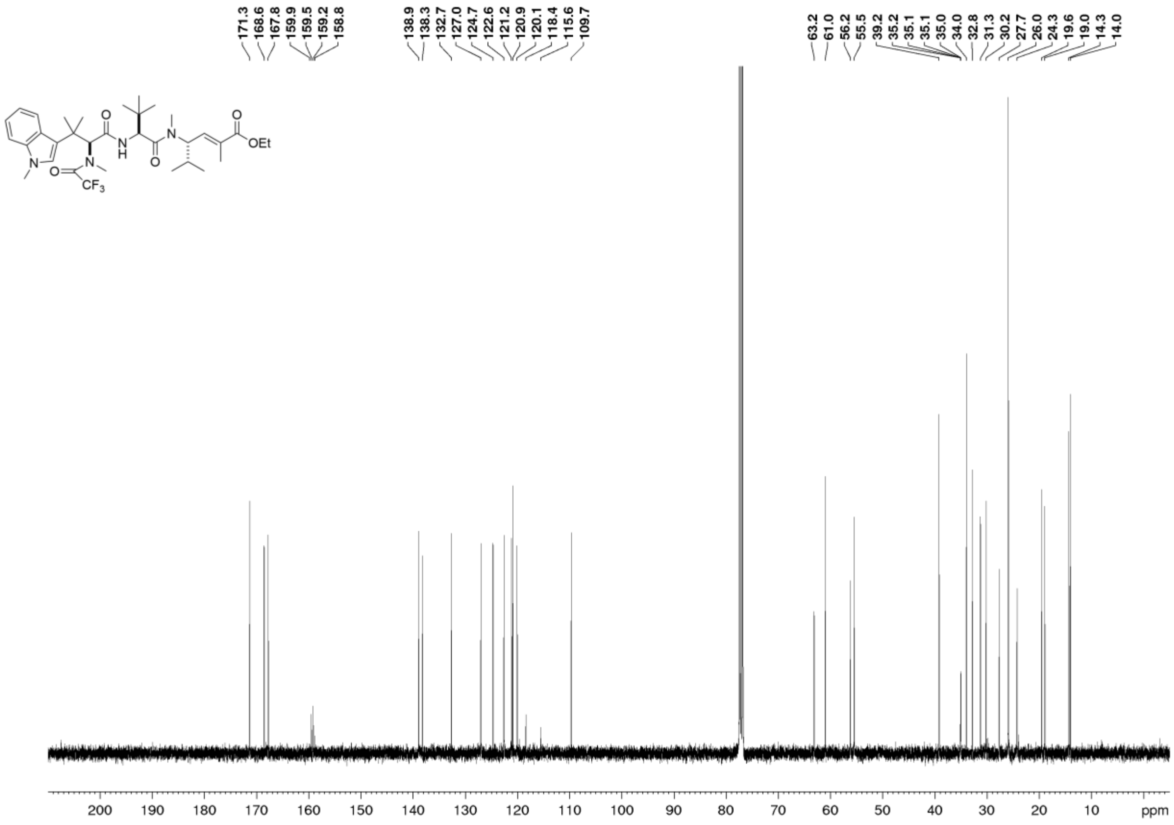

^{19}F-NMR

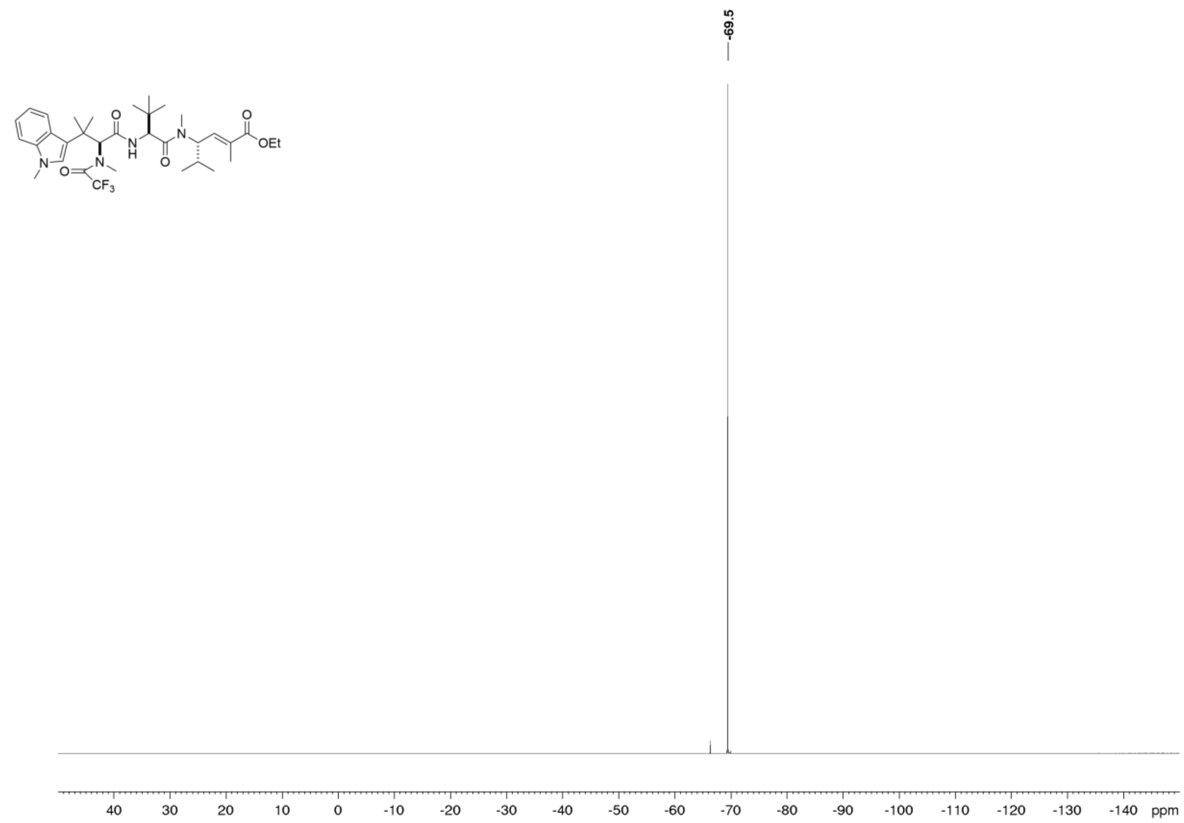
-69.5
OEt
N
H
CF3
40 30 20 10 0 -10 -20 -30 -40 -50 -60 -70 -80 -90 -100 -110 -120 -130 -140 ppm

***N*-trifluoroacetyl *epi*-hemiasterlin ethyl ester (15b)**

^{1}H-NMR

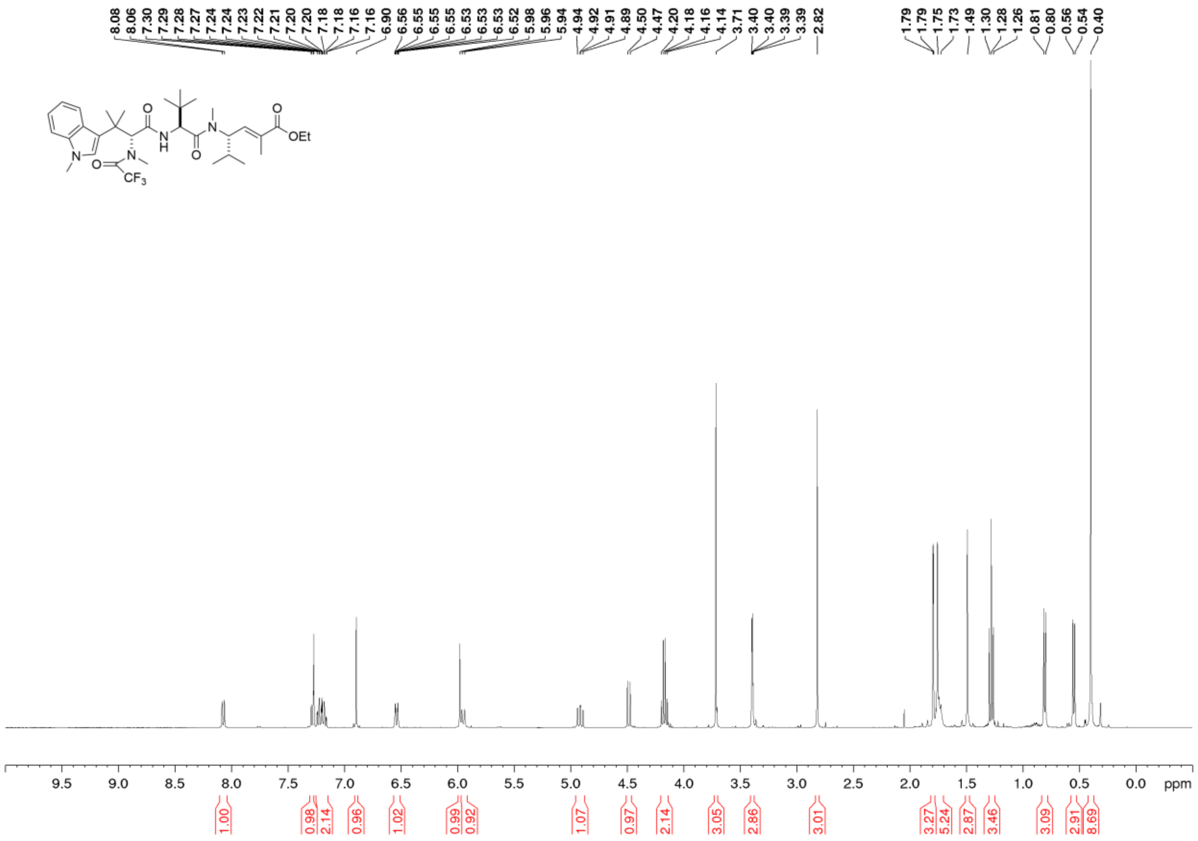

^{13}C-NMR

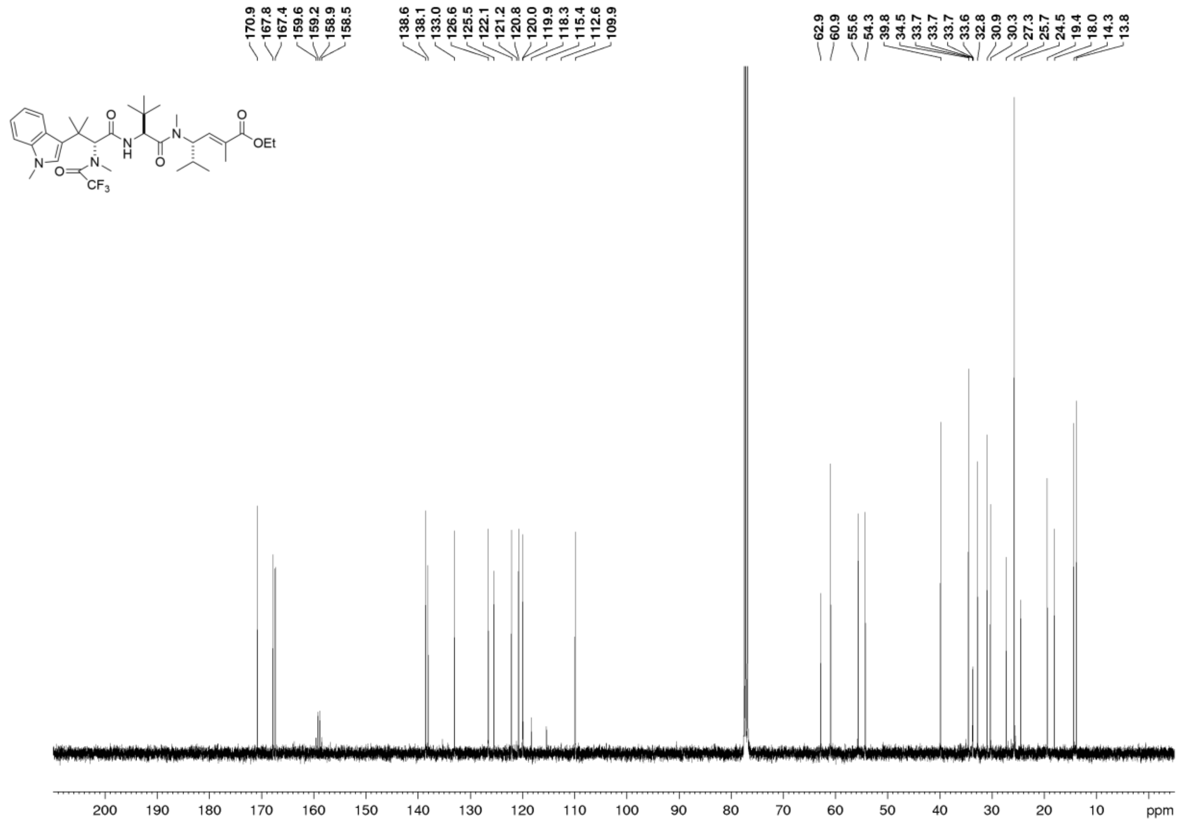

^{19}F-NMR

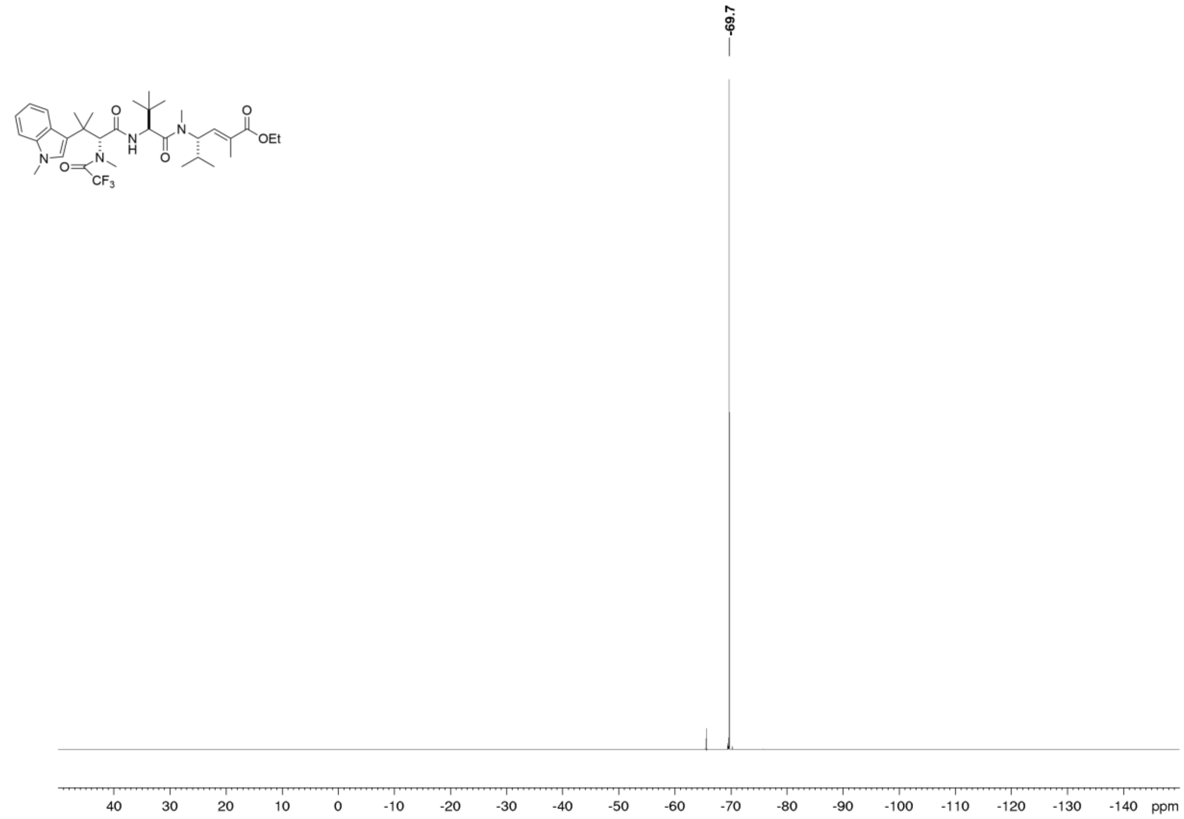
-69.7
N
O
CF3
N
H
O
N
O
OEt
40 30 20 10 0 -10 -20 -30 -40 -50 -60 -70 -80 -90 -100 -110 -120 -130 -140 ppm

Hemiasterlin trifluoroacetate salt (1•TFA)

^{1}H-NMR

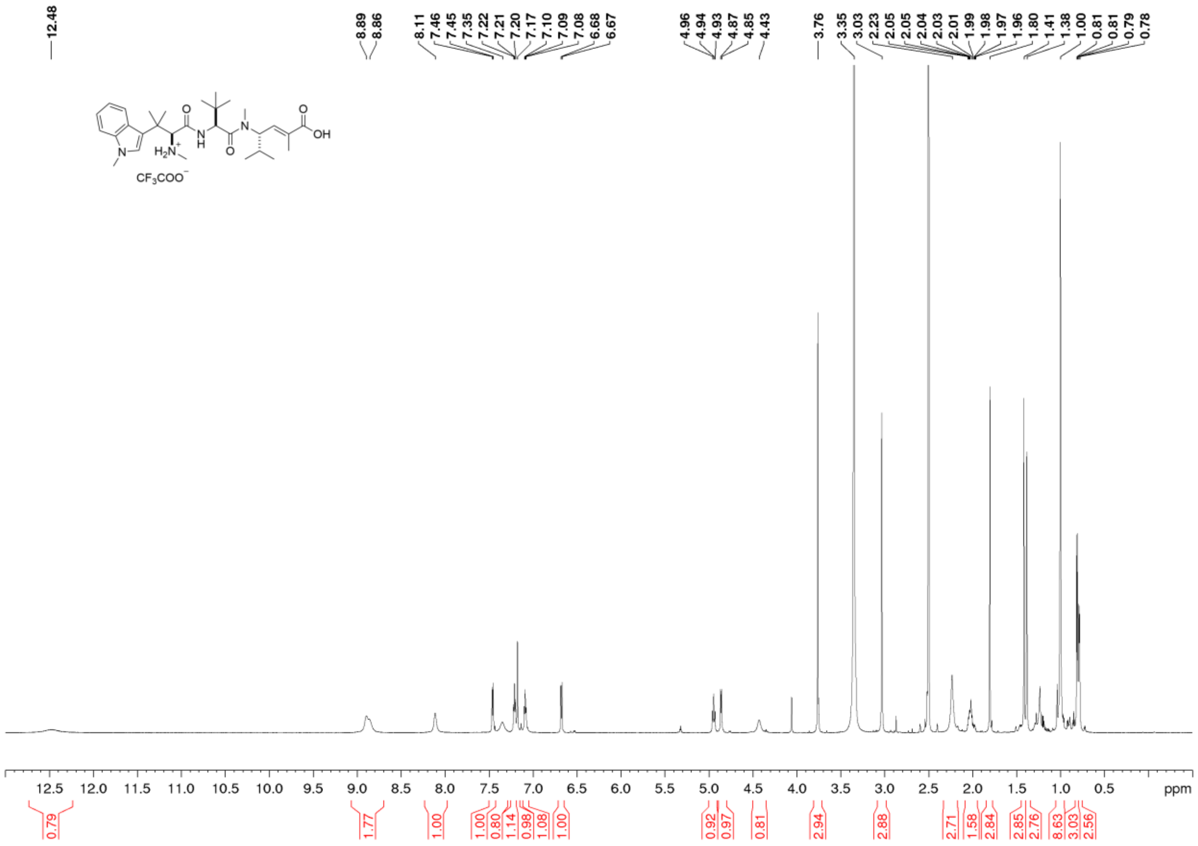

^{13}C-NMR

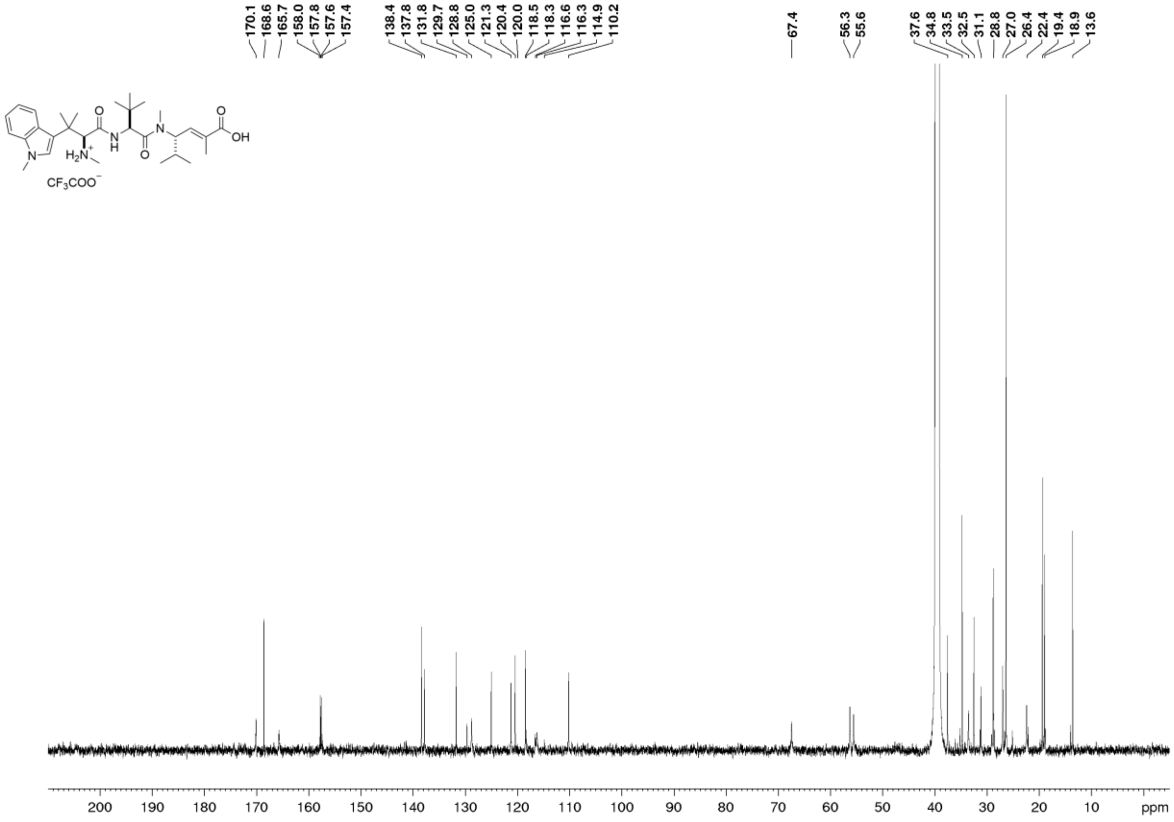

^{19}F-NMR

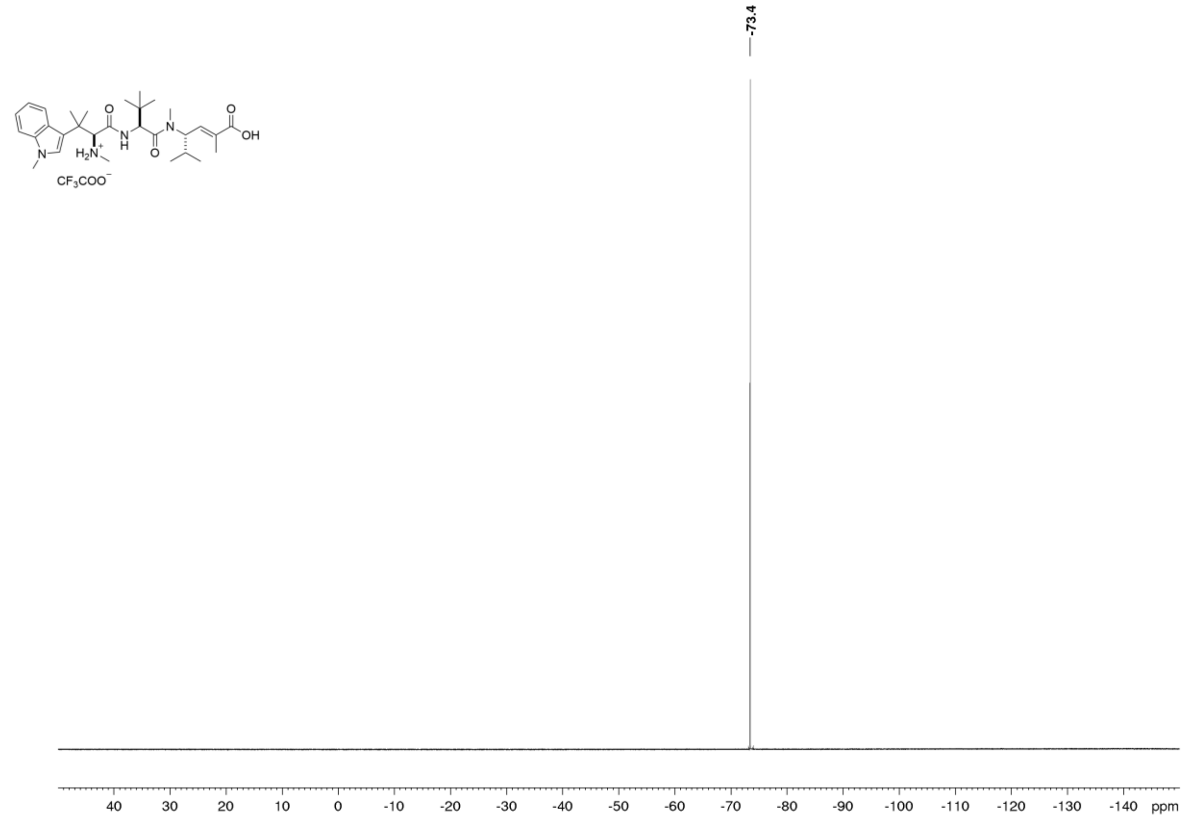
-73.4
H_2N^+
NH
N
OH
CF_3COO^-
40 30 20 10 0 -10 -20 -30 -40 -50 -60 -70 -80 -90 -100 -110 -120 -130 -140 ppm

***epi*-hemiasterlin trifluoroacetate salt (1b•TFA)**

^{1}H-NMR

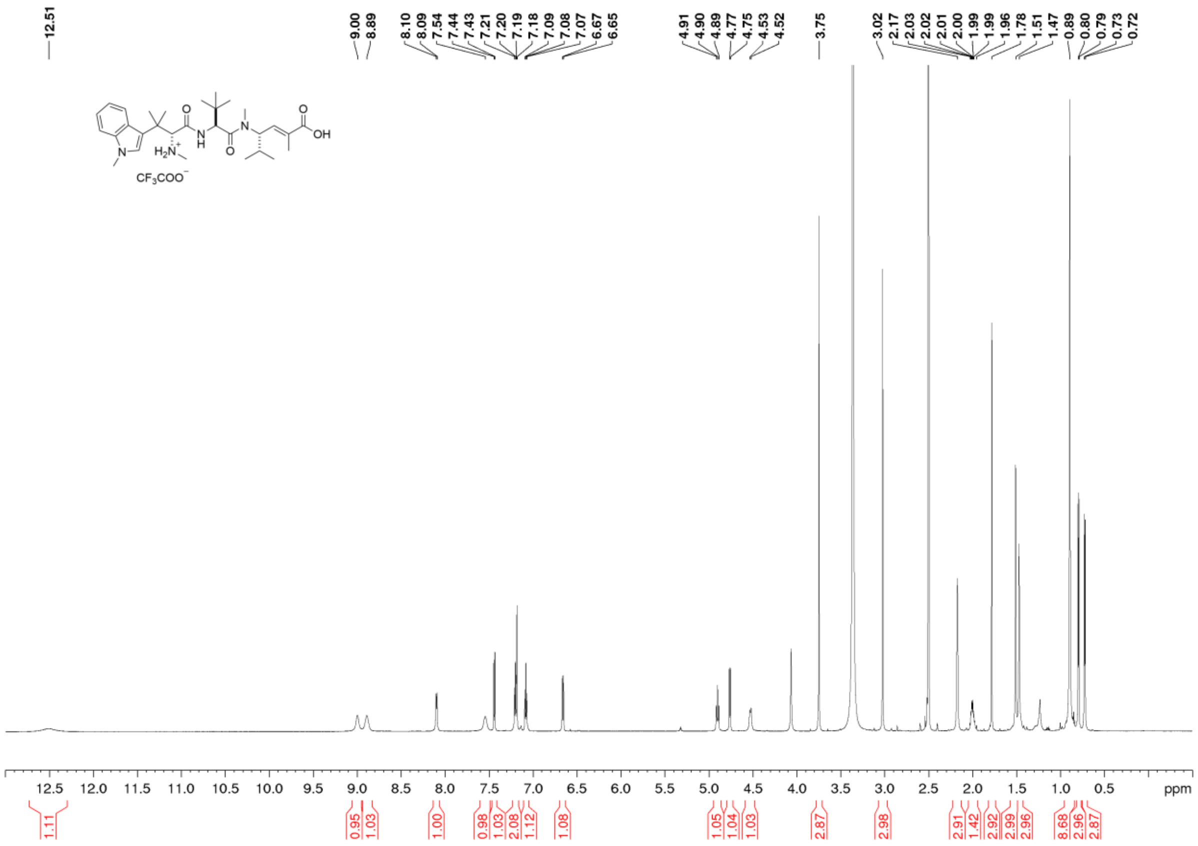

^{13}C-NMR

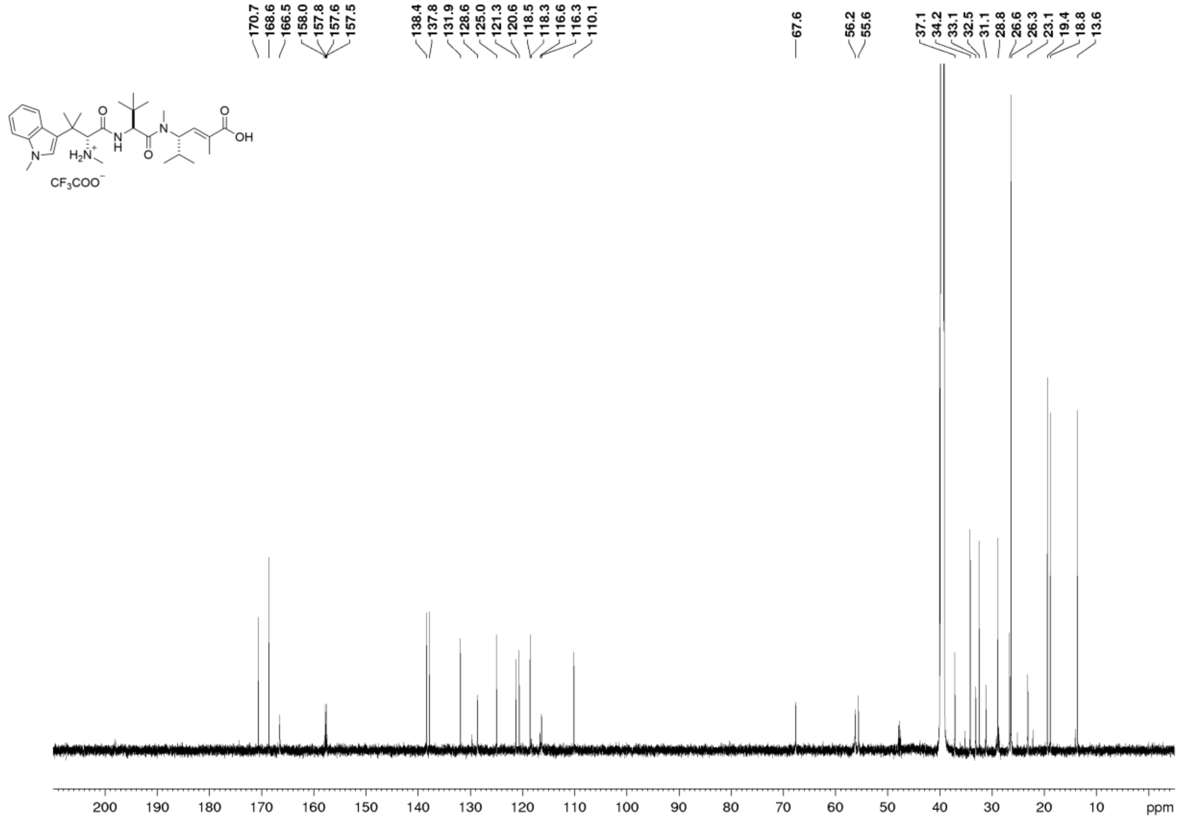

^{19}F-NMR

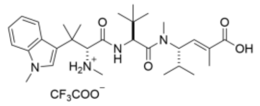
H2N+
CF3COO−
OH

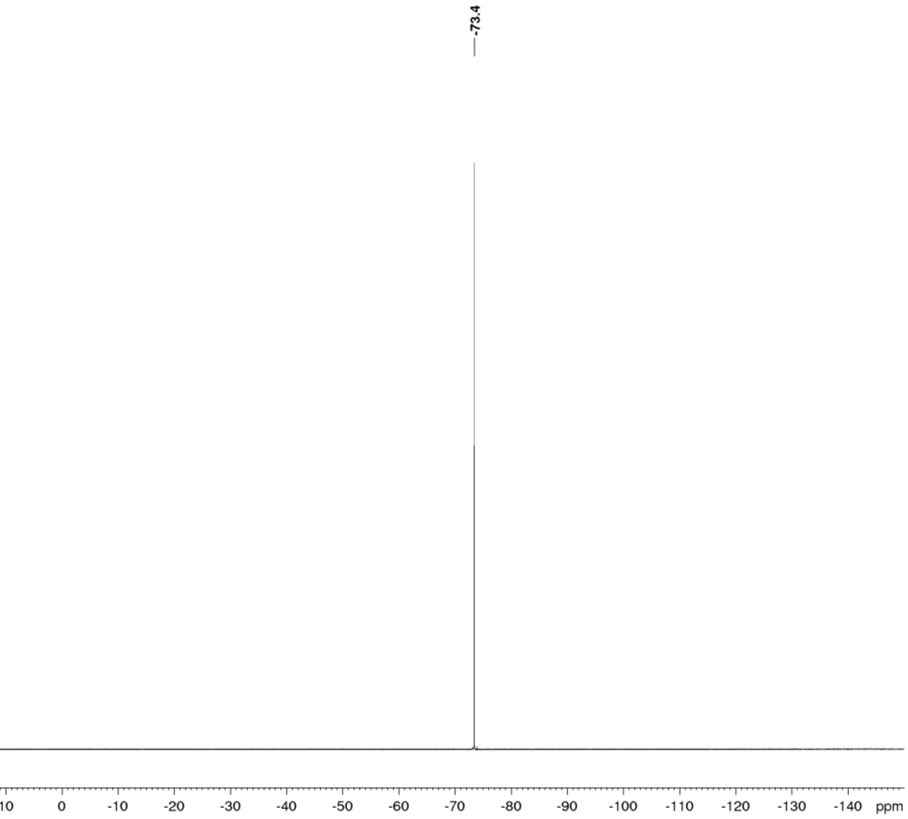
-73.4
40
30
20
10
0
-10
-20
-30
-40
-50
-60
-70
-80
-90
-100
-110
-120
-130
-140
ppm

2-Methyl-2-phenylpropanal (18)

^{1}H-NMR

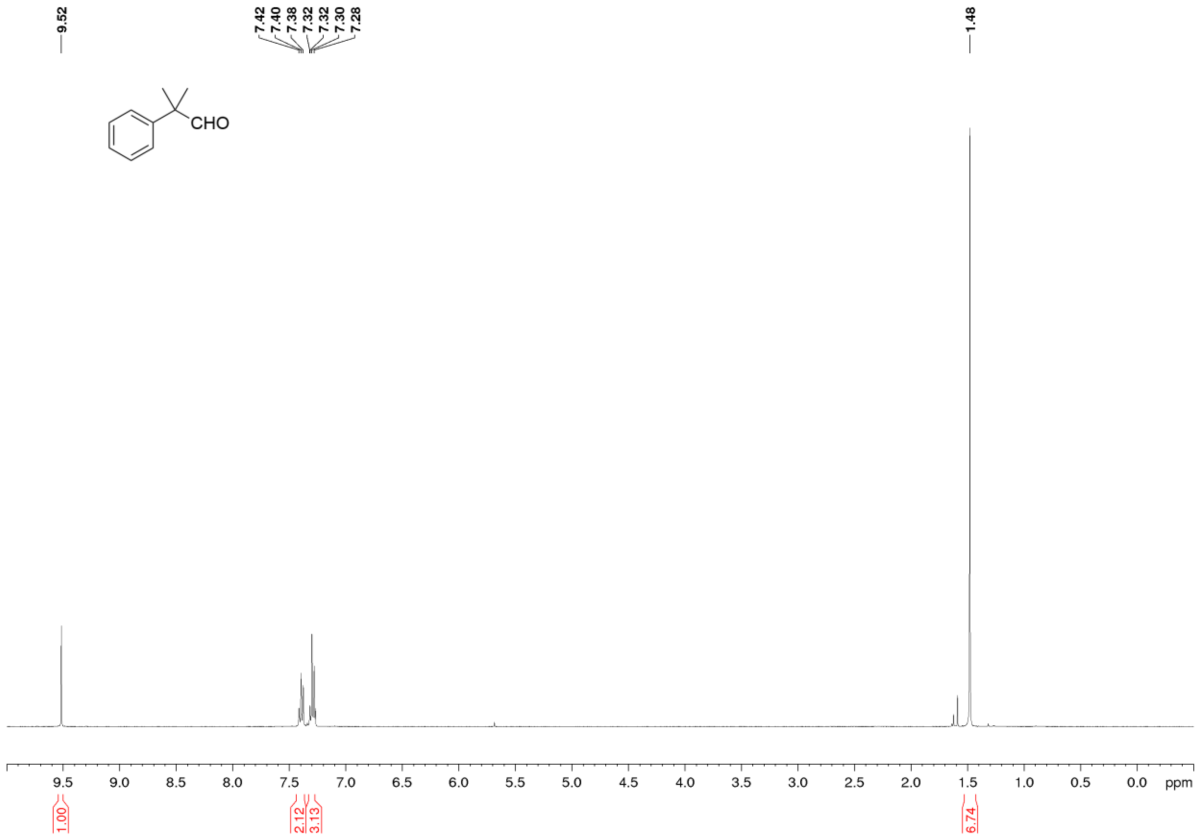

^{13}C-NMR

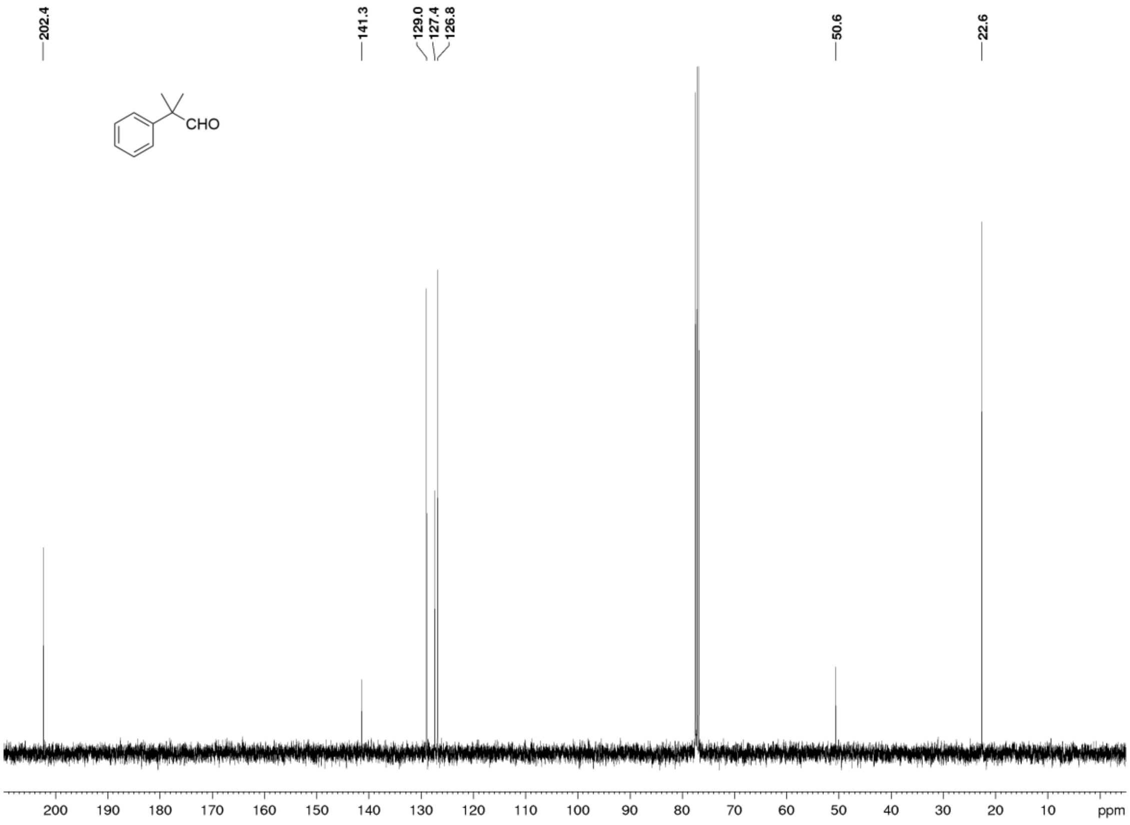

Taltobulin trifluoroacetate salt (8•TFA)

^{1}H-NMR

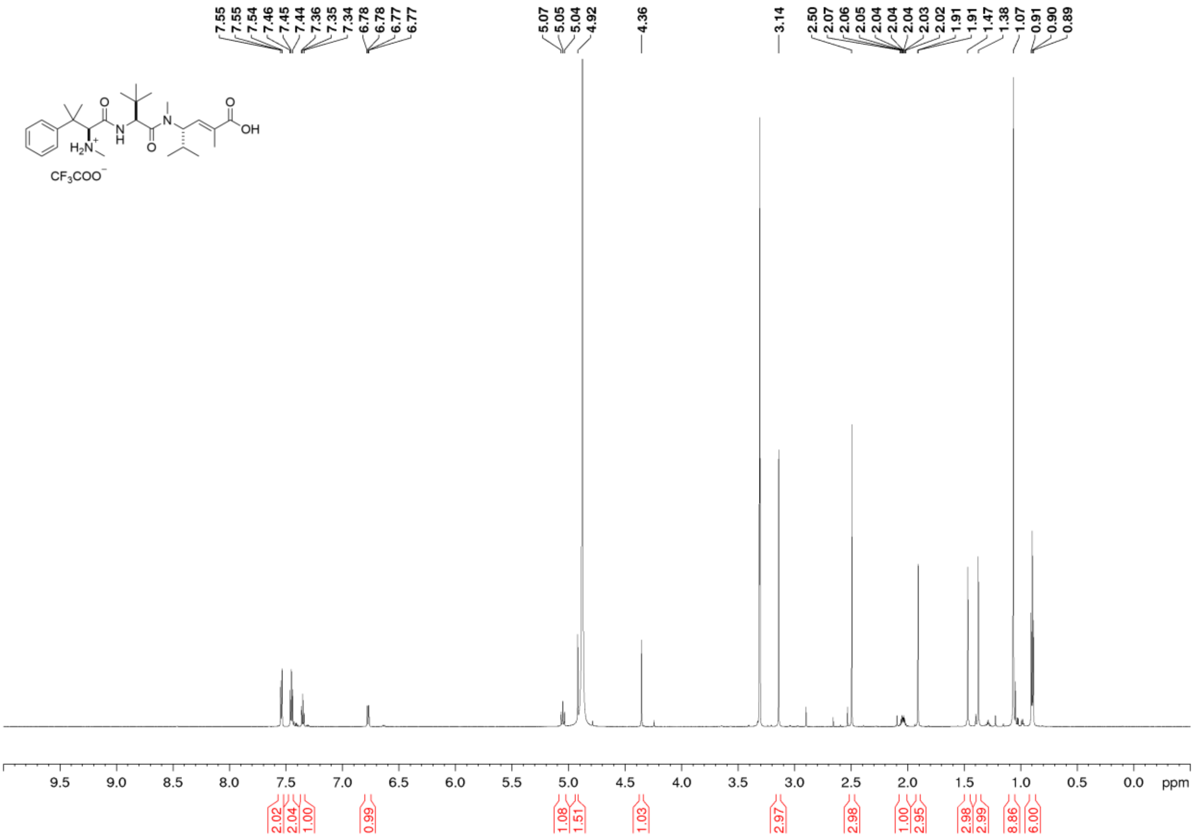

^{13}C-NMR

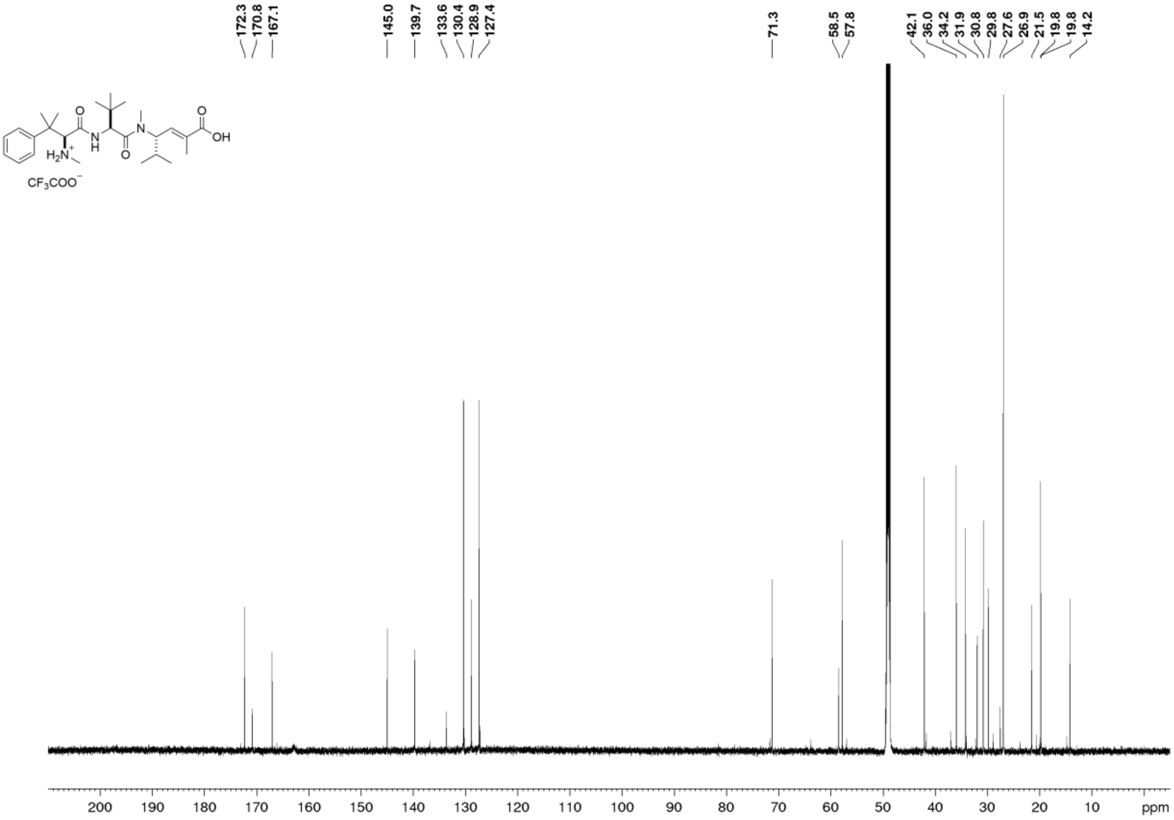

^{19}F-NMR

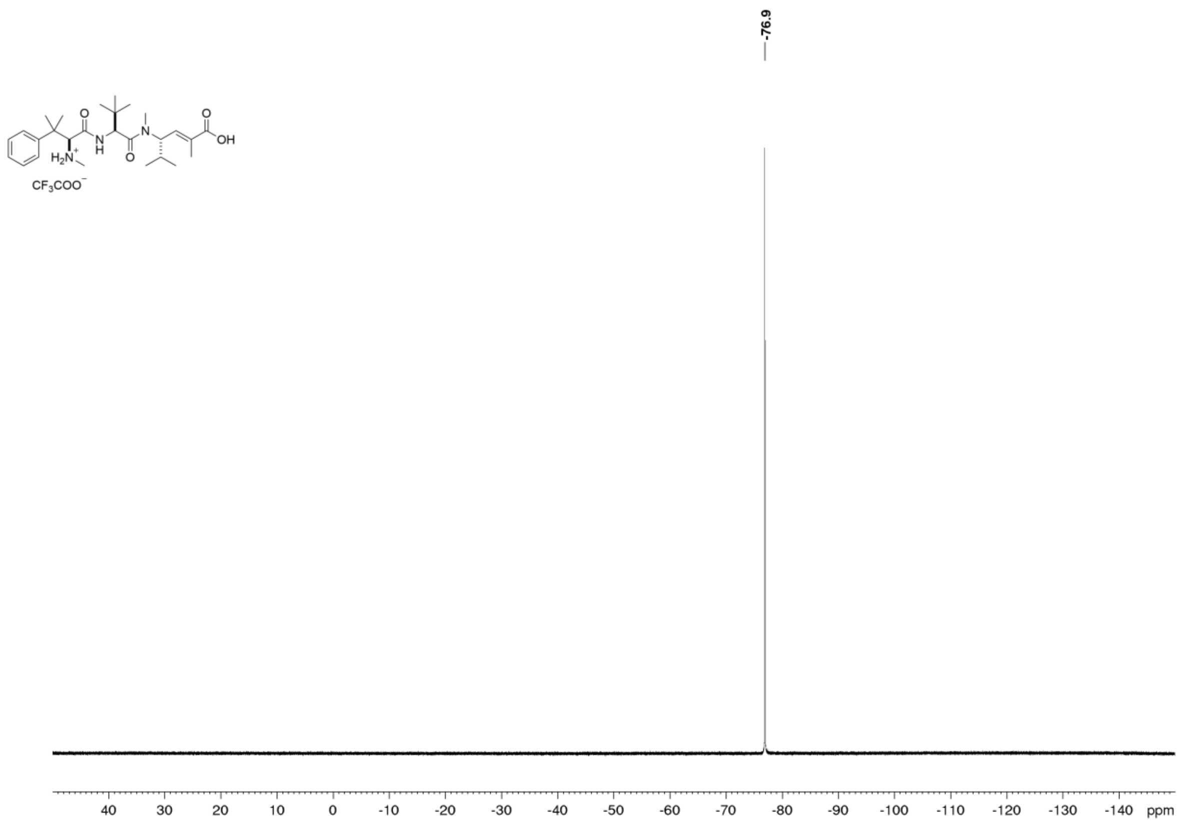
-76.9
H_2N^+
CF_3COO^-
OH
40
30
20
10
0
-10
-20
-30
-40
-50
-60
-70
-80
-90
-100
-110
-120
-130
-140
ppm

epi-Taltobulin trifluoroacetate salt (8b•TFA)
^{1}H-NMR

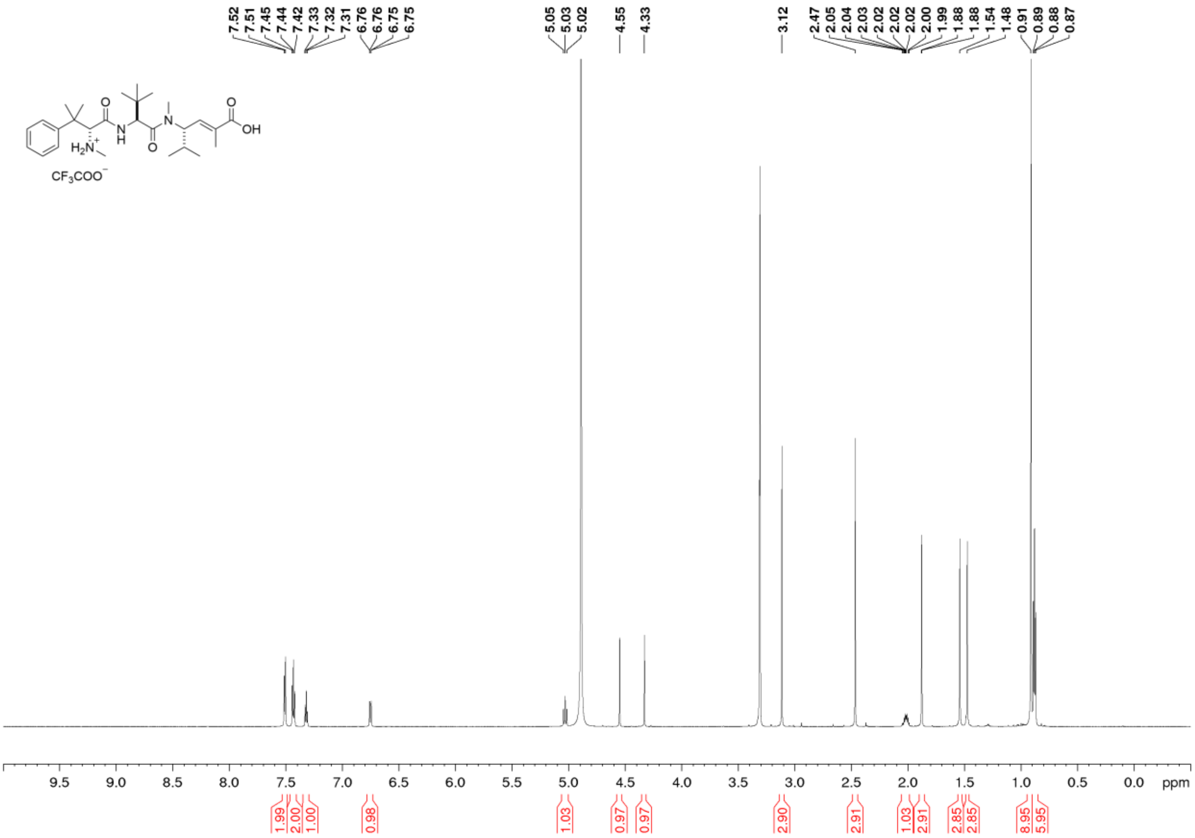

^{13}C-NMR

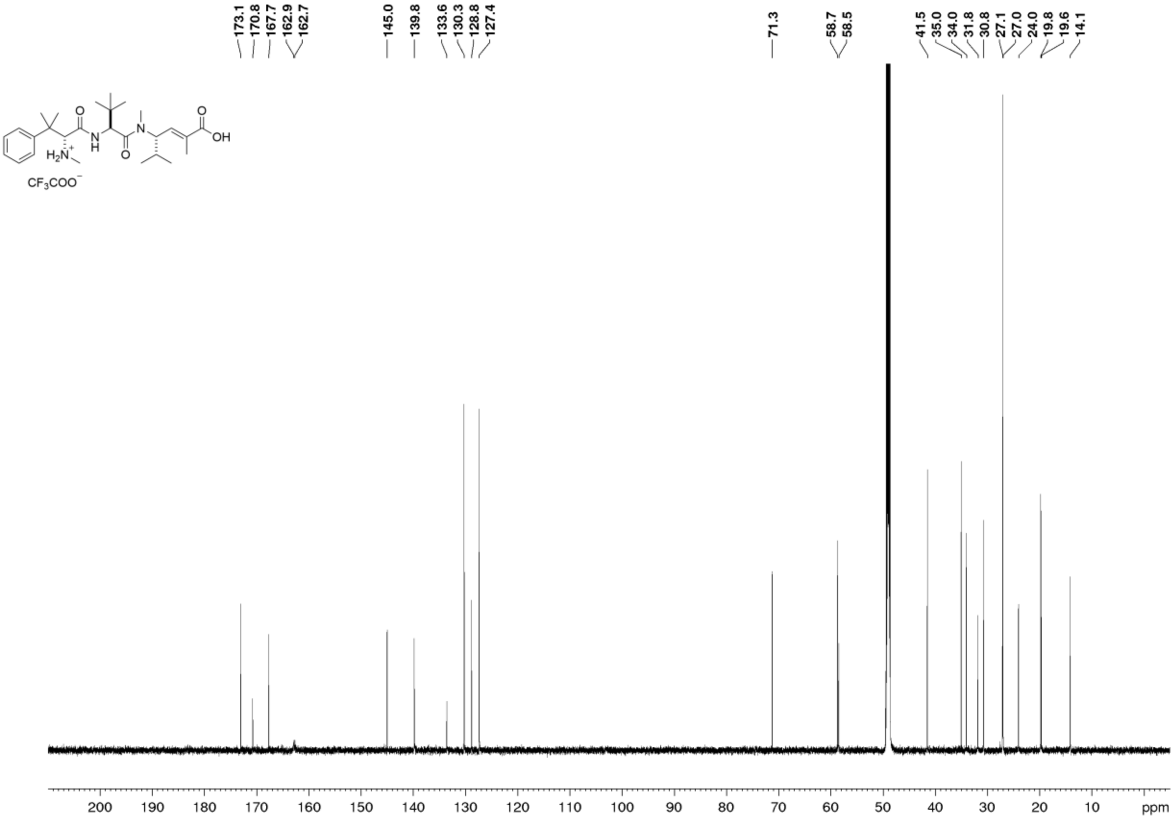

^{19}F-NMR

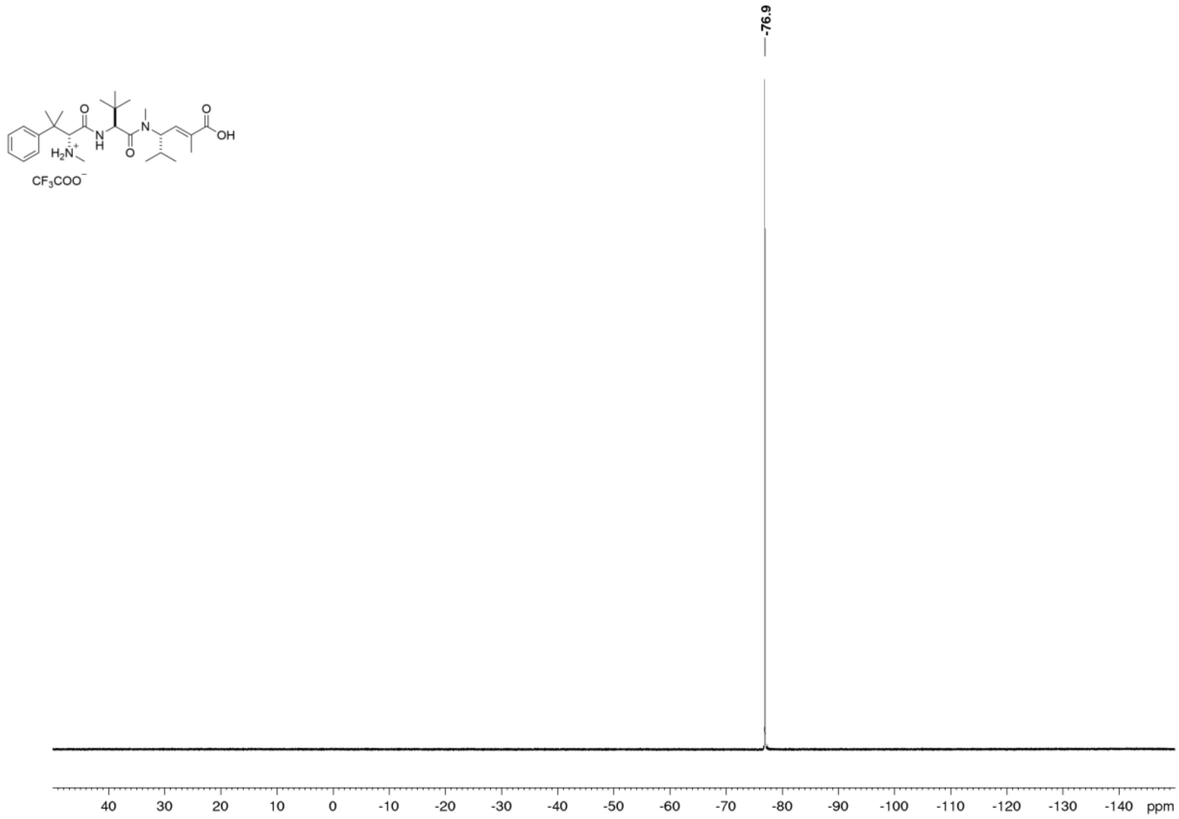
-76.9
H2N+
CF3COO−
OH
40 30 20 10 0 -10 -20 -30 -40 -50 -60 -70 -80 -90 -100 -110 -120 -130 -140 ppm

H-Val-Ala-PABA•TFA (S9)

^{1}H-NMR

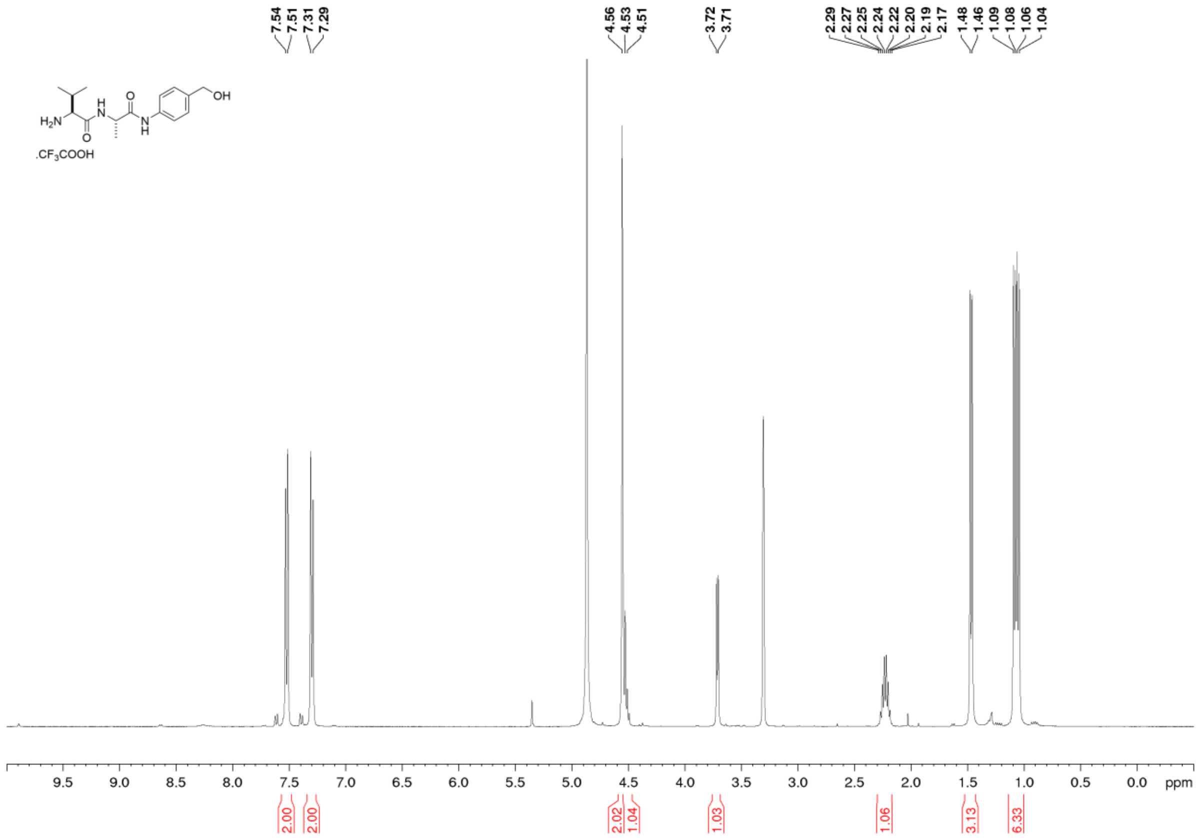

^{13}C-NMR

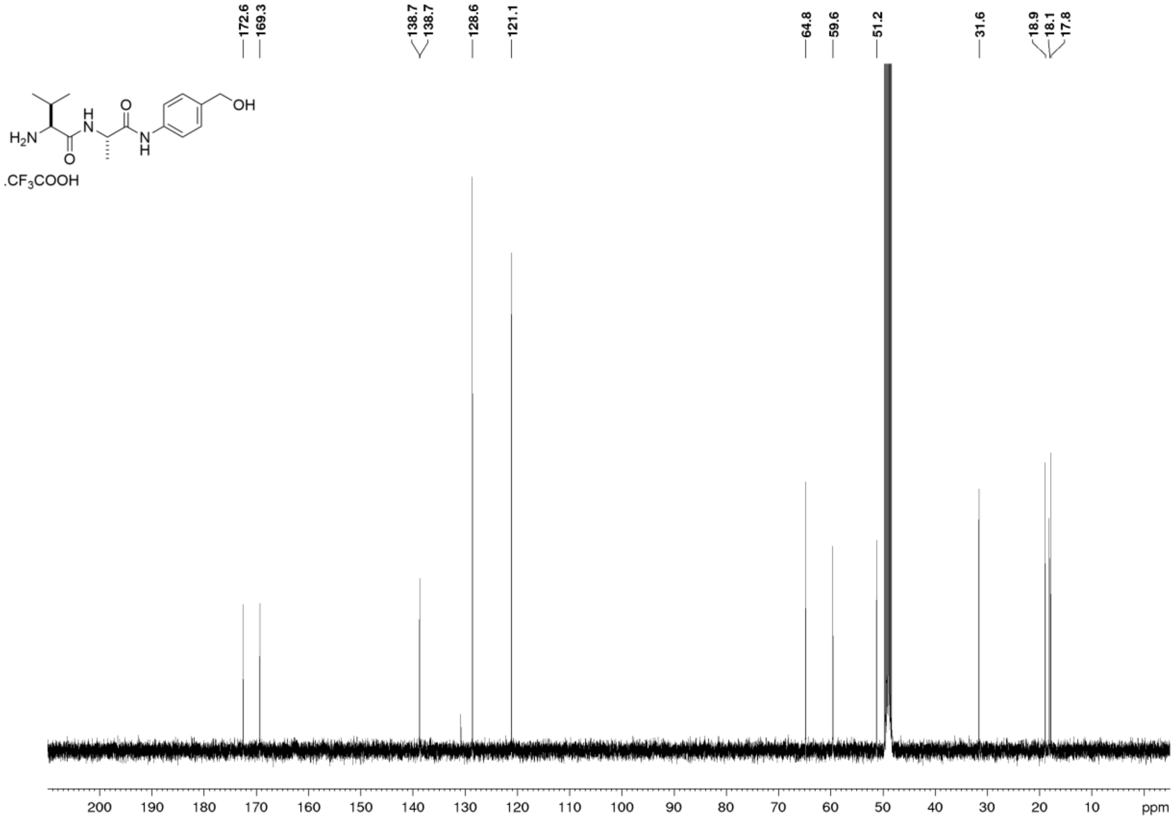

^{19}F-NMR

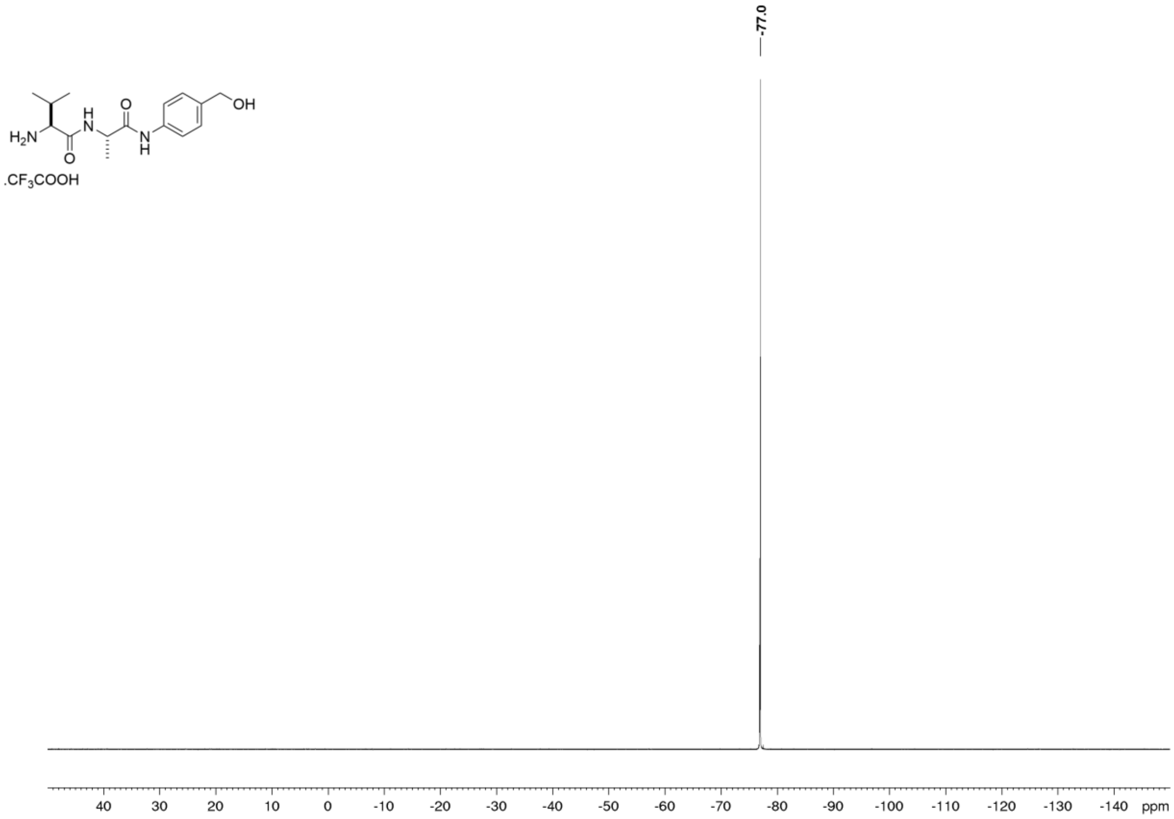
-77.0
OH
H
O
H2N
N
O
N
H
.CF3COOH
40
30
20
10
0
-10
-20
-30
-40
-50
-60
-70
-80
-90
-100
-110
-120
-130
-140
ppm

N_3-PEG$_4$-Val-Ala-PABA (S10)

^{1}H-NMR

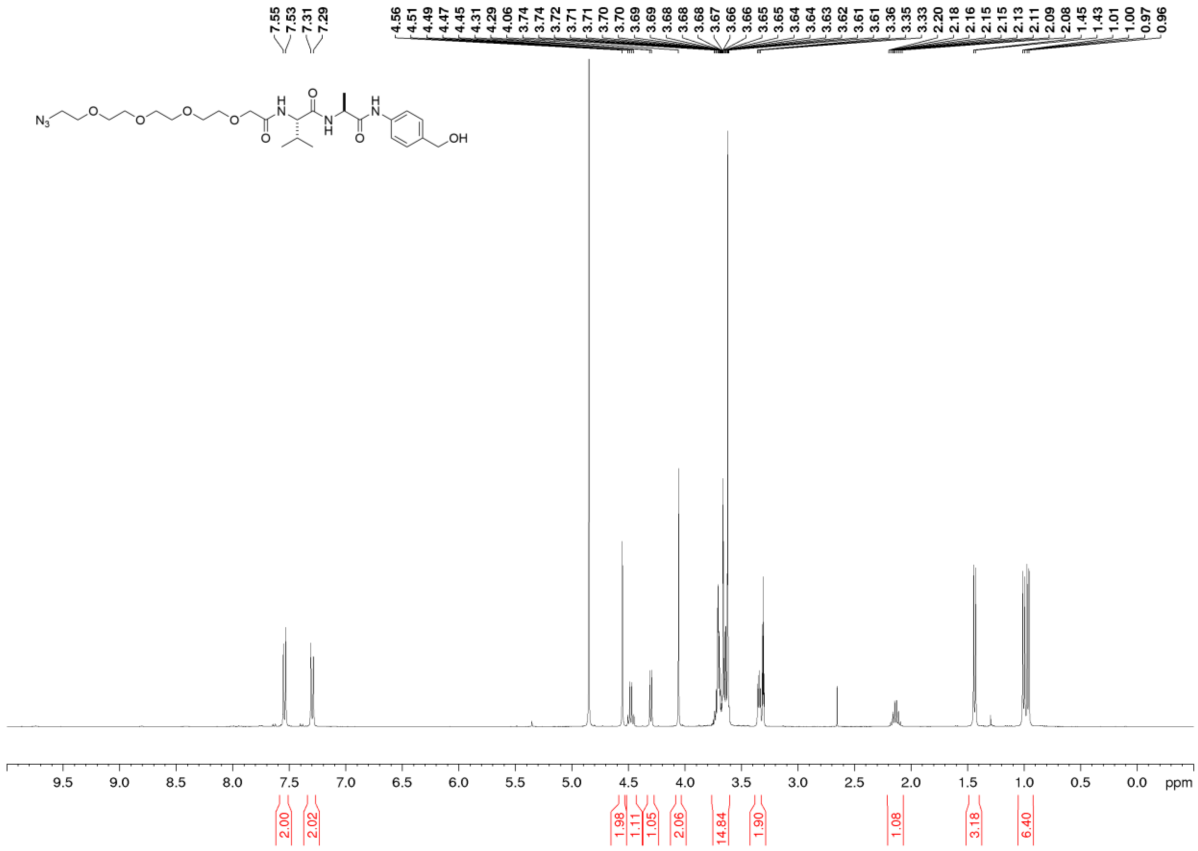

^{13}C-NMR

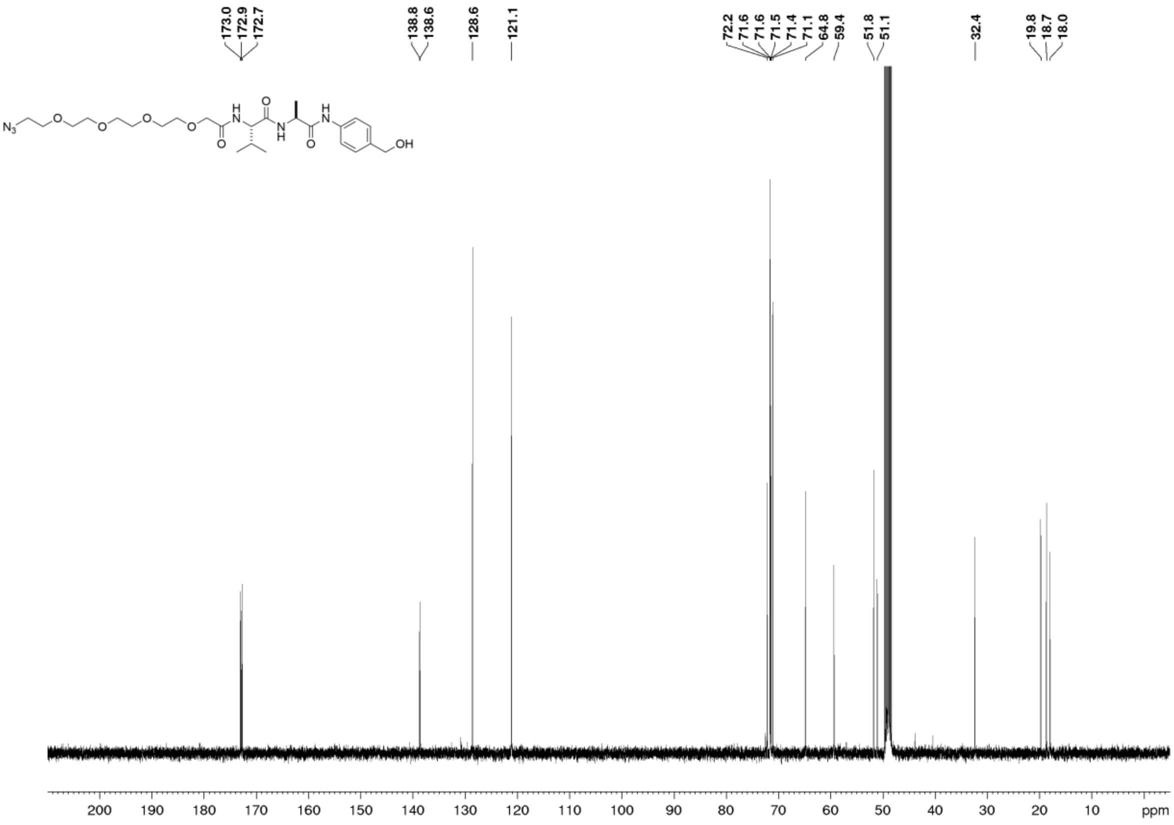

N_3-PEG$_4$-Val-Ala-PABC-OPNP (22)
^{1}H-NMR

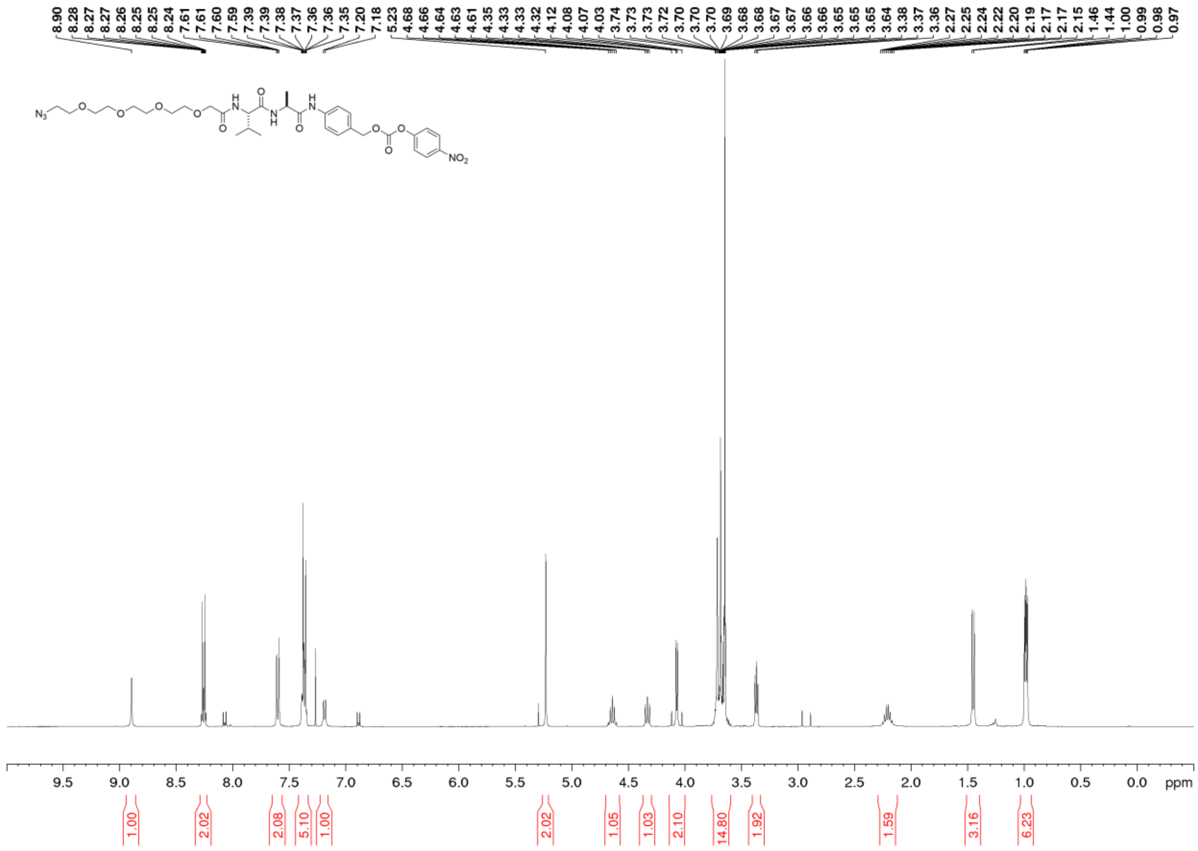

^{13}C-NMR

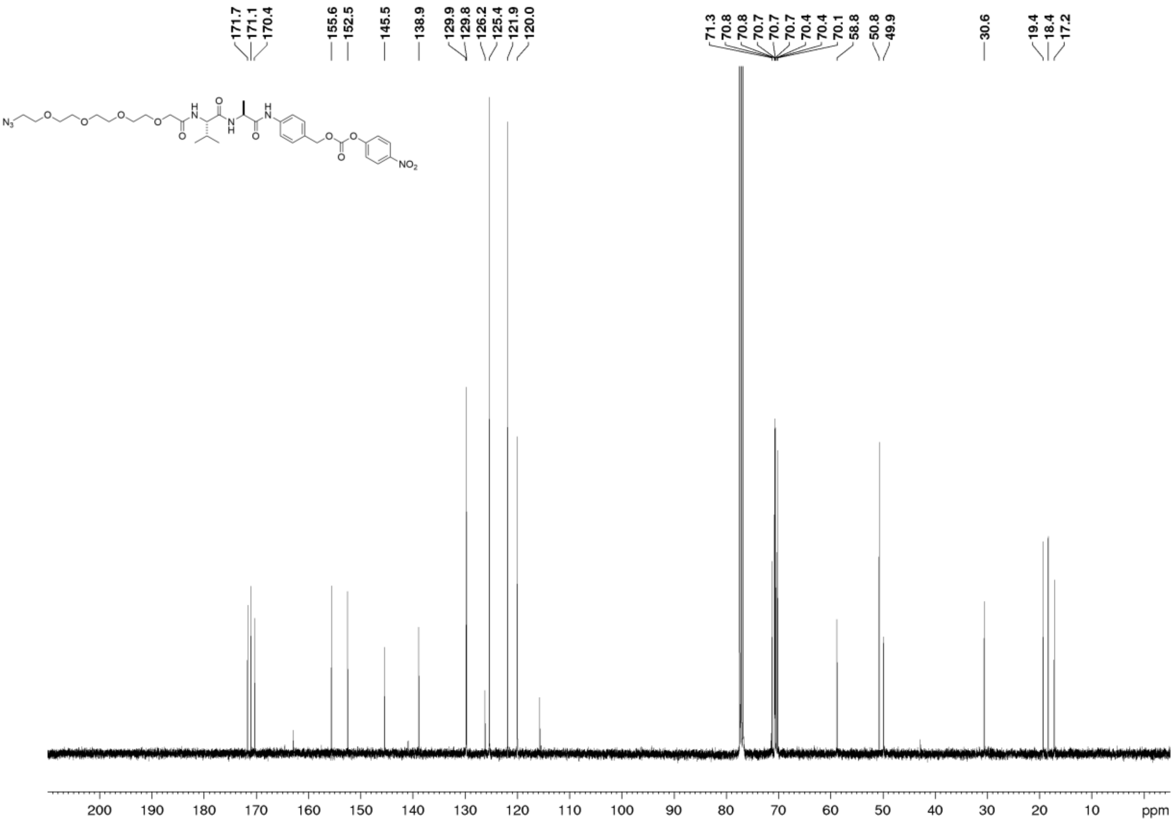

SUPPORTING INFORMATION

Hemiasterlin ethyl ester trifluoroacetate salt (20)

^{1}H-NMR

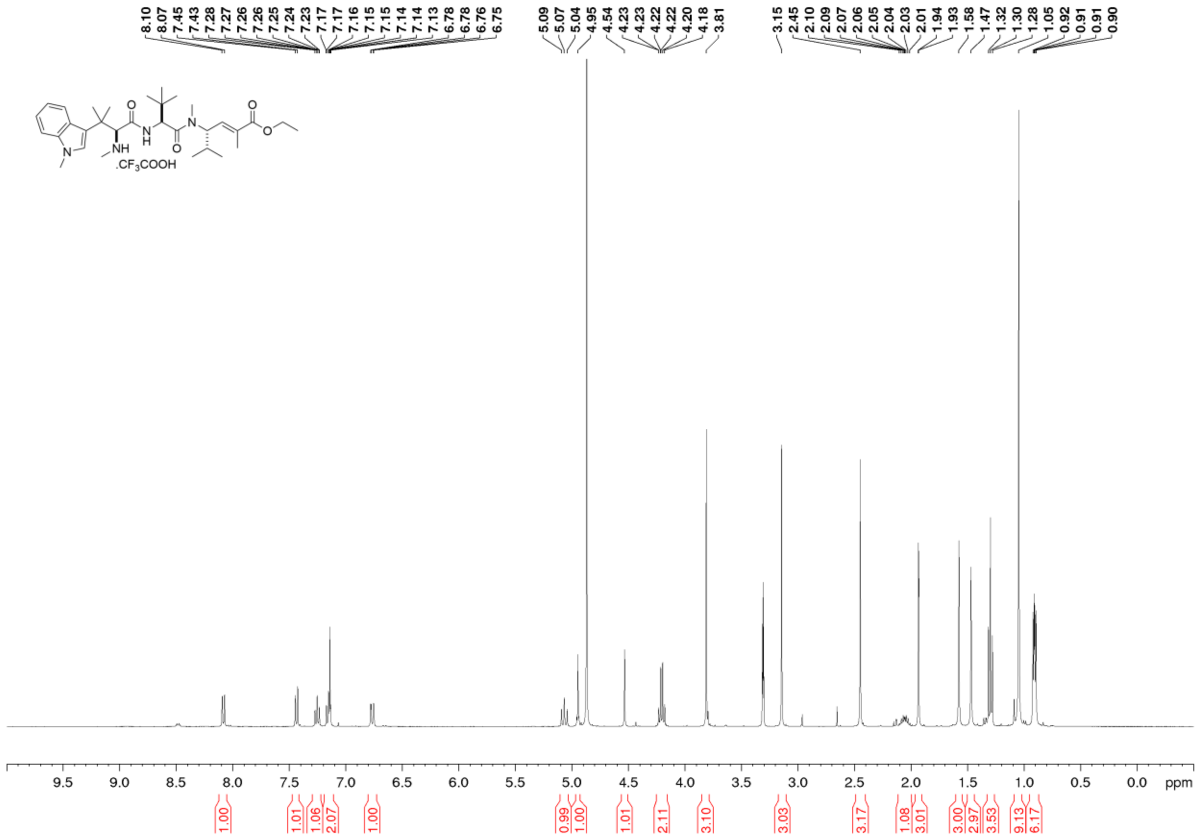

^{13}C-NMR

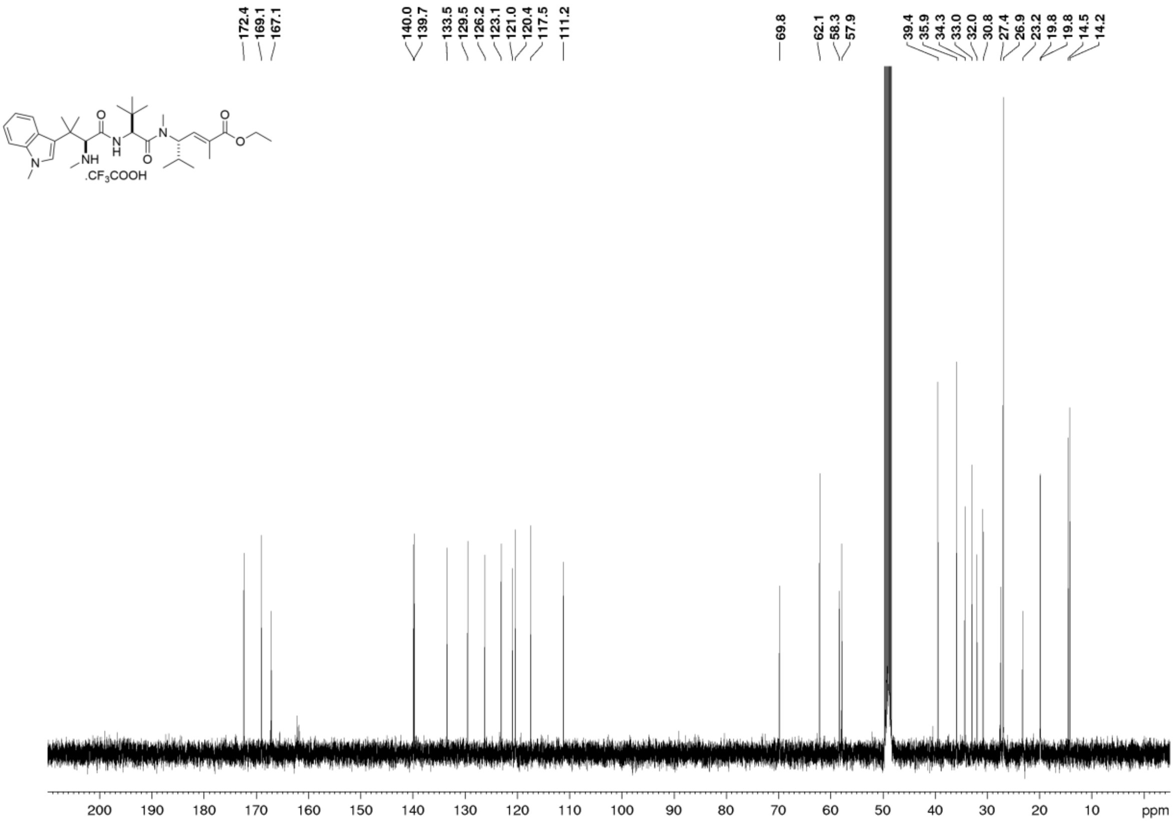

^{19}F-NMR

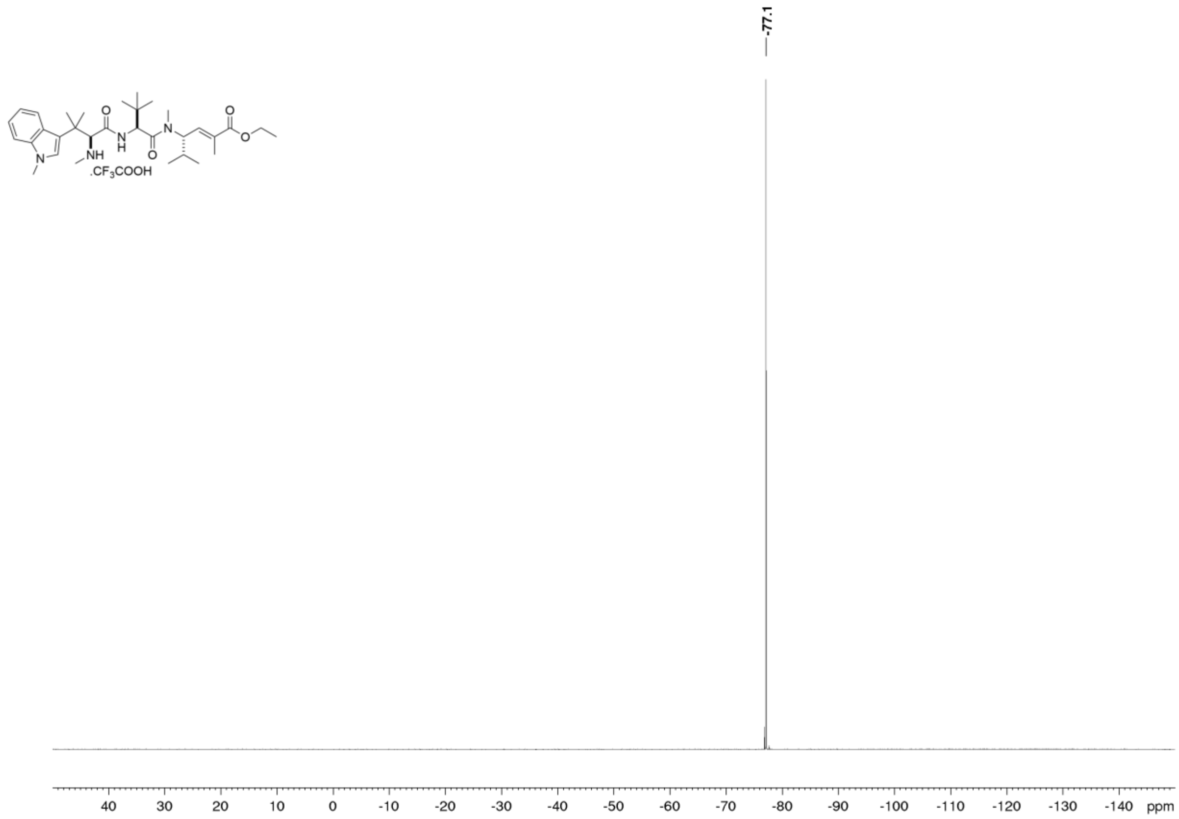
-77.1
.CF3COOH
40 30 20 10 0 -10 -20 -30 -40 -50 -60 -70 -80 -90 -100 -110 -120 -130 -140 ppm

N$_3$-PEG$_4$-Val-Ala-PABC-Hemiasterlin-OEt (23)
^{1}H-NMR

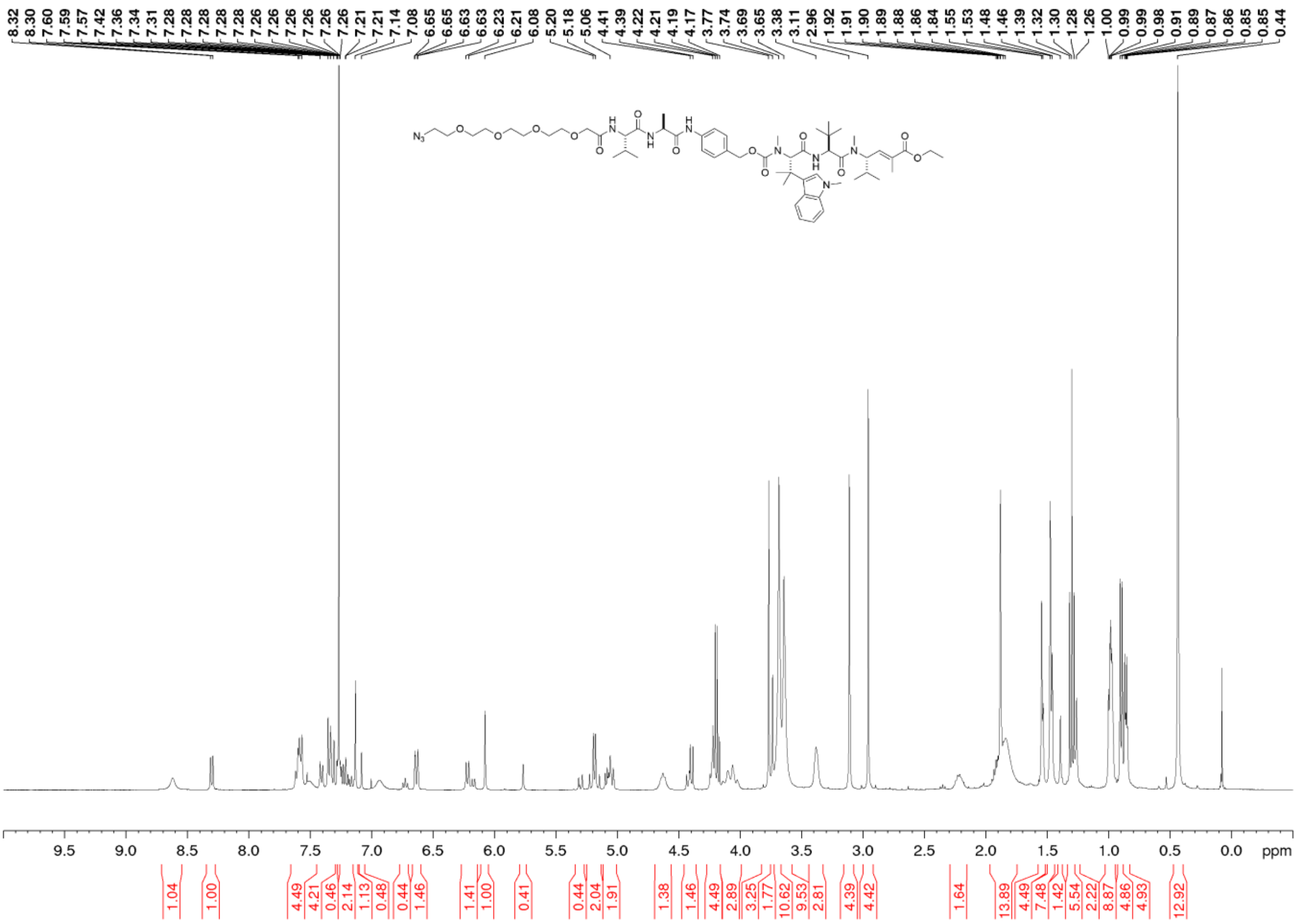

Taltobulin ethyl ester trifluoroacetate salt (21)

^{1}H-NMR

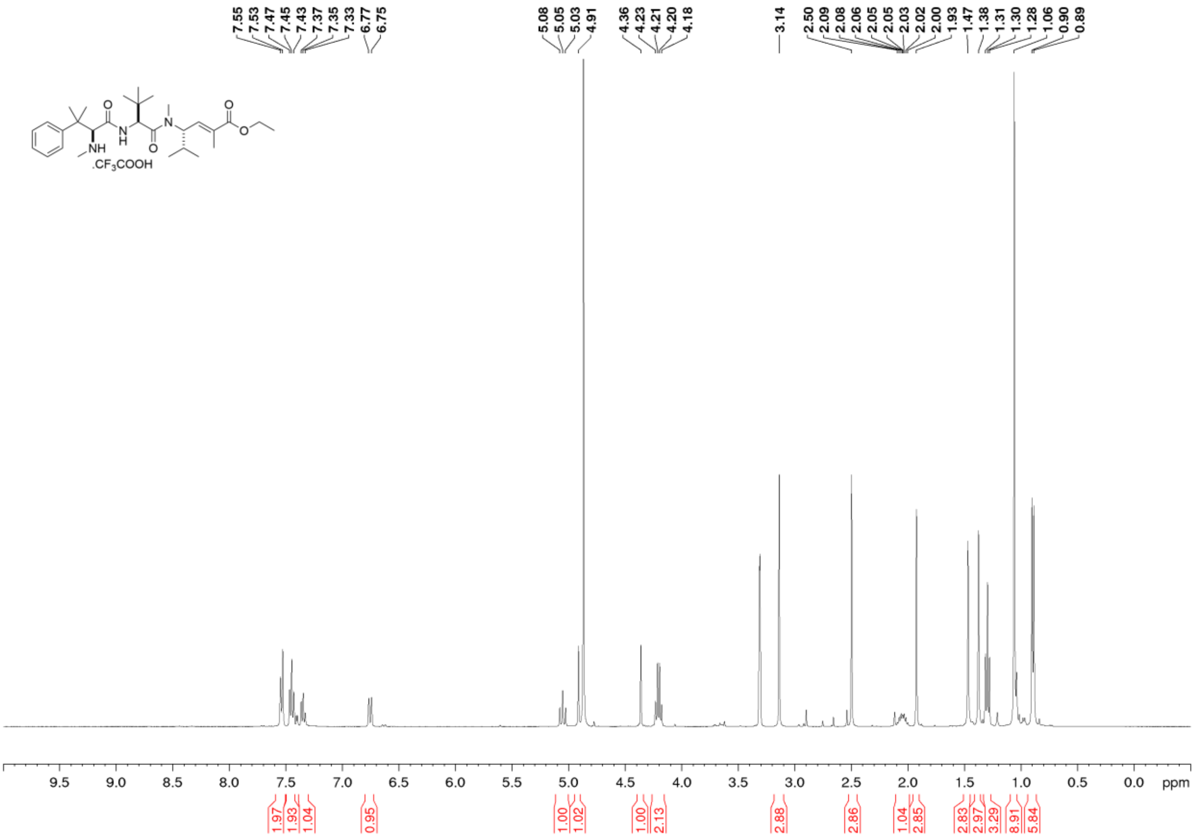

^{13}C-NMR

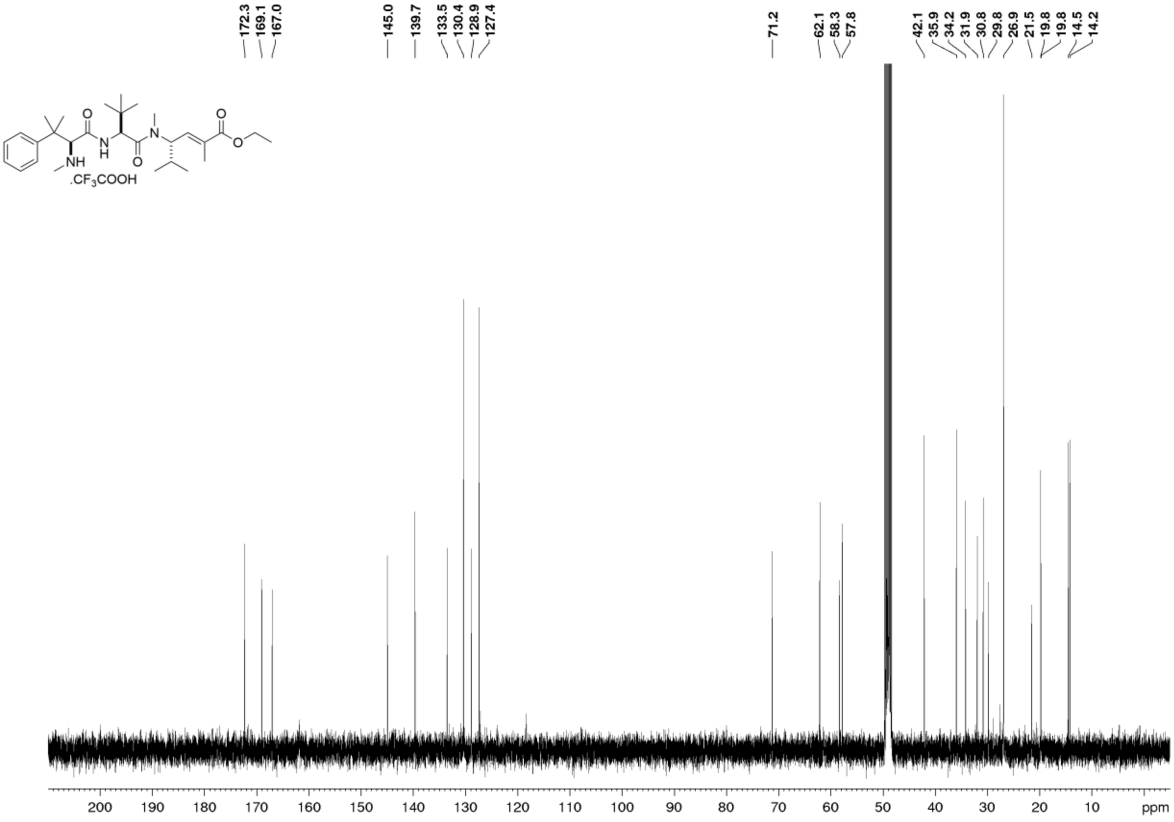

^{19}F-NMR

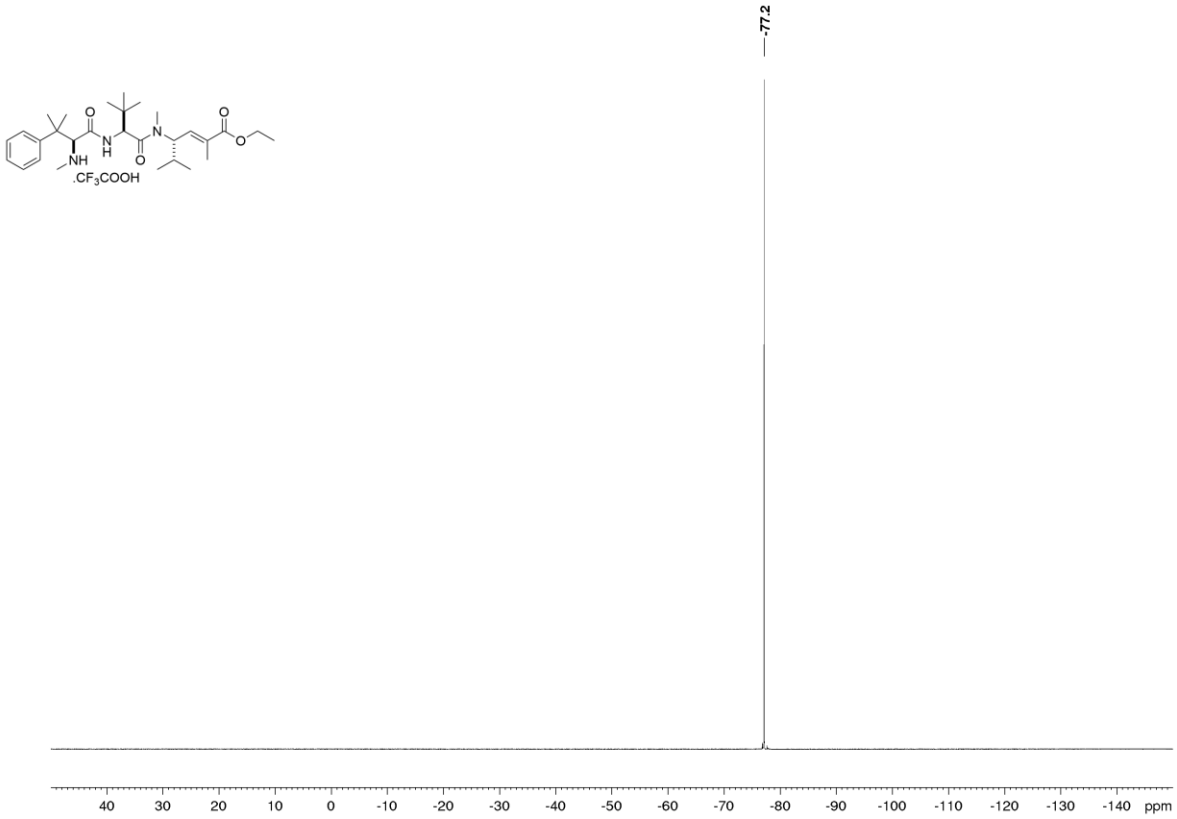
NH
H
N
O
.CF3COOH
-77.2
40
30
20
10
0
-10
-20
-30
-40
-50
-60
-70
-80
-90
-100
-110
-120
-130
-140
ppm

N_3-PEG$_4$-Val-Ala-PABC-Taltobulin-OEt (24)

^{1}H-NMR

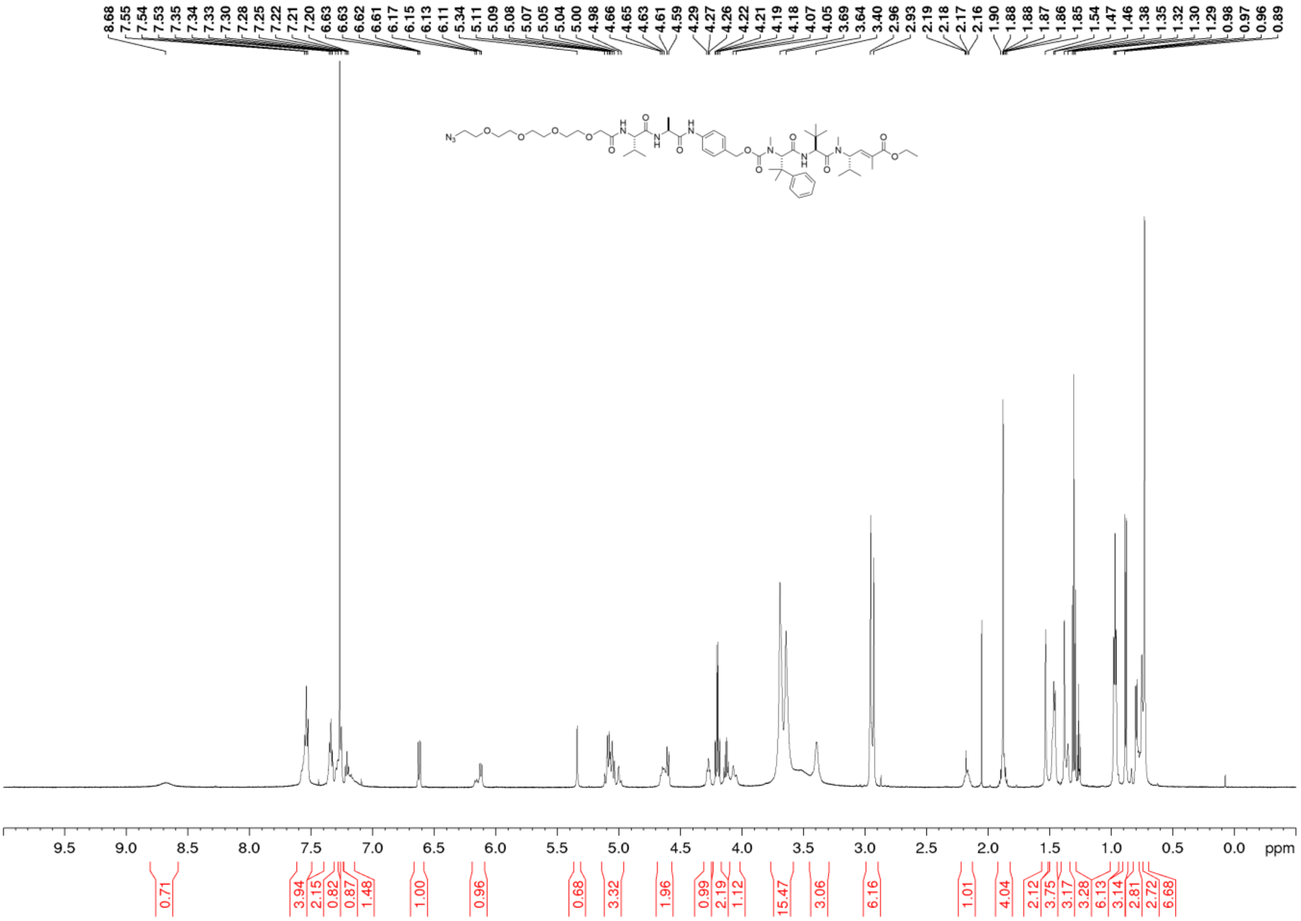

(*S*)-3,3-dimethyl-2-((*S*)-3-methyl-3-(1-methyl-1*H*-indol-3-yl)-2-(2,2,2-trifluoro-*N*-methylacetamido)butanamido)butanoic acid (S13a)

^{1}H-NMR

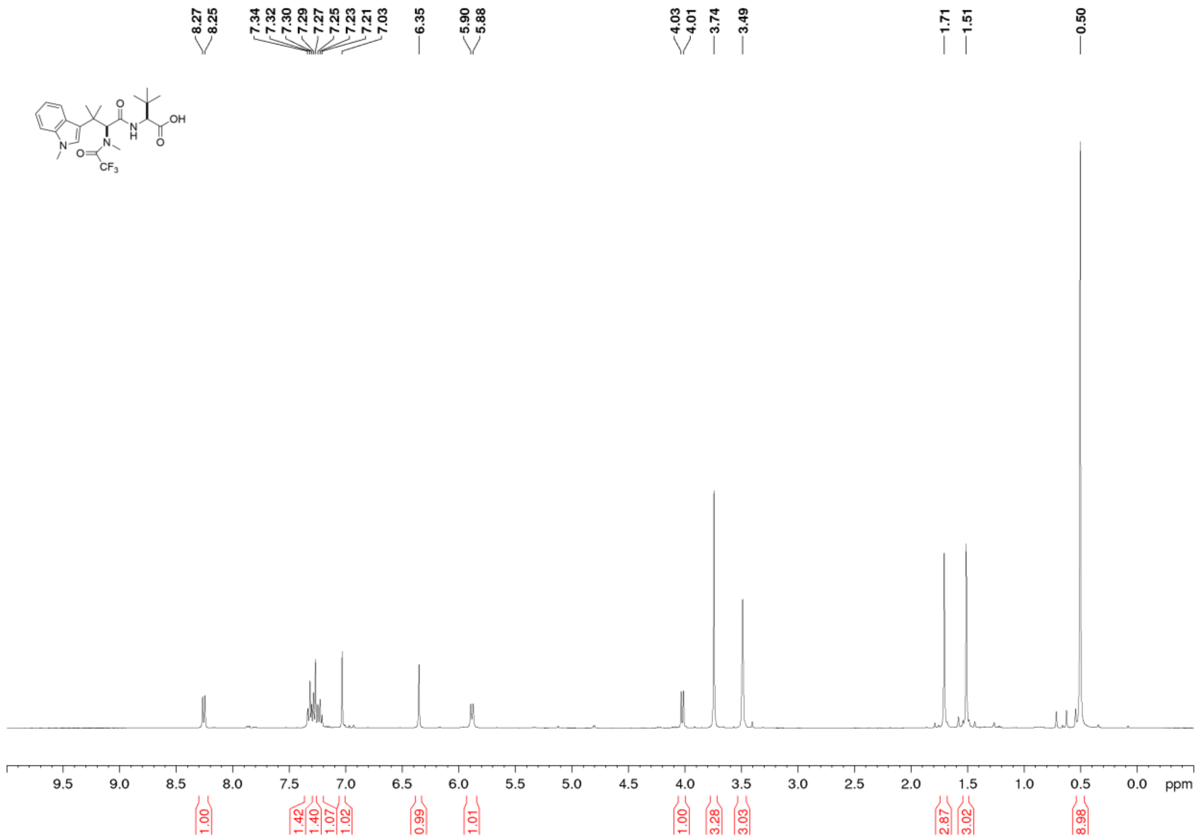

^{13}C-NMR

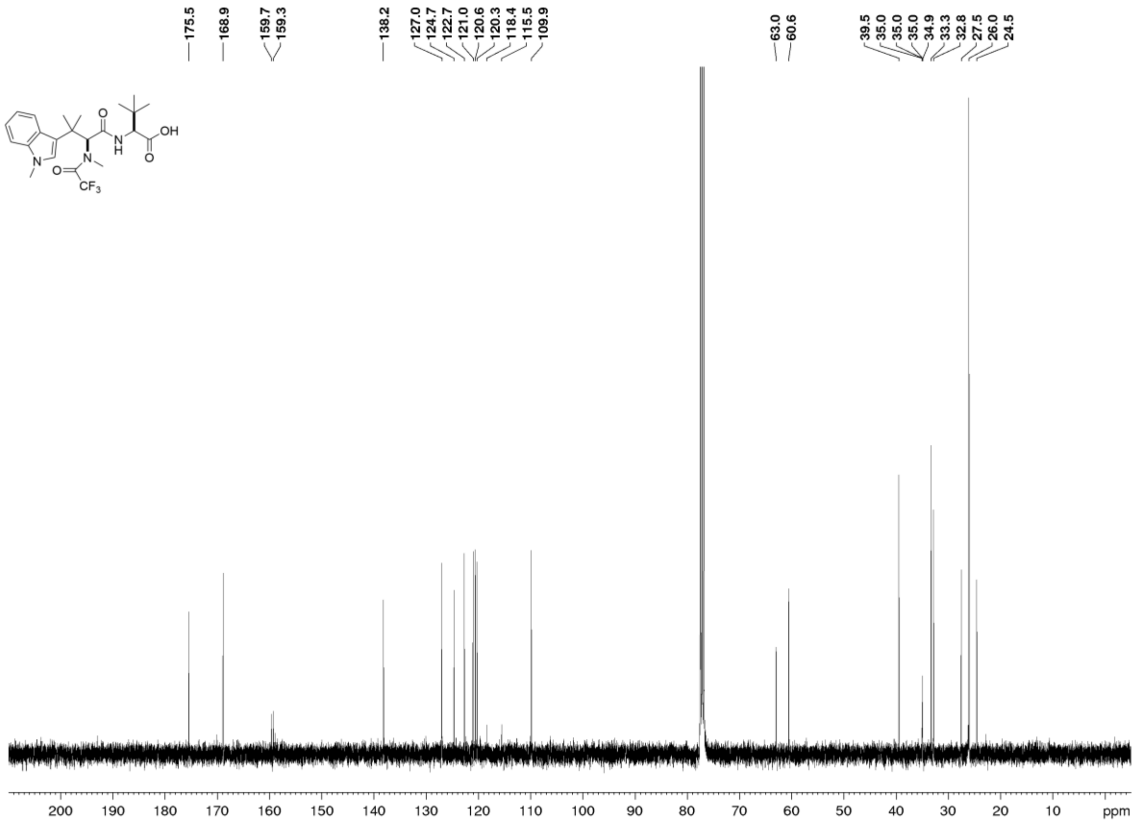

^{19}F-NMR

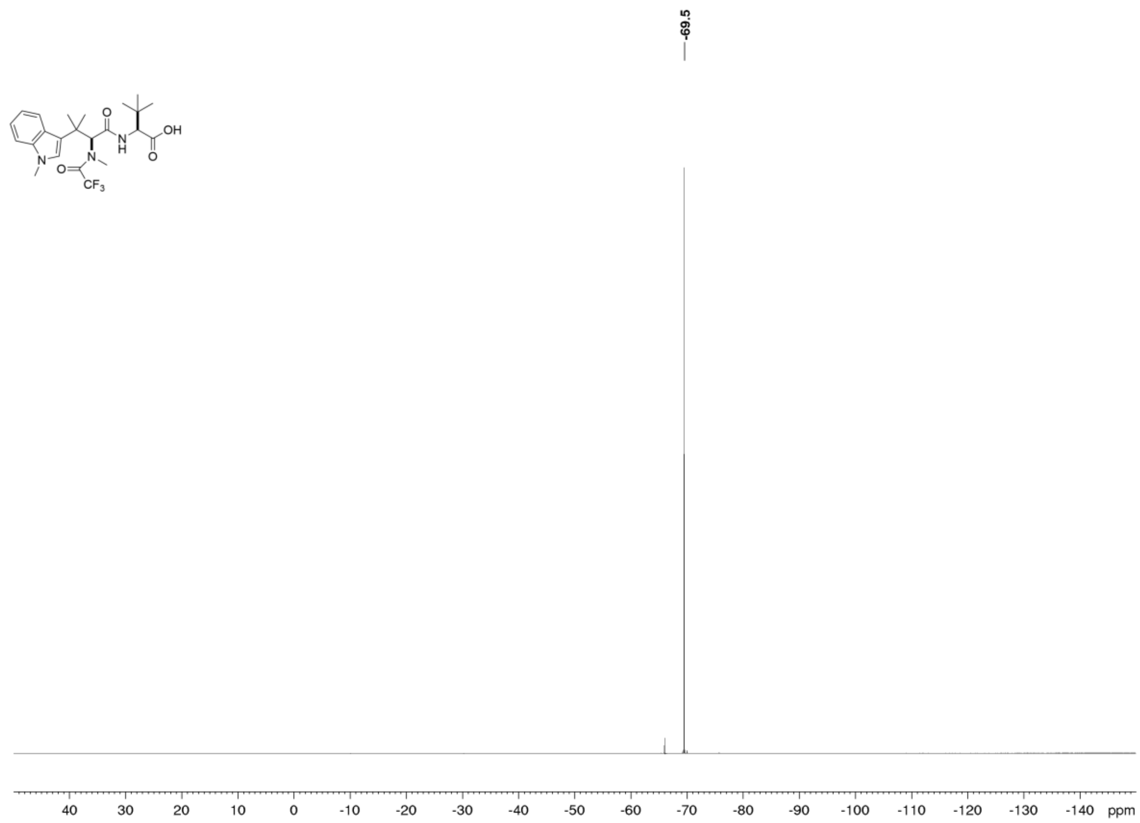
-69.5
OH
O
N
H
N
O
CF3
ppm
40
30
20
10
0
-10
-20
-30
-40
-50
-60
-70
-80
-90
-100
-110
-120
-130
-140

(*S*)-3,3-dimethyl-2-((*R*)-3-methyl-3-(1-methyl-1*H*-indol-3-yl)-2-(2,2,2-trifluoro-*N*-methylacetamido)butanamido)butanoic acid (S13b)

^{1}H-NMR

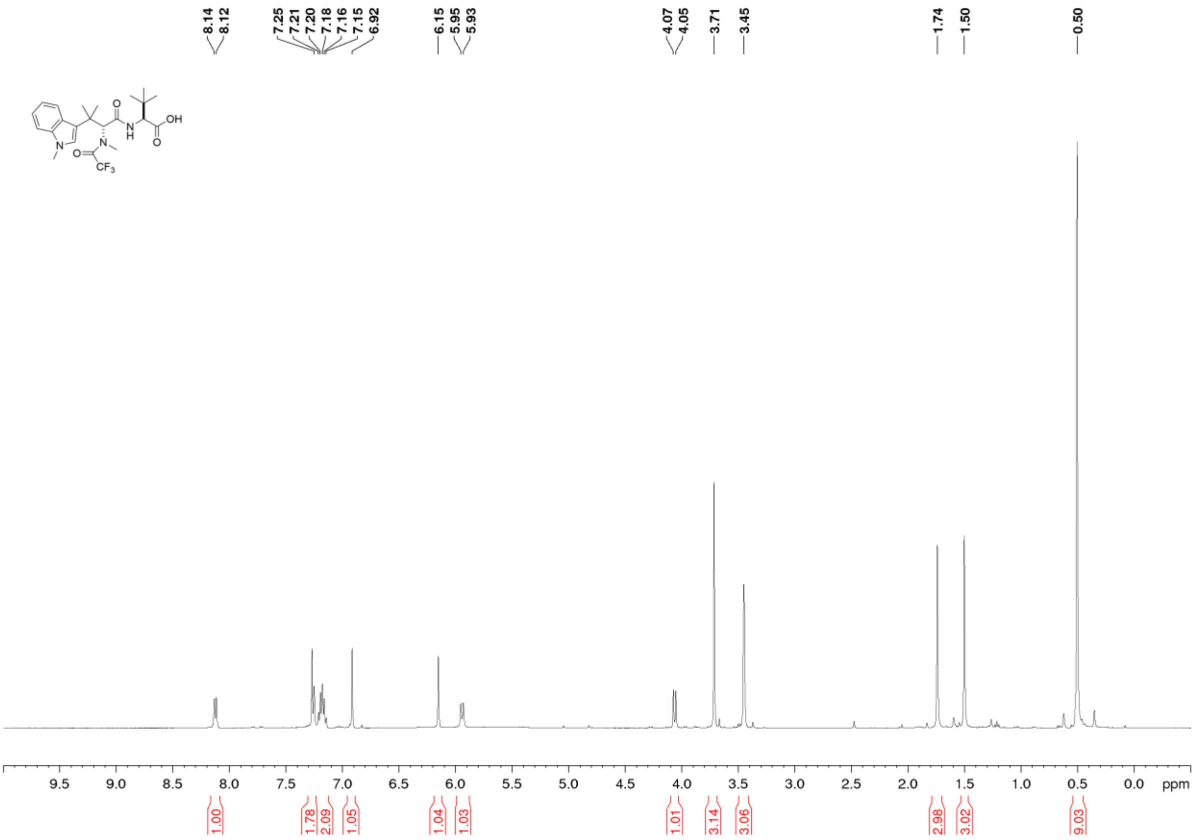

^{13}C-NMR

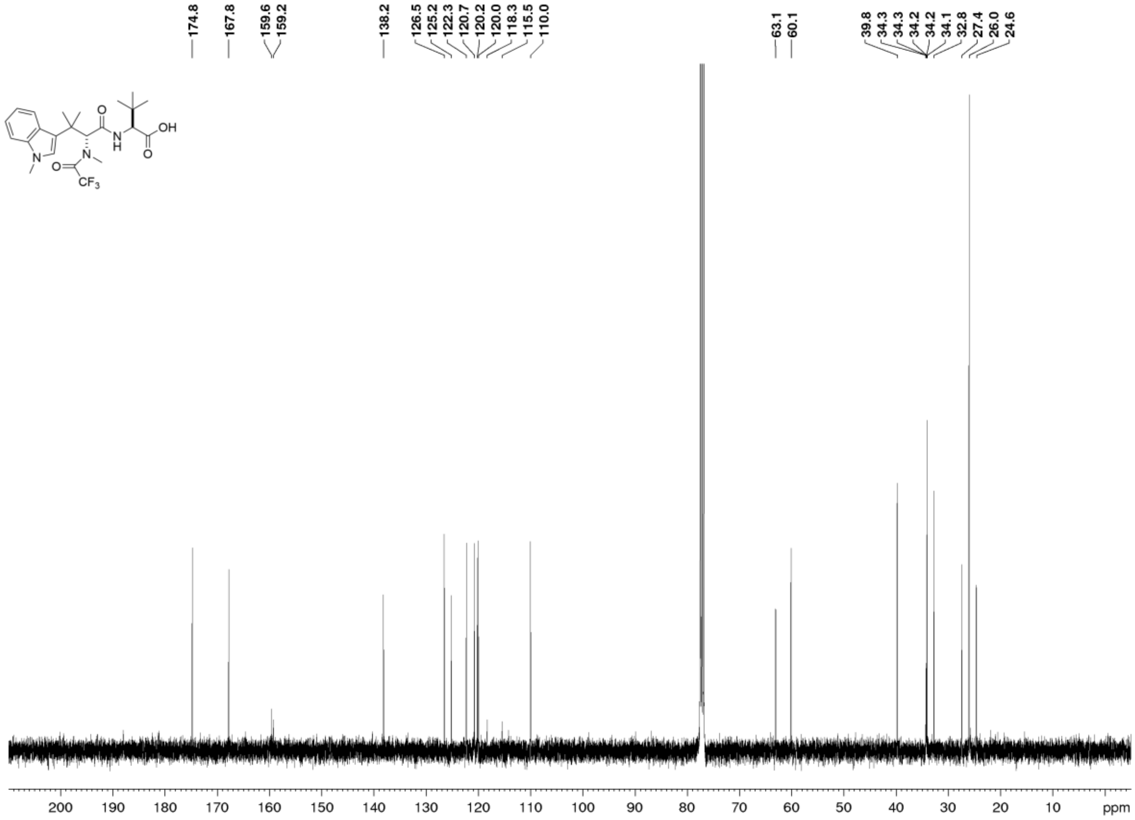

^{19}F-NMR

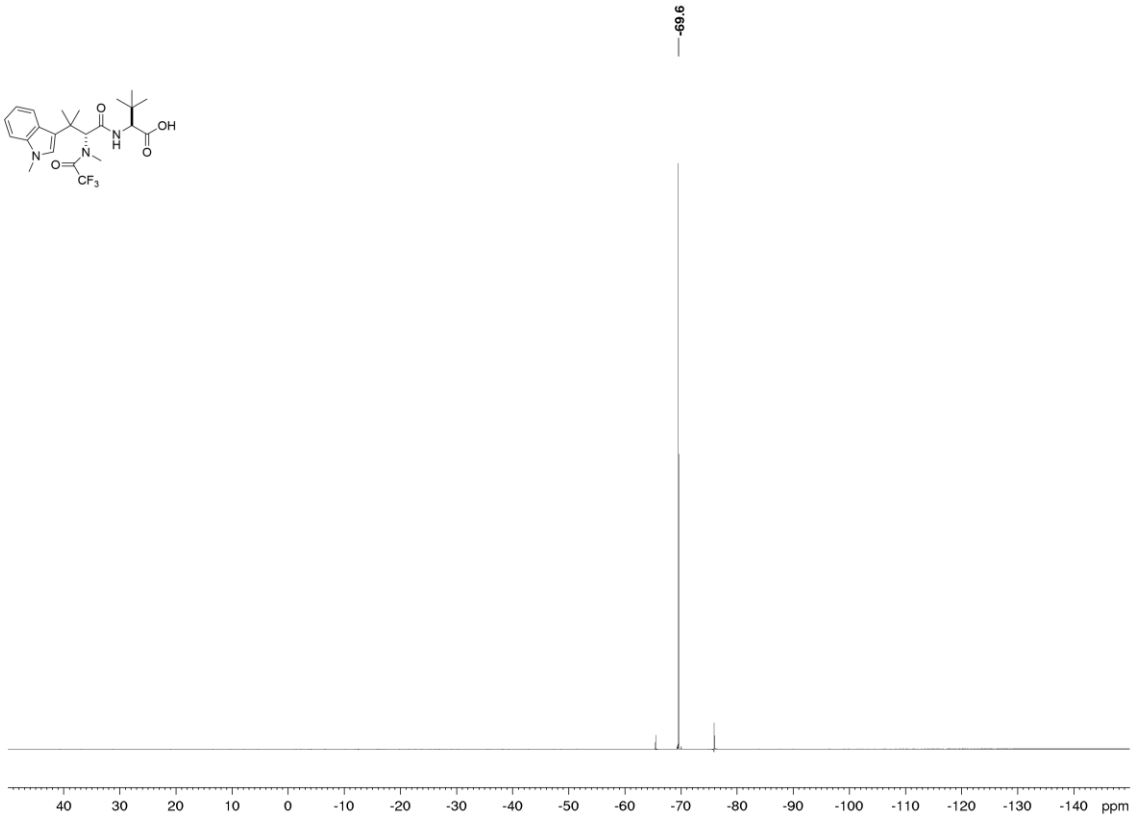
-69.6
OH
O
N
H
N
O
CF3
40 30 20 10 0 -10 -20 -30 -40 -50 -60 -70 -80 -90 -100 -110 -120 -130 -140 ppm

Oxazolone S14 (as an inseparable diastereomeric mixture of 1.7:1 *dr*)

^{1}H-NMR

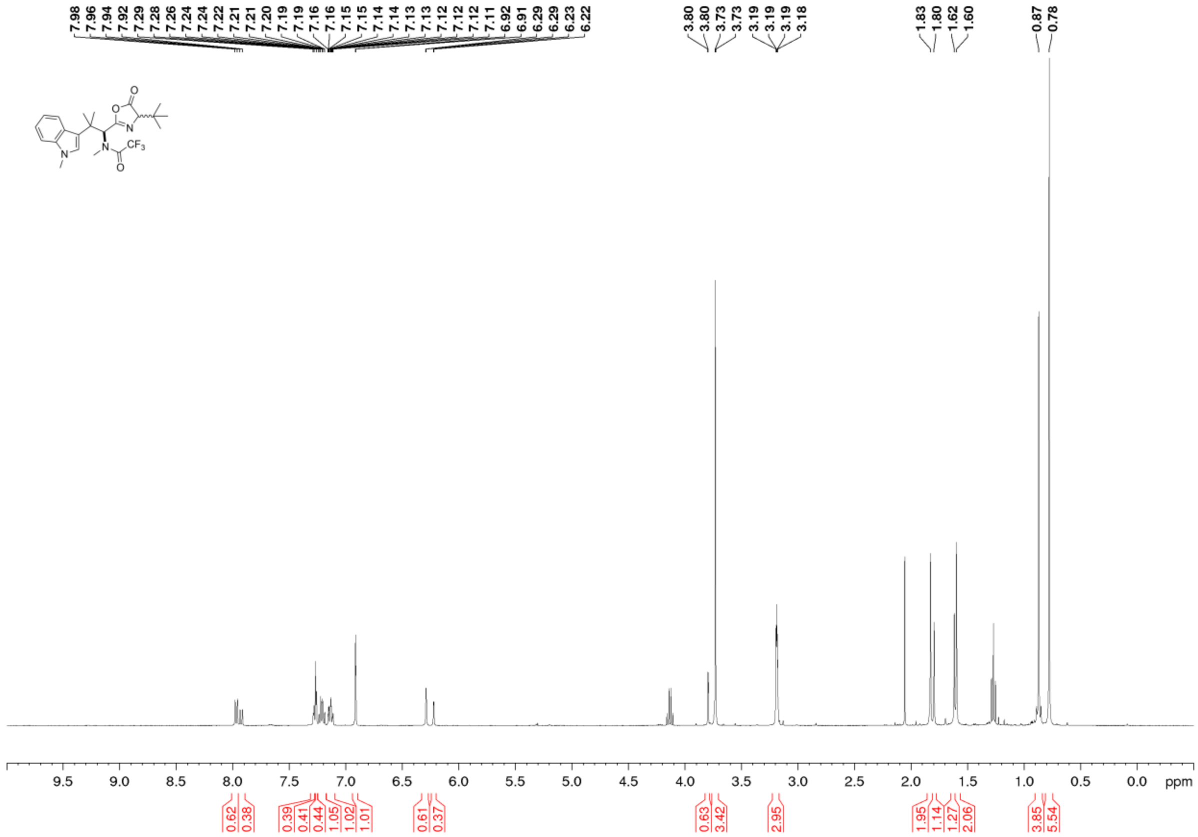

^{13}C-NMR

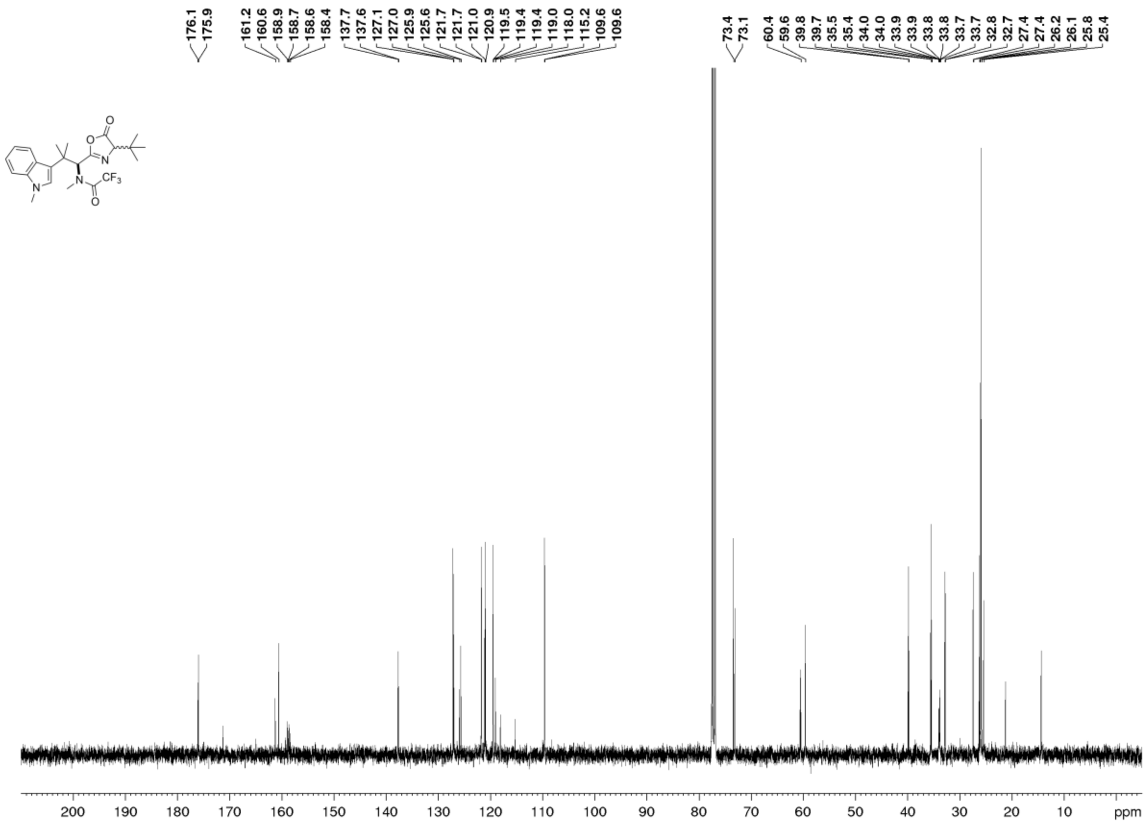

^{19}F-NMR

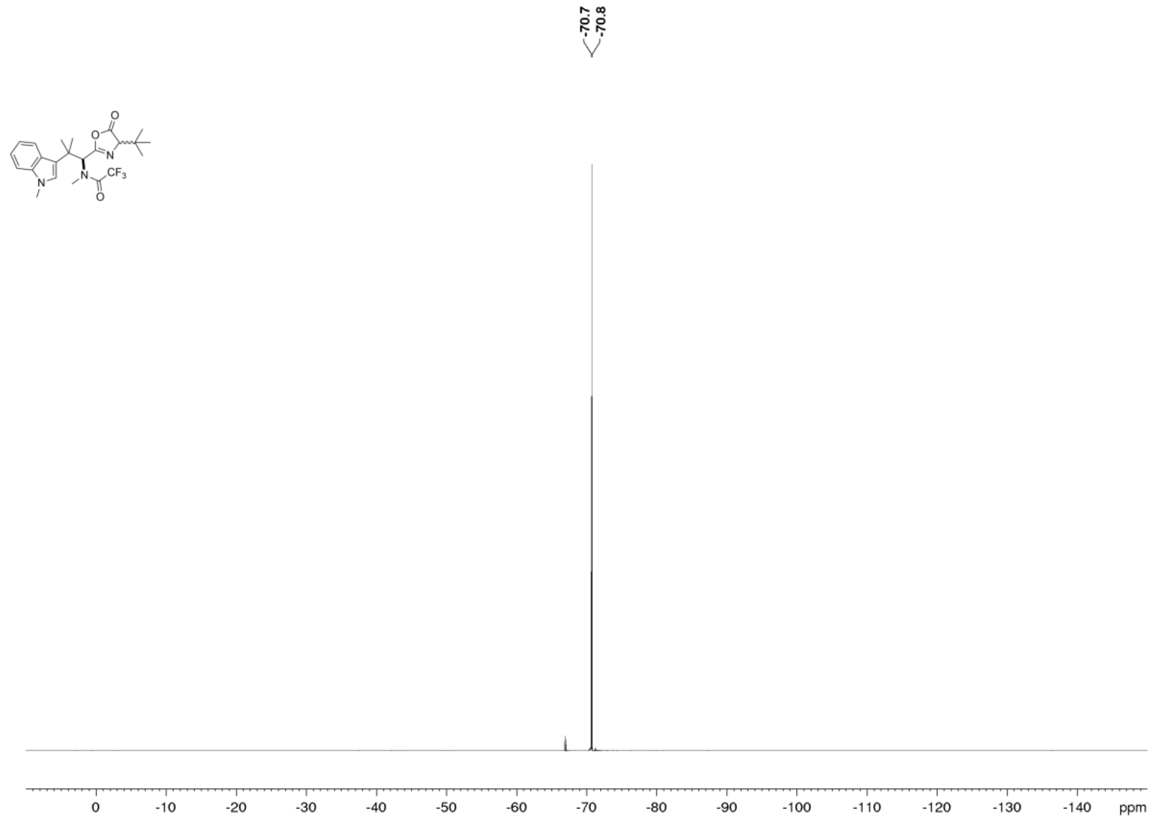
-70.7
-70.8
O
O
N
N
CF3
O
N
0
-10
-20
-30
-40
-50
-60
-70
-80
-90
-100
-110
-120
-130
-140
ppm

Analytical HPLC traces

All compounds were run at 5-95%B over 15 minutes gradient. The traces show the absorbance at 220 nm. All peaks at $t_R \approx 2$ min correspond to DMSO.

Hemiasterlin trifluoroacetate salt (1•TFA)

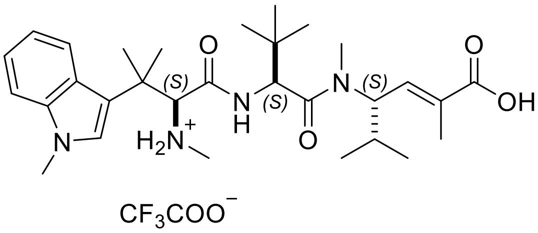

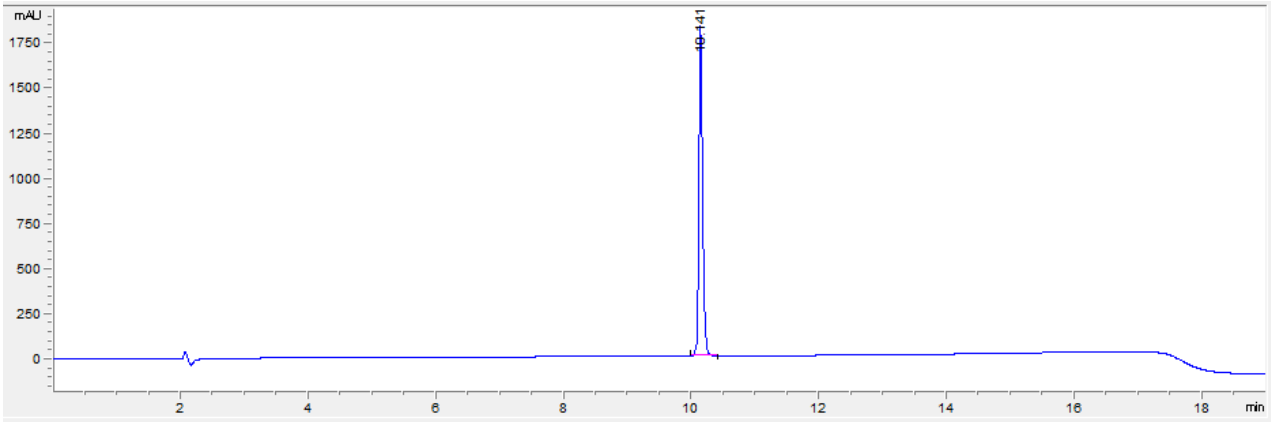

***epi*-hemiasterlin trifluoroacetate salt (1b•TFA)**

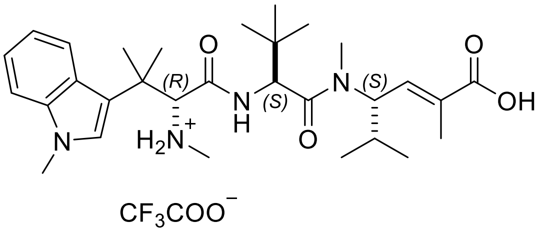

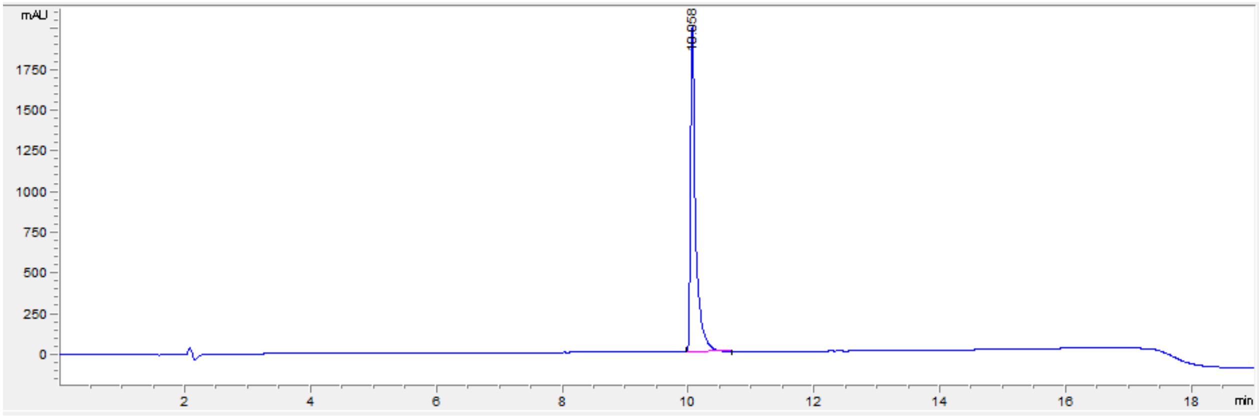

Taltobulin trifluoroacetate salt (8•TFA)

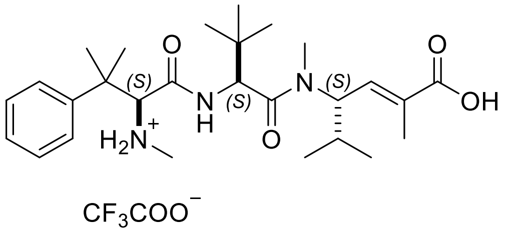

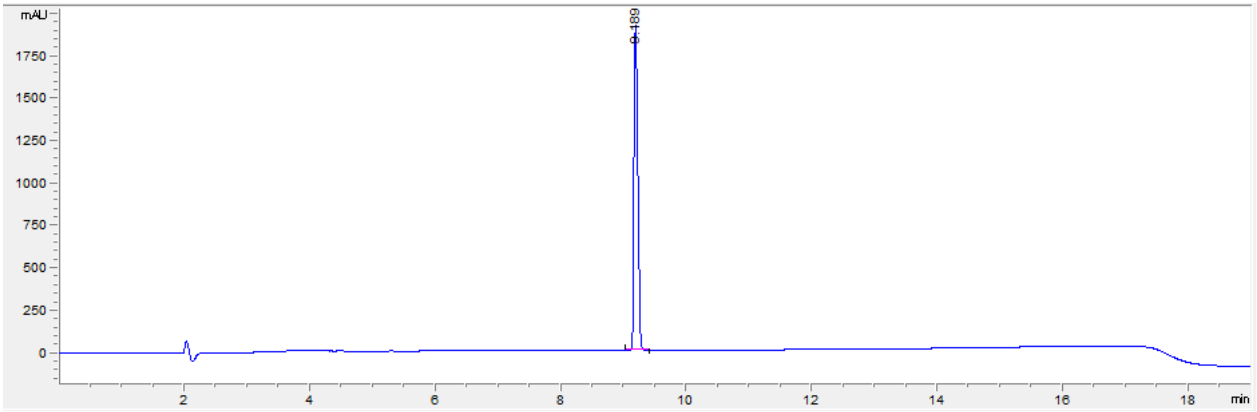

***epi*-Taltobulin trifluoroacetate salt (8b•TFA)**

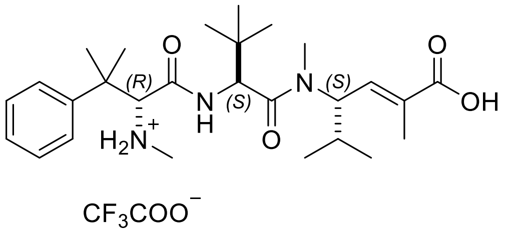

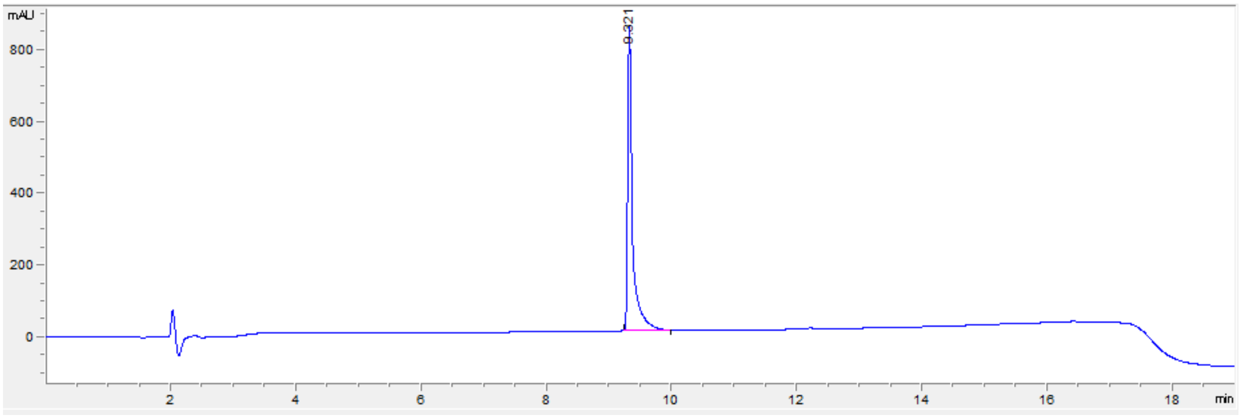

H-Val-Ala-PABA•TFA (S9)

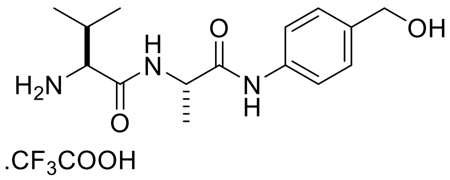

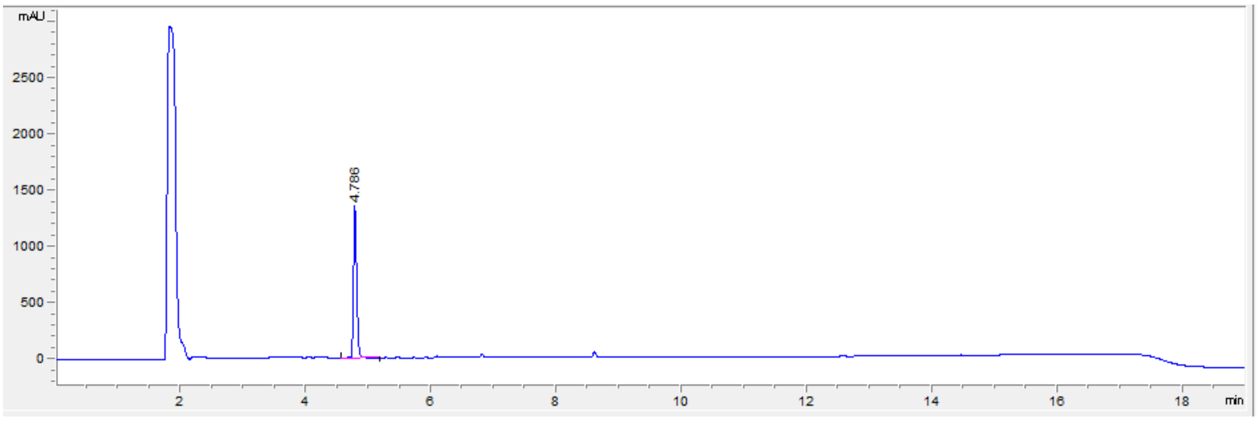

N_3-PEG_4-Val-Ala-PABA (S10)

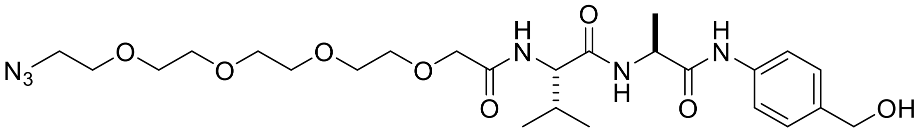

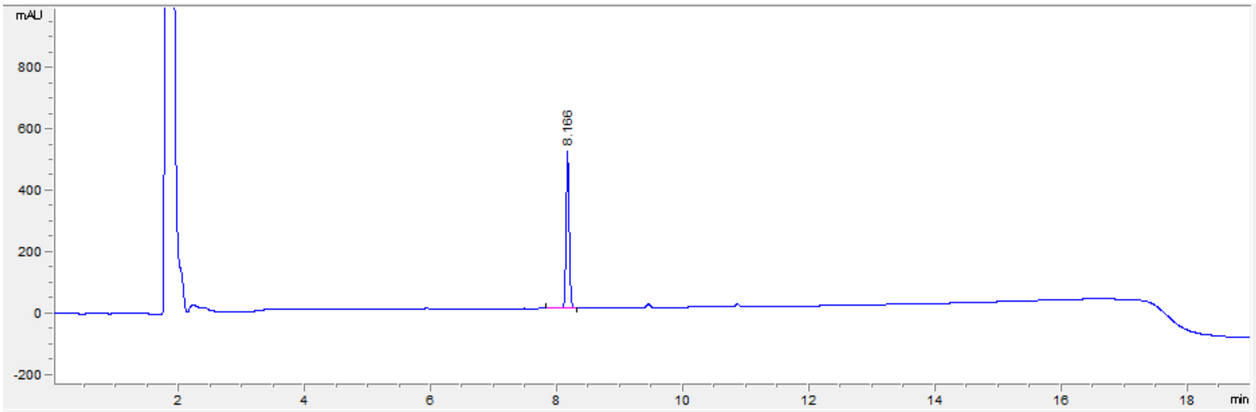

Hemiasterlin ethyl ester trifluoroacetate salt (20)

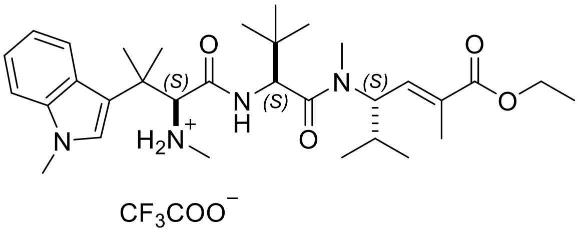

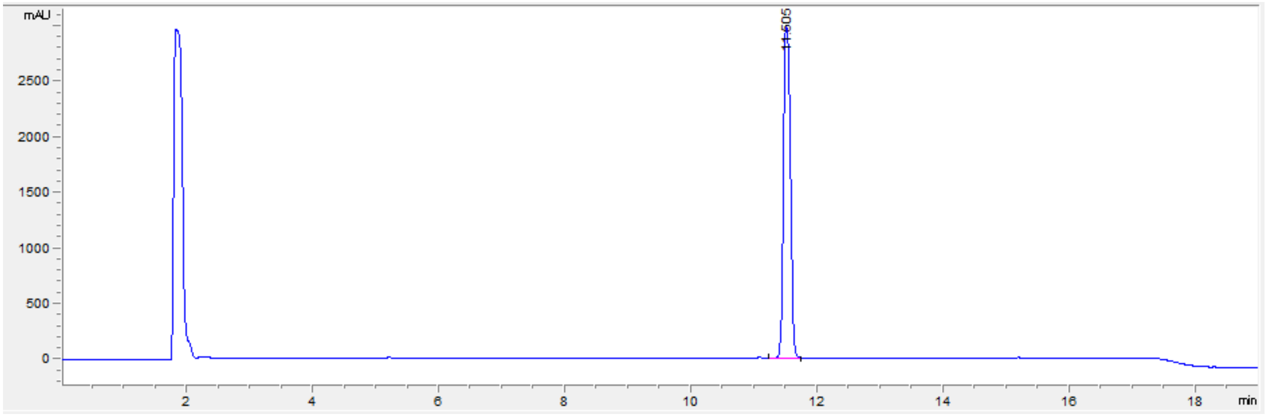

N3-PEG4-Val-Ala-PABC-hemiasterlin-OEt (23)

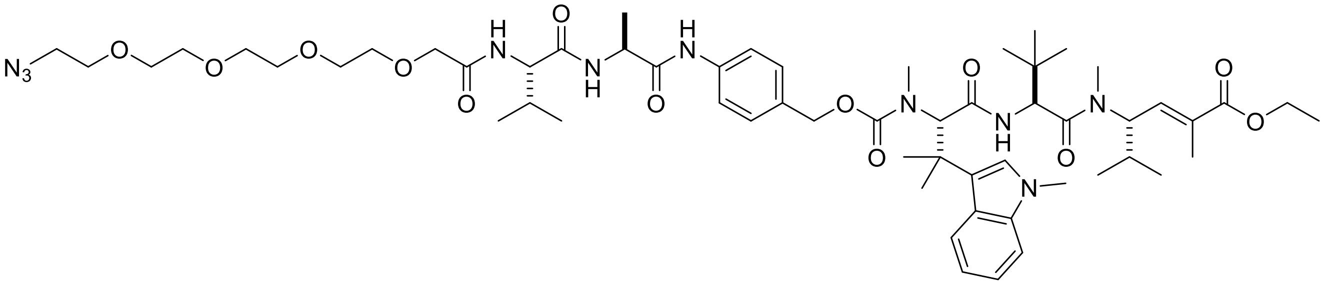

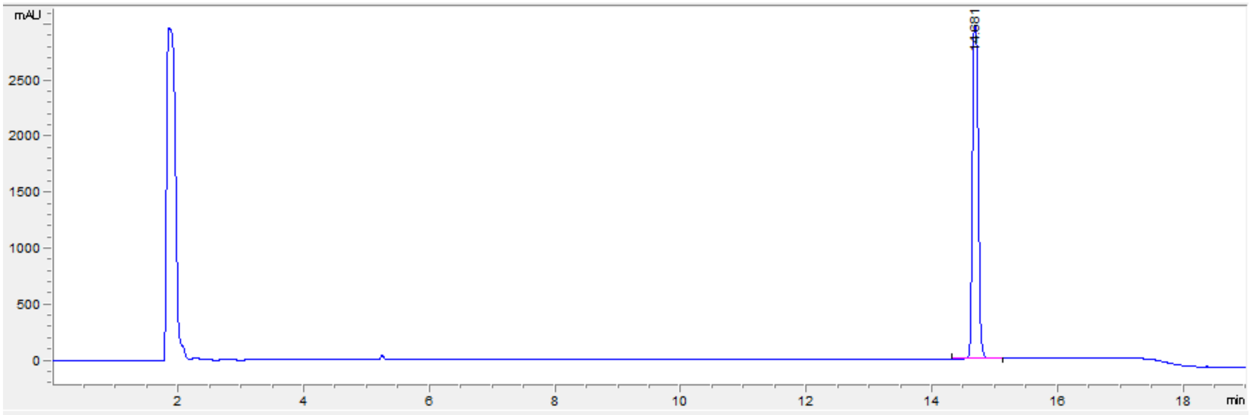

Taltobulin ethyl ester trifluoroacetate salt (21)

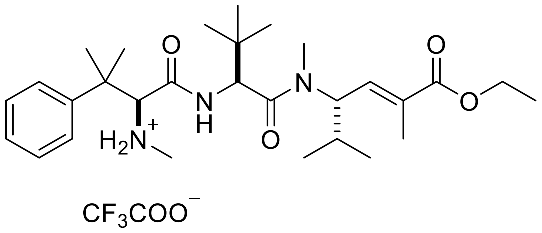

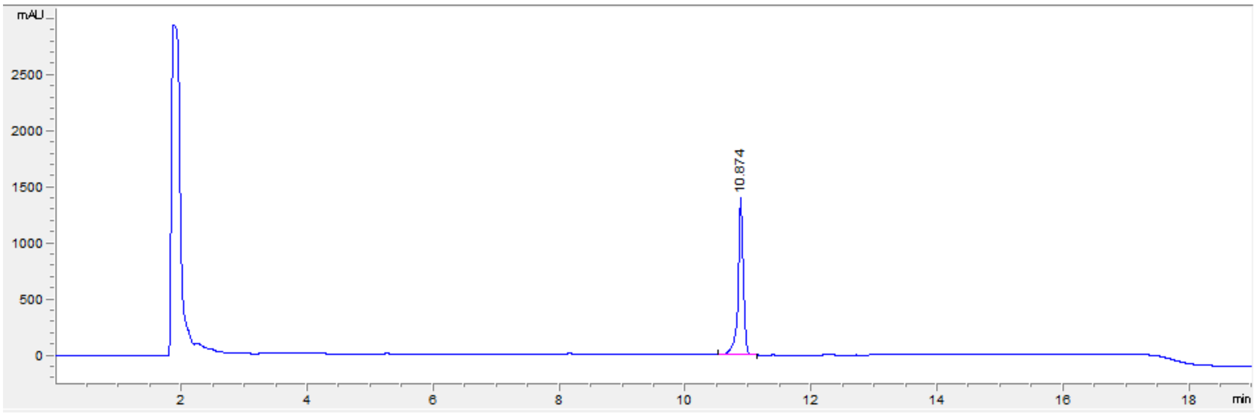

N_3-PEG$_4$-Val-Ala-PABC-taltobulin-OEt (24)

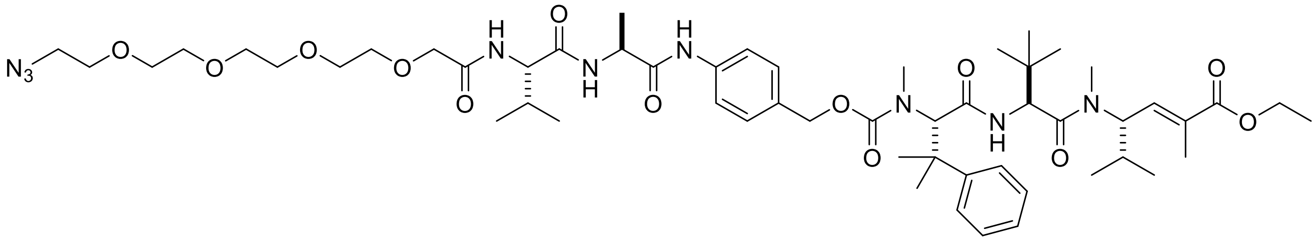

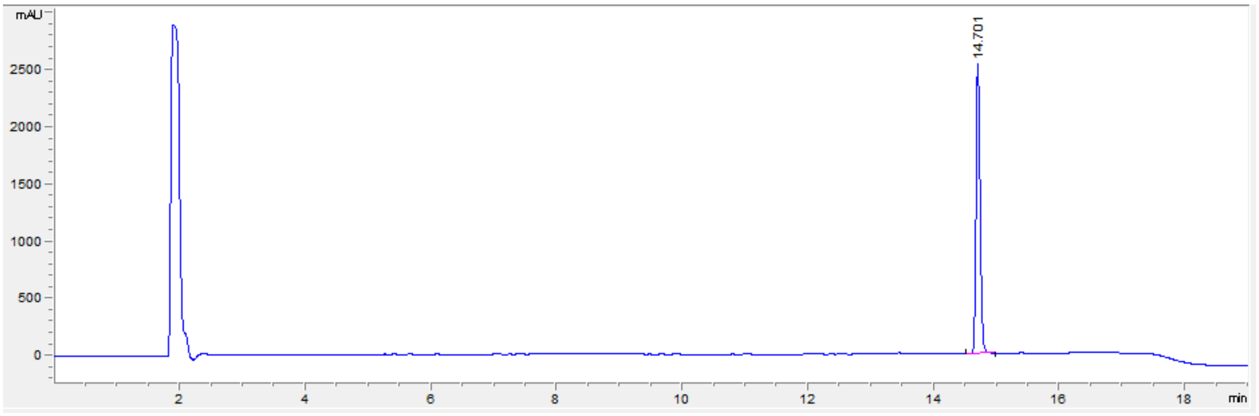

H-PEG2-Glu3-PEG2-propargylGly-NH2•TFA (S11)

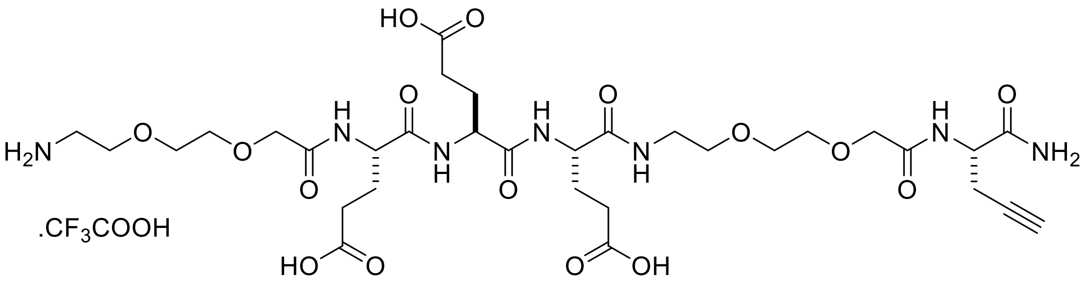

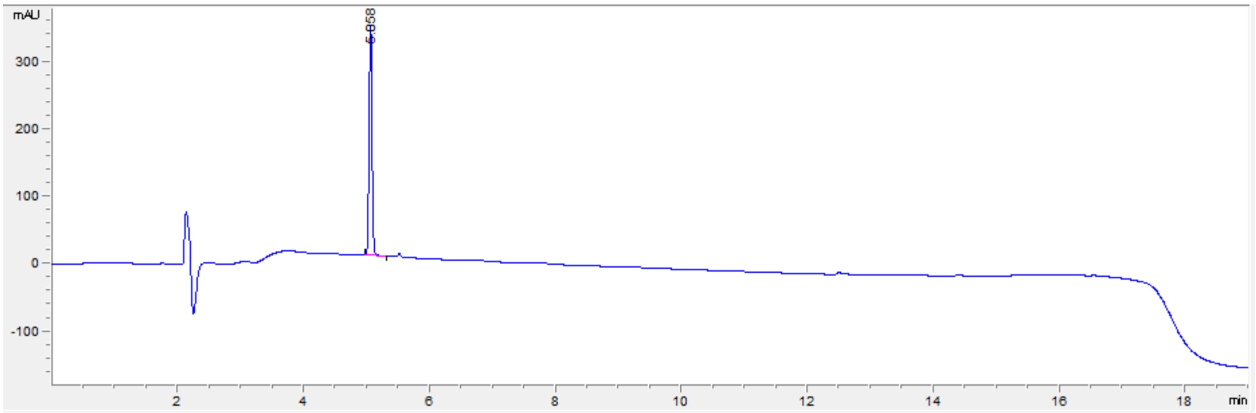

DVP-PEG2-Glu3-PEG2-propargylGly-NH2 (25)

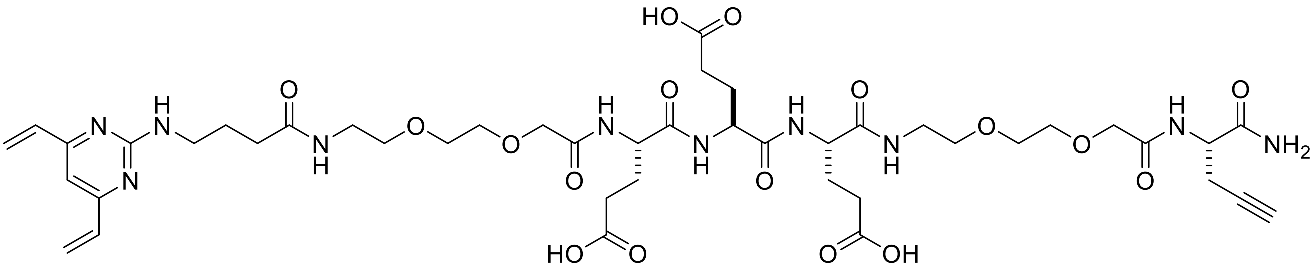

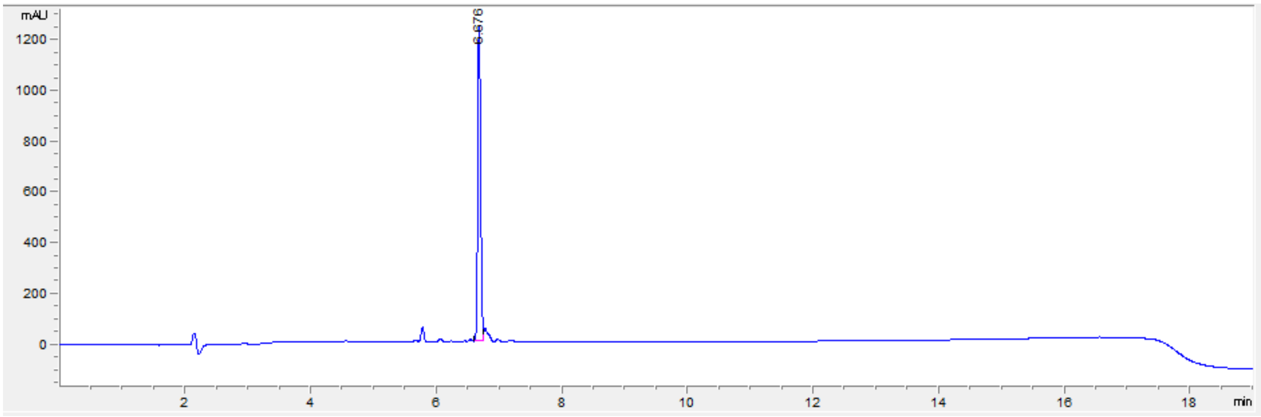

DVP-PEG$_2$-Glu$_3$-PEG$_2$-triazole-PEG$_4$-Val-Ala-PABC-hemiasterlin ammonium salt (26)

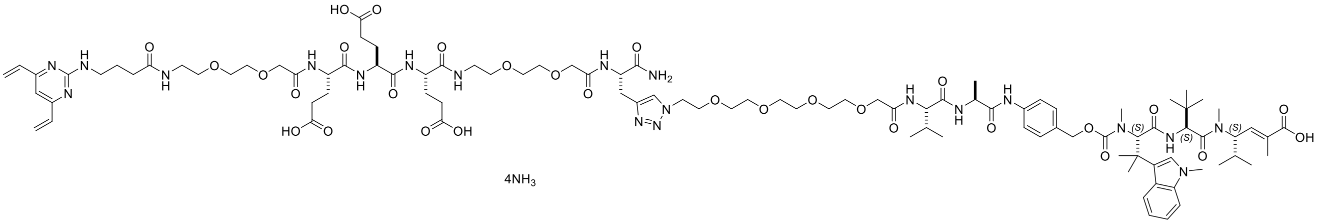

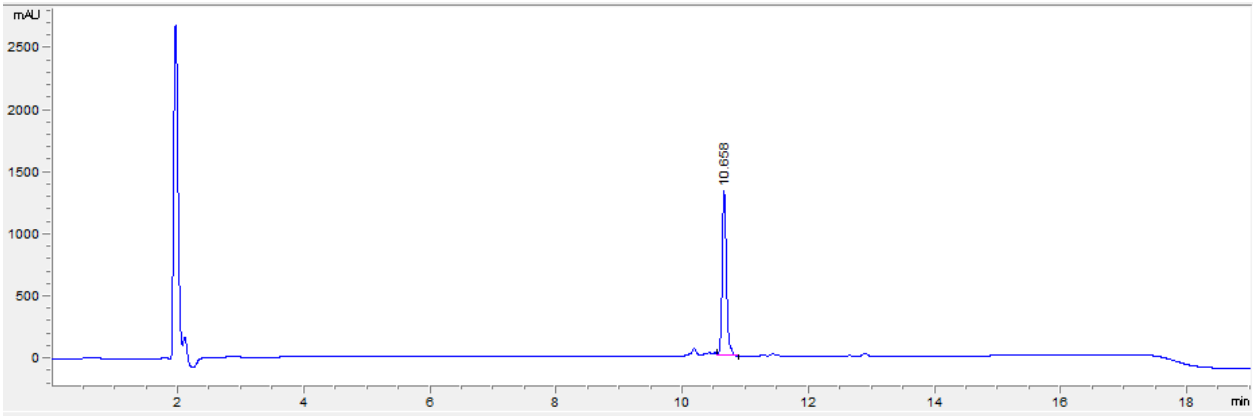

DVP-PEG$_2$-Glu$_3$-PEG$_2$-triazole-PEG$_4$-Val-Ala-PABC-taltobulin ammonium salt (27)

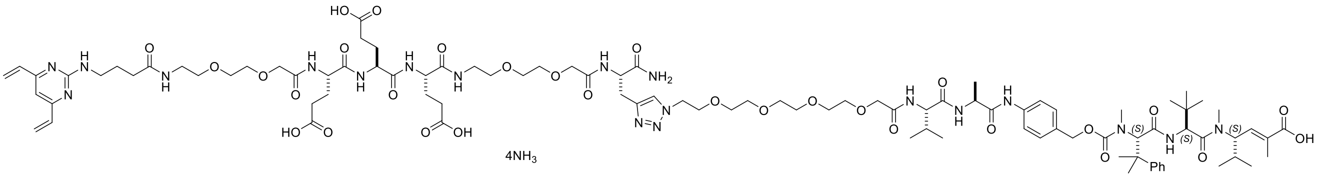

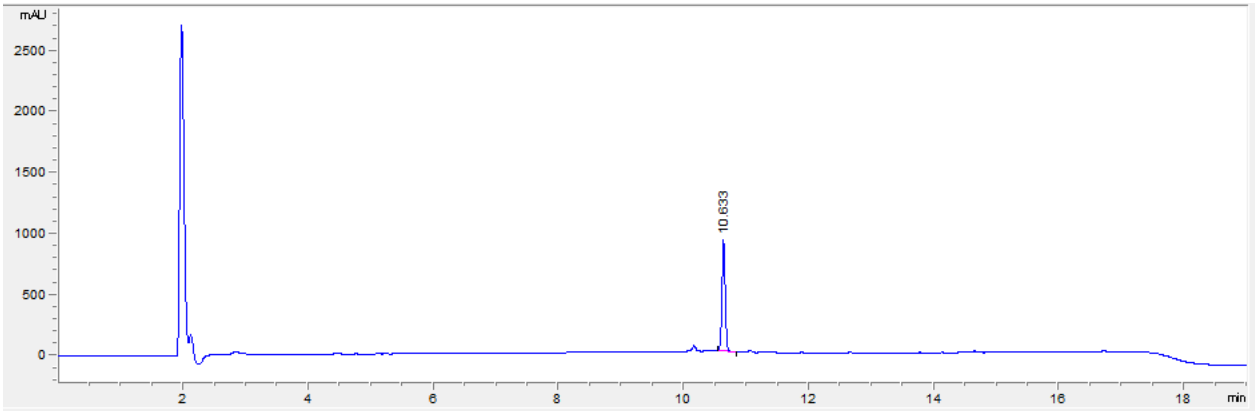

References

[1] H. E. Gottlieb, V. Kotlyar, A. Nudelman, *J. Org. Chem.* **1997**, *62*, 7512–7515.

[2] S. Ackermann, H.-G. Lerchen, D. Häbich, A. Ullrich, U. Kazmaier, *Beilstein J. Org. Chem.* **2012**, *8*, 1652–1656.

[3] J. Zhu, X. Wu, S. J. Danishefsky, *Tetrahedron Lett.* **2009**, *50*, 577–579.

[4] J. A. Nieman, J. E. Coleman, D. J. Wallace, E. Piers, L. Y. Lim, M. Roberge, R. J. Andersen, *J. Nat. Prod.* **2003**, *66*, 183–199.

[5] I. Coldham, S. Raimbault, D. T. E. Whittaker, P. T. Chovatia, D. Leonori, J. J. Patel, N. S. Sheikh, *Chem. - Eur. J.* **2010**, *16*, 4082–4090.

[6] X. Cai, A. Keshavarz, J. D. Omaque, B. J. Stokes, *Org. Lett.* **2017**, *19*, 2626–2629.

[7] J. D. Bargh, S. J. Walsh, A. Isidro-Llobet, S. Omarjee, J. S. Carroll, D. R. Spring, *Chem. Sci.* **2020**, *11*, 2375–2380.

[8] G. Lesma, I. Bassanini, R. Bortolozzi, C. Colletto, R. Bai, E. Hamel, F. Meneghetti, G. Rainoldi, M. Stucchi, A. Sacchetti, A. Silvani, G. Viola, *Org. Biomol. Chem.* **2015**, *13*, 11633–11644.

[9] D. W. Carney, K. R. Schmitz, J. V. Truong, R. T. Sauer, J. K. Sello, *J. Am. Chem. Soc.* **2014**, *136*, 1922–1929.

[10] W. Gong, G. Zhang, T. Liu, R. Giri, J. Q. Yu, *J. Am. Chem. Soc.* **2014**, *136*, 16940–16946.

[11] W. Felzmann, S. Brunner, T. Wilhelm, *PROCESS FOR THE SYNTHESIS OF TELAPREVIR, OR PHARMACEUTICALLY ACCEPTABLE SALTS OR SOLVATES AS WELL AS INTERMEDIATE PRODUCTS THEREOF*, **2013**, WO2013/135870.

[12] M. E. Due-Hansen, S. K. Pandey, E. Christiansen, R. Andersen, S. V. F. Hansen, T. Ulven, *Org. Biomol. Chem.* **2016**, *14*, 430–433.

[13] S. J. Walsh, S. Omarjee, W. R. J. D. Galloway, T. T.-L. Kwan, H. F. Sore, J. S. Parker, M. Hyvönen, J. S. Carroll, D. R. Spring, *Chem. Sci.* **2019**, *10*, 694–700.
